# **A Bifurcated Multicomponent Synthesis Approach to Polycyclic Quinazolinones**

**DOI:** 10.1021/acs.joc.2c01561

**Published:** 2022-09-12

**Authors:** Ruixue Xu, Zefeng Wang, Qiang Zheng, Pravin Patil, Alexander Dömling

**Affiliations:** Drug Design Group, Department of Pharmacy, University of Groningen, Groningen 9713, AV, The Netherlands

## Abstract

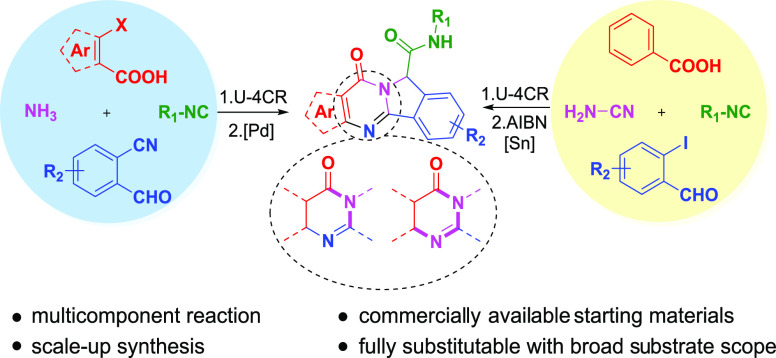

The rapid synthesis of diverse substituted polycyclic quinazolinones was achieved by two orthogonal Ugi four-component reaction (Ugi-4CR)-based protocols: the first two-step approach via an ammonia-Ugi-4CR followed by palladium-catalyzed annulation; in the second approach, cyanamide was used unprecedently as an amine component in Ugi-4CR followed by an AIBN/tributyltin hydride-induced radical reaction. Like no other method, MCR and cyclization could efficiently construct many biologically interesting compounds with tailored properties in very few steps.

## Introduction

Quinazolinones are among the very important class of biologically active N-fused heterocyclic scaffolds that have been involved in many marketed drugs or potential candidates.^1^ Notably, polycyclic quinazolinones, such as luotonins (**1**–**3**), sclerotigenin (**4**), and rutaecarpine (**5**), have been reported to exhibit a broad spectrum of biological activities.^2^ Thus, the large structural diversity and promising therapeutic potential of polycyclic quinazolinone analogs have inspired chemists to further explore novel variants of quinazolinone structures or develop more efficient synthetic methodologies.

Multicomponent reactions (MCRs) are reactions that employ at least three starting materials to form one single product, where the majority of the atoms from starting materials are incorporated into final products.^3^ Due to their high efficiency, mild conditions, and large scaffolds diversity, the MCRs have become a unique tool for the rapid generation of a great variety of natural products and pharmaceuticals.^4^ For example, β-amino amides have been successfully synthesized in a one-pot manner by applying triazenyl alkynes, carboxylic acids, aldehydes, and anilines as the starting materials.^5^ The Ugi reaction is one of the most well-known and broadly used MCRs.^6^ It has been involved in the synthesis of diverse scaffolds as a key step. For instance, the synthesis of a potent amino acid antibiotic furanomycin and a naturally cyclic peptide ustiloxin D used the Ugi reaction as the critical step.^7^ In our previous studies, we have introduced Ugi reactions for constructing important bioactive scaffolds, such as isoquinolone-4-carboxylic acid and isoquinoline scaffolds.^8^

**Scheme 1:**
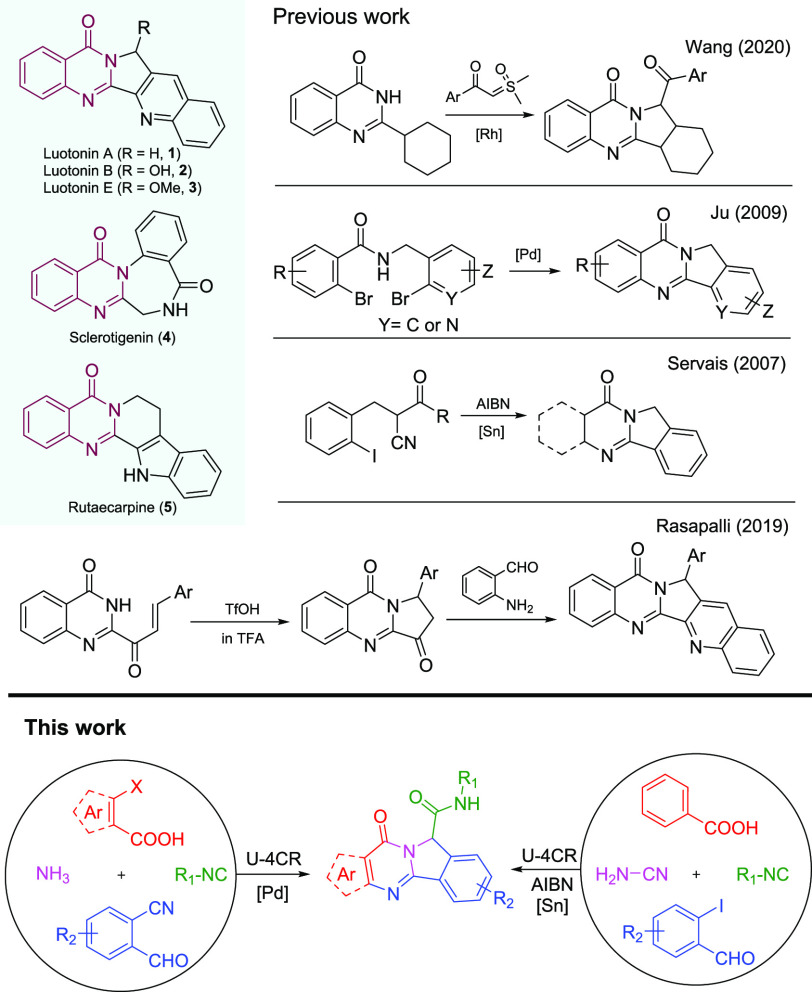
Representatives and Synthesis of Polycyclic Quinazolinones

The rapid construction of polycyclic quinazolinones is an extensively studied topic. Several metal catalysts, such as copper, rhodium, and palladium, have been used for the synthesis of the corresponding compounds.^9^ The application of radical cyclization as well as self-catalyzed phototandem perfluoroalkylation/cyclization of unactivated alkenes also has been reported for the synthesis of the same scaffolds.^10^ As shown in Scheme 1, these approaches suffer from one or many issues, such as lengthy sequential synthesis, limited scope, and generality.^9−11^ Inspired by previous syntheses and based on our deep interest in MCR chemistry, we envisioned that isoindolo[1,2-*b*]quinazolinone derivatives could be synthesized in a concise manner by an Ugi-4CR reaction of *o*-bromobenzoic acids, *o*-cyanobenzaldehydes, isocyanides, and ammonia followed by a metal-catalyzed intramolecular N-arylation to form the desired products.^12^ Alternatively, we figured, in a second strategy, that it could also be synthesized by an Ugi-4CR using *o*-iodobenzaldehyde, benzoic acid, isocyanides, and cyanamide followed by radical cyclization of the N-acylcyanamide moiety since the cyano group is a well-established radical acceptor that has been involved in the build of various heterocycles and carbocycles.^13^ We were the first to report cyanamide as an acid component in the Ugi reaction, reacting with enamines and isocyanides in the presence of Lewis acids to give the medicinally important scaffold α-amino-N-cyanoamidines.^14^ Here, we report for the first time cyanamide to react as an amine component in the Ugi reaction.

## Results and Discussion

As the starting point of our work to elaborate the first strategy, the model Ugi-4CR with 2-bromobenzoic acid (**6a**), 2-cyanobenzaldehyde (**7a**), cyclohexyl isocyanide (**8a**), and NH_4_Cl (**9a**) was performed in MeOH/H_2_O (3:1) under room temperature for 12 h (entry 1) as shown in Table 1.^15^ The precipitated Ugi product **10a** formed, and the solid was filtered and washed with diethyl ether giving a 33% yield. We then used MeOH as the solvent (entry 2) or increased the temperature to 55 °C (entry 3), giving yields of 60% and 33%, respectively. Not satisfied, we tried to use ammonia (7 N in MeOH, **9c**) instead of NH_4_Cl in 2,2,2-trifluoroethanol (TFE) at 55 °C in a closed vial for 12 h (entry 5).^16^ No precipitated Ugi product was observed, but a yield of 62% was isolated after flash chromatography purification. Thereafter, the reactions were carried out under room temperature in TFE (entry 6) or MeOH (entry 7), which both formed the precipitated Ugi product with yields of 75% and 60%, respectively. When ammonia (in water, **9b**) was used (entry 4), a trace amount of Ugi product was formed. Though 2,4-dimethoxylbenzylamine (entry 8) as an ammonia surrogate showed good performance in this reaction, two steps with a total yield of 59% were not competitive. Finally, the optimized reaction condition was concluded to be ammonia (in MeOH, **9c**) in TFE at room temperature for 12 h (entry 6).

**Table 1:**
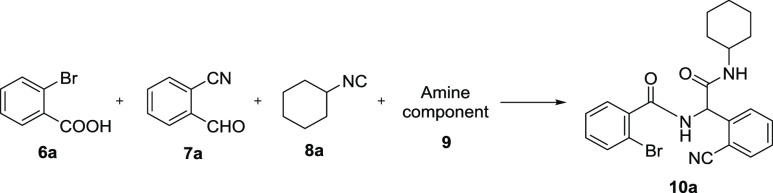
Optimization of Ugi-4CR Conditions

entry^a^	amine component	solvent	*T* (°C)	yield^b^
1	NH_4_Cl (**9a**)	MeOH/H_2_O = 3:1	r.t.	33%
2	NH_4_Cl	MeOH	r.t.	41%
3	NH_4_Cl	MeOH/H_2_O = 3:1	55 °C	33%
4	ammonia in water (**9b**)	TFE	r.t.	trace
5	ammonia in MeOH (**9c**)	TFE	55 °C	62%
6	ammonia in MeOH	TFE	r.t.	75%
7	ammonia in MeOH	MeOH	r.t.	60%
8^c^	2,4-dimethoxybenzylamine (**9d**)	TFE	r.t.	59%

a Reaction conditions: **6a** (1 mmol), **7a** (1 mmol), **8a** (1 mmol), **9** (1 mmol), solvent (1 mL).

b Isolated yields.

c Followed by HCl-catalyzed cleavage.

With optimized conditions for the Ugi-4CR in hand, we next investigated the subsequent palladium-catalyzed annulation step by exploring varied catalysts, ligands, and bases (Table 2). When the reaction was performed in the presence of 5 mol % Pd(OAc)_2_, 10 mol % 1,1′-bis(diphenylphosphino)ferrocene (dppf), and 2 equiv of K_3_PO_4_ in 1,4-dioxane at reflux for overnight, the desired product **10b** was obtained in 65% yield (entry 9). When PdCl_2_ (entry 10) or PdO (entry 11) has been used as the catalyst, the yield decreased to 16% and 20%, respectively. Replacing the dppf with Xantphos resulted in a lower yield (33%, entry 12). To our delight, the desired product **10b** was formed in 81% yield by using K_2_CO_3_ (entry 15) as the base, compared with K_3_PO_4_ (65%, entry 9) and Cs_2_CO_3_ (72%, entry 14). Notably, microwaves did not facilitate the reaction as we expected (42%, entry 13). Furthermore, we tried different organometallic complexes giving no product formation or low yields (entry 16–19). Finally, the optimized condition was concluded to be the Ugi adduct **10a**, Pd(OAc)_2_, dppf, and K_2_CO_3_ in 1,4-dioxane at reflux overnight (entry 15).

**Table 2:**
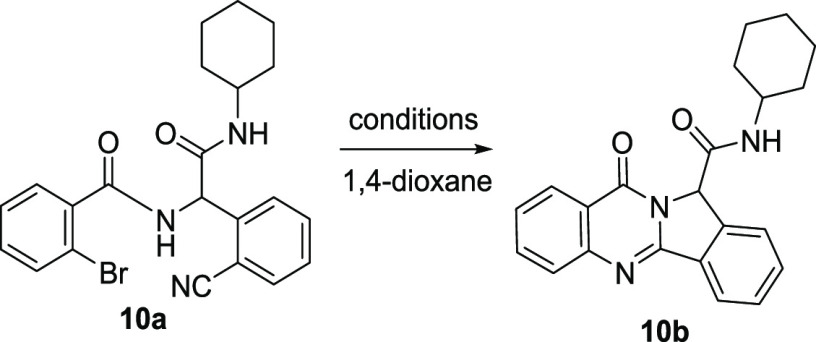
Optimization of [Pd]-Catalyzed Annulation

entry^a^	catalyst	ligand	base	yield^c^
9	Pd(OAc)_2_	dppf	K_3_PO_4_	65%
10	PdCl_2_	dppf	K_3_PO_4_	16%
11	PdO	dppf	K_3_PO_4_	20%
12	Pd(OAc)_2_	Xantphos	K_3_PO_4_	33%
13^b^	Pd(OAc)_2_	dppf	K_3_PO_4_	42%
14	Pd(OAc)_2_	dppf	Cs_2_CO_3_	72%
15	Pd(OAc)_2_	dppf	K_2_CO_3_	81%
16	PdCl_2_(PPh_3_)_2_		K_3_PO_4_	n.d^d^
17	Pd(dppf)Cl_2_		K_3_PO_4_	34%
18	[(C_6_H_5_)_3_P]_2_Pd(CH_2_C_6_H_5_)Cl		K_3_PO_4_	trace
19	PdCl_2_·(CH_3_CN)_2_		K_3_PO_4_	trace

a Reaction conditions: **10a** (0.5 mmol), catalyst (5 mol %), ligand (10 mol %), base (1 mmol), 1,4 dioxane (5 mL), reflux, overnight.

b Microwave, 120 °C, 30 min.

c Isolated yields.

d Not detected.

With the optimal conditions in hand, we then set out to explore the scope and limitation of these tandem reactions. A set of Ugi-4CR adducts **10a**–**33a** were efficiently synthesized in moderate to good yields without column purification required for most cases. Aromatic/aliphatic isocyanides, versatile *o*-bromobenzoic acids, and substituted o-cyanobenzaldehydes were all tolerated well in this ammonia Ugi four-component reaction. Then, all the Ugi adducts were examined to determine the scope of the tandem reaction to furnish the corresponding products **10b**–**33b**.

**Scheme 2:**
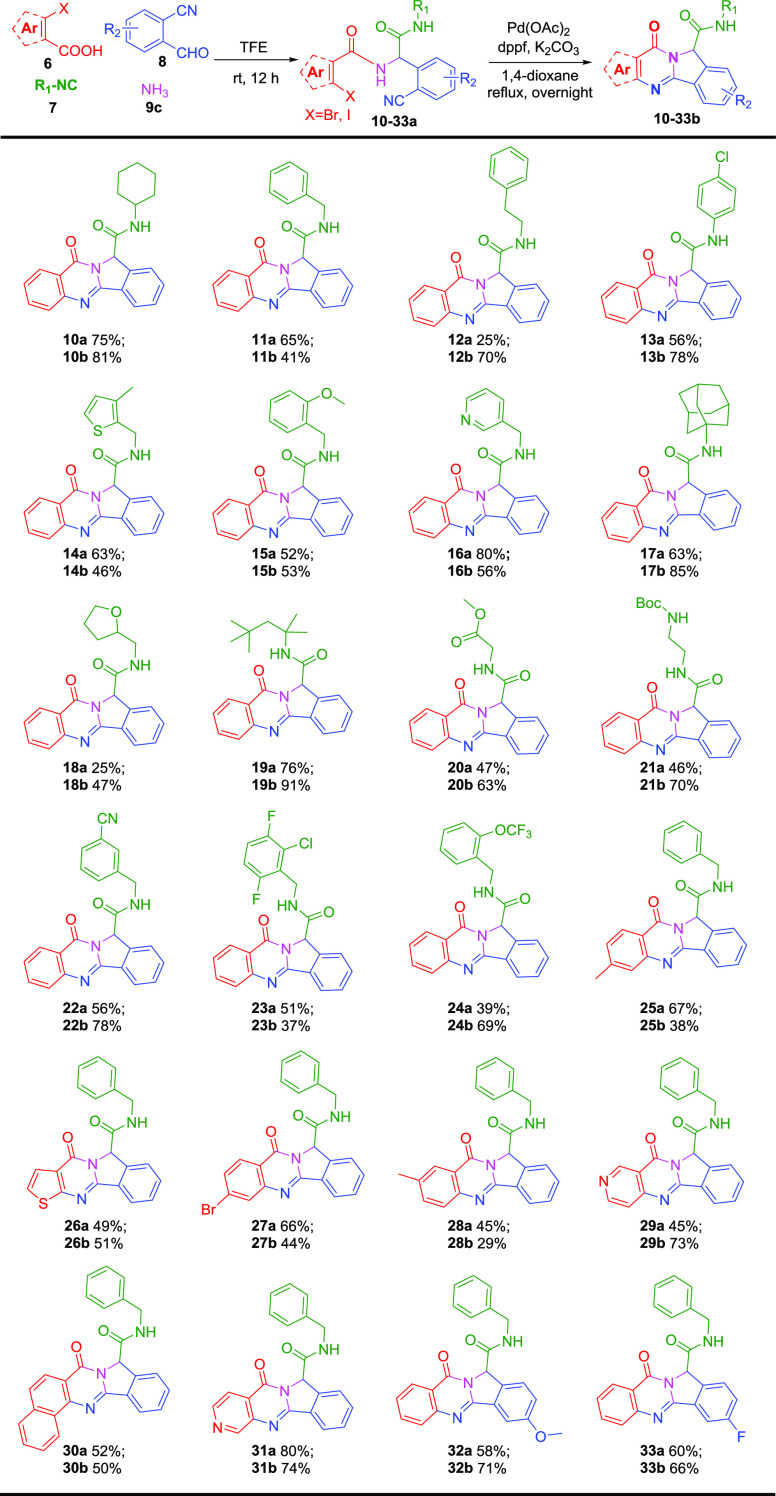
Substrate Scope in the [Pd]-Catalyzed Synthesis of Polycyclic Quinazolinones^–^ Acid, isocyanide, aldehyde, and ammonia components are depicted with red, green, blue, and pink color, respectively. Ugi reaction was carried out using **6** (1 mmol), **7** (1 mmol), **8** (1 mmol), and **9c** (1 mmol) in TFE (1 M) for 12 h at r.t. [Pd]-catalyzed reaction conditions: **10**–**34a** (0.2 mmol), Pd(OAc)_2_ (5 mol %), dppf (10 mol %), K_2_CO_3_ (1 mmol), 1,4 dioxane (2 mL), reflux, overnight. Yield refers to purified products.

As can be seen in Scheme 2, all the Ugi adducts led to the expected polycyclic quinazolinones. We initially replaced cyclohexyl isocyanide with various aromatic isocyanides, such as benzyl (**11b**, 41%), phenethyl (**12b**, 70%), phenyl (**13b**, 78%), thienylmethyl (**14b**, 46%), and pyridylmethyl (**16b**, 56%), all giving good yields. Benzyl isocyanides bearing diverse functional groups also performed well in this reaction, as examples, methoxy (**15b**, 53%), nitrile (**22b**, 78%), halide (**23b**, 37%), and trifluoromethoxy (**24b**, 69%). Further, aliphatic isocyanides 1-adamantyl isocyanide (**17b**, 85%), tetrahydrofuran-2-ylmethyl isocyanide (**18b**, 47%), *tert*-octyl isocyanide (**19b**, 91%), methyl isocyanoacetate (**20b**, 63%), and *tert*-butyl (2-isocyanoethyl)carbamate (**21b**, 70%) gave good yields as well. This is meaningful since the previously reported methods either have the limited type of substitutions or are non-substituted in this position.^9−11^ Meanwhile, by using our strategy, we opened the substitution possibilities at this position for all available isocyanides providing the opportunity for various further chemical modifications.

Subsequently, we examined the ability of these tandem reactions to incorporate a diverse panel of carboxylic acids and aldehydes components. All the used acid and aldehyde components were well-tolerated in Ugi-4CR, giving good yields ranging from 45% to 80%. For the subsequent [Pd]-catalyzed annulation, thiophenecarboxylic acid (**26b**), nicotinic acids (**29b**, **31b**), and naphthoic acid (**30b**) proceeded smoothly. Nicotinic acids significantly increased yields when compared with benzoic acid (**11b**), which may probably benefit from the electron-withdrawing effect of the nitrogen atom. Additionally, different functional groups at the aromatic ring of *o*-bromobenzoic acid were well tolerated, such as methyl (**25b**, **27b**) and bromine (**27b**). The relatively lower yields have been observed when the methyl group attached may be due to its electron-donating effect, making the acid less reactive. In addition, good substrate tolerance was also achieved for the aldehyde component. 2-Cyano-4-methoxybenzaldehyde (**32b**) and 4-fluoro-3-cyanobenzaldehyde (**33b**) yielded 71% and 66% of products. A plausible mechanism of this reaction is that the cyano group is first activated by a transition metal to form a σ-coordination, which will facilitate further nucleophilic addition. Then, the following intramolecular cyclization might be executed by the metal-catalyzed S_N_Ar reaction.

**Scheme 3:**
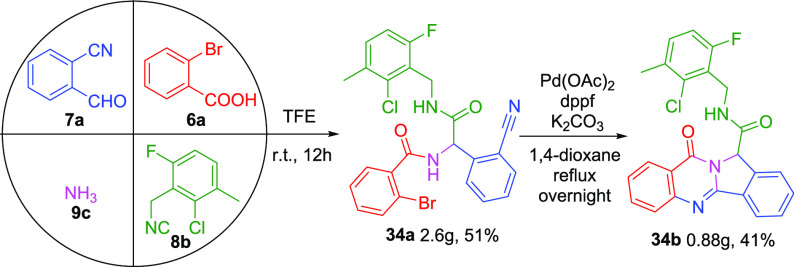
Scale-up Synthesis

**Scheme 4:**
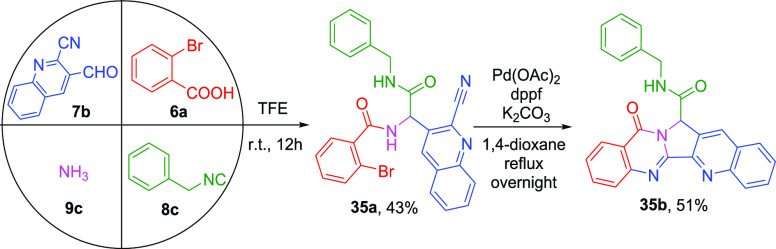
Synthesis of luotonin Derivatives

Furthermore, the scalability of this method was investigated. A four-component reaction of 2-bromobenzoic acid (**6a**), 2-cyanobenzaldehyde (**7a**), 2-chloro-3-methyl-6-fluorobenzyl isocyanide (**8b**), and ammonia (**9c**) was conducted on a 10 mmol scale. The participate was filtered and washed with diethyl ether, affording 2.6 g of Ugi adduct **34a** (51%). Then, Ugi adduct **34a** yielded 0.88 g of the final cyclized product **34b** (41%) through silica gel flash chromatography as shown in Scheme 3.

luotonins A, B, and E (**1**–**3**) are naturally occurring cytotoxic alkaloids, being demonstrated as human DNA topoisomerase I (hTopI) poisons.^17^ The current synthesis of luotonin derivatives may suffered from multiple synthetic procedures and/or a limited substrate scope.^9−11,18^Scheme 4 illustrates our initial success of luotonin A derivative **35b** (51%) synthesis of which the Ugi adduct **35a** (43%) was assembled from the appropriate 2-bromobenzoic acid (**6a**), 2-quinolinecarbonitrile (**7b**), ammonia (**9c**), and benzyl isocyanide (**8c**). Despite this, the Passerini three-component (P-3CR) byproduct was observed in Ugi-4CR. Our strategy could incorporate a variety of functionalities from largely available isocyanides into the luotonin scaffold.

**Scheme 5:**
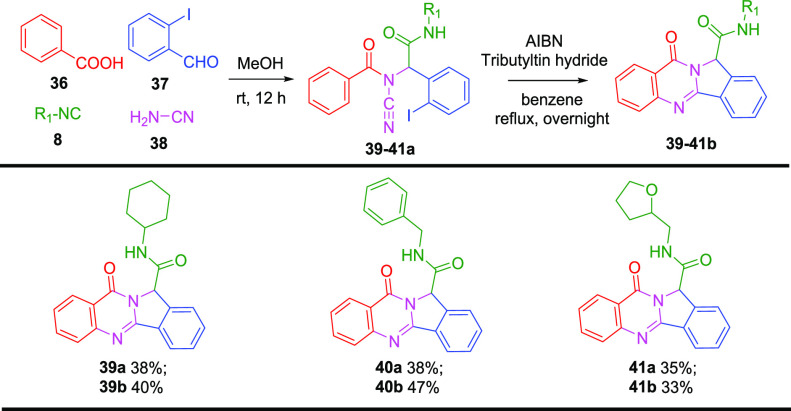
Various Isocyanides in the Synthesis of Polycyclic Quinazolinones via Radical Cyclization^–^ Acid, aldehyde, isocyanide, and cyanamide components are depicted with red, blue, green, and pink color, respectively. Ugi reaction was carried out using **36** (1 mmol), **37** (1 mmol), **8** (1 mmol), and **38** (1 mmol) in TFE (1 M) for 12 h at r.t. Radical cyclization conditions: **39**–**41a** (0.15 mmol), tributyltin hydride (0.3 mmol), AIBN (0.15 mmol), benzene (7 mL), reflux, overnight. Yield refers to purified products.

Encouraged by our successful development, we continued to explore the second approach via a cyanamide-Ugi-4CR followed by an AIBN/tributyltin hydride-induced radical cyclization. The reaction started with benzoic acid (**36**), 2-iodobenzaldehyde (**37**), cyanamide (**38**), and three different isocyanides (Scheme 5). The reactions afforded the corresponding Ugi products **39a**–**41a** with moderate yields because P-3CR byproducts were isolated with a nearly 1:1 ratio from each reaction. The P-3CR product is often observed when less nucleophilic amines are employed in the Ugi-4CR, which can be explained by a low formation of the iminium intermediates in the reaction mixture.^19^ To optimize the reaction, we tried several reaction conditions: (i) using 2 equiv of cyanamide; (ii) heating to 60 °C; (iii) using microwaves (100 °C, 30 min); (iv) changing the solvent; (v) adding Lewis acids as the catalyst, such as ZnCl_2_, TiCl_4_, HClO_4_, scandium(III) triflate, ytterbium(III) triflate.^20^ Unfortunately, they failed to improve the yields.

Despite achieving moderate yields, we were able to isolate sufficient quantities of the Ugi products to examine the subsequent step. The reactions were performed between Ugi adducts, tributyltin hydride, and AIBN in benzene at reflux overnight. To our delight, all the three Ugi adducts yielded the corresponding products **39**–**41b**, giving yields of 40%, 47%, and 33%, respectively. Therefore, we have successfully introduced cyanamide into the Ugi-4CR as the amino component that generated the stable *N*-acylcyanamide moiety, which could undergo further cyclization leading to the desired polycyclic quinazolinones. A plausible mechanism for this radical cascade is that the trapped aromatic radical on the cyanamide triple bond could further cyclize on an aryl substituent. This cyclization undergoes direct addition to form the hexadienyl radical, which further goes with rearomatization.^21^

## Conclusions

Two orthogonal Ugi-4CR-based synthesis strategies for fully substituted polycyclic quinazolinone derivatives have been developed. Alternative Ugi-4CRs were instrumental in this approach for introducing the critical cyano group, leading to potentially bioactive polycyclic quinazolinones through two different cyclization mechanisms. For this, we introduced cyanamide for the first time as an amine component in the Ugi-4CR. Remarkably, cyanamide is thus the first amphoteric building block that can function as an acid and amine component in Ugi-type transformations. Significantly, the successfully synthesized *N*-acylcyanamide moiety, as an efficient amide-iminyl radical, opens the door for the rapid and straightforward synthesis of pharmaceutically important heterocycles by Ugi-4CR. Diverse isocyanides with versatile functional groups can be introduced into the polycyclic quinazolinone scaffold allowing the modification of physicochemical properties, further chemical manipulations, and so on. Our protocol is outperforming all known strategies toward this important scaffold in terms of utilization and accessibility of commercially available starting materials, mild conditions, and simple purification work-up and is of excellent maneuverability and efficiency. Work aiming at the optimization of cyanamide involved Ugi-4CR as well as extending the scope of the subsequent annulation is in progress and will be reported in due course.

## Experimental Section

### General Information

Nuclear magnetic resonance spectra were recorded on a Bruker Avance 500 spectrometer. Chemical shifts for ^1^H NMR were reported relative to TMS (δ 0 ppm) or the internal solvent peak (CDCl_3_ δ 7.26 ppm, DMSO-*d*_6_ δ 2.50 ppm or CD_3_OD δ 3.31 ppm) and coupling constants were in hertz (Hz). The following abbreviations were used for spin multiplicity: s = singlet, d = doublet, t = triplet, dt = double triplet, ddd = doublet of double doublet, m = multiplet, and br = broad. Chemical shifts for ^13^C NMR reported in ppm relative to the solvent peak (CDCl_3_ δ 77.23 ppm, DMSO δ 39.52 ppm, CD_3_OD δ 49.00 ppm). Flash chromatography was performed on a Grace Reveleris X2 using Grace Reveleris Silica columns (12 g) and a gradient of petroleum ether/ethyl acetate (0–100%) or dichloromethane/methanol (0–10%) was applied. Thin-layer chromatography was performed on Fluka precoated silica gel plates (0.20 mm thick, particle size 25 μm). Reagents were available from commercial suppliers and used without any purification unless otherwise noted. All isocyanides were made in house by performing the Ugi procedure. Other reagents were purchased from Sigma Aldrich, ABCR, Acros, Fluorochem, and AK Scientific and were used without further purification. High-resolution mass spectra (HRMS) were recorded using a QTOF Bruker Maxis Plus, mass range 100–1500 *m*/*z*, spectra rate 2.00 Hz. Yields given refer to chromatographically purified and spectroscopically pure compounds unless otherwise stated.

### General Experimental Procedure of Ammonia-Ugi-4CR

To a stirred solution of aldehyde (1 mmol, 1 equiv) in 2,2,2-trifluoroethanol (1 mL) was added ammonia solution (7 N in methanol, 1 mmol, 1 equiv) and stirred for 10 min followed by the addition of isocyanide (1 mmol, 1 equiv) and carboxylic acid (1 mmol, 1 equiv). The reaction was allowed to stir at room temperature in a close screwed vial for 12 h. The precipitate was filtered and washed with diethyl ether, affording the corresponding Ugi adduct. Otherwise, the Ugi adduct was purified by silica gel flash chromatography using either PE/EA or DCM/MeOH as the eluent.

### General Experimental Procedure of [Pd]-Catalyzed Annulation

The Ugi adduct (0.15–0.4 mmol, 1 equiv), Pd(OAc)_2_ (0.0075–0.02 mmol, 0.05 equiv), dppf (0.015–0.04 mmol, 0.1 equiv), and K_2_CO_3_ (0.3–0.8 mmol, 2 equiv) were added to a 5 mL (or 10 mL) reaction tube equipped with a magnetic stir bar, and 1.5–4 mL of 1,4-dioxane was added. The mixture was heated to reflux in a metal heating block overnight. After the reaction was completed, the solvent was removed under reduced pressure, and the residue was purified by silica gel flash chromatography using either PE/EA or DCM/MeOH as the eluent.

#### Scale-up Synthesis of **34b**

A 50 mL flask equipped with a magnetic stirrer bar was charged with 2-formylbenzonitrile (10 mmol, 1.3 g, 1 equiv) and a calculated amount of 7 N ammonia solution (in methanol, 10 mmol, 1.4 mL, 1 equiv) in 2,2,2-trifluoroethanol (10 mL). We sealed the reaction flask immediately and allowed it to stir for 10 min. Then, 2-chloro-4-fluoro-3-(isocyanomethyl)-1-methylbenzene (10 mmol, 1.8 g, 1 equiv) and 2-bromobenzoic acid (10 mmol, 2.0 g, 1 equiv) were added to the solution and the reaction mixture was stirred at room temperature for 12 h. The precipitate was filtrated and washed by diethyl ether, affording the pure Ugi adduct **34a** with a yield of 51%. Then, the Ugi adduct (5 mmol, 2.6 g, 1 equiv) was added to Pd(OAc)_2_ (0.25 mmol, 56 mg, 0.05 equiv), dppf (0.5 mmol, 277 mg, 0.1 equiv), and K_2_CO_3_ (10 mmol, 1.4 g, 2 equiv) in 1,4-dioxane (50 mL) in a 200 mL round flask with a magnetic stirrer bar. The reaction mixture was heated to reflux in an oil bath overnight. After the reaction was completed, the solvent was removed under reduced pressure, and the crude product was purified by column chromatography (silica gel, petroleum ether/ethyl acetate = 2:1) to afford the product **34b** (0.88 g, 41% yield).

#### Synthesis of Luotonin Derivative **35b**

To a stirred solution of 2-quinolinecarbonitrile (1 mmol, 154 mg, 1 equiv) in 2,2,2-trifluoroethanol (1 mL) was added ammonia solution (7 N in methanol, 1 mmol, 140 μL, 1 equiv) and stirred for 10 min followed by the addition of benzyl isocyanide (1 mmol, 117 mg, 1 equiv) and 2-bromobenzoic acid (1 mmol, 201 mg, 1 equiv). The reaction was allowed to stir at room temperature in a close screwed vial for 12 h. The reaction was concentrated and purified by column chromatography (silica gel, petroleum ether/ethyl acetate as eluent) to afford the corresponding Ugi product **35a**. Subsequently, the Ugi adduct (0.15 mmol, 75 mg, 1 equiv), Pd(OAc)_2_ (0.0075 mmol, 1.7 mg, 0.05 equiv), dppf (0.015 mmol, 8.3 mg, 0.1 equiv), and K_2_CO_3_ (0.3 mmol, 41.5 mg, 2 equiv) were added to a 5 mL reaction tube equipped with a magnetic stir bar, and 1.5 mL of 1,4-dioxane was added. The mixture was heated to reflux in a metal heating block overnight. After the reaction was completed, the solvent was removed under reduced pressure, and the residue was purified by silica gel flash chromatography using PE/EA as the eluent.

### General Experimental Procedure of Cyanamide-Ugi-4CR

To a stirred solution of aldehyde (1 mmol, 1 equiv) in MeOH (1 mL) was added cyanamide (1 mmol, 1 equiv) and stirred for 30 min followed by the addition of isocyanide (1 mmol, 1 equiv) and carboxylic acid (1 mmol, 1 equiv). The reaction was allowed to stir at room temperature for 12 h. The reaction was concentrated and purified by column chromatography (silica gel, petroleum ether/ethyl acetate = 2:1) to afford the corresponding Ugi product.

### General Experimental Procedure of Radical Cyclization

The Ugi adduct (0.15 mmol, 1 equiv), tributyltin hydride (0.3 mmol, 2 equiv), and azobisisobutyronitrile (AIBN, 0.15 mmol, 1 equiv) were added to a 25 mL round flask equipped with a magnetic stir bar, and 7 mL of benzene was added. The mixture was heated to reflux overnight. After the reaction was completed, NaOH (aq, 1 M, 10 mL) was added to the reaction mixture and stirred for 30 min. The organic phase was extracted with ethyl acetate (2 × 20 mL), dried over MgSO_4_, and concentrated under vacuum. The residue was purified by silica gel flash chromatography using PE/EA as the eluent.

#### 2-Bromo-*N*-(1-(2-cyanophenyl)-2-(cyclohexylamino)-2-oxoethyl)benzamide (**10a**)

Obtained from a 1 mmol reaction as a white solid, 330 mg, yield 75%; ^1^H NMR (500 MHz, DMSO-*d*_6_) δ 9.30 (d, *J* = 7.7 Hz, 1H), 8.18 (d, *J* = 7.8 Hz, 1H), 7.87 (d, *J* = 8.8 Hz, 1H), 7.72 (td, *J* = 7.7, 1.5 Hz, 1H), 7.65 (d, *J* = 8.0 Hz, 1H), 7.60 (d, *J* = 7.8 Hz, 1H), 7.52 (td, *J* = 7.5, 1.2 Hz, 1H), 7.49–7.42 (m, 2H), 7.37 (td, *J* = 7.8, 2.0 Hz, 1H), 5.82 (d, *J* = 7.7 Hz, 1H), 3.67–3.53 (m, 1H), 1.82–1.62 (m, 4H), 1.59–1.51 (m, 1H), 1.31–1.22 (m, 3H), 1.20–1.06 (m, 2H). ^13^C{^1^H} NMR (126 MHz, DMSO-*d*_6_) δ 167.3, 167.3, 141.9, 138.5, 133.6, 133.5, 133.2, 133.0, 131.5, 129.7, 128.9, 127.8, 119.5, 117.8, 112.7, 55.9, 48.5, 32.6, 25.6, 24.8. HRMS (ESI) *m*/*z* calculated for C_22_H_23_BrN_3_O_2_ [M + H]^+^: 440.0974, found [M + H]^+^: 440.0967.

#### *N*-(2-(benzylamino)-1-(2-cyanophenyl)-2-oxoethyl)-2-bromobenzamide (**11a**)

Obtained from a 1 mmol reaction as a white solid, 290 mg, yield 65%; ^1^H NMR (500 MHz, chloroform-*d*) δ 7.74–7.69 (m, 3H), 7.64 (td, *J* = 7.6, 1.3 Hz, 1H), 7.60 (dt, *J* = 7.6, 1.5 Hz, 2H), 7.45 (td, *J* = 7.6, 1.1 Hz, 1H), 7.36 (td, *J* = 7.5, 1.2 Hz, 1H), 7.32–7.26 (m, 4H), 7.18–7.14 (m, 2H), 6.47 (t, *J* = 5.6 Hz, 1H), 5.96 (d, *J* = 6.1 Hz, 1H), 4.54–4.39 (m, 2H). ^13^C{^1^H} NMR (126 MHz, CDCl_3_) δ 167.8, 166.5, 141.4, 136.9, 136.2, 133.6, 133.5, 133.2, 131.8, 130.1, 128.8, 128.8, 128.0, 127.7, 127.6, 127.5, 119.5, 117.9, 111.6, 56.5, 44.1. HRMS (ESI) *m*/*z* calculated for C_23_H_19_BrN_3_O_2_ [M + H]^+^: 448.0661, found [M + H]^+^: 448.0654.

#### 2-Bromo-*N*-(1-(2-cyanophenyl)-2-oxo-2-(phenethylamino) ethyl)benzamide (**12a**)

Obtained from a 1 mmol reaction as a light yellow solid, 95 mg, yield 25%; eluent: *V*_PE_/*V*_EA_ = 2:1; ^1^H NMR (500 MHz, chloroform-*d*) ^1^H NMR (500 MHz, chloroform-*d*) δ 7.77 (d, *J* = 6.0 Hz, 1H), 7.71–7.59 (m, 5H), 7.46 (td, *J* = 7.4, 1.6 Hz, 1H), 7.38 (td, *J* = 7.5, 1.2 Hz, 1H), 7.32 (td, *J* = 7.6, 1.8 Hz, 1H), 7.26–7.17 (m, 3H), 7.06–7.01 (m, 2H), 6.16 (t, *J* = 5.9 Hz, 1H), 5.86 (d, *J* = 5.9 Hz, 1H), 3.70–3.60 (m, 1H), 3.53–3.43 (m, 1H), 2.83–2.73 (m, 2H). ^13^C{^1^H} NMR (126 MHz, CDCl_3_) δ 167.7, 166.4, 141.6, 138.0, 136.2, 133.5, 133.5, 133.2, 131.7, 130.0, 128.7, 128.6, 128.6, 127.9, 127.6, 126.6, 119.5, 117.8, 111.5, 56.3, 41.2, 35.2. HRMS (ESI) *m*/*z* calculated for C_24_H_21_BrN_3_O_2_ [M + H]^+^: 462.0817, found [M + H]^+^: 462.0812.

#### 2-Bromo-*N*-(2-((4-chlorophenyl)amino)-1-(2-cyanophenyl)-2-oxoethyl)benzamide (**13a**)

Obtained from a 1 mmol reaction as a yellow solid, 260 mg, yield 56%; ^1^H NMR (500 MHz, DMSO-*d*_6_) δ 10.63 (s, 1H), 9.49 (d, *J* = 7.1 Hz, 1H), 7.93 (dd, *J* = 7.7, 1.4 Hz, 1H), 7.74 (td, *J* = 7.7, 1.4 Hz, 1H), 7.71–7.64 (m, 3H), 7.62–7.51 (m, 3H), 7.47 (td, *J* = 7.5, 1.2 Hz, 1H), 7.43–7.36 (m, 3H), 6.00 (d, *J* = 7.1 Hz, 1H). ^13^C{^1^H} NMR (126 MHz, DMSO-*d*_6_) δ 167.5, 167.4, 140.7, 138.4, 138.1, 133.8, 133.7, 133.2, 133.1, 131.6, 129.8, 129.2, 129.2, 127.7, 121.5, 121.4, 119.5, 117.7, 112.8, 56.7. HRMS (ESI) *m*/*z* calculated for C_22_H_16_BrClN_3_O_2_ [M + H]^+^: 468.0114, found [M + H]^+^: 468.0109.

#### 2-Bromo-*N*-(1-(2-cyanophenyl)-2-(((3-methylthiophen-2-yl) Methyl)amino)-2-oxoethyl)benzamide (**14a**)

Obtained from a 1 mmol reaction as a light yellow solid, 294 mg, yield 63%; ^1^H NMR (500 MHz, chloroform-*d*) δ 7.76–7.57 (m, 6H), 7.47 (td, *J* = 7.6, 1.3 Hz, 1H), 7.42–7.37 (m, 1H), 7.32 (td, *J* = 7.8, 1.8 Hz, 1H), 7.12 (d, *J* = 5.1 Hz, 1H), 6.80 (d, *J* = 5.1 Hz, 1H), 6.37 (s, 1H), 5.95 (d, *J* = 6.1 Hz, 1H), 4.65–4.50 (m, 2H), 2.18 (s, 3H). ^13^C{^1^H} NMR (126 MHz, CDCl_3_) δ 167.4, 166.5, 141.2, 136.2, 135.4, 133.5, 133.2, 132.1, 131.8, 131.0, 130.2, 130.1, 128.9, 128.1, 127.6, 123.6, 119.4, 117.9, 111.6, 56.4, 37.0, 13.5. HRMS (ESI) *m*/*z* calculated for C_22_H_19_BrN_3_O_2_S [M + H]^+^: 468.0381, found [M + H]^+^: 468.0375.

#### 2-Bromo-*N*-(1-(2-cyanophenyl)-2-((2-methoxybenzyl)amino)-2-oxoethyl)benzamide (**15a**)

Obtained from a 1 mmol reaction as a brown solid, 248 mg, yield 52%; ^1^H NMR (500 MHz, chloroform-*d*) δ 7.84–7.77 (m, 1H), 7.74–7.68 (m, 2H), 7.65–7.58 (m, 3H), 7.44 (td, *J* = 7.6, 1.1 Hz, 1H), 7.38 (td, *J* = 7.5, 1.3 Hz, 1H), 7.34–7.29 (m, 2H), 7.18 (dd, *J* = 7.3, 1.7 Hz, 1H), 6.92–6.86 (m, 2H), 6.80 (t, *J* = 5.9 Hz, 1H), 5.91 (d, *J* = 5.8 Hz, 1H), 4.55–4.37 (m, 2H), 3.85 (s, 3H). ^13^C{^1^H} NMR (126 MHz, CDCl_3_) δ 167.3, 166.3, 157.5, 141.9, 136.3, 133.5, 133.4, 133.0, 131.7, 130.0, 129.6, 129.2, 128.6, 127.6, 127.5, 124.9, 120.5, 119.5, 117.8, 111.6, 110.2, 56.4, 55.2, 40.7. HRMS (ESI) *m*/*z* calculated for C_24_H_21_BrN_3_O_3_ [M + H]^+^: 478.0766, found [M + H]^+^: 478.0760.

#### 2-Bromo-*N*-(1-(2-cyanophenyl)-2-oxo-2-((pyridin-3-ylmethyl)amino)ethyl) Benzamide (**16a**)

Obtained from a 1 mmol reaction as a yellow solid, 359 mg, yield 80%; ^1^H NMR (500 MHz, chloroform-*d*) δ 8.50 (dd, *J* = 4.9, 1.6 Hz, 1H), 8.45 (d, *J* = 2.3 Hz, 1H), 7.77 (d, *J* = 6.3 Hz, 1H), 7.74–7.69 (m, 2H), 7.65 (td, *J* = 7.7, 1.4 Hz, 1H), 7.63–7.56 (m, 3H), 7.47 (td, *J* = 7.6, 1.1 Hz, 1H), 7.37 (td, *J* = 7.5, 1.2 Hz, 1H), 7.34–7.29 (m, 1H), 7.27–7.23 (m, 1H), 7.08 (t, *J* = 6.1 Hz, 1H), 6.04 (d, *J* = 6.4 Hz, 1H), 4.54–4.43 (m, 2H). ^13^C{^1^H} NMR (126 MHz, CDCl_3_) δ 168.2, 166.7, 148.6, 141.0, 136.1, 135.7, 133.6, 133.4, 131.8, 131.2, 130.6, 130.0, 129.0, 128.2, 127.6, 127.1, 123.7, 119.4, 117.9, 111.6, 56.4, 41.5. HRMS (ESI) *m*/*z* calculated for C_22_H_18_BrN_4_O_2_ [M + H]^+^: 449.0613, found [M + H]^+^: 449.0606.

#### *N*-(2-(((3*S*,5*S*,7*S*)-Adamantan-1-yl)amino)-1-(2-cyanophenyl)-2-oxoethyl)-2-bromobenzamide (**17a**)

Obtained from a 1 mmol reaction as a white solid, 310 mg, yield 63%; ^1^H NMR (500 MHz, chloroform-*d*) δ 7.74–7.67 (m, 3H), 7.66–7.59 (m, 3H), 7.43 (td, *J* = 7.6, 1.3 Hz, 1H), 7.37 (td, *J* = 7.6, 1.2 Hz, 1H), 7.30 (td, *J* = 7.7, 1.8 Hz, 1H), 5.82 (s, 1H), 5.79 (d, *J* = 6.0 Hz, 1H), 2.05 (t, *J* = 3.2 Hz, 3H), 2.00–1.90 (m, 6H), 1.69–1.61 (m, 6H). ^13^C{^1^H} NMR (126 MHz, CDCl_3_) δ 166.4, 166.2, 142.2, 136.2, 133.6, 133.5, 133.1, 131.7, 130.2, 128.5, 127.6, 127.6, 119.5, 112.3, 111.2, 56.9, 53.1, 41.3, 36.1, 29.3. HRMS (ESI) *m*/*z* calculated for C_26_H_27_BrN_3_O_2_ [M + H]^+^: 492.1287, found [M + H]^+^: 492.1283.

#### 2-Bromo-*N*-(1-(2-cyanophenyl)-2-oxo-2-(((tetrahydrofuran-2-yl)methyl)amino)ethyl) Benzamide (**18a**)

Obtained from a 1 mmol reaction as a white solid, 110 mg, yield 25%; eluent: *V*_DCM_/*V*_MeOH_ = 20:1; ^1^H NMR (500 MHz, chloroform-*d*) δ 7.75 (t, *J* = 6.6 Hz, 1H), 7.71–7.66 (m, 2H), 7.62 (dt, *J* = 7.7, 1.8 Hz, 1H), 7.60–7.56 (m, 2H), 7.43 (td, *J* = 7.6, 1.2 Hz, 1H), 7.34 (td, *J* = 7.5, 1.2 Hz, 1H), 7.30–7.25 (m, 1H), 6.46–6.37 (m, 1H), 5.90 (s, 1H), 3.97–3.86 (m, 1H), 3.85–3.62 (m, 2H), 3.54–3.42 (m, 1H), 3.35–3.23 (m, 1H), 1.96–1.82 (m, 2H), 1.81–1.62 (m, 1H), 1.57–1.29 (m, 1H). ^13^C{^1^H} NMR (126 MHz, CDCl_3_) δ 167.9, 166.5, 141.6, 136.3, 133.5, 133.3, 131.7, 130.0, 128.8, 128.3, 128.2, 127.5, 119.5, 117.7, 111.6, 76.9, 68.2, 56.5, 43.4, 28.4, 25.8. HRMS (ESI) *m*/*z* calculated for C_21_H_21_BrN_3_O_3_ [M + H]^+^: 442.0766, found [M + H]^+^: 442.0759.

#### 2-Bromo-*N*-(1-(2-cyanophenyl)-2-oxo-2-((2,4,4-trimethylpentan-2-yl)amino)ethyl) Benzamide (**19a**)

Obtained from a 1 mmol reaction as a light yellow solid, 356 mg, yield 76%; ^1^H NMR (500 MHz, DMSO-*d*_6_) δ 9.18 (d, *J* = 7.8 Hz, 1H), 7.84 (dd, *J* = 7.7, 1.4 Hz, 1H), 7.71 (td, *J* = 7.7, 1.4 Hz, 1H), 7.68–7.61 (m, 3H), 7.50 (td, *J* = 7.6, 1.2 Hz, 1H), 7.45–7.42 (m, 2H), 7.39–7.35 (m, 1H), 5.81 (d, *J* = 7.9 Hz, 1H), 1.79 (d, *J* = 14.6 Hz, 1H), 1.64 (d, *J* = 14.6 Hz, 1H), 1.32 (d, *J* = 7.8 Hz, 6H), 0.92 (s, 9H). ^13^C{^1^H} NMR (126 MHz, DMSO-*d*_6_) δ 167.2, 167.1, 142.1, 138.6, 133.7, 133.3, 133.2, 133.0, 131.5, 129.7, 128.8, 127.9, 119.4, 118.0, 112.5, 56.3, 55.1, 50.6, 31.7, 29.5, 29.4. HRMS (ESI) *m*/*z* calculated for C_24_H_29_BrN_3_O_2_ [M + H]^+^: 470.1443, found [M + H]^+^: 470.1429.

#### Methyl (2-(2-bromobenzamido)-2-(2-cyanophenyl)acetyl)glycinate (**20a**)

Obtained from a 1 mmol reaction as a white solid, 200 mg, yield 47%; eluent: *V*_PE_/*V*_EA_ = 2:1; ^1^H NMR (500 MHz, chloroform-*d*) δ 7.77–7.70 (m, 3H), 7.67 (td, *J* = 7.7, 1.4 Hz, 1H), 7.65–7.61 (m, 2H), 7.49 (td, *J* = 7.6, 1.3 Hz, 1H), 7.39 (td, *J* = 7.5, 1.2 Hz, 1H), 7.32 (td, *J* = 7.7, 1.8 Hz, 1H), 6.67 (t, *J* = 5.4 Hz, 1H), 6.02 (d, *J* = 6.0 Hz, 1H), 4.15–4.02 (m, 2H), 3.75 (s, 3H). ^13^C{^1^H} NMR (126 MHz, CDCl_3_) δ 169.2, 168.1, 166.5, 141.0, 136.1, 133.6, 133.3, 131.8, 130.1, 129.0, 128.3, 127.6, 119.4, 117.8, 111.7, 56.3, 52.5, 41.6. HRMS (ESI) *m*/*z* calculated for C_19_H_17_BrN_3_O_4_ [M + H]^+^: 430.0402, found [M + H]^+^: 430.0392.

#### *tert*-Butyl (2-(2-(2-bromobenzamido)-2-(2-cyanophenyl)acetamido)ethyl) Carbamate (**21a**)

Obtained from a 1 mmol reaction as a white solid, 230 mg, yield 46%; eluent: *V*_PE_/*V*_EA_ = 2:1; ^1^H NMR (500 MHz, chloroform-*d*) δ 7.76–7.69 (m, 3H), 7.68–7.61 (m, 3H), 7.47 (t, *J* = 7.4 Hz, 1H), 7.39 (t, *J* = 7.4 Hz, 1H), 7.35–7.30 (m, 1H), 6.86 (s, 1H), 5.91 (d, *J* = 5.5 Hz, 1H), 4.92 (s, 1H), 3.54–3.45 (m, 1H), 3.41–3.34 (m, 1H), 3.30–3.24 (m, 2H), 1.43 (s, 9H). ^13^C{^1^H} NMR (126 MHz, CDCl_3_) δ 168.2, 166.6, 141.3, 136.2, 133.5, 133.5, 133.4, 131.7, 130.1, 128.8, 128.7, 127.6, 119.5, 117.8, 111.6, 79.8, 56.6, 41.3, 40.0, 28.3. HRMS (ESI) *m*/*z* calculated for C_23_H_26_BrN_4_O_4_ [M + H]^+^: 501.1137, found [M + H]^+^: 501.1132.

#### 2-Bromo-*N*-(2-((3-cyanobenzyl)amino)-1-(2-cyanophenyl)-2-oxoethyl)benzamide (**22a**)

Obtained from a 1 mmol reaction as a yellow solid, 264 mg, yield 56%; ^1^H NMR (500 MHz, chloroform-*d*) δ 7.81–7.72 (m, 3H), 7.69 (td, *J* = 7.7, 1.4 Hz, 1H), 7.65–7.60 (m, 2H), 7.55–7.50 (m, 2H), 7.46–7.43 (m, 1H), 7.42–7.36 (m, 3H), 7.35–7.30 (m, 1H), 7.05 (t, *J* = 6.2 Hz, 1H), 6.09 (d, *J* = 6.4 Hz, 1H), 4.55–4.45 (m, 2H). ^13^C{^1^H} NMR (126 MHz, DMSO-*d*_6_) δ 168.8, 167.5, 141.3, 141.3, 138.4, 133.5, 133.1, 132.5, 131.6, 131.0, 129.9, 129.6, 129.4, 129.1, 128.9, 127.8, 127.5, 119.5, 119.2, 117.8, 112.7, 111.6, 56.04 42.2. HRMS (ESI) *m*/*z* calculated for C_24_H_18_BrN_4_O_2_ [M + H]^+^: 473.0613, found [M + H]^+^: 473.0607.

#### 2-Bromo-*N*-(2-((2-chloro-3,6-difluorobenzyl)amino)-1-(2-cyanophenyl)-2-oxoethyl) Benzamide (**23a**)

Obtained from a 1 mmol reaction as a white solid, 263 mg, yield 51%; ^1^H NMR (500 MHz, chloroform-*d*) δ 7.73 (dd, *J* = 7.8, 1.4 Hz, 1H), 7.71–7.57 (m, 5H), 7.47 (td, *J* = 7.6, 1.4 Hz, 1H), 7.38 (td, *J* = 7.6, 1.3 Hz, 1H), 7.32 (td, *J* = 7.7, 1.8 Hz, 1H), 7.15–7.07 (m, 1H), 7.00 (td, *J* = 8.9, 4.1 Hz, 1H), 6.56 (t, *J* = 6.2 Hz, 1H), 5.94 (d, *J* = 6.0 Hz, 1H), 4.77–4.58 (m, 2H). ^13^C{^1^H} NMR (126 MHz, CDCl_3_) δ 167.6, 166.5, 164.3 (d, *J* = 279.3 Hz), 163.7 (d, *J* = 281.6 Hz), 141.1, 136.1, 133.6, 133.6 (d, *J* = 2.5 Hz), 133.2, 131.8, 130.0, 128.9, 128.0, 127.6, 124.0 (d, *J* = 6.0 Hz), 119.4, 117.9, 116.4 (dd, *J* = 32.0, 3.1 Hz), 114.9 (dd, *J* = 37.0, 7,5 Hz) , 111.5, 56.3, 35.6. HRMS (ESI) *m*/*z* calculated for C_23_H_16_BrClF_2_N_3_O_2_ [M + H]^+^: 518.0082, found [M + H]^+^: 518.0078.

#### 2-Bromo-*N*-(1-(2-cyanophenyl)-2-oxo-2-((2-(trifluoromethoxy)benzyl)amino)ethyl) Benzamide (**24a**)

Obtained from a 1 mmol reaction as a white solid, 207 mg, yield 39%; ^1^H NMR (500 MHz, chloroform-*d*) δ 7.73–7.67 (m, 3H), 7.63 (td, *J* = 7.7, 1.4 Hz, 1H), 7.61–7.58 (m, 2H), 7.46 (td, *J* = 7.6, 1.4 Hz, 1H), 7.39–7.27 (m, 4H), 7.24–7.19 (m, 2H), 6.45 (t, *J* = 5.7 Hz, 1H), 5.96 (d, *J* = 6.0 Hz, 1H), 4.59–4.48 (m, 2H). ^13^C{^1^H} NMR (126 MHz, DMSO-*d*_6_) δ 170.1, 168.4, 167.1, 146.1, 140.8, 138.0, 131.3, 130.1 (q, *J* = 254.5 Hz), 129.2, 128.8, 128.6, 127.4, 127.0, 120.6, 119.1, 118.6, 117.4, 112.3, 55.6, 37.1. HRMS (ESI) *m*/*z* calculated for C_24_H_18_BrF_3_N_3_O_3_ [M + H]^+^: 532.0484, found [M + H]^+^: 532.0479.

#### *N*-(2-(benzylamino)-1-(2-cyanophenyl)-2-oxoethyl)-2-bromo-4-methylbenzamide (**25a**)

Obtained from a 1 mmol reaction as a white solid, 310 mg, yield 67%; ^1^H NMR (500 MHz, chloroform-*d*) δ 7.81 (d, *J* = 6.2 Hz, 1H), 7.76–7.70 (m, 2H), 7.66 (td, *J* = 7.7, 1.4 Hz, 1H), 7.56 (d, *J* = 7.8 Hz, 1H), 7.50–7.43 (m, 2H), 7.34–7.28 (m, 3H), 7.19 (dt, *J* = 7.9, 2.1 Hz, 3H), 6.47 (t, *J* = 5.9 Hz, 1H), 5.98 (d, *J* = 6.0 Hz, 1H), 4.56–4.42 (m, 2H), 2.38 (s, 3H). ^13^C{^1^H} NMR (126 MHz, CDCl_3_) δ 167.9, 166.4, 142.6, 141.6, 137.0, 134.0, 133.6, 133.2, 132.9, 130.2, 128.8, 128.8, 128.4, 128.0, 127.7, 127.5, 119.3, 118.0, 111.5, 56.5, 44.1, 20.9. HRMS (ESI) *m*/*z* calculated for C_24_H_21_BrN_3_O_2_ [M + H]^+^: 462.0817, found [M + H]^+^: 462.0813.

#### *N*-(2-(benzylamino)-1-(2-cyanophenyl)-2-oxoethyl)-2-bromothiophene-3-carboxamide (**26a**)

Obtained from a 1 mmol reaction as a white solid, 220 mg, yield 49%; ^1^H NMR (500 MHz, chloroform-*d*) ^1^H NMR (500 MHz, chloroform-*d*) δ 8.29 (d, *J* = 5.8 Hz, 1H), 7.71–7.66 (m, 2H), 7.62 (t, *J* = 7.7 Hz, 1H), 7.43 (t, *J* = 7.6 Hz, 1H), 7.35 (d, *J* = 5.8 Hz, 1H), 7.31–7.21 (m, 4H), 7.18–7.12 (m, 2H), 6.43 (t, *J* = 6.7 Hz, 1H), 5.93 (d, *J* = 5.7 Hz, 1H), 4.53–4.39 (m, 2H). ^13^C{^1^H} NMR (126 MHz, CDCl_3_) δ 168.0, 161.0, 141.7, 137.0, 134.6, 133.7, 133.2, 129.6, 128.8, 128.7, 127.9, 127.7, 127.4, 126.2, 117.9, 113.7, 111.5, 56.3, 44.1. HRMS (ESI) *m*/*z* calculated for C_21_H_17_BrN_3_O_2_S [M + H]^+^: 454.0225, found [M + H]^+^: 454.0219.

#### *N*-(2-(benzylamino)-1-(2-cyanophenyl)-2-oxoethyl)-4-bromo-2-iodobenzamide (**27a**)

Obtained from a 1 mmol reaction as a yellow solid, 377 mg, yield 66%; ^1^H NMR (500 MHz, chloroform-*d*) δ 8.01 (d, *J* = 1.9 Hz, 1H), 7.73–7.68 (m, 2H), 7.61 (td, *J* = 1.5 Hz, 1H), 7.59 (d, *J* = 6.2 Hz, 1H), 7.50 (dd, *J* = 8.2, 1.9 Hz, 1H), 7.45 (td, *J* = 7.7, 1.2 Hz, 1H), 7.41 (dd, *J* = 8.1, 1.9 Hz, 1H), 7.32 (d, *J* = 8.3 Hz, 1H), 7.30–7.27 (m, 1H), 7.26–7.23 (m, 1H), 7.15 (dd, *J* = 7.7, 1.9 Hz, 2H), 6.60 (t, *J* = 5.9 Hz, 1H), 5.95 (d, *J* = 6.2 Hz, 1H), 4.51–4.36 (m, 2H). ^13^C{^1^H} NMR (126 MHz, CDCl_3_) δ 167.7, 167.5, 142.2, 141.0, 139.3, 136.9, 133.5, 133.4, 131.4, 129.7, 129.0, 128.7, 128.3, 127.8, 127.5, 124.9, 117.8, 111.6, 92.9, 56.3, 44.1. HRMS (ESI) *m*/*z* calculated for C_23_H_18_BrIN_3_O_2_ [M + H]^+^: 573.9627, found [M + H]^+^: 573.9622.

#### *N*-(2-(benzylamino)-1-(2-cyanophenyl)-2-oxoethyl)-2-bromo-5-methylbenzamide (**28a**)

Obtained from a 1 mmol reaction as a white solid, 203 mg, yield 45%; ^1^H NMR (500 MHz, chloroform-*d*) δ 7.71 (d, *J* = 9.0 Hz, 3H), 7.64 (td, *J* = 7.7, 1.4 Hz, 1H), 7.49–7.40 (m, 3H), 7.32–7.25 (m, 3H), 7.19–7.14 (m, 2H), 7.10 (dd, *J* = 8.3, 2.3 Hz, 1H), 6.43 (s, 1H), 5.95 (d, *J* = 6.0 Hz, 1H), 4.55–4.39 (m, 2H), 2.31 (s, 3H). ^13^C{^1^H} NMR (126 MHz, CDCl_3_) δ 167.8, 166.6, 141.5, 137.8, 136.9, 135.7, 133.6, 133.3, 133.2, 132.6, 130.8, 128.8, 128.8, 128.0, 127.7, 127.5, 117.9, 116.0, 111.6, 56.5, 44.1, 20.7. HRMS (ESI) *m*/*z* calculated for C_24_H_21_BrN_3_O_2_ [M + H]^+^: 462.0817, found [M + H]^+^: 462.0808.

#### *N*-(2-(benzylamino)-1-(2-cyanophenyl)-2-oxoethyl)-4-bromonicotinamide (**29a**)

Obtained from a 1 mmol reaction as a white solid, 200 mg, yield 45%; ^1^H NMR (500 MHz, chloroform-*d*) δ 8.72 (s, 1H), 8.40 (d, *J* = 5.3 Hz, 1H), 8.08 (d, *J* = 6.1 Hz, 1H), 7.71 (d, *J* = 8.9 Hz, 1H), 7.64 (td, *J* = 7.7, 1.4 Hz, 1H), 7.54 (d, *J* = 5.3 Hz, 1H), 7.50–7.43 (m, 1H), 7.31–7.24 (m, 3H), 7.15 (dd, *J* = 7.6, 1.9 Hz, 2H), 6.76 (t, *J* = 5.9 Hz, 1H), 6.01 (d, *J* = 6.2 Hz, 1H), 4.51–4.37 (m, 2H). ^13^C{^1^H} NMR (126 MHz, CDCl_3_) δ 167.6, 164.1, 151.6, 150.3, 141.1, 136.9, 133.6, 133.3, 132.2, 130.3, 129.0, 128.7, 128.3, 127.9, 127.7, 127.4, 117.8, 111.7, 56.3, 44.1. HRMS (ESI) *m*/*z* calculated for C_22_H_18_BrN_4_O_2_ [M + H]^+^: 449.0613, found [M + H]^+^: 449.0606.

#### *N*-(2-(benzylamino)-1-(2-cyanophenyl)-2-oxoethyl)-1-bromo-2-naphthamide (**30a**)

Obtained from a 1 mmol reaction as a light yellow solid, 260 mg, yield 52%; ^1^H NMR (500 MHz, chloroform-*d*) δ 8.36 (d, *J* = 8.5 Hz, 1H), 7.89–7.83 (m, 2H), 7.78 (dd, *J* = 8.0, 1.2 Hz, 1H), 7.75 (dd, *J* = 7.7, 1.4 Hz, 1H), 7.71–7.66 (m, 3H), 7.65–7.59 (m, 1H), 7.54 (d, *J* = 8.4 Hz, 1H), 7.49 (td, *J* = 7.7, 1.2 Hz, 1H), 7.37–7.27 (m, 3H), 7.22–7.17 (m, 2H), 6.62 (t, *J* = 6.0 Hz, 1H), 6.07 (d, *J* = 6.3 Hz, 1H), 4.55–4.42 (m, 2H). ^13^C{^1^H} NMR (126 MHz, CDCl_3_) δ 167.8, 167.7, 141.3, 137.0, 134.9, 134.8, 133.6, 133.3, 131.9, 128.9, 128.8, 128.4, 128.3, 128.3, 128.2, 127.9, 127.8, 127.7, 127.5, 125.1, 120.27, 117.9, 111.6, 56.4, 44.1. HRMS (ESI) *m*/*z* calculated for C_27_H_21_BrN_3_O_2_ [M + H]^+^: 498.0817, found [M + H]^+^: 498.0811.

#### *N*-(2-(benzylamino)-1-(2-cyanophenyl)-2-oxoethyl)-3-bromoisonicotinamide (**31a**)

Obtained from a 1 mmol reaction as a yellow solid, 360 mg, yield 80%; ^1^H NMR (500 MHz, DMSO-*d*_6_) δ 9.64 (d, *J* = 7.4 Hz, 1H), 8.93 (t, *J* = 5.9 Hz, 1H), 8.81 (s, 1H), 8.65 (d, *J* = 4.8 Hz, 1H), 7.91 (dd, *J* = 7.7, 1.4 Hz, 1H), 7.74 (td, *J* = 7.7, 1.4 Hz, 1H), 7.62 (dd, *J* = 7.9, 1.2 Hz, 1H), 7.56 (td, *J* = 7.6, 1.2 Hz, 1H), 7.53 (d, *J* = 4.9 Hz, 1H), 7.36–7.23 (m, 5H), 5.94 (d, *J* = 7.4 Hz, 1H), 4.44–4.34 (m, 2H). ^13^C{^1^H} NMR (126 MHz, DMSO-*d*_6_) δ 168.2, 165.6, 152.1, 149.0, 145.2, 141.1, 139.3, 133.6, 129.3, 128.9, 128.8, 128.7, 127.7, 127.3, 123.8, 117.8, 117.7, 112.7, 55.8, 43.0. HRMS (ESI) *m*/*z* calculated for C_22_H_18_BrN_4_O_2_ [M + H]^+^: 449.0613, found [M + H]^+^: 449.0605.

#### *N*-(2-(benzylamino)-1-(2-cyano-4-methoxyphenyl)-2-oxoethyl)-2-bromobenzamide (**32a**)

Obtained from a 1 mmol reaction as a light yellow solid, 277 mg, yield 58%; ^1^H NMR (500 MHz, chloroform-*d*) δ 7.69 (d, *J* = 6.0 Hz, 1H), 7.64–7.59 (m, 3H), 7.38 (td, *J* = 7.5, 1.2 Hz, 1H), 7.35–7.29 (m, 4H), 7.23–7.15 (m, 4H), 6.41 (t, *J* = 5.8 Hz, 1H), 5.91 (d, *J* = 6.0 Hz, 1H), 4.55–4.41 (m, 2H), 3.86 (s, 3H). ^13^C{^1^H} NMR (126 MHz, CDCl_3_) δ 168.1, 166.5, 159.4, 137.0, 136.3, 133.5, 133.5, 131.7, 130.0, 129.5, 128.7, 127.7, 127.6, 127.5, 120.0, 119.4, 117.7, 117.6, 112.4, 55.9, 55.7, 44.1. HRMS (ESI) *m*/*z* calculated for C_24_H_21_BrN_3_O_3_ [M + H]^+^: 478.0766, found [M + H]^+^: 478.0760.

#### *N*-(2-(benzylamino)-1-(2-cyano-4-fluorophenyl)-2-oxoethyl)-2-bromobenzamide (**33a**)

Obtained from a 1 mmol reaction as a white solid, 273 mg, yield 60%; ^1^H NMR (500 MHz, chloroform-*d*) δ 7.74–7.68 (m, 2H), 7.61–7.56 (m, 2H), 7.41–7.37 (m, 1H), 7.37–7.31 (m, 2H), 7.31–7.27 (m, 3H), 7.27–7.26 (m, 1H), 7.17 (dd, 2H), 6.46 (t, *J* = 5.9 Hz, 1H), 5.92 (d, *J* = 6.0 Hz, 1H), 4.54–4.37 (m, 2H). ^13^C{^1^H} NMR (126 MHz, CDCl_3_) δ 167.6, 166.5, 161.7(d, *J* = 252.9 Hz), 137.6 (d, *J* = 4.0 Hz), 136.8, 136.0, 133.6, 131.9, 130.3(d, *J* = 8.7 Hz), 130.1, 128.8, 127.7 (d, *J* = 26.4 Hz), 121.3(d, *J* = 21.2 Hz), 119.9 (d, *J* = 24.8 Hz), 119.4, 55.9, 44.2. HRMS (ESI) *m*/*z* calculated for C_23_H_18_BrFN_3_O_2_ [M + H]^+^: 466.0566, found [M + H]^+^: 466.0559.

#### 2-Bromo-*N*-(2-((2-chloro-6-fluoro-3-methylbenzyl)amino)-1-(2-cyanophenyl)-2-oxoethyl)benzamide (**34a**)

Obtained from a 10 mmol reaction as a light yellow solid, 2.6 g, yield 51%; ^1^H NMR (500 MHz, chloroform-*d*) δ 7.71–7.68 (m, 1H), 7.68–7.65 (m, 1H), 7.63–7.58 (m, 1H), 7.58–7.55 (m, 1H), 7.54–7.49 (m, 1H), 7.43 (td, *J* = 7.6, 1.3 Hz, 1H), 7.35 (td, *J* = 7.5, 1.2 Hz, 1H), 7.28 (td, *J* = 7.8, 1.9 Hz, 1H), 7.25–7.13 (m, 2H), 6.90 (t, *J* = 8.7 Hz, 1H), 6.47 (t, *J* = 5.5 Hz, 1H), 5.89 (d, *J* = 6.0 Hz, 1H), 4.80–4.47 (m, 2H), 2.32 (s, 3H). ^13^C{^1^H} NMR (126 MHz, DMSO-*d*_6_) δ 170.3, 167.8, 166.8, 160.0 (d, *J* = 246.5.4 Hz), 143.6, 141.0, 138.1, 134.7 (d, *J* = 5.7 Hz), 133.0, 132.4, 132.1 (d, *J* = 3.7 Hz), 131.1, 128.5 (d, *J* = 32.6 Hz), 126.9, 123.1, 123.0, 119.0, 118.6, 117.3, 113.8 (d, *J* = 23.7 Hz), 112.4, 55.3, 35.1, 19.7. HRMS (ESI) *m*/*z* calculated for C_24_H_19_BrClFN_3_O_2_ [M + H]^+^: 514.0333, found [M + H]^+^: 514.0326.

#### *N*-(2-(benzylamino)-1-(2-cyanoquinolin-3-yl)-2-oxoethyl)-2-bromobenzamide (**35a**)

Obtained from a 1 mmol reaction as a yellow solid, 214 mg, yield 43%; eluent: *V*_PE_/*V*_EA_ = 1:1; ^1^H NMR (500 MHz, DMSO-*d*_6_) δ 9.45 (d, *J* = 7.3 Hz, 1H), 8.95 (t, *J* = 5.9 Hz, 1H), 8.50 (s, 1H), 8.18 (d, *J* = 8.5 Hz, 1H), 8.10 (d, *J* = 8.1 Hz, 1H), 7.97 (t, *J* = 7.7 Hz, 1H), 7.84 (t, *J* = 7.6 Hz, 1H), 7.67 (d, *J* = 8.0 Hz, 1H), 7.57 (dd, *J* = 7.6, 1.8 Hz, 1H), 7.47 (t, *J* = 7.5 Hz, 1H), 7.39 (td, *J* = 7.7, 1.8 Hz, 1H), 7.35–7.29 (m, 4H), 7.27–7.20 (m, 1H), 6.10 (d, *J* = 7.3 Hz, 1H), 4.56–4.35 (m, 2H). ^13^C{^1^H} NMR (126 MHz, DMSO-*d*_6_) δ 168.1, 167.5, 147.0, 139.3, 138.3, 137.2, 137.1, 134.6, 133.3, 133.1, 132.2, 131.7, 130.5, 129.8, 129.3, 128.7, 128.5, 127.8, 127.4, 119.5, 116.6, 54.5, 43.1. HRMS (ESI) *m*/*z* calculated for C_26_H_20_BrN_4_O_2_ [M + H]^+^: 499.0770, found [M + H]^+^: 499.0764.

#### *N*-Cyano-*N*-(2-(cyclohexylamino)-1-(2-iodophenyl)-2-oxoethyl)benzamide (**39a**)

Obtained from a 1 mmol reaction as a white solid, 135 mg, yield 38%; eluent: *V*_PE_/*V*_EA_ = 2:1; ^1^H NMR (500 MHz, chloroform-*d*) δ 8.14–8.09 (m, 2H), 7.87 (dd, *J* = 8.0, 1.2 Hz, 1H), 7.63–7.56 (m, 2H), 7.45 (t, *J* = 7.8 Hz, 2H), 7.39 (td, *J* = 7.6, 1.3 Hz, 1H), 7.05 (td, *J* = 7.7, 1.7 Hz, 1H), 6.44 (s, 1H), 6.12 (d, *J* = 8.3 Hz, 1H), 3.91–3.78 (m, 1H), 2.02–1.95 (m, 1H), 1.88–1.81 (m, 1H), 1.73–1.54 (m, 3H), 1.43–1.30 (m, 2H), 1.28–1.22 (m, 1H), 1.20–1.10 (m, 2H). ^13^C{^1^H} NMR (126 MHz, CDCl_3_) δ 166.4, 165.2, 139.9, 138.5, 133.6, 130.6, 129.9, 129.3, 129.1, 128.7, 128.5, 98.9, 78.9, 48.5, 32.8, 32.7, 25.4, 24.6. HRMS (ESI) *m*/*z* calculated for C_22_H_23_IN_3_O_2_ [M + H]^+^: 488.0835, found [M + H]^+^: 488.0828.

#### *N*-(2-(benzylamino)-1-(2-iodophenyl)-2-oxoethyl)-*N*-cyanobenzamide (**40a**)

Obtained from a 1 mmol reaction as a white solid, 188 mg, yield 38%; eluent: *V*_PE_/*V*_EA_ = 2:1; ^1^H NMR (500 MHz, chloroform-*d*) δ 7.98 (dd, *J* = 8.0, 1.3 Hz, 1H), 7.92–7.88 (m, 2H), 7.62 (t, *J* = 7.5 Hz, 1H), 7.54 (dd, *J* = 7.8, 1.7 Hz, 1H), 7.50 (t, *J* = 7.8 Hz, 2H), 7.42 (td, *J* = 7.6, 1.3 Hz, 1H), 7.37–7.32 (m, 2H), 7.32–7.30 (m, 1H), 7.28–7.26 (m, 2H), 7.16 (td, *J* = 7.7, 1.6 Hz, 1H), 6.24 (t, *J* = 5.9 Hz, 1H), 6.11 (s, 1H), 4.62–4.47 (m, 2H). ^13^C{^1^H} NMR (126 MHz, CDCl_3_) δ 168.5, 166.4, 140.4, 137.2, 134.7, 133.4, 131.9, 131.0, 130.4, 129.2, 129.0, 128.8, 128.6, 127.8, 127.7, 108.9, 102.1, 66.5, 44.1. HRMS (ESI) *m/z* calculated for C_23_H_19_IN_3_O_2_ [M + H]^+^: 496.0522, found [M + H]^+^: 496.0513.

#### *N*-Cyano-*N*-(1-(2-iodophenyl)-2-oxo-2-(((tetrahydrofuran-2-yl)methyl)amino)ethyl) Benzamide (**41a**)

Obtained from a 1 mmol reaction as a white solid, 171 mg, yield 35%; eluent: *V*_PE_/*V*_EA_ = 2:1; ^1^H NMR (500 MHz, DMSO-*d*_6_) δ 8.94–8.86 (m, 1H), 8.12–8.07 (m, 1H), 7.89–7.79 (m, 2H), 7.72 (t, *J* = 7.5 Hz, 1H), 7.61 (t, *J* = 7.8 Hz, 2H), 7.52 (t, *J* = 7.6 Hz, 1H), 7.41–7.35 (m, 1H), 7.24 (t, *J* = 7.6 Hz, 1H), 5.98 (s, 1H), 3.97–3.87 (m, 1H), 3.82–3.71 (m, 1H), 3.67–3.59 (m, 1H), 3.53–3.30 (m, 1H), 3.30–3.15 (m, 1H), 1.99–1.87 (m, 1H), 1.86–1.74 (m, 2H), 1.61–1.49 (m, 1H). ^13^C{^1^H} NMR (126 MHz, DMSO-*d*_6_) δ 168.5, 166.9, 140.4, 136.1, 133.9, 131.9, 131.0, 130.9, 129.3, 129.2, 128.8, 109.6, 103.5, 77.3, 67.7, 66.0, 43.5, 28.9, 25.6. HRMS (ESI) *m*/*z* calculated for C_21_H_21_IN_3_O_3_ [M + H]^+^: 490.0628, found [M + H]^+^: 490.0621.

#### *N*-Cyclohexyl-10-oxo-10,12-Dihydroisoindolo[1,2-*b*]quinazoline-12-carboxamide (**10b**)

Obtained from a 0.2 mmol reaction as a white solid, 58 mg, yield 81%; eluent: *V*_PE_/*V*_EA_ = 2:1; ^1^H NMR (500 MHz, DMSO-*d*_6_) δ 8.82 (d, *J* = 7.9 Hz, 1H), 8.21 (d, *J* = 8.9 Hz, 1H), 8.11 (d, *J* = 7.6 Hz, 1H), 7.92–7.87 (m, 1H), 7.84 (d, *J* = 7.8 Hz, 1H), 7.78–7.65 (m, 3H), 7.58 (t, *J* = 7.4 Hz, 1H), 6.00 (s, 1H), 3.55 (m, 1H), 1.90–1.81 (m, 1H), 1.78–1.66 (m, 3H), 1.62–1.52 (m, 1H), 1.40–1.15 (m, 5H). ^13^C{^1^H} NMR (126 MHz, DMSO-*d*_6_) δ 164.6, 159.3, 155.5, 149.5, 141.1, 135.0, 133.2, 132.4, 130.0, 127.7, 127.0, 126.4, 123.4, 123.1, 121.2, 64.1, 48.6, 48.5, 32.7, 24.8. HRMS (ESI) *m*/*z* calculated for C_22_H_22_N_3_O_2_ [M + H]^+^: 360.1712, found [M + H]^+^: 360.1705.

#### *N*-Benzyl-10-oxo-10,12-Dihydroisoindolo[1,2-*b*]quinazoline-12-carboxamide (**11b**)

Obtained from a 0.2 mmol reaction as a white solid, 30 mg, yield 41%; eluent: *V*_PE_/*V*_EA_ = 2:1; ^1^H NMR (500 MHz, DMSO-*d*_6_) δ 9.38 (t, *J* = 5.8 Hz, 1H), 8.25 (d, *J* = 7.9 Hz, 1H), 8.13 (d, *J* = 7.6 Hz, 1H), 7.91 (td, *J* = 7.7, 6.9, 1.5 Hz, 1H), 7.87–7.84 (m, 1H), 7.79–7.73 (m, 2H), 7.69 (td, *J* = 7.1, 6.3, 1.8 Hz, 1H), 7.59 (t, *J* = 7.5 Hz, 1H), 7.38–7.26 (m, 5H), 6.09 (s, 1H), 4.45–4.31 (m, 2H). ^13^C{^1^H} NMR (126 MHz, DMSO-*d*_6_) δ 165.8, 159.5, 155.4, 149.5, 140.8, 139.1, 135.0, 133.2, 132.4, 130.1, 128.8, 127.7, 127.6, 127.4, 127.0, 126.5, 123.5, 123.4, 121.2, 64.2, 42.9. HRMS (ESI) *m*/*z* calculated for C_23_H_18_N_3_O_2_ [M + H]^+^: 368.1399, found [M + H]^+^: 368.1391.

#### 10-oxo-*N*-Phenethyl-10,12-Dihydroisoindolo[1,2-*b*]quinazoline-12-carboxamide (**12b**)

Obtained from a 0.2 mmol reaction as a light yellow solid, 40 mg, yield 70%; eluent: *V*_DCM_/*V*_MeOH_ = 20:1; ^1^H NMR (500 MHz, DMSO-*d*_6_) δ 8.93 (t, *J* = 5.5 Hz, 1H), 8.23 (dd, *J* = 7.9, 1.6 Hz, 1H), 8.11–8.08 (m, 1H), 7.93–7.88 (m, 1H), 7.84 (dd, *J* = 8.2, 1.2 Hz, 1H), 7.71–7.63 (m, 2H), 7.59 (ddd, *J* = 8.2, 7.0, 1.3 Hz, 1H), 7.51 (d, *J* = 6.9 Hz, 1H), 7.34–7.29 (m, 2H), 7.26–7.22 (m, 3H), 5.98 (s, 1H), 3.47–3.36 (m, 2H), 2.78 (t, *J* = 7.1 Hz, 2H). ^13^C{^1^H} NMR (126 MHz, DMSO-*d*_6_) δ 169.5, 165.6, 159.4, 155.4, 149.5, 140.8, 139.6, 134.9, 133.1, 132.3, 131.1, 130.0, 129.2, 128.8, 127.7, 127.0, 126.6, 126.5, 123.4, 121.2, 119.0, 64.2, 41.0, 35.2. HRMS (ESI) *m*/*z* calculated for C_24_H_20_N_3_O_2_ [M + H]^+^: 382.1556, found [M + H]^+^: 382.1548.

#### *N*-(4-Chlorophenyl)-10-oxo-10,12-dihydroisoindolo[1,2-*b*] Quinazoline-12-carboxamide (**13b**)

Obtained from a 0.15 mmol reaction as a white solid, 45 mg, yield 78%; eluent: *V*_DCM_/*V*_MeOH_ = 20:1; ^1^H NMR (500 MHz, chloroform-*d*) δ 10.14 (s, 1H), 8.41 (d, *J* = 7.7 Hz, 1H), 8.14 (d, *J* = 7.6 Hz, 1H), 7.89–7.78 (m, 3H), 7.67 (td, *J* = 7.6, 1.3 Hz, 1H), 7.63–7.58 (m, 1H), 7.56–7.49 (m, 1H), 7.44–7.39 (m, 2H), 7.12–7.07 (m, 2H), 6.33 (s, 1H). ^13^C{^1^H} NMR (126 MHz, CDCl_3_) δ 163.6, 161.5, 154.5, 149.5, 139.1, 138.2, 136.2, 135.1, 133.6, 132.8, 132.1, 130.0, 128.7, 127.8, 127.0, 126.7, 123.7, 121.0, 120.4, 65.0. HRMS (ESI) *m*/*z* calculated for C_22_H_15_ClN_3_O_2_ [M + H]^+^: 388.0853, found [M + H]^+^: 388.0849.

#### *N*-((3-Methylthiophen-2-yl)methyl)-10-oxo-10,12-dihydroisoindolo[1,2-*b*]quinazoline-12-carboxamide (**14b**)

Obtained from a 0.2 mmol reaction as a white solid, 46 mg, yield 46%; eluent: *V*_PE_/*V*_EA_ = 2:1; ^1^H NMR (500 MHz, DMSO-*d*_6_) δ 9.35 (t, *J* = 5.6 Hz, 1H), 8.22 (dd, *J* = 7.9, 1.6 Hz, 1H), 8.11 (d, *J* = 7.5 Hz, 1H), 7.90 (td, *J* = 7.5, 6.8, 1.6 Hz, 1H), 7.85 (d, *J* = 8.1 Hz, 1H), 7.78–7.65 (m, 3H), 7.59 (t, *J* = 7.5 Hz, 1H), 7.33 (d, *J* = 5.1 Hz, 1H), 6.86 (d, *J* = 5.0 Hz, 1H), 6.04 (s, 1H), 4.45 (d, *J* = 5.6 Hz, 2H), 2.17 (s, 3H). ^13^C{^1^H} NMR (126 MHz, DMSO-*d*_6_) δ 165.4, 159.4, 155.4, 149.5, 140.7, 135.0, 134.8, 134.6, 133.1, 132.4, 130.4, 130.2, 130.0, 127.7, 126.9, 126.5, 123.9, 123.5, 121.2, 64.0, 36.2, 13.7. HRMS (ESI) *m*/*z* calculated for C_22_H_18_N_3_O_2_S [M + H]^+^: 388.1120, found [M + H]^+^: 388.1115.

#### *N*-(2-methoxybenzyl)-10-oxo-10,12-dihydroisoindolo[1,2-*b*]quinazoline-12-carboxamide (**15b**)

Obtained from a 0.17 mmol reaction as a white solid, 35 mg, yield 53%; eluent: *V*_PE_/*V*_EA_ = 2:1; ^1^H NMR (500 MHz, chloroform-*d*) δ 8.36 (dd, *J* = 7.9, 1.6 Hz, 1H), 8.13 (dd, *J* = 6.8, 1.5 Hz, 1H), 7.87–7.78 (m, 2H), 7.74 (d, *J* = 7.6 Hz, 1H), 7.66–7.57 (m, 2H), 7.51 (td, *J* = 7.5, 6.8, 1.5 Hz, 1H), 7.24–7.17 (m, 2H), 6.95 (t, *J* = 6.0 Hz, 1H), 6.85 (td, *J* = 7.5, 1.1 Hz, 1H), 6.78 (d, *J* = 8.7 Hz, 1H), 5.88 (s, 1H), 4.54–4.39 (m, 2H), 3.67 (s, 3H). ^13^C{^1^H} NMR (126 MHz, CDCl_3_) δ 165.2, 157.4, 149.3, 139.5, 136.3, 134.7, 132.6, 132.0, 129.7, 129.7, 129.0, 127.6, 126.7, 126.7, 125.2, 123.9, 123.5, 120.8, 120.6, 118.9, 110.2, 64.5, 55.0, 40.2. HRMS (ESI) *m*/*z* calculated for C_24_H_20_N_3_O_3_ [M + H]^+^: 398.1505, found [M + H]^+^: 398.1495.

#### 10-Oxo-*N*-(pyridin-3-ylmethyl)-10,12-dihydroisoindolo[1,2-*b*]quinazoline-12-carboxamide (**16b**)

Obtained from a 0.29 mmol reaction as a brown solid, 60 mg, yield 56%; eluent: *V*_DCM_/*V*_MeOH_ = 20:1; ^1^H NMR (500 MHz, chloroform-*d*) δ 8.41 (s, 1H), 8.39 (d, *J* = 3.3 Hz, 1H), 8.20 (d, *J* = 8.1 Hz, 1H), 7.99 (d, *J* = 7.6 Hz, 1H), 7.79–7.68 (m, 4H), 7.64–7.57 (m, 2H), 7.53 (t, *J* = 7.5 Hz, 1H), 7.41 (ddd, *J* = 8.1, 6.4, 1.8 Hz, 1H), 7.16 (dd, *J* = 7.8, 4.8 Hz, 1H), 5.88 (s, 1H), 4.53–4.39 (m, 2H). ^13^C{^1^H} NMR (126 MHz, CDCl_3_) δ 166.0, 160.7, 154.5, 149.2, 148.9, 148.9, 139.1, 135.5, 134.8, 133.3, 132.8, 131.9, 129.9, 127.5, 126.9, 126.7, 123.7, 123.6, 123.5, 120.6, 64.3, 41.3. HRMS (ESI) *m*/*z* calculated for C_22_H_17_N_4_O_2_ [M + H]^+^: 369.1352, found [M + H]^+^: 369.1342.

#### *N*-((3*S*,5*S*)-Adamantan-1-yl)-10-oxo-10,12-dihydroisoindolo-[1,2-*b*]quinazoline-12-Carboxamide (**17b**)

Obtained from a 0.19 mmol reaction as a white solid, 80 mg, yield 85%; eluent: *V*_PE_/*V*_EA_ = 2:1; ^1^H NMR (500 MHz, chloroform-*d*) δ 8.35 (dd, *J* = 7.9, 1.4 Hz, 1H), 8.12 (d, *J* = 7.0 Hz, 1H), 7.84–7.76 (m, 2H), 7.69 (d, *J* = 7.5 Hz, 1H), 7.61 (td, *J* = 7.5, 1.3 Hz, 1H), 7.59–7.55 (m, 1H), 7.49 (ddd, *J* = 8.1, 6.7, 1.5 Hz, 1H), 6.40 (s, 1H), 5.76 (s, 1H), 2.08–2.03 (m, 3H), 2.03–1.99 (m, 6H), 1.64 (t, *J* = 3.1 Hz, 6H). ^13^C{^1^H} NMR (126 MHz, CDCl_3_) δ 164.2, 160.6, 154.7, 149.4, 139.8, 134.5, 132.5, 132.2, 129.6, 127.6, 126.7, 126.6, 123.5, 123.4, 120.8, 65.1, 52.9, 41.3, 36.2, 29.4. HRMS (ESI) *m*/*z* calculated for C_26_H_26_N_3_O_2_ [M + H]^+^: 412.2025, found [M + H]^+^: 412.2016.

#### 10-Oxo-*N*-((tetrahydrofuran-2-yl)methyl)-10,12-dihydroisoindolo-[1,2-*b*]quinazoline-12-carboxamide (**18b**)

Obtained from a 0.18 mmol reaction as a white solid, 31 mg, yield 47%, dr ratio = 1:1; eluent: *V*_PE_/*V*_EA_ = 2:1; ^1^H NMR (500 MHz, chloroform-*d*) δ 8.33 (ddd, *J* = 7.9, 4.2, 1.5 Hz, 1H), 8.14 (d, *J* = 8.4 Hz, 1H), 7.85–7.71 (m, 3H), 7.65–7.56 (m, 2H), 7.48 (ddd, *J* = 8.2, 6.9, 1.4 Hz, 1H), 6.80 (dt, *J* = 74.6, 5.7 Hz, 1H), 5.88 (d, *J* = 17.3 Hz, 1H), 4.04–3.89 (m, 1H), 3.81–3.62 (m, 2H), 3.62–3.43 (m, 1H), 3.40–3.23 (m, 1H), 1.96–1.78 (m, 3H), 1.64–1.48 (m, 1H). ^13^C{^1^H} NMR (126 MHz, CDCl_3_) δ 165.7, 160.4, 154.6, 149.3, 139.5, 134.6, 132.6, 132.1, 129.8, 127.6, 126.7, 126.6, 123.6, 123.4, 120.8, 77.5, 68.2, 64.4, 43.3, 28.3, 25.8. HRMS (ESI) *m*/*z* calculated for C_21_H_20_N_3_O_3_ [M + H]^+^: 362.1505, found [M + H]^+^: 362.1499.

#### 10-Oxo-*N*-(2,4,4-trimethylpentan-2-yl)-10,12-dihydroisoindolo-[1,2-*b*]quinazoline-12-carboxamide (**19b**)

Obtained from a 0.2 mmol reaction as a white solid, 85 mg, yield 91%; eluent: *V*_PE_/*V*_EA_ = 2:1; ^1^H NMR (500 MHz, DMSO-*d*_6_) δ 8.46 (s, 1H), 8.22 (dd, *J* = 8.0, 1.5 Hz, 1H), 8.09 (d, *J* = 7.6 Hz, 1H), 7.88 (td, *J* = 7.6, 6.9, 1.6 Hz, 1H), 7.82 (d, *J* = 7.0 Hz, 1H), 7.74 (d, *J* = 4.2 Hz, 2H), 7.68–7.63 (m, 1H), 7.56 (t, *J* = 7.4 Hz, 1H), 6.02 (s, 1H), 1.86 (d, *J* = 14.6 Hz, 1H), 1.57 (d, *J* = 14.6 Hz, 1H), 1.38 (s, 3H), 1.30 (s, 3H), 1.01 (s, 9H). ^13^C{^1^H} NMR (126 MHz, DMSO-*d*_6_) δ 164.2, 159.2, 155.5, 149.5, 141.1, 134.9, 133.0, 132.5, 129.9, 127.7, 126.9, 126.6, 123.4, 121.2, 64.7, 55.4, 50.9, 31.6, 29.5, 29.0. HRMS (ESI) *m*/*z* calculated for C_24_H_28_N_3_O_2_ [M + H]^+^: 390.2182, found [M + H]^+^: 390.2174.

#### Methyl (10-Oxo-10,12-dihydroisoindolo[1,2-*b*]quinazoline-12-carbonyl)glycinate (**20b**)

Obtained from a 0.2 mmol reaction as a white solid, 44 mg, yield 63%; eluent: *V*_PE_/*V*_EA_ = 1:1; ^1^H NMR (500 MHz, DMSO-*d*_6_) δ 9.42 (t, *J* = 6.0 Hz, 1H), 8.21 (d, *J* = 8.0 Hz, 1H), 8.11 (d, *J* = 7.6 Hz, 1H), 7.90 (td, *J* = 7.6, 7.0, 1.5 Hz, 1H), 7.84 (d, *J* = 8.3 Hz, 2H), 7.78 (t, *J* = 7.0 Hz, 1H), 7.69 (t, *J* = 7.5 Hz, 1H), 7.58 (t, *J* = 7.3 Hz, 1H), 6.12 (s, 1H), 4.10–3.95 (m, 2H), 3.63 (s, 3H). ^13^C{^1^H} NMR (126 MHz, DMSO-*d*_6_) δ 170.3, 166.3, 159.3, 155.4, 149.5, 140.6, 135.0, 133.1, 132.3, 130.1, 127.7, 127.0, 126.5, 123.9, 123.4, 121.1, 63.8, 52.3, 41.1. HRMS (ESI) *m*/*z* calculated for C_19_H_16_N_3_O_4_ [M + H]^+^: 350.1141, found [M + H]^+^: 350.1131.

#### *tert*-Butyl-(2-(10-oxo-10,12-dihydroisoindolo[1,2-*b*]quinazoline-12-carboxamido)ethyl)-carbamate (**21b**)

Obtained from a 0.2 mmol reaction as a white solid, 58 mg, yield 70%; eluent: *V*_PE_/*V*_EA_ = 1:1; ^1^H NMR (500 MHz, DMSO-*d*_6_) δ 8.87 (t, *J* = 5.7 Hz, 1H), 8.15 (d, *J* = 8.0 Hz, 1H), 8.05 (d, *J* = 7.6 Hz, 1H), 7.84 (td, *J* = 7.6, 6.9, 1.4 Hz, 1H), 7.78 (d, *J* = 8.2 Hz, 1H), 7.73–7.64 (m, 2H), 7.62 (t, *J* = 7.3 Hz, 1H), 7.52 (td, *J* = 7.5, 6.9, 1.3 Hz, 1H), 6.78 (t, *J* = 5.7 Hz, 1H), 5.94 (s, 1H), 3.26–3.18 (m, 1H), 3.09–3.01 (m, 1H), 2.99–2.93 (m, 2H), 1.33 (s, 9H). ^13^C{^1^H} NMR (126 MHz, DMSO-*d*_6_) δ 165.9, 159.4, 156.0, 155.4, 149.5, 140.8, 135.0, 133.2, 132.3, 130.1, 127.7, 127.0, 126.5, 123.6, 123.4, 121.2, 78.2, 64.2, 28.7, 28.6. HRMS (ESI) *m*/*z* calculated for C_23_H_25_N_4_O_4_ [M + H]^+^: 421.1876, found [M + H]^+^: 421.1868.

#### *N*-(3-Cyanobenzyl)-10-oxo-10,12-dihydroisoindolo[1,2-*b*]quinazoline-12-carboxamide (**22b**)

Obtained from a 0.2 mmol reaction as a light purple solid, 61 mg, yield 78%; eluent: *V*_DCM_/*V*_MeOH_ = 20:1; ^1^H NMR (500 MHz, DMSO-*d*_6_) δ 9.44 (t, *J* = 6.0 Hz, 1H), 8.25 (dd, *J* = 7.9, 1.5 Hz, 1H), 8.14 (d, *J* = 7.6 Hz, 1H), 7.91 (td, *J* = 7.6, 6.9, 1.6 Hz, 1H), 7.86 (d, *J* = 8.2 Hz, 1H), 7.78–7.73 (m, 4H), 7.72–7.69 (m, 1H), 7.65 (d, *J* = 7.9 Hz, 1H), 7.62–7.55 (m, 2H), 6.11 (s, 1H), 4.49–4.38 (m, 2H). ^13^C{^1^H} NMR (126 MHz, DMSO-*d*_6_) δ 166.3, 159.6, 155.3, 149.5, 141.0, 140.6, 135.0, 133.3, 132.4, 132.3, 131.1, 130.9, 130.2, 130.0, 127.8, 127.1, 126.5, 123.6, 123.4, 121.2, 119.2, 111.8, 64.2, 42.2. HRMS (ESI) *m*/*z* calculated for C_24_H_17_N_4_O_2_ [M + H]^+^: 393.1352, found [M + H]^+^: 393.1342.

#### *N*-(2-Chloro-3,6-difluorobenzyl)-10-oxo-10,12-dihydroisoindolo[1,2-*b*]quinazoline-12-carboxamide (**23b**)

Obtained from a 0.2 mmol reaction as a white solid, 32 mg, yield 37%; eluent: *V*_PE_/*V*_EA_ = 2:1; ^1^H NMR (500 MHz, DMSO-*d*_6_) δ 9.38 (t, *J* = 5.1 Hz, 1H), 8.21 (dt, *J* = 7.6, 1.1 Hz, 1H), 8.10 (d, *J* = 7.1 Hz, 1H), 7.92–7.87 (m, 1H), 7.84 (d, *J* = 8.1 Hz, 1H), 7.73 (t, *J* = 6.9 Hz, 1H), 7.69–7.62 (m, 2H), 7.61–7.55 (m, 1H), 7.50 (td, *J* = 8.9, 4.6 Hz, 1H), 7.36 (td, *J* = 9.1, 4.1 Hz, 1H), 6.03 (s, 1H), 4.58–4.42 (m, 2H). ^13^C{^1^H} NMR (126 MHz, DMSO-*d*_6_) δ 165.1, 158.9, 157.0 (dd, *J* = 240.1, 2.1 Hz), 154.9, 154.1 (dd, *J* = 243.0, 2.5 Hz), 149.0, 140.2, 132.0, 129.7, 126.6, 126.1 (d, *J* = 11.8 Hz), 125.0 (d, *J* = 20.0 Hz), 123.0 (dd, *J* = 20.6, 6.5 Hz), 121.4 (dd, *J* = 20.0, 5.4 Hz), 120.7, 63.3, 35.0. HRMS (ESI) *m*/*z* calculated for C_23_H_15_ClF_2_N_3_O_2_ [M + H]^+^: 438.0821, found [M + H]^+^: 438.0814.

#### 10-Oxo-*N*-(2-(trifluoromethoxy)benzyl)-10,12-dihydroisoindolo[1,2-*b*]quinazoline-12-carboxamide (**24b**)

Obtained from a 0.2 mmol reaction as a white solid, 62 mg, yield 69%; eluent: *V*_PE_/*V*_EA_ = 2:1; ^1^H NMR (500 MHz, DMSO-*d*_6_) δ 9.42 (t, *J* = 5.8 Hz, 1H), 8.25 (dd, *J* = 7.9, 1.5 Hz, 1H), 8.13 (dd, *J* = 7.6, 1.0 Hz, 1H), 7.90 (ddd, *J* = 8.4, 6.7, 1.6 Hz, 1H), 7.85 (dd, *J* = 8.1, 1.2 Hz, 1H), 7.76 (d, *J* = 4.0 Hz, 2H), 7.73–7.67 (m, 1H), 7.59 (d, *J* = 7.4 Hz, 1H), 7.54 (dd, *J* = 6.8, 2.5 Hz, 1H), 7.47–7.39 (m, 2H), 7.40–7.35 (m, 1H), 6.12 (s, 1H), 4.50–4.37 (m, 2H). ^13^C{^1^H} NMR (126 MHz, DMSO-*d*_6_) δ 165.8, 159.1, 154.9, 149.1, 146.1, 140.2, 133.7 (q, *J* = 241.0 Hz), 132.0, 131.0, 129.4, 129.0, 127.5, 126.6, 126.1, 123.1, 120.8, 120.6, 119.2, 63.8, 37.2. HRMS (ESI) *m*/*z* calculated for C_24_H_17_F_3_N_3_O_3_ [M + H]^+^: 452.1222, found [M + H]^+^: 452.1216.

#### *N*-Benzyl-7-methyl-10-oxo-10,12-dihydroisoindolo[1,2-*b*]quinazoline-12-carboxamide (**25b**)

Obtained from a 0.2 mmol reaction as a white solid, 29 mg, yield 38%; eluent: *V*_PE_/*V*_EA_ = 2:1; ^1^H NMR (500 MHz, DMSO-*d*_6_) δ 9.36 (t, *J* = 6.0 Hz, 1H), 8.16–8.08 (m, 2H), 7.79–7.72 (m, 2H), 7.72–7.64 (m, 2H), 7.41 (dd, *J* = 8.2, 1.6 Hz, 1H), 7.38–7.34 (m, 2H), 7.33–7.30 (m, 2H), 7.30–7.25 (m, 1H), 6.07 (s, 1H), 4.43–4.32 (m, 2H), 2.48 (s, 3H). ^13^C{^1^H} NMR (126 MHz, DMSO-*d*_6_) δ 165.9, 159.4, 155.4, 149.6, 145.5, 140.8, 139.2, 132.5, 128.8, 128.4, 127.6, 127.5, 127.4, 126.4, 126.3, 123.5, 123.4, 123.3, 118.9, 64.0, 42.9, 21.8. HRMS (ESI) *m*/z calculated for C_24_H_20_N_3_O_2_ [M + H]^+^: 382.1556, found [M + H]^+^: 382.1550.

#### *N*-Benzyl-4-oxo-4,6-dihydrothieno[2′,3′:4,5]pyrimido[2,1-*a*]isoindole-6-carboxamide (**26b**)

Obtained from a 0.2 mmol reaction as a white solid, 38 mg, yield 51%; eluent: *V*_PE_/*V*_EA_ = 2:1; ^1^H NMR (500 MHz, DMSO-*d*_6_) δ 9.37 (t, *J* = 6.0 Hz, 1H), 8.09 (d, *J* = 7.6 Hz, 1H), 7.78–7.73 (m, 2H), 7.71–7.67 (m, 1H), 7.65 (d, *J* = 5.8 Hz, 1H), 7.51 (d, *J* = 5.7 Hz, 1H), 7.39–7.30 (m, 4H), 7.30–7.25 (m, 1H), 6.07 (s, 1H), 4.46–4.30 (m, 2H). ^13^C{^1^H} NMR (126 MHz, DMSO-*d*_6_) δ 165.5, 165.2, 156.2, 156.1, 140.9, 139.1, 133.0, 131.8, 130.2, 128.8, 127.6, 127.4, 124.6, 123.3, 123.0, 122.3, 122.2, 64.2, 43.0. HRMS (ESI) *m*/*z* calculated for C_21_H_16_N_3_O_2_S [M + H]^+^: 374.0963, found [M + H]^+^: 374.0957.

#### *N*-Benzyl-7-bromo-10-oxo-10,12-dihydroisoindolo[1,2-*b*]quinazoline-12-carboxamide (**27b**)

Obtained from a 0.2 mmol reaction as a white solid, 39 mg, yield 44%; eluent: *V*_PE_/*V*_EA_ = 2:1; ^1^H NMR (500 MHz, DMSO-*d*_6_) δ 9.36 (t, *J* = 5.9 Hz, 1H), 8.16 (d, *J* = 8.5 Hz, 1H), 8.12 (d, *J* = 7.5 Hz, 1H), 8.06 (d, *J* = 1.9 Hz, 1H), 7.82–7.73 (m, 3H), 7.71 (t, *J* = 7.4 Hz, 1H), 7.39–7.25 (m, 5H), 6.09 (s, 1H), 4.45–4.30 (m, 2H). ^13^C{^1^H} NMR (126 MHz, DMSO-*d*_6_) δ 170.3, 165.1, 158.6, 156.2, 150.3, 140.6, 138.7, 133.2, 133.1, 131.6, 129.7, 129.5, 128.3, 128.1, 127.2, 127.0, 123.2, 123.0, 119.9, 63.9, 42.5. HRMS (ESI) *m*/*z* calculated for C_23_H_17_BrN_3_O_2_ [M + H]^+^: 446.0504, found [M + H]^+^: 446.0498.

#### *N*-Benzyl-8-methyl-10-oxo-10,12-dihydroisoindolo[1,2-*b*]quinazoline-12-carboxamide (**28b**)

Obtained from a 0.2 mmol reaction as a white solid, 22 mg, yield 29%; eluent: *V*_PE_/*V*_EA_ = 2:1; ^1^H NMR (500 MHz, chloroform-*d*) δ 8.12 (s, 1H), 8.05 (d, *J* = 7.6 Hz, 1H), 7.77 (dd, *J* = 7.6, 1.0 Hz, 1H), 7.69 (d, *J* = 8.2 Hz, 1H), 7.66–7.54 (m, 3H), 7.29–7.26 (m, 1H), 7.25–7.18 (m, 4H), 6.93 (t, *J* = 5.9 Hz, 1H), 5.89 (s, 1H), 4.54–4.42 (m, 2H), 2.51 (s, 3H). ^13^C{^1^H} NMR (126 MHz, CDCl_3_) δ 165.7, 160.7, 153.8, 147.2, 139.2, 137.4, 137.1, 136.2, 132.4, 132.1, 129.8, 128.6, 127.6, 127.5, 127.3, 126.2, 123.8, 123.3, 120.4, 64.4, 43.9, 21.3. HRMS (ESI) *m*/*z* calculated for C_24_H_20_N_3_O_2_ [M + H]^+^: 382.1556, found [M + H]^+^: 382.1549.

#### *N*-Benzyl-12-oxo-10,12-dihydropyrido[4′,3′:4,5]pyrimido[2,1-*a*]isoindole-10-carboxamide (**29b**)

Obtained from a 0.15 mmol reaction as a white solid, 40 mg, yield 73%; eluent: *V*_PE_/*V*_EA_ = 2:1; ^1^H NMR (500 MHz, DMSO-*d*_6_) δ 9.39 (t, *J* = 4.7 Hz, 2H), 8.91 (d, *J* = 5.7 Hz, 1H), 8.17 (d, *J* = 7.6 Hz, 1H), 7.85–7.81 (m, 1H), 7.80–7.70 (m, 3H), 7.38–7.33 (m, 2H), 7.33–7.26 (m, 3H), 6.13 (s, 1H), 4.45–4.31 (m, 2H). ^13^C{^1^H} NMR (126 MHz, DMSO-*d*_6_) δ 165.3, 159.5, 158.9, 154.7, 154.0, 149.9, 141.5, 139.0, 134.2, 131.8, 130.4, 128.8, 127.6, 127.5, 124.2, 123.5, 121.0, 116.9, 64.6, 43.0. HRMS (ESI) *m*/z calculated for C_22_H_17_N_4_O_2_ [M + H]^+^: 369.1352, found [M + H]^+^: 369.1343.

#### *N*-Benzyl-7-oxo-7,9-dihydrobenzo[*h*]isoindolo[1,2-*b*]quinazoline-9-carboxamide (**30b**)

Obtained from a 0.3 mmol reaction as a light pink solid, 63 mg, yield 50%; eluent: *V*_DCM_/*V*_MeOH_ = 20:1; ^1^H NMR (500 MHz, DMSO-*d*_6_) δ 9.42 (t, *J* = 5.9 Hz, 1H), 9.20–9.13 (m, 1H), 8.30 (d, *J* = 7.4 Hz, 1H), 8.19 (d, *J* = 8.7 Hz, 1H), 8.14–8.09 (m, 1H), 8.02 (d, *J* = 8.7 Hz, 1H), 7.86–7.82 (m, 2H), 7.81–7.74 (m, 3H), 7.40–7.31 (m, 4H), 7.31–7.26 (m, 1H), 6.17 (s, 1H), 4.45–4.34 (m, 2H). ^13^C{^1^H} NMR (126 MHz, DMSO-*d*_6_) δ 165.7, 159.5, 155.8, 147.9, 140.9, 139.1, 136.2, 133.3, 132.6, 130.2, 129.9, 129.7, 128.8, 128.5, 127.6, 127.4, 127.0, 125.2, 123.7, 123.6, 121.8, 117.4, 64.5, 43.0. HRMS (ESI) *m*/*z* calculated for C_27_H_20_N_3_O_2_ [M + H]^+^: 418.1556, found [M + H]^+^: 418.1548.

#### *N*-Benzyl-5-oxo-5,7-dihydropyrido[3′,4′:4,5]pyrimido[2,1-*a*]isoindole-7-carboxamide (**31b**)

Obtained from a 0.2 mmol reaction as a white solid, 54 mg, yield 74%; eluent: *V*_DCM_/*V*_MeOH_ = 20:1; ^1^H NMR (500 MHz, DMSO-*d*_6_) δ 9.37 (t, *J* = 5.9 Hz, 1H), 9.24 (s, 1H), 8.74 (d, *J* = 5.1 Hz, 1H), 8.17 (d, *J* = 7.6 Hz, 1H), 8.09 (dd, *J* = 5.2, 0.9 Hz, 1H), 7.85–7.69 (m, 3H), 7.38–7.33 (m, 2H), 7.32–7.25 (m, 3H), 6.12 (s, 1H), 4.44–4.31 (m, 2H). ^13^C{^1^H} NMR (126 MHz, DMSO-*d*_6_) δ 165.4, 158.6, 157.2, 150.9, 146.4, 144.2, 140.8, 139.1, 133.8, 131.9, 130.3, 128.8, 127.6, 127.4, 126.2, 123.8, 123.4, 118.8, 64.5, 43.0. HRMS (ESI) *m*/*z* calculated for C_22_H_17_N_4_O_2_ [M + H]^+^: 369.1352, found [M + H]^+^: 369.1346.

#### *N*-Benzyl-3-methoxy-10-oxo-10,12-dihydroisoindolo[1,2-*b*]quinazoline-12-carboxamide (**32b**)

Obtained from a 0.2 mmol reaction as a white solid, 56 mg, yield 71%; eluent: *V*_DCM_/*V*_MeOH_ = 20:1; ^1^H NMR (500 MHz, DMSO-*d*_6_) δ 9.31 (t, *J* = 5.9 Hz, 1H), 8.24 (dd, *J* = 7.9, 1.5 Hz, 1H), 7.90 (td, *J* = 6.9, 1.5 Hz, 1H), 7.85 (m, 1H), 7.65–7.56 (m, 3H), 7.38–7.34 (m, 2H), 7.34–7.30 (m, 3H), 7.29–7.27 (m, 1H), 5.99 (s, 1H), 4.37 (t, *J* = 5.7 Hz, 2H), 3.93 (s, 3H). ^13^C{^1^H} NMR (126 MHz, DMSO-*d*_6_) δ 169.5, 166.0, 161.0, 159.4, 155.3, 149.4, 139.8, 139.2, 134.9, 133.8, 133.1, 131.1, 128.8, 127.6, 127.4, 126.5, 121.3, 121.1, 106.3, 63.6, 56.2, 42.9. HRMS (ESI) *m*/*z* calculated for C_24_H_20_N_3_O_3_ [M + H]^+^: 398.1505, found [M + H]^+^: 398.1501.

#### *N*-Benzyl-3-fluoro-10-oxo-10,12-dihydroisoindolo[1,2-*b*]quinazoline-12-carboxamide (**33b**)

Obtained from a 0.15 mmol reaction as a white solid, 38 mg, yield 66%; eluent: *V*_PE_/*V*_EA_ = 2:1; ^1^H NMR (500 MHz, DMSO-*d*_6_) δ 9.36 (t, *J* = 5.9 Hz, 1H), 8.25 (dd, *J* = 7.9, 1.5 Hz, 1H), 7.95–7.90 (m, 2H), 7.86 (d, *J* = 8.1 Hz, 1H), 7.79–7.75 (m, 1H), 7.65–7.58 (m, 2H), 7.38–7.33 (m, 2H), 7.33–7.26 (m, 3H), 6.07 (s, 1H), 4.44–4.30 (m, 2H). ^13^C{^1^H} NMR (126 MHz, CDCl_3_) δ 165.2, 162.9 (d, *J* = 246.4 Hz), 158.9, 154.1(d, *J* = 4.0 Hz), 148.8, 138.6, 136.3, 134.1(d, *J* = 9.8 Hz), 127.3 (d, *J* = 20.2 Hz), 127.1 (d, *J* = 21.9 Hz), 126.1, 120.9, 63.4, 42.5. HRMS (ESI) *m*/*z* calculated for C_23_H_17_FN_3_O_2_ [M + H]^+^: 386.1305, found [M + H]^+^: 386.1299.

#### *N*-(2-chloro-6-fluoro-3-methylbenzyl)-10-oxo-10,12-dihydroisoindolo[1,2-*b*]quinazoline-12-carboxamide (**34b**)

Obtained from a 5 mmol reaction as a white solid, 0.88 g, yield 41%; eluent: *V*_PE_/*V*_EA_ = 2:1; ^1^H NMR (500 MHz, DMSO-*d*_6_) δ 9.27 (t, *J* = 5.0 Hz, 1H), 8.22 (d, *J* = 8.0 Hz, 1H), 8.10 (d, *J* = 7.6 Hz, 1H), 7.89 (t, *J* = 7.4 Hz, 1H), 7.84 (d, *J* = 8.1 Hz, 1H), 7.73 (t, *J* = 7.5 Hz, 1H), 7.69–7.62 (m, 2H), 7.59 (t, *J* = 7.3 Hz, 1H), 7.41 (t, *J* = 7.3 Hz, 1H), 7.20 (t, *J* = 8.9 Hz, 1H), 6.04 (s, 1H), 4.59–4.40 (m, 2H), 2.36 (s, 3H). ^13^C{^1^H} NMR (126 MHz, DMSO-*d*_6_) δ 165.0, 159.5 (d, *J* = 246.7 Hz), 158.9, 154.9, 149.0, 140.3, 134.7, 132.2 (d, *J* = 3.4 Hz), 132.0, 131.1, 129.7 (d, *J* = 27.2 Hz), 126.6 (d, *J* = 20.3 Hz), 126.0, 123.0, 122.8, 120.7, 113.9 (d, *J* = 22.8 Hz), 63.5, 35.4, 19.7. HRMS (ESI) *m*/*z* calculated for C_24_H_18_ClFN_3_O_2_ [M + H]^+^: 434.1072, found [M + H]^+^: 434.1067.

#### *N*-Benzyl-11-oxo-11,13-dihydroquinolino[2′,3′:3,4]pyrrolo[2,1-b]quinazoline-13-carboxamide (**35b**)

Obtained from a 0.2 mmol reaction as a white solid, 42 mg, yield 51%; eluent: *V*_PE_/*V*_EA_ = 2:1; ^1^H NMR (500 MHz, DMSO-*d*_6_) δ 9.48 (t, *J* = 5.9 Hz, 1H), 8.71 (s, 1H), 8.34–8.29 (m, 2H), 8.25 (d, *J* = 8.1 Hz, 1H), 8.03–7.94 (m, 3H), 7.82 (t, *J* = 7.5 Hz, 1H), 7.68 (ddd, *J* = 8.2, 5.9, 2.3 Hz, 1H), 7.36–7.32 (m, 4H), 7.30–7.25 (m, 1H), 6.27 (s, 1H), 4.48–4.31 (m, 2H). ^13^C{^1^H} NMR (126 MHz, DMSO-*d*_6_) δ 168.1, 167.5, 147.0, 139.3, 138.3, 137.2, 137.1, 134.6, 133.3, 133.1, 132.2, 131.7, 130.5, 129.8, 129.3, 128.7, 128.5, 127.8, 127.4, 119.5, 116.6, 54.5, 43.1. HRMS (ESI) *m*/*z* calculated for C_26_H_19_N_4_O_2_ [M + H]^+^: 419.1508, found [M + H]^+^: 419.1500.

#### *N*-Cyclohexyl-10-oxo-10,12-dihydroisoindolo[1,2-*b*]quinazoline-12-carboxamide (**39b**)

Obtained from a 0.15 mmol reaction as a white solid, 21 mg, yield 40%; eluent: *V*_PE_/*V*_EA_ = 2:1; ^1^H NMR (500 MHz, DMSO-*d*_6_) δ 8.78 (d, *J* = 7.8 Hz, 1H), 8.20 (dd, *J* = 7.9, 1.5 Hz, 1H), 8.09 (d, *J* = 7.6 Hz, 1H), 7.88 (ddd, *J* = 8.5, 6.9, 1.6 Hz, 1H), 7.83 (d, *J* = 6.9 Hz, 1H), 7.77–7.70 (m, 2H), 7.66 (t, *J* = 7.4 Hz, 1H), 7.56 (ddd, *J* = 8.1, 7.0, 1.3 Hz, 1H), 5.99 (s, 1H), 3.60–3.50 (m, 1H), 1.88–1.81 (m, 1H), 1.78–1.67 (m, 3H), 1.59–1.53 (m, 1H), 1.38–1.20 (m, 5H). ^13^C{^1^H} NMR (126 MHz, DMSO-*d*_6_) δ 164.6, 159.3, 155.5, 149.5, 141.1, 135.0, 133.2, 132.4, 130.0, 127.7, 127.0, 126.5, 123.4, 123.2, 121.2, 64.1, 48.5, 32.6, 25.6, 24.7. HRMS (ESI) *m*/*z* calculated for C_22_H_22_N_3_O_2_ [M + H]^+^: 360.1712, found [M + H]^+^: 360.1706.

#### *N*-Benzyl-10-oxo-10,12-dihydroisoindolo[1,2-*b*]quinazoline-12-carboxamide (**40b**)

Obtained from a 0.15 mmol reaction as a white solid, 26 mg, yield 47%; eluent: *V*_PE_/*V*_EA_ = 2:1; ^1^H NMR (500 MHz, DMSO-*d*_6_) δ 9.37 (t, *J* = 5.9 Hz, 1H), 8.24 (dd, *J* = 7.9, 1.5 Hz, 1H), 8.13 (d, *J* = 7.6 Hz, 1H), 7.90 (ddd, *J* = 8.5, 7.0, 1.6 Hz, 1H), 7.85 (d, *J* = 8.1 Hz, 1H), 7.79–7.73 (m, 2H), 7.69 (ddd, *J* = 8.6, 6.5, 2.0 Hz, 1H), 7.59 (ddd, *J* = 8.0, 7.0, 1.3 Hz, 1H), 7.38–7.30 (m, 4H), 7.30–7.25 (m, 1H), 6.09 (s, 1H), 4.45–4.29 (m, 2H). ^13^C{^1^H} NMR (126 MHz, DMSO-*d*_6_) δ 165.9, 159.5, 155.4, 149.5, 140.8, 139.1, 135.0, 133.3, 133.1, 132.4, 130.2, 128.8, 127.6, 127.4, 127.0, 126.6, 126.5, 123.5, 121.2, 64.1, 42.9. HRMS (ESI) *m*/*z* calculated for C_23_H_18_N_3_O_2_ [M + H]^+^: 368.1399, found [M + H]^+^: 368.1393.

#### 10-Oxo-*N*-((tetrahydrofuran-2-yl)methyl)-10,12-dihydroisoindolo[1,2-*b*]quinazoline-12-carboxamide (**41b**)

Obtained from a 0.15 mmol reaction as a white solid, 18 mg, yield 33%; eluent: *V*_PE_/*V*_EA_ = 2:1; ^1^H NMR (500 MHz, DMSO-*d*_6_) δ 9.04 (q, *J* = 5.8 Hz, 1H), 8.21 (d, *J* = 7.9 Hz, 1H), 8.11 (d, *J* = 7.7 Hz, 1H), 7.90 (td, *J* = 7.5, 6.9, 1.6 Hz, 1H), 7.84 (d, *J* = 8.0 Hz, 1H), 7.78–7.72 (m, 2H), 7.70–7.66 (m, 1H), 7.58 (t, *J* = 7.4 Hz, 1H), 6.09 (s, 1H), 3.92–3.80 (m, 2H), 3.72–3.62 (m, 1H), 3.32–3.17 (m, 2H), 1.94–1.78 (m, 4H). ^13^C{^1^H} NMR (126 MHz, DMSO-*d*_6_) δ 165.9, 159.3, 155.4, 149.5, 141.0, 134.9, 133.1, 132.4, 130.1, 127.7, 127.0, 126.5, 123.5, 123.4, 121.2, 77.5, 67.8, 64.1, 43.3, 28.7, 25.8. HRMS (ESI) *m*/*z* calculated for C_21_H_20_N_3_O_3_ [M + H]^+^: 362.1505, found [M + H]^+^: 362.1495.

## Supporting Information Available

Characterization data and copies of NMR spectra (PDF)
