# Supplementary material for: A Bifurcated Multicomponent Synthesis Approach to Polycyclic Quinazolinones
Source: J Org Chem. 2022 Sep 12;87(19):13023–33. doi: 10.1021/acs.joc.2c01561 (PMC9552225; doi:10.1021/acs.joc.2c01561)
Supplement: Supplementary file 1 — jo2c01561_si_001.pdf [file jo2c01561_si_001.pdf]

Supporting information

A bifurcated multicomponent synthesis approach to polycyclic quinazolinones

Ruixue Xu†, Zefeng Wang†, Qiang Zheng, Pravin Patil, Alexander Dömling*

University of Groningen, Department of Drug Design, A. Deusinglaan 1, 9713 AV, Groningen, The Netherlands

†These authors contributed equally.

*E-mail: a.s.s.domling@rug.nl, www.drugdesign.nl.

Table of Contents

^{1}H NMR & ^{13}C NMR chromatograms of U-4CR products 2

^{1}H NMR & ^{13}C NMR chromatograms of cyclization products 31

^{1}H NMR & ^{13}C{^{1}H} NMR chromatograms of U-4CR products

2-bromo-N-(1-(2-cyanophenyl)-2-(cyclohexylamino)-2-oxoethyl)benzamide (**10a**)

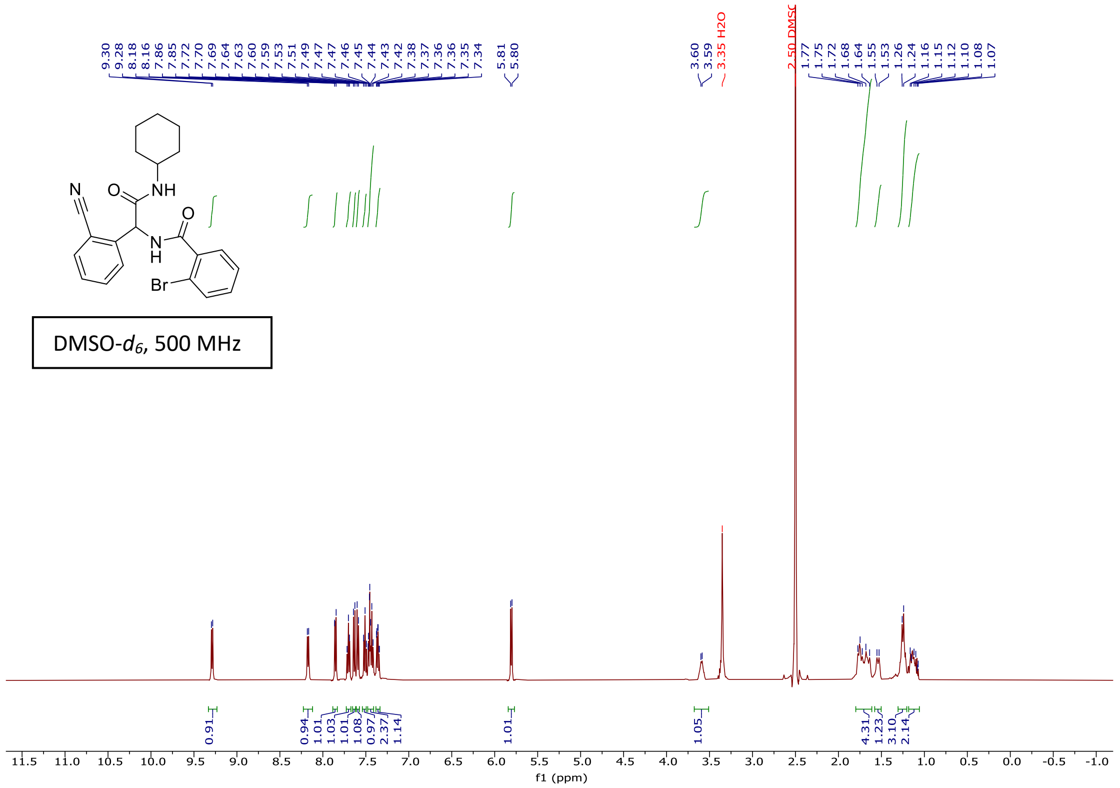

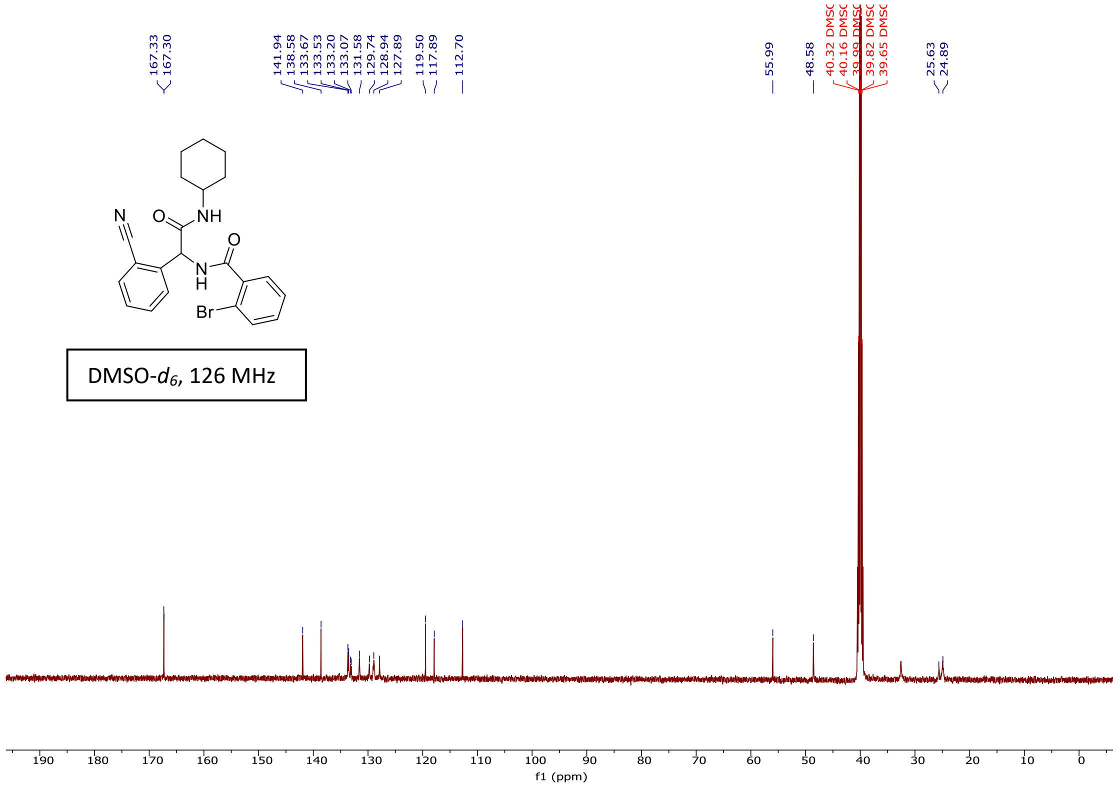

N-(2-(benzylamino)-1-(2-cyanophenyl)-2-oxoethyl)-2-bromobenzamide (**11a**)

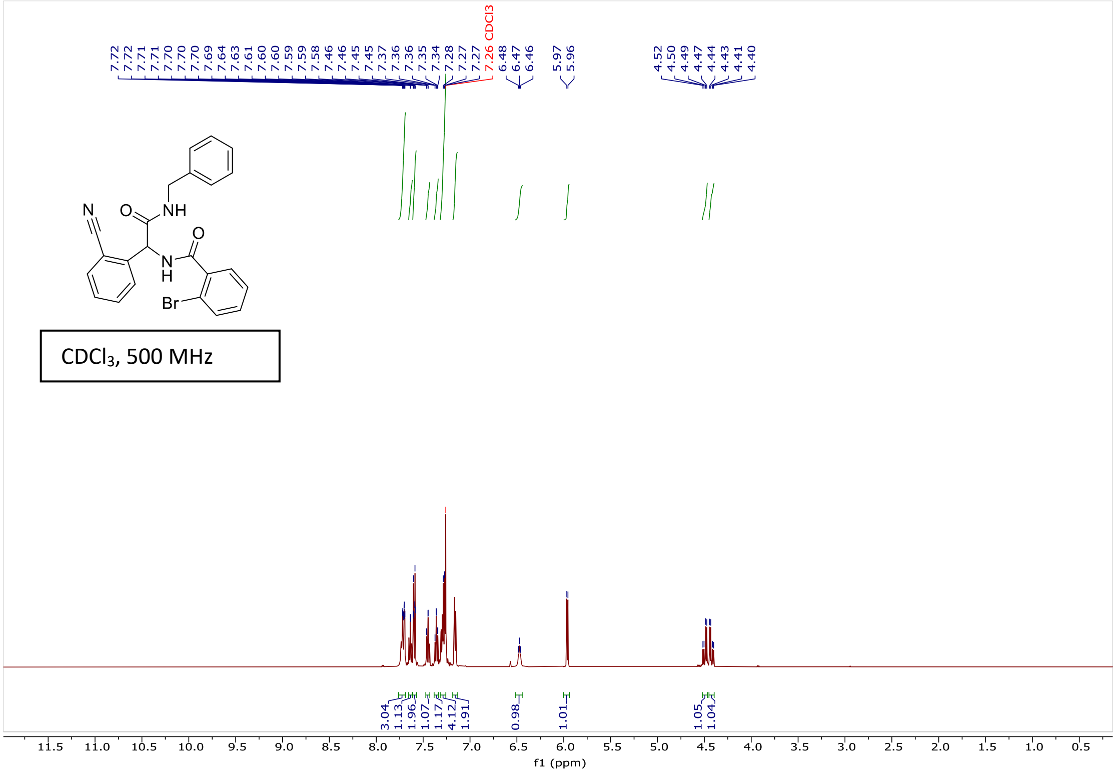

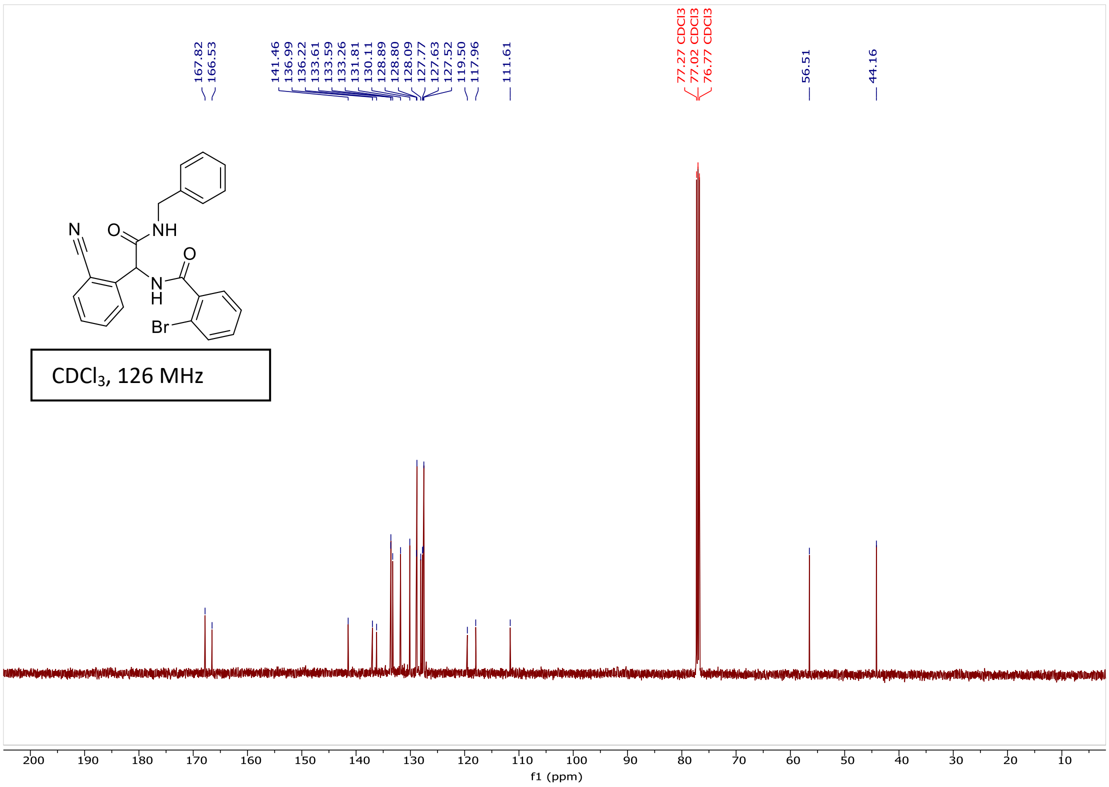

2-bromo-N-(1-(2-cyanophenyl)-2-oxo-2-(phenethylamino)ethyl)benzamide (**12a**)

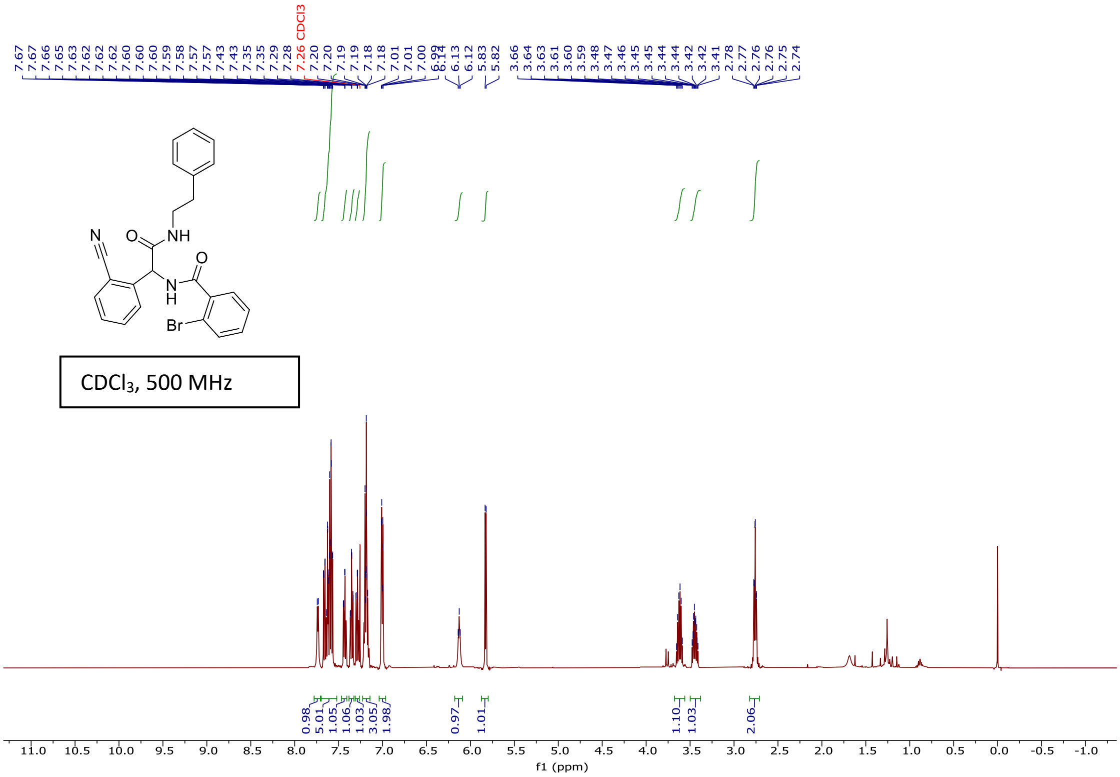

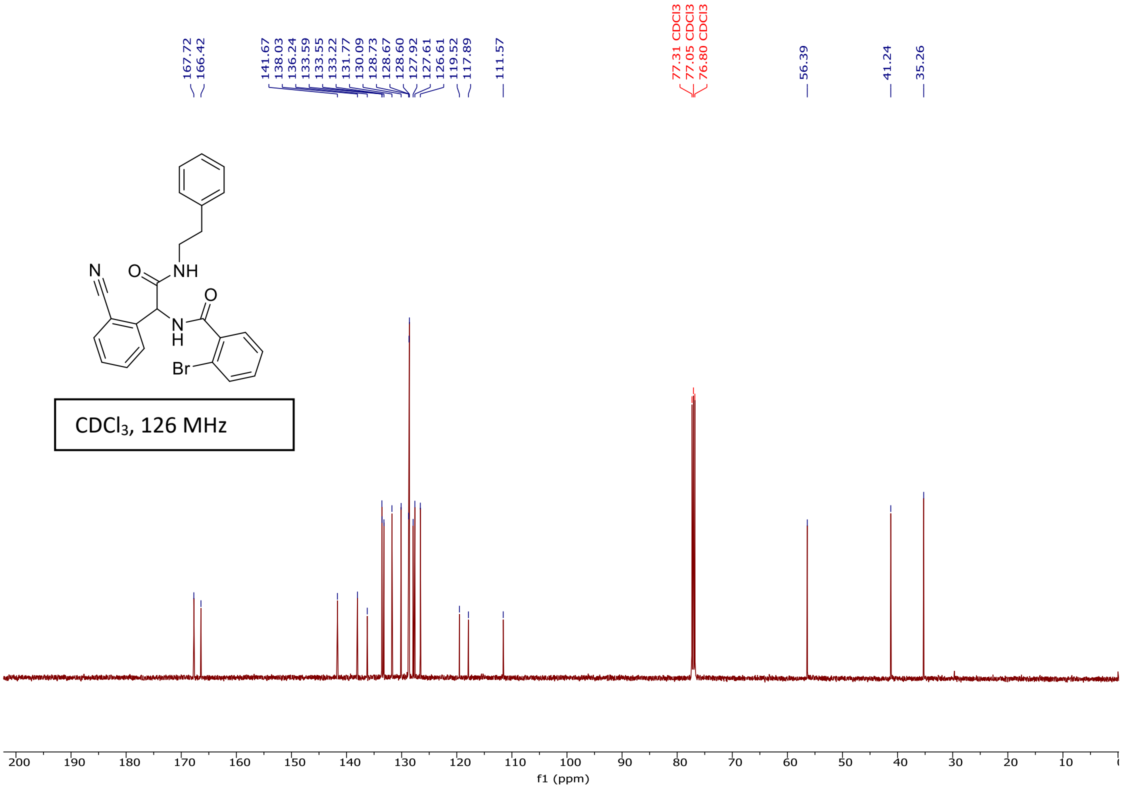

2-bromo-N-(2-((4-chlorophenyl)amino)-1-(2-cyanophenyl)-2-oxoethyl)benzamide (**13a**)

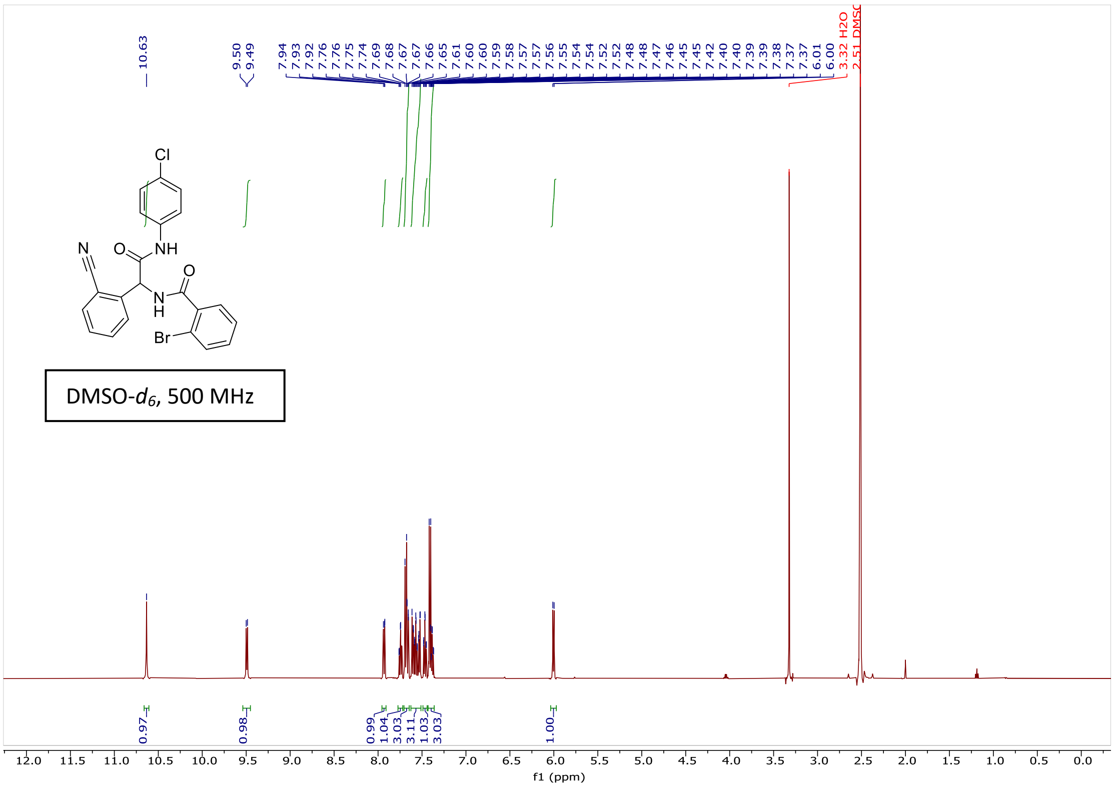

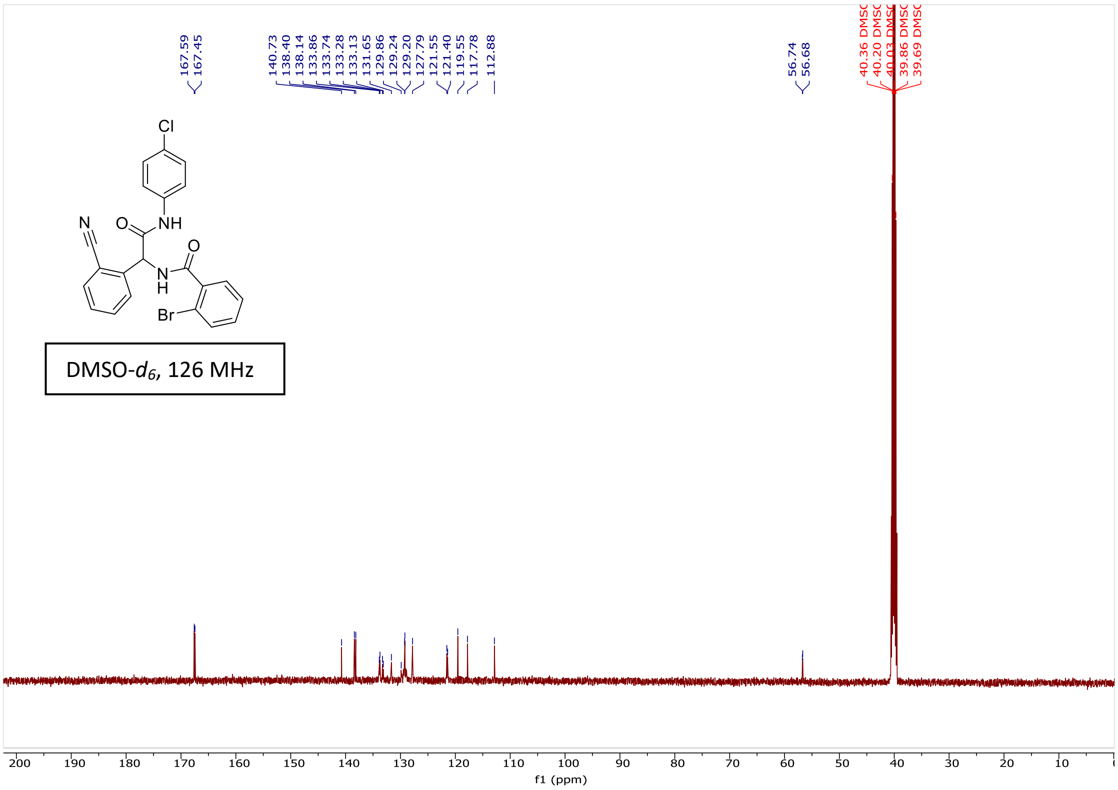

2-bromo-N-(1-(2-cyanophenyl)-2-(((3-methylthiophen-2-yl)methyl)amino)-2-oxoethyl) benzamide (**14a**)

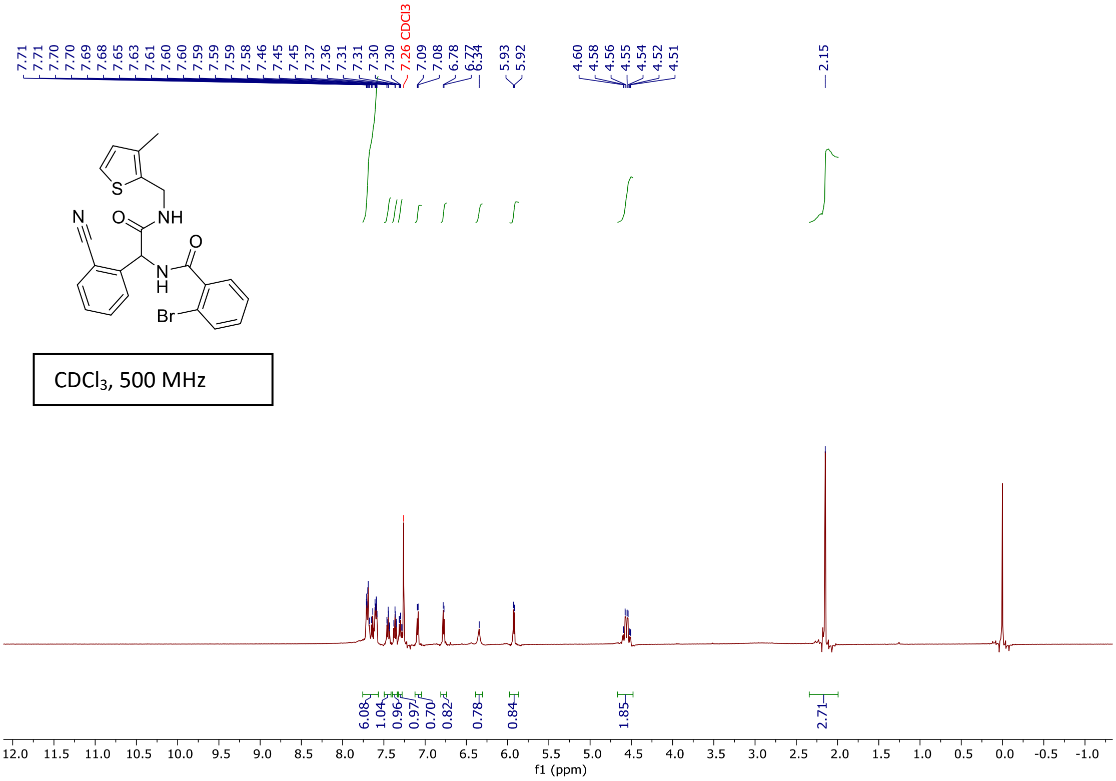

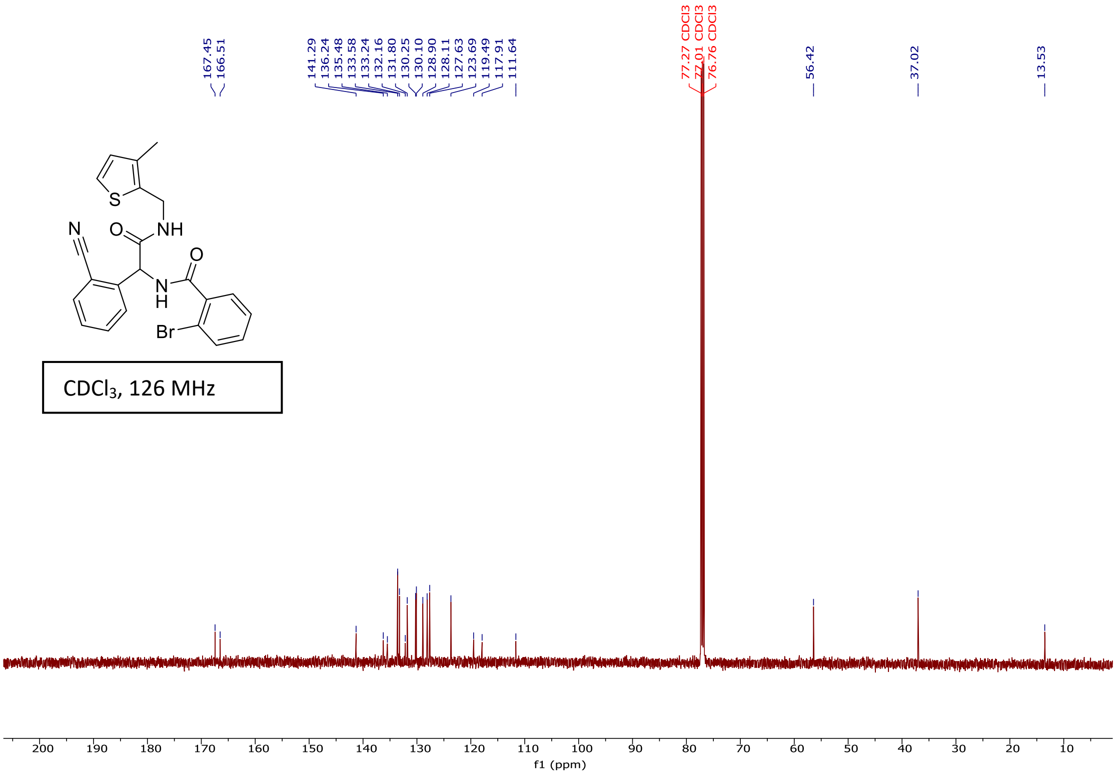

2-bromo-N-(1-(2-cyanophenyl)-2-((2-methoxybenzyl)amino)-2-oxoethyl)benzamide (**15a**)

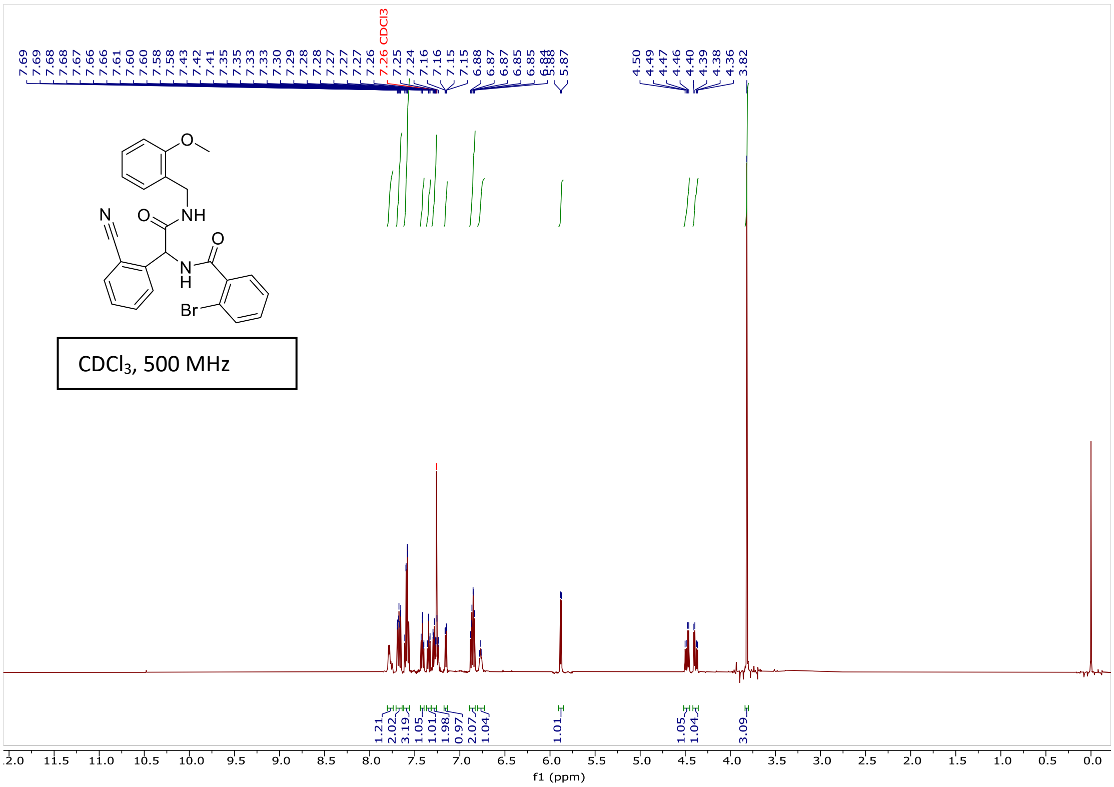

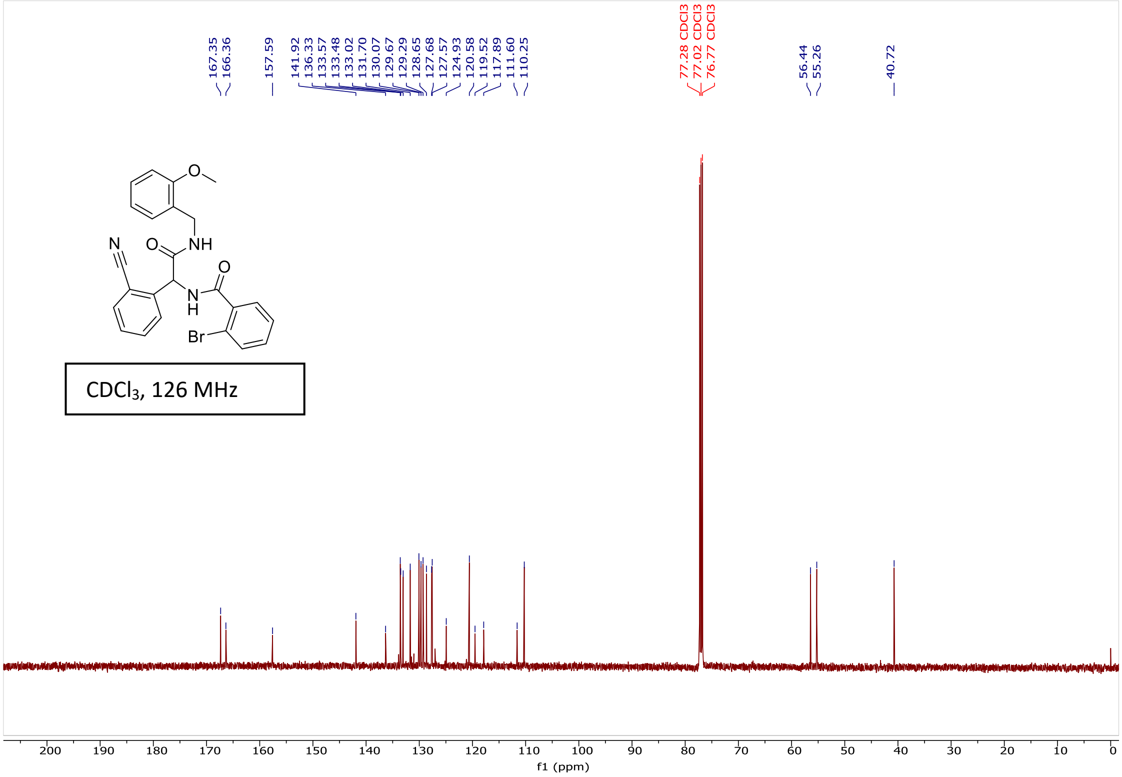

2-bromo-N-(1-(2-cyanophenyl)-2-oxo-2-((pyridin-3-ylmethyl)amino)ethyl)benzamide (**16a**)

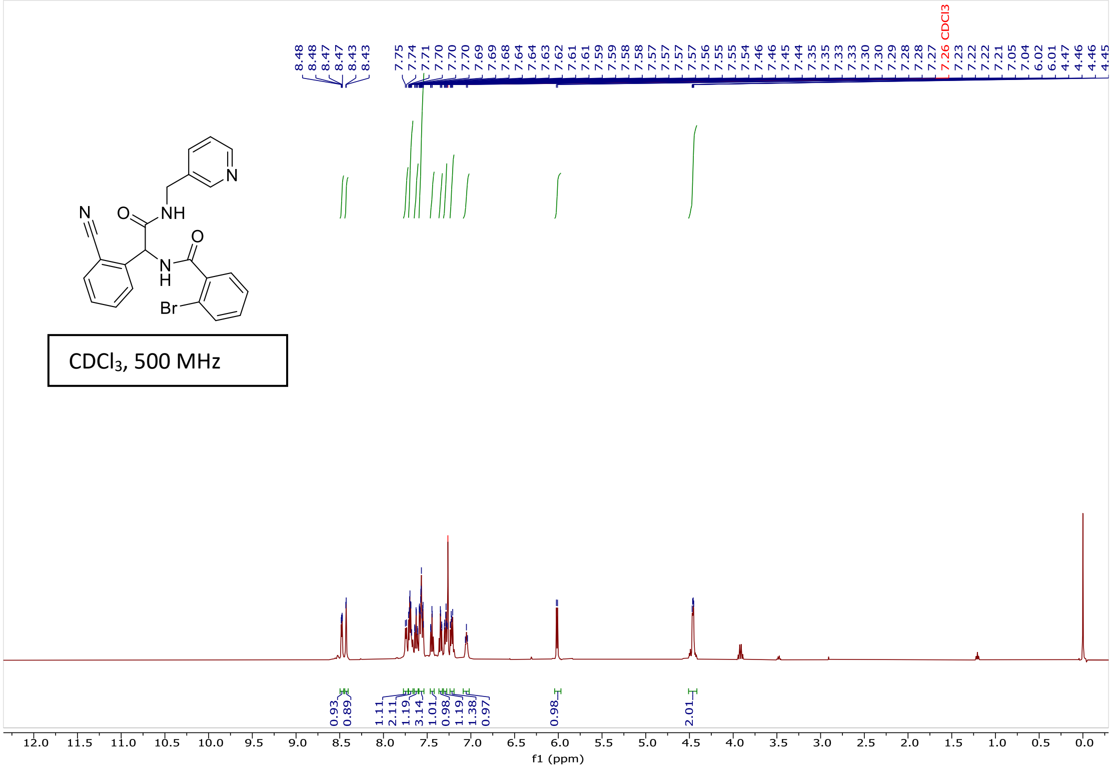

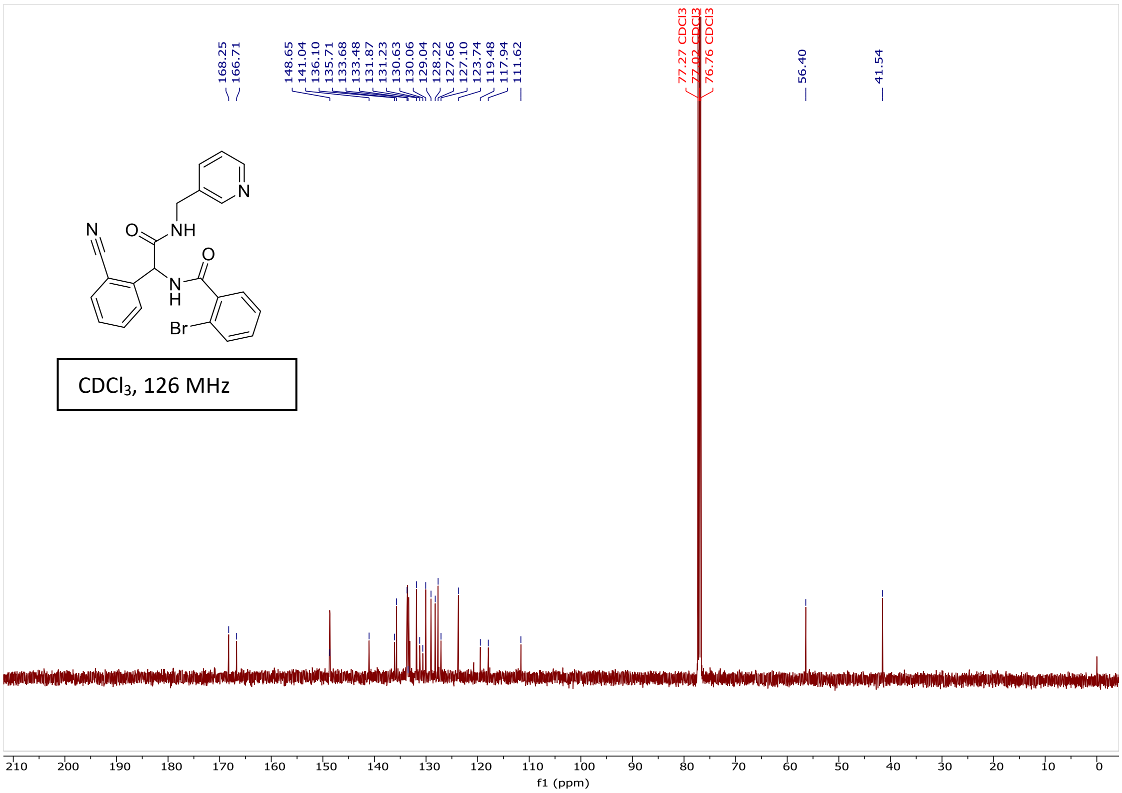

N-(2-(((3s,5s,7s)-adamantan-1-yl)amino)-1-(2-cyanophenyl)-2-oxoethyl)-2-bromobenzamide (**17a**)

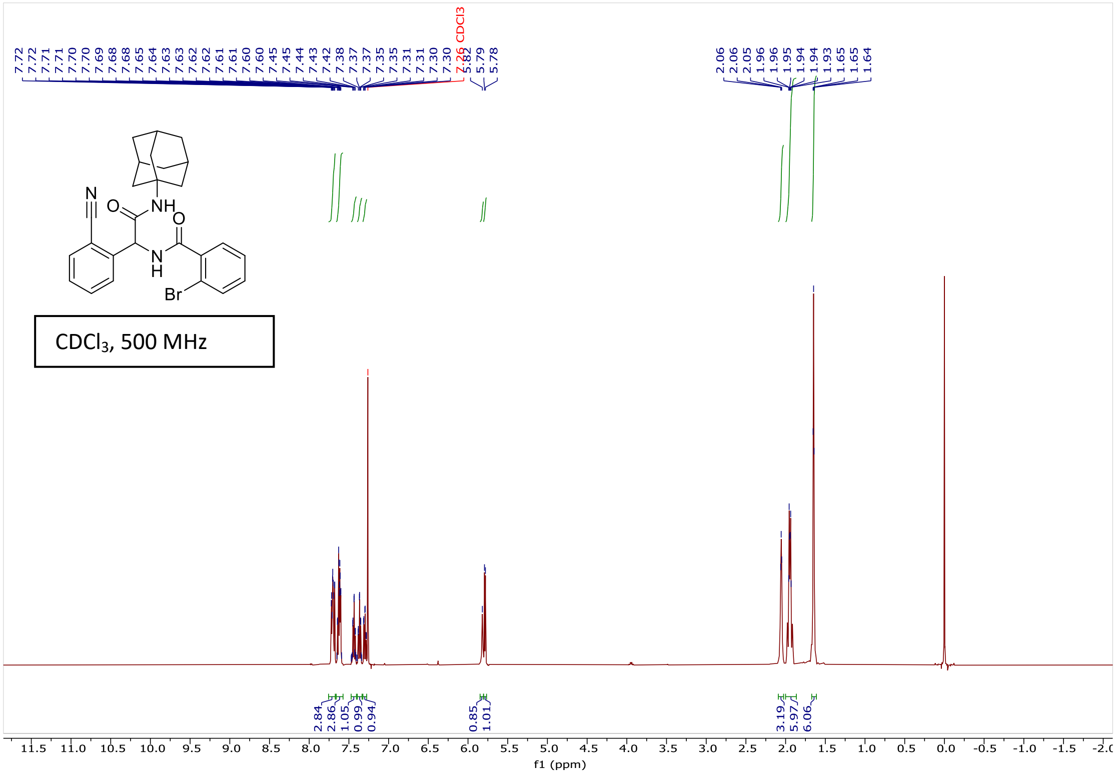

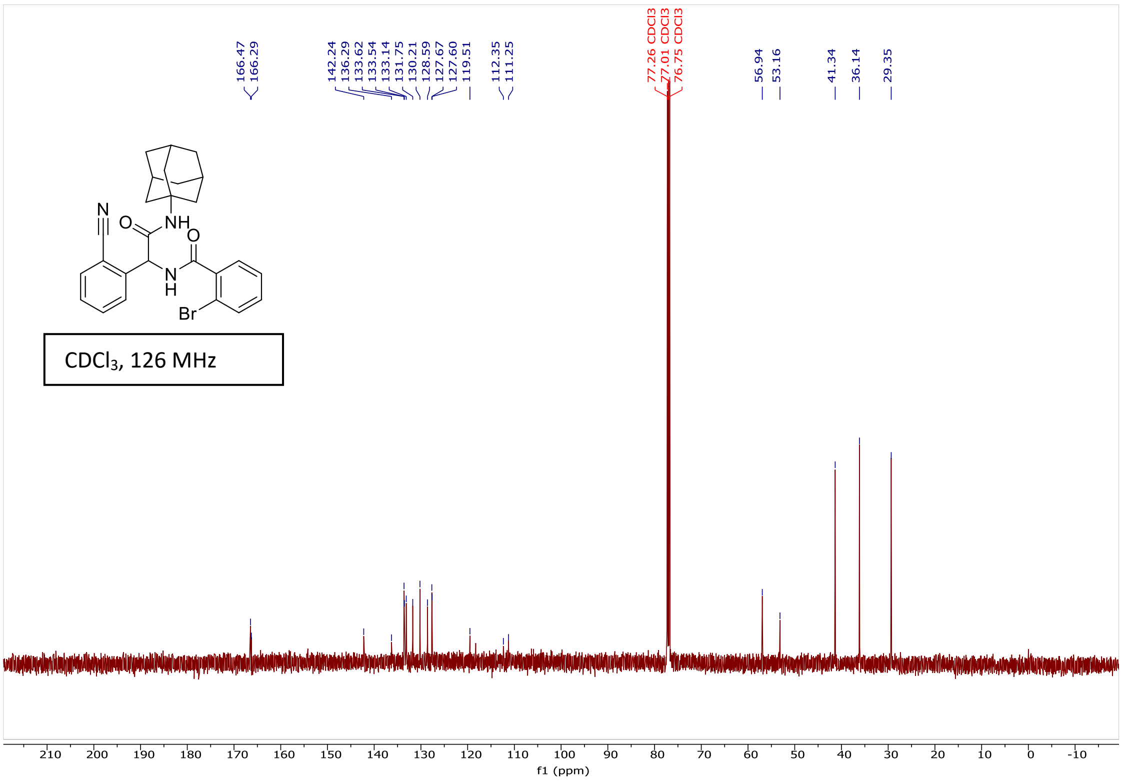

2-bromo-N-(1-(2-cyanophenyl)-2-oxo-2-(((tetrahydrofuran-2-yl)methyl)amino)ethyl) benzamide (**18a**)

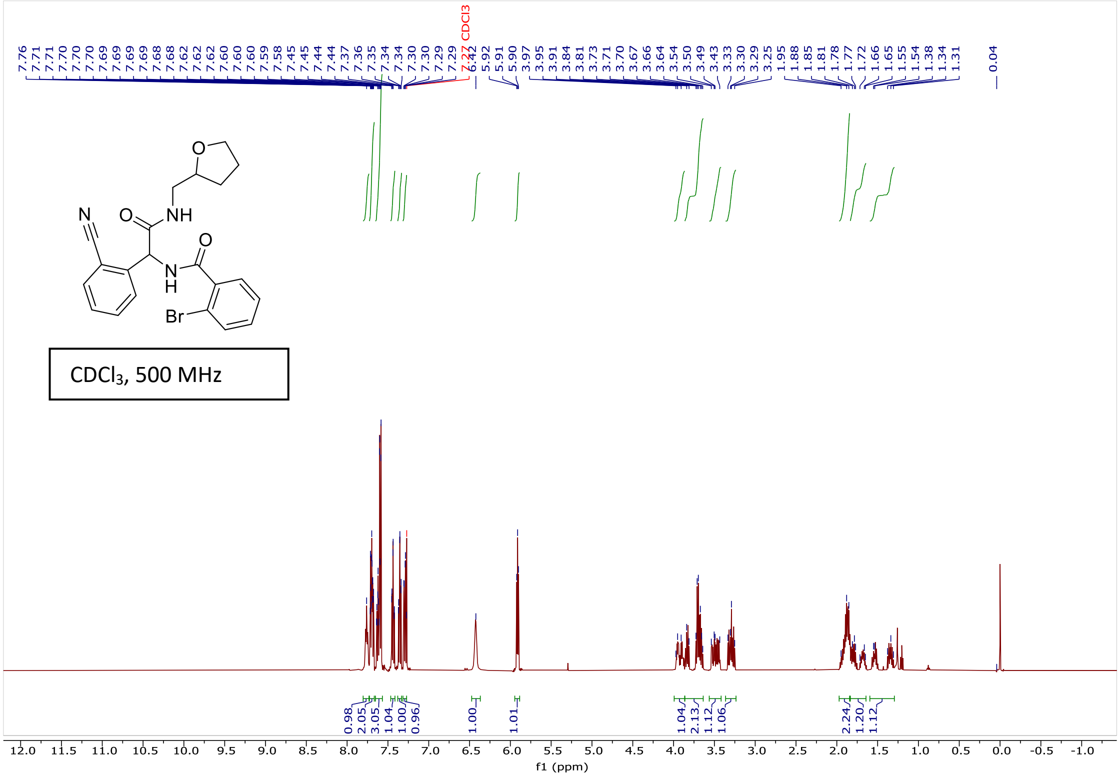

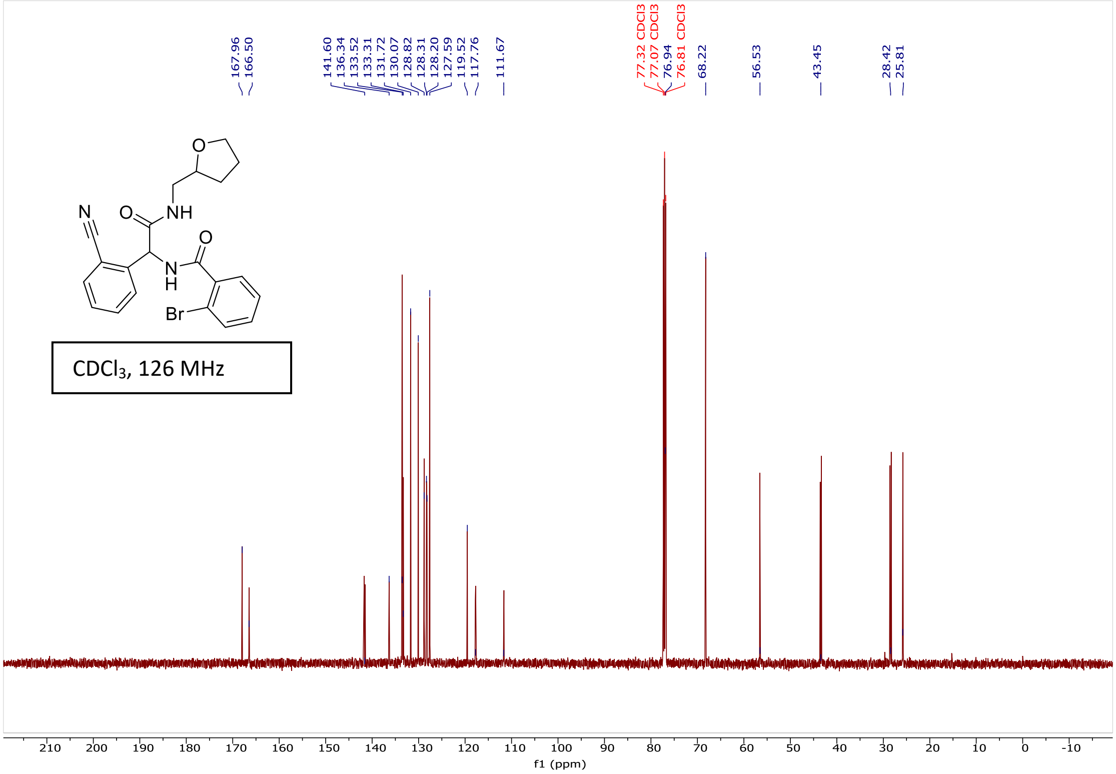

2-bromo-N-(1-(2-cyanophenyl)-2-oxo-2-((2,4,4-trimethylpentan-2-yl)amino)ethyl) benzamide (**19a**)

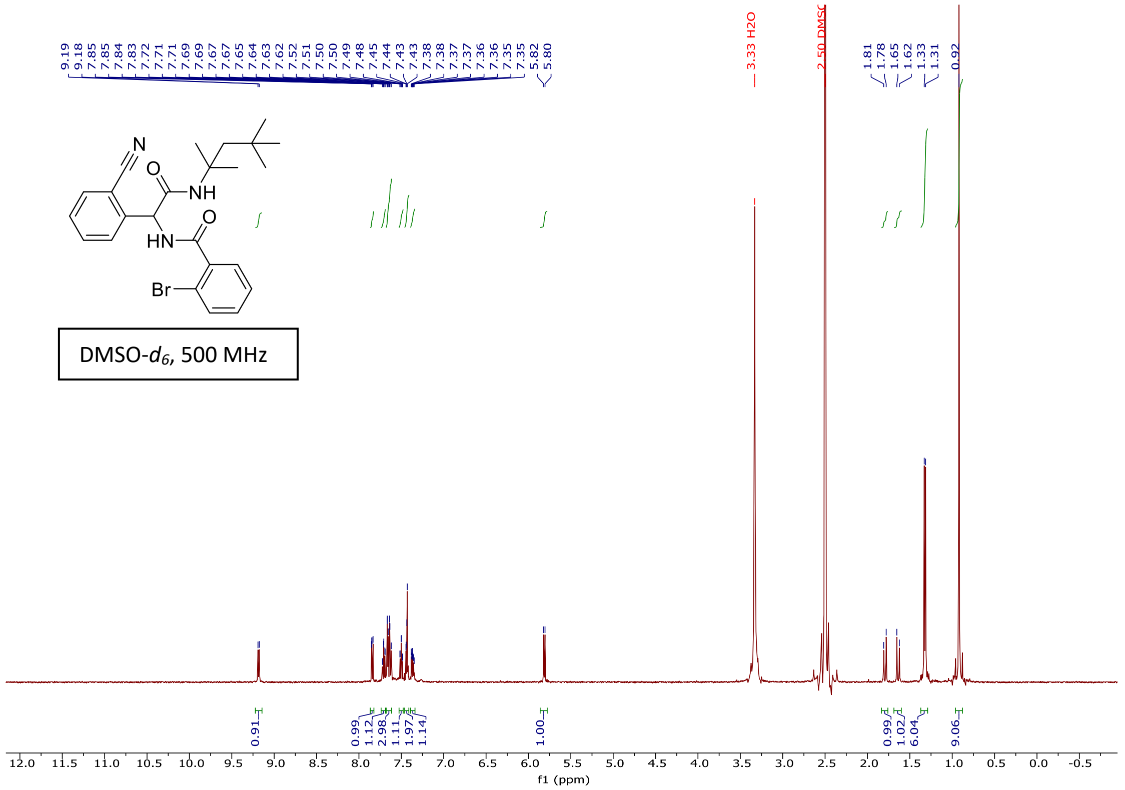

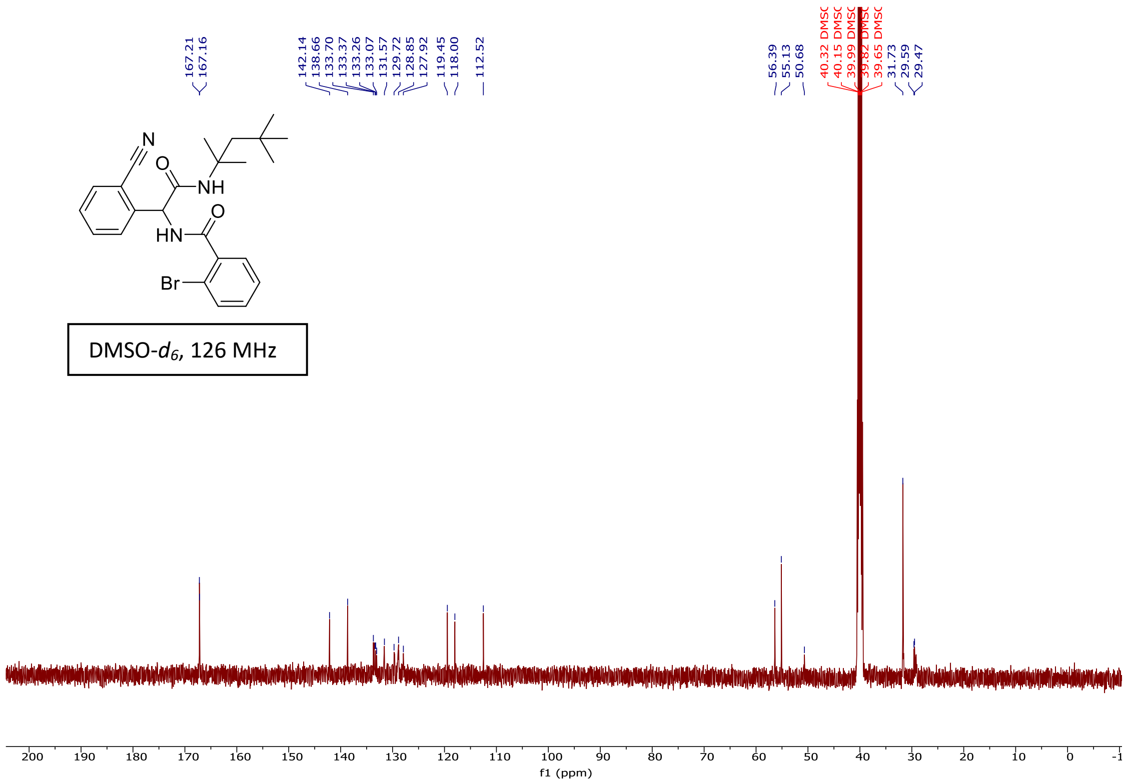

methyl (2-(2-bromobenzamido)-2-(2-cyanophenyl)acetyl)glycinate (**20a**)

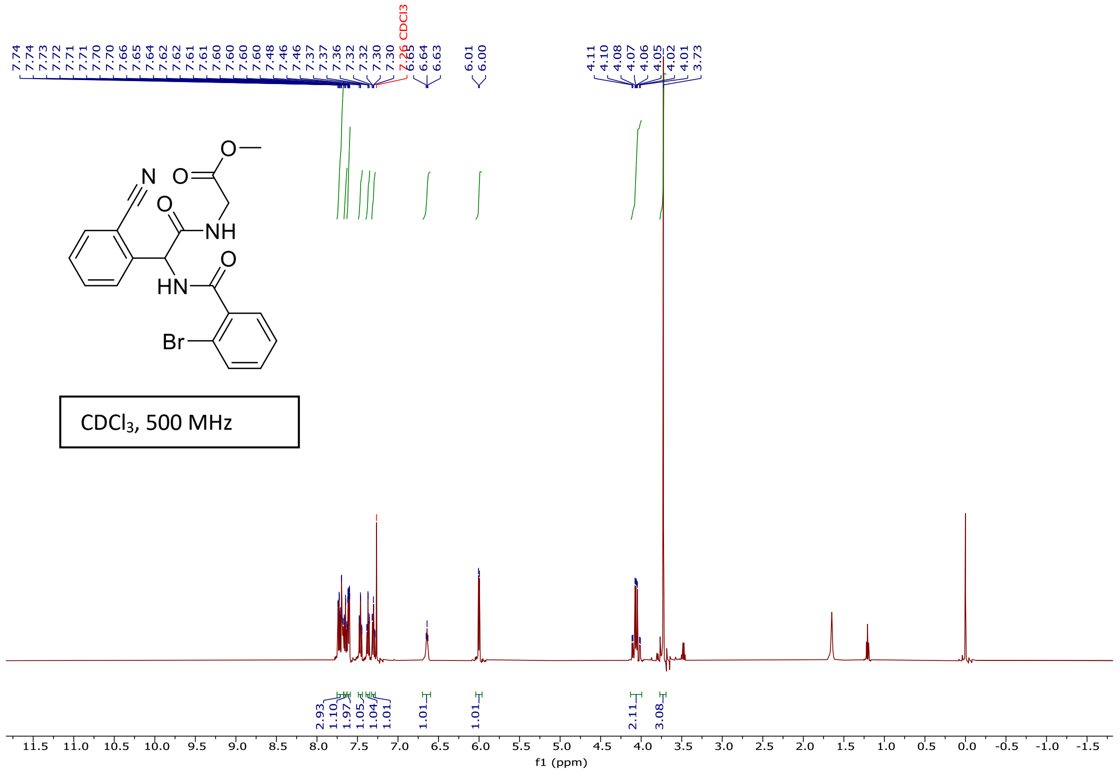

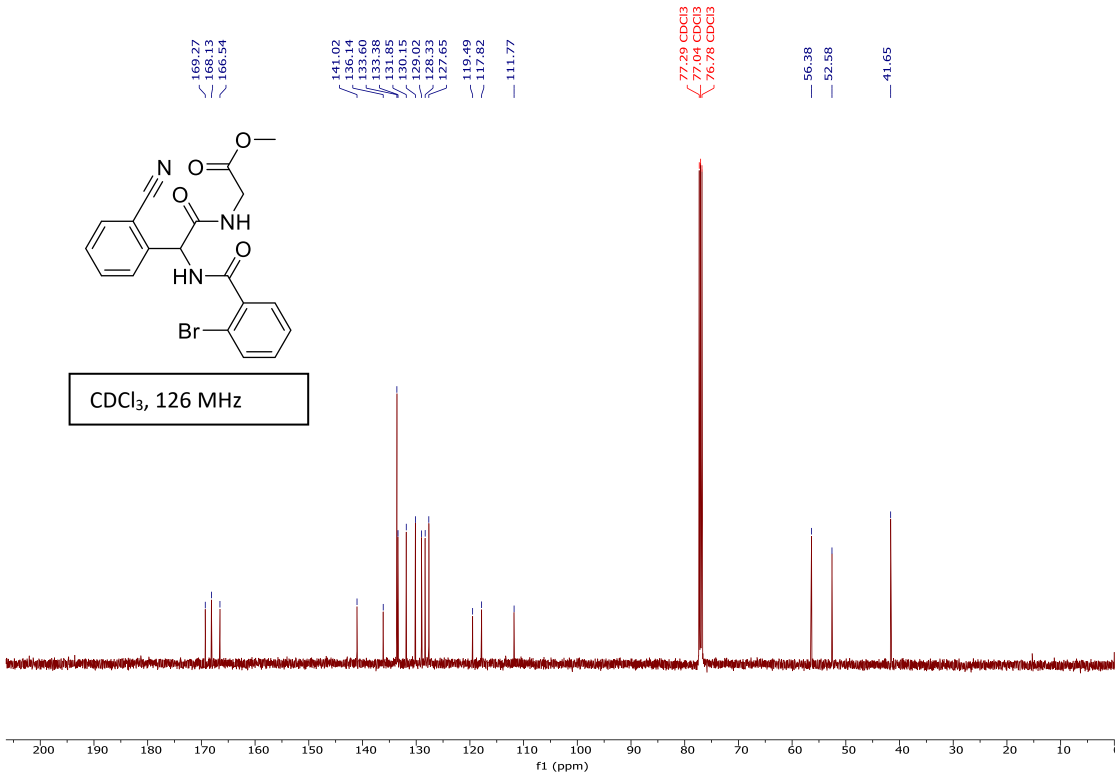

tert-butyl (2-(2-(2-bromobenzamido)-2-(2-cyanophenyl)acetamido)ethyl)carbamate (**21a**)

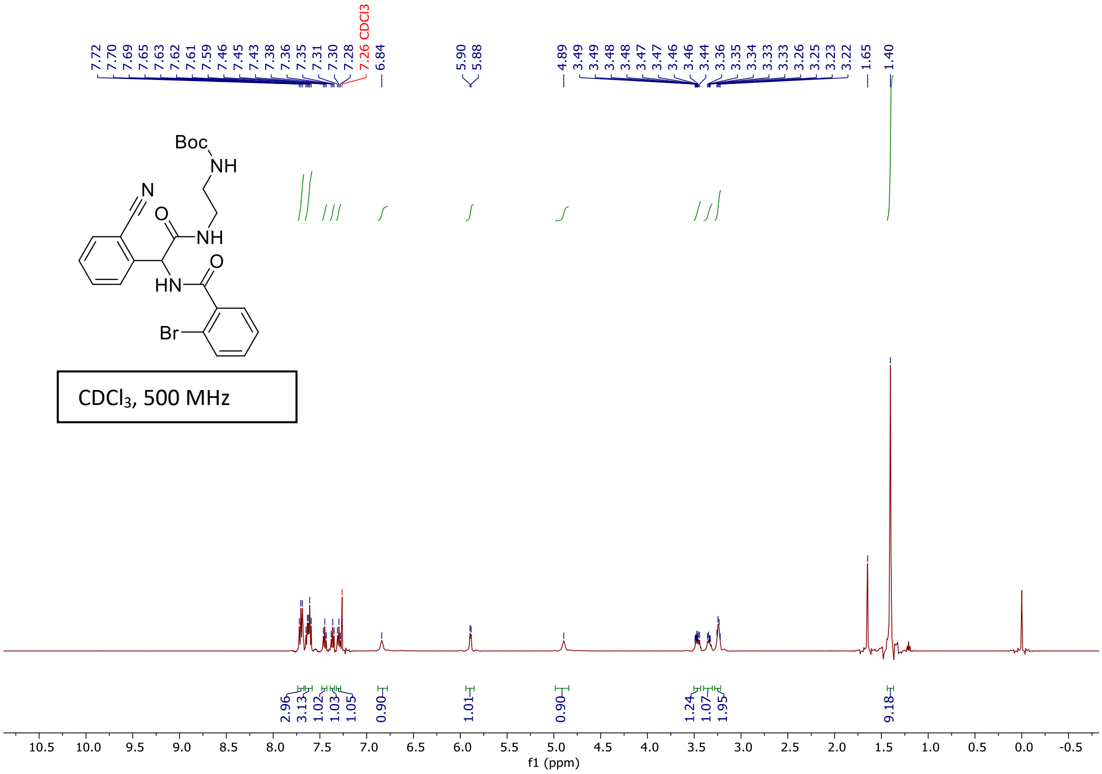

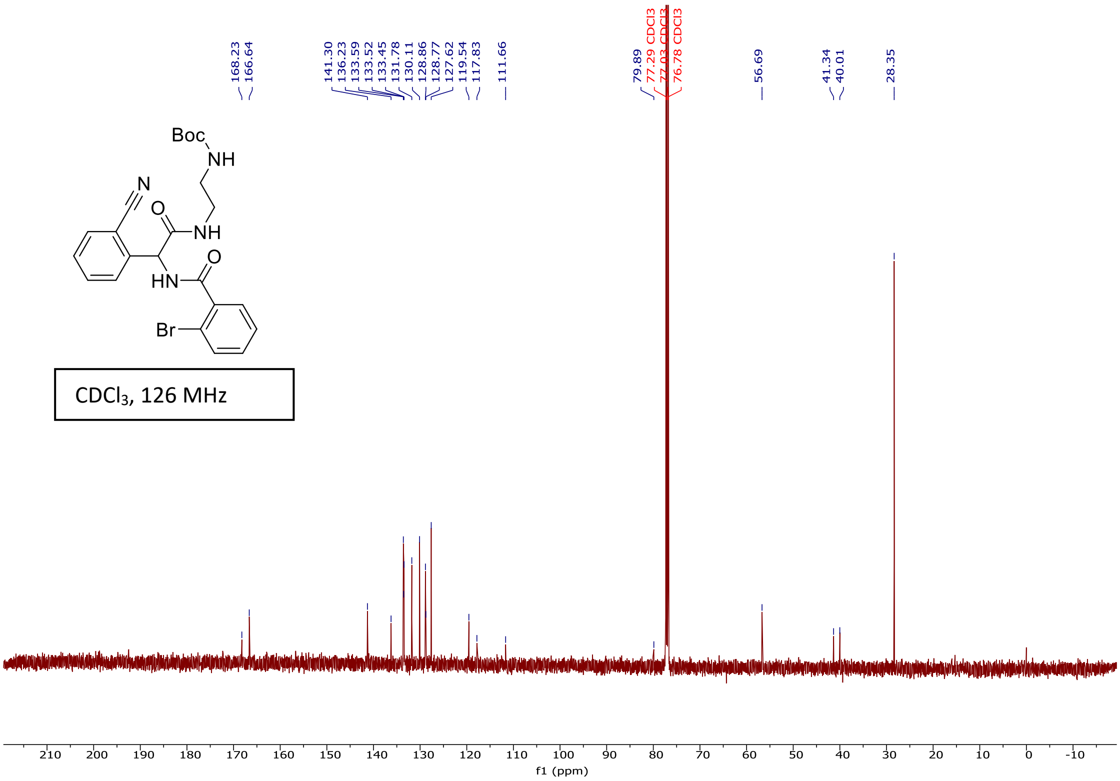

2-bromo-N-(2-((3-cyanobenzyl)amino)-1-(2-cyanophenyl)-2-oxoethyl)benzamide (**22a**)

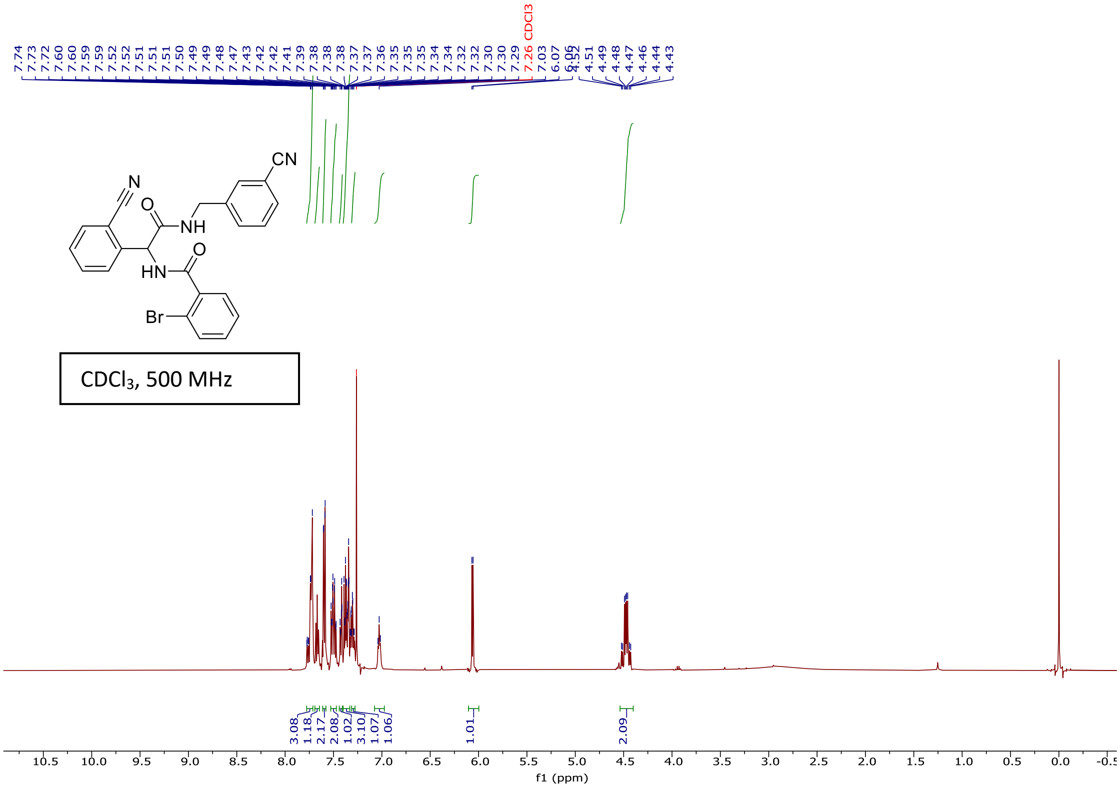

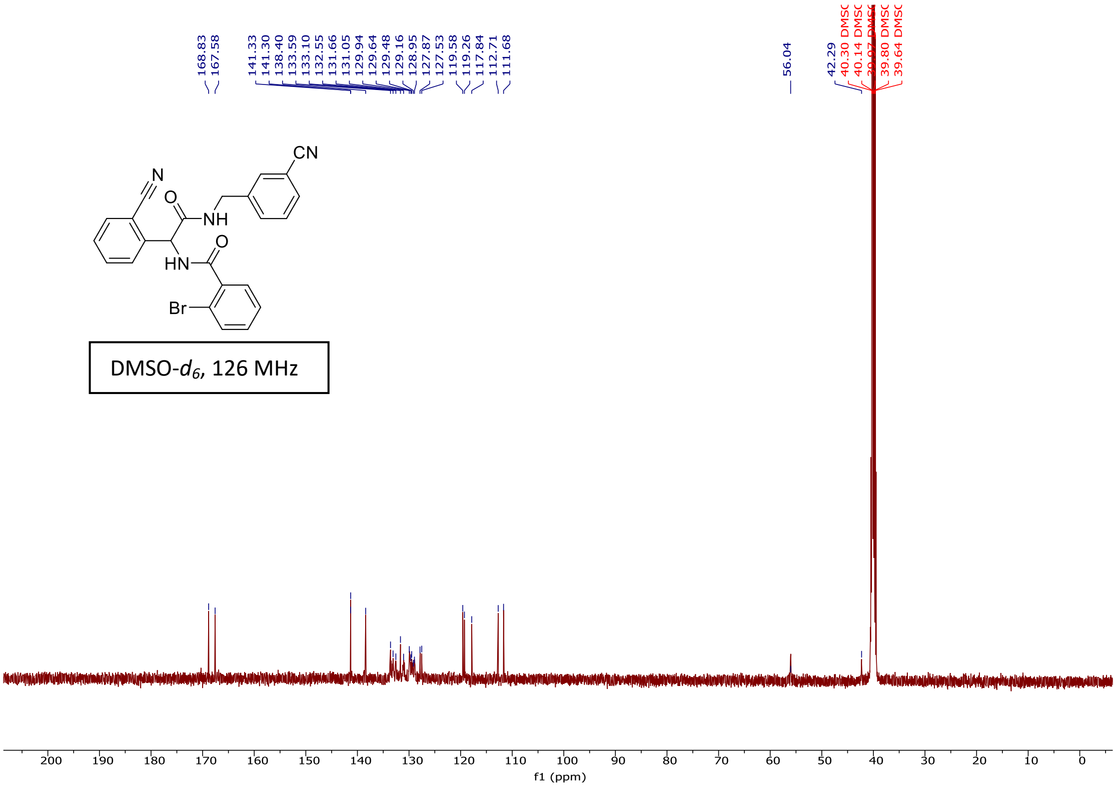

2-bromo-N-(2-((2-chloro-3,6-difluorobenzyl)amino)-1-(2-cyanophenyl)-2-oxoethyl) benzamide (**23a**)

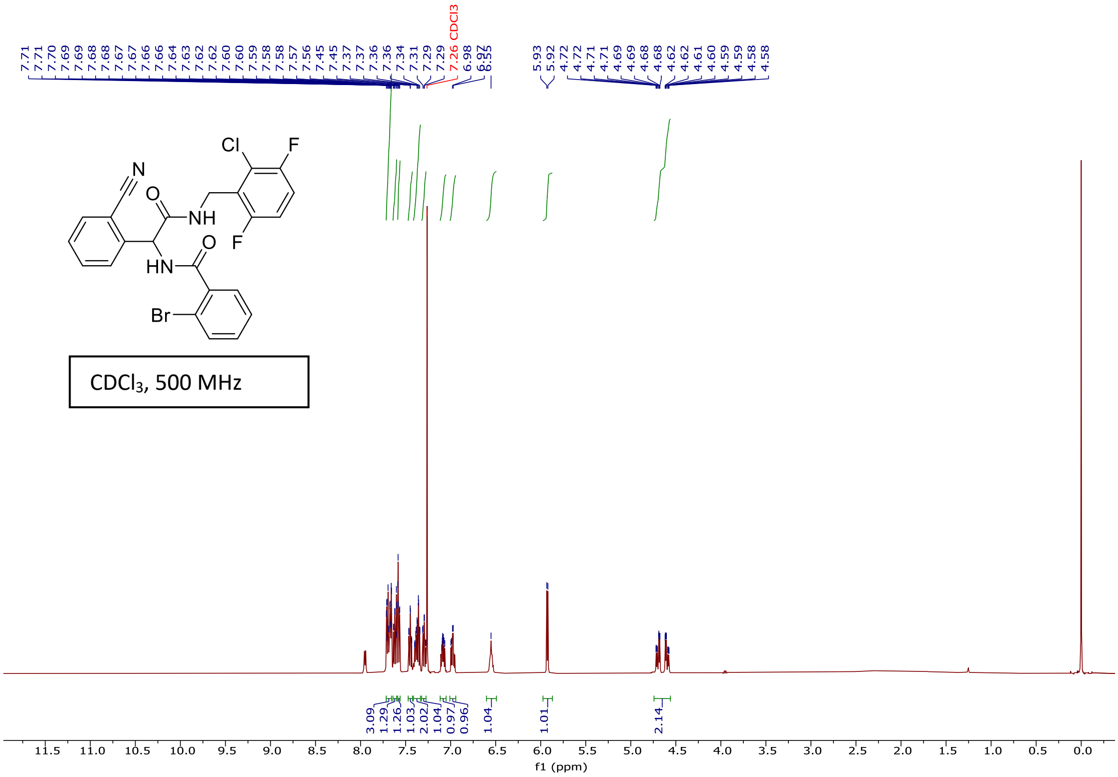

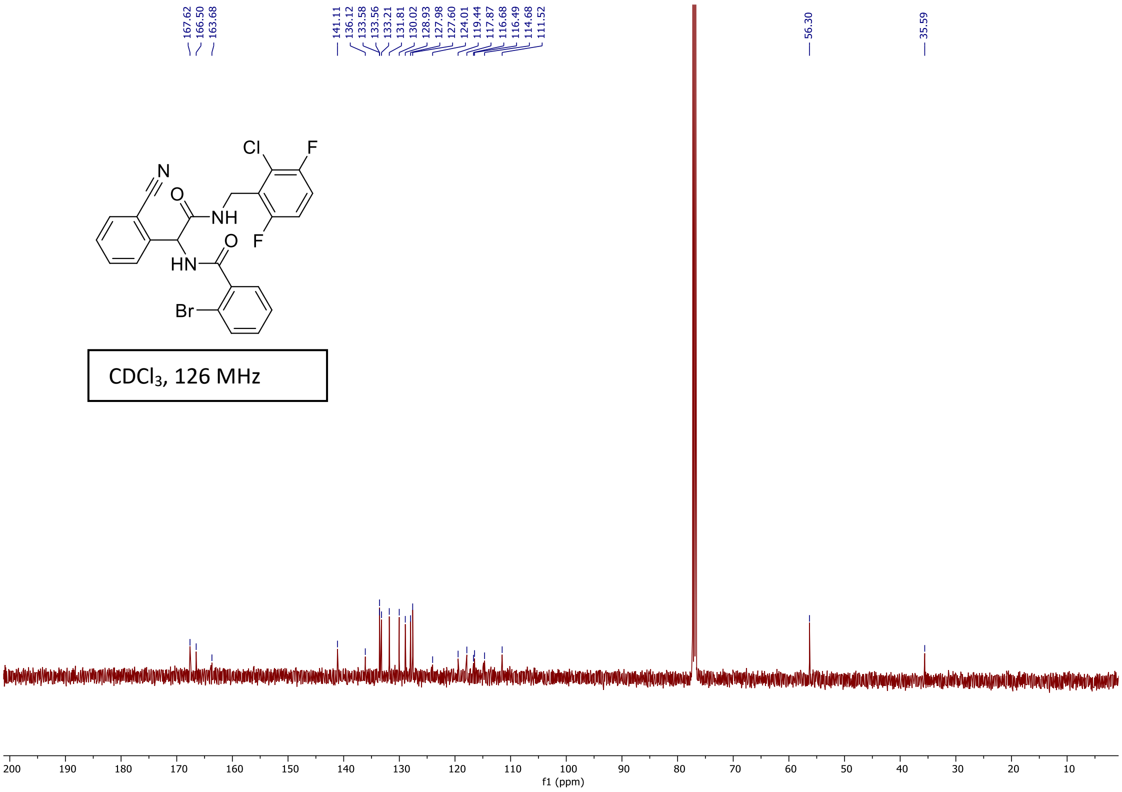

2-bromo-N-(1-(2-cyanophenyl)-2-oxo-2-((2-(trifluoromethoxy)benzyl)amino)ethyl) benzamide (**24a**)

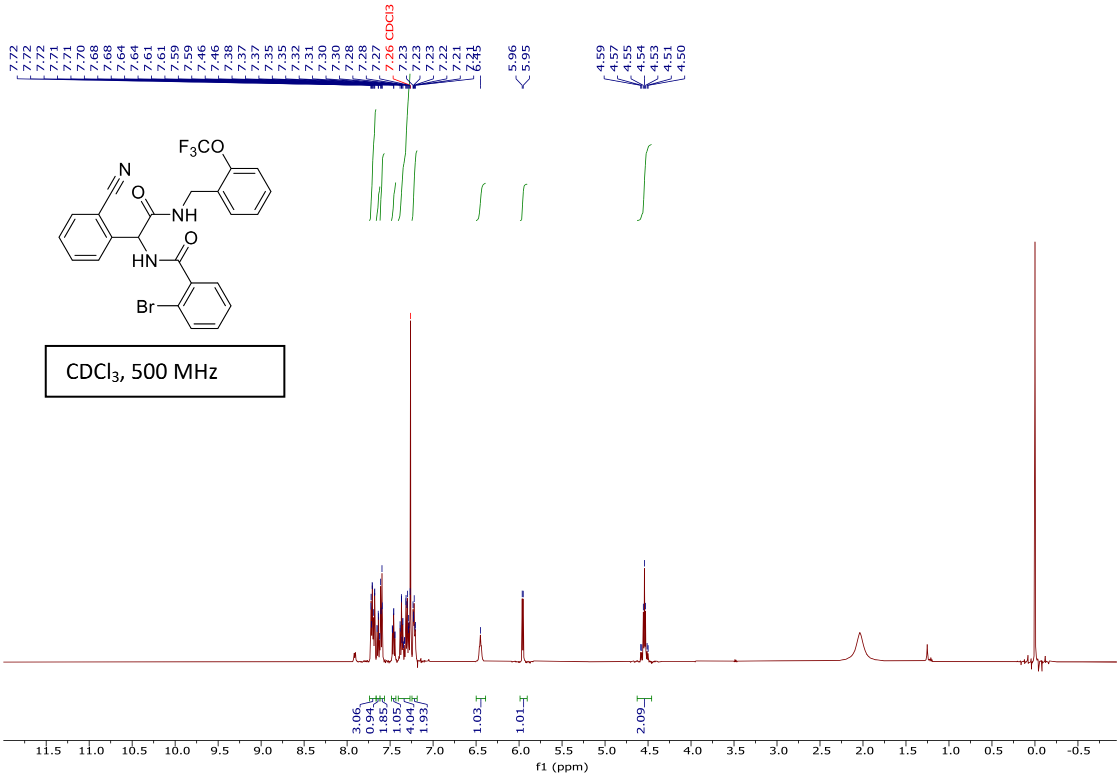

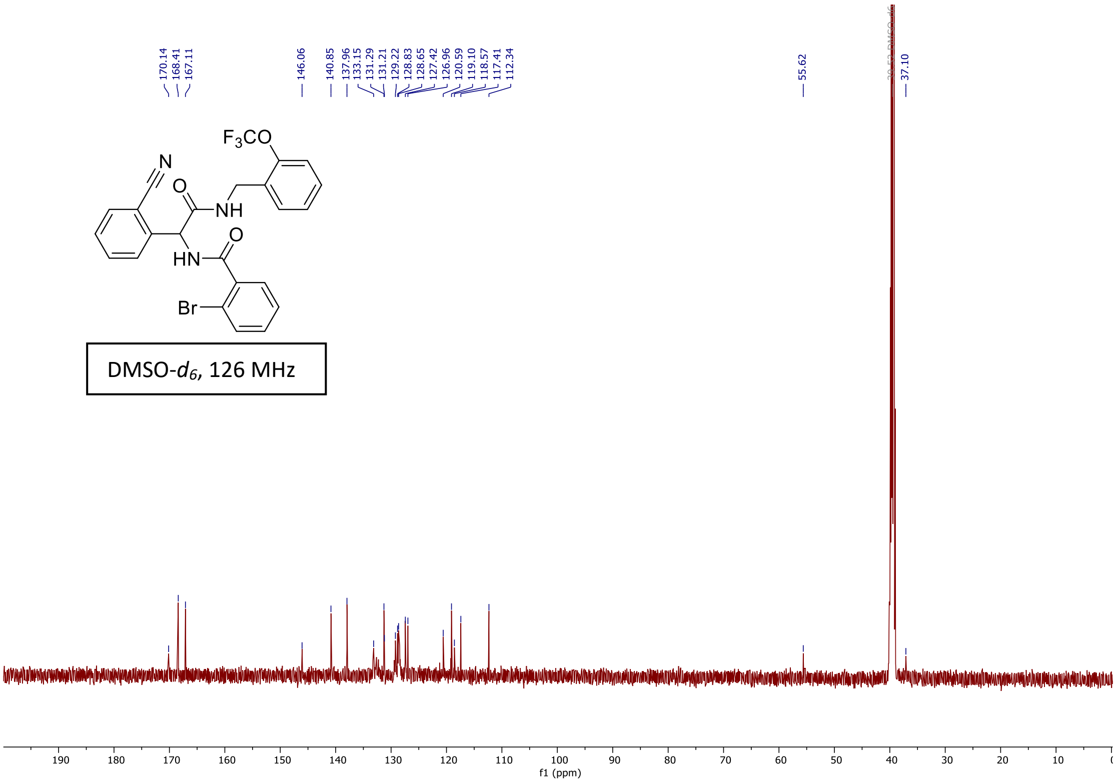

N-(2-(benzylamino)-1-(2-cyanophenyl)-2-oxoethyl)-2-bromo-4-methylbenzamide (**25a**)

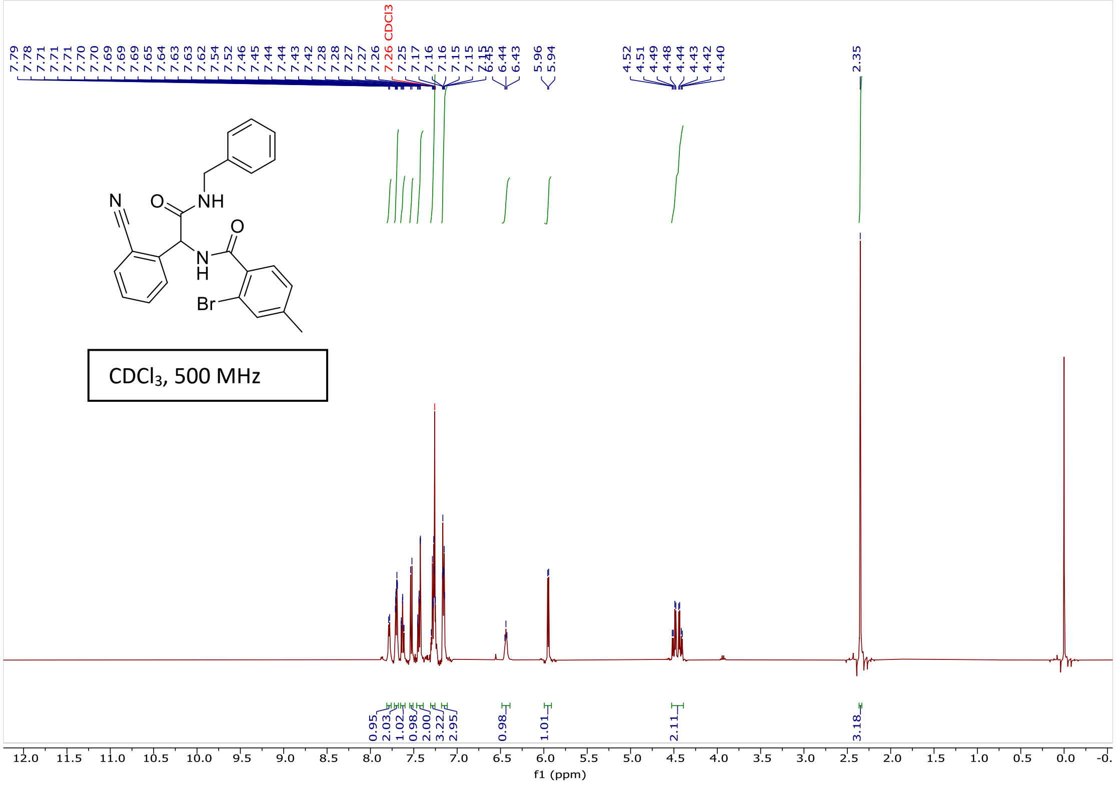

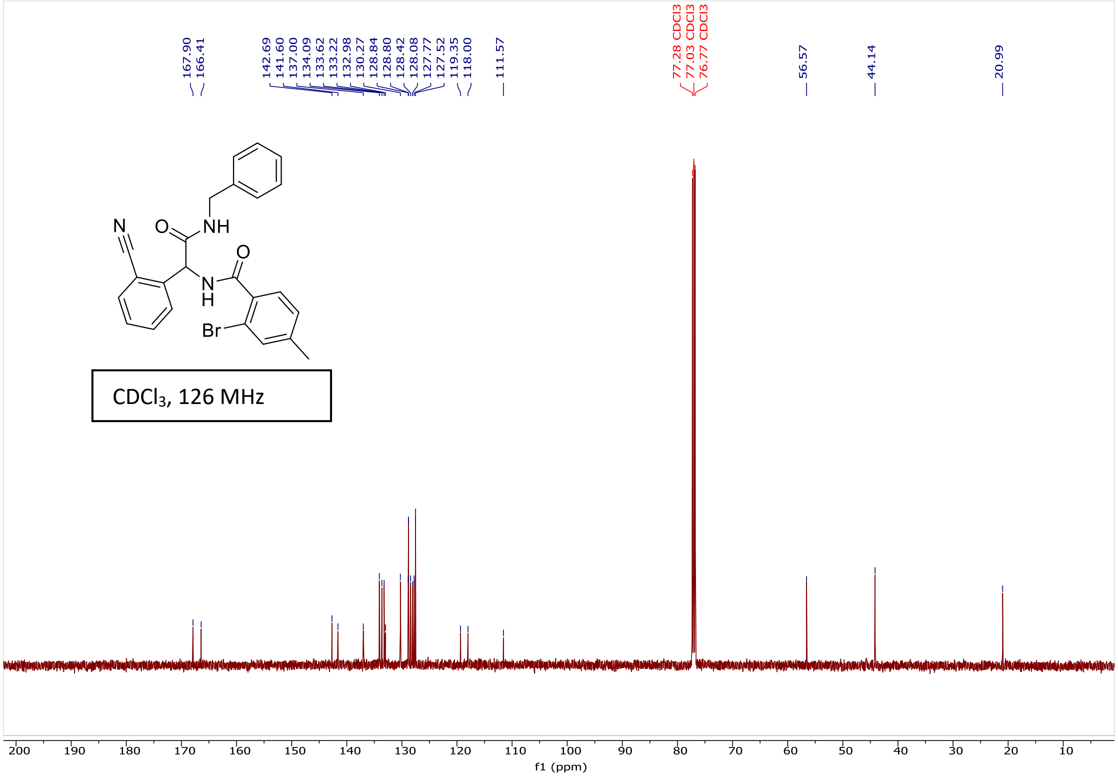

N-(2-(benzylamino)-1-(2-cyanophenyl)-2-oxoethyl)-2-bromothiophene-3-carboxamide (**26a**)

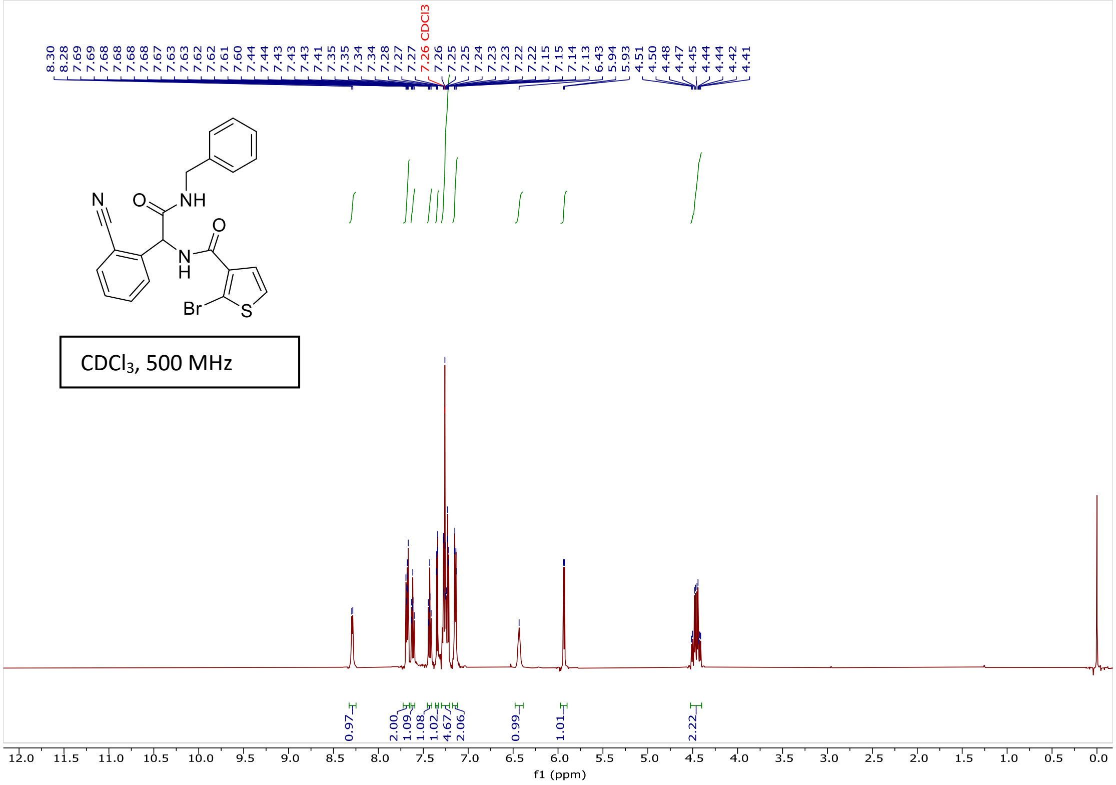

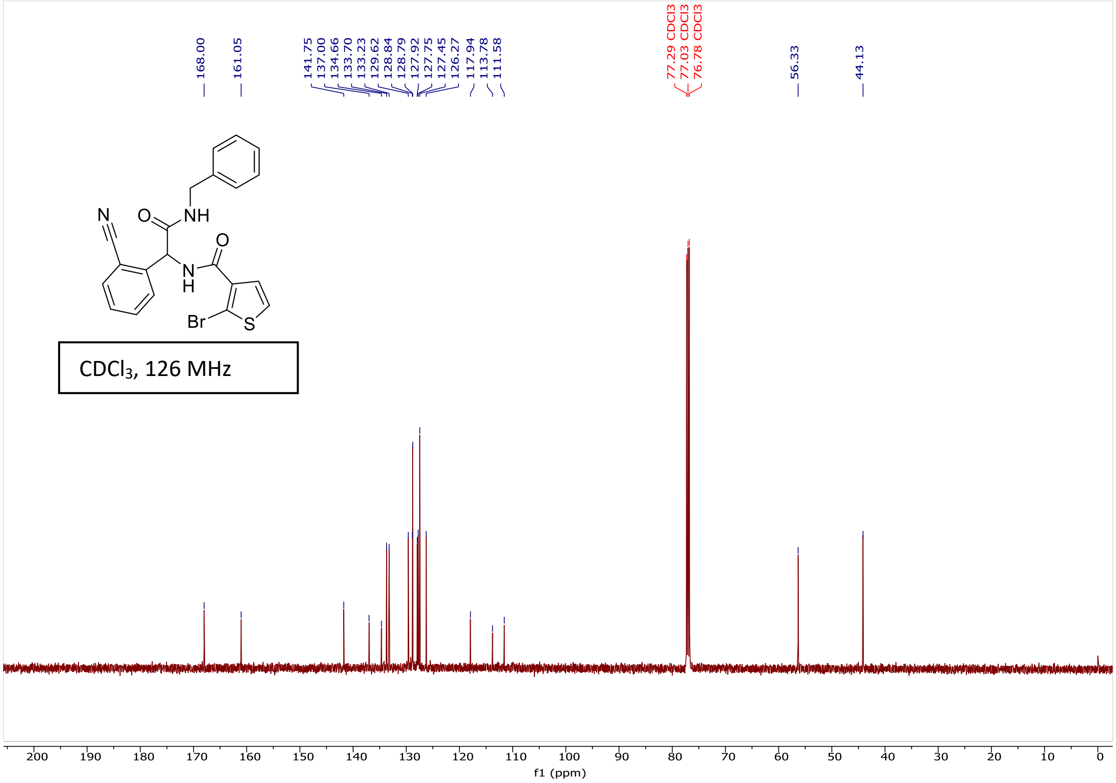

N-(2-(benzylamino)-1-(2-cyanophenyl)-2-oxoethyl)-4-bromo-2-iodobenzamide (**27a**)

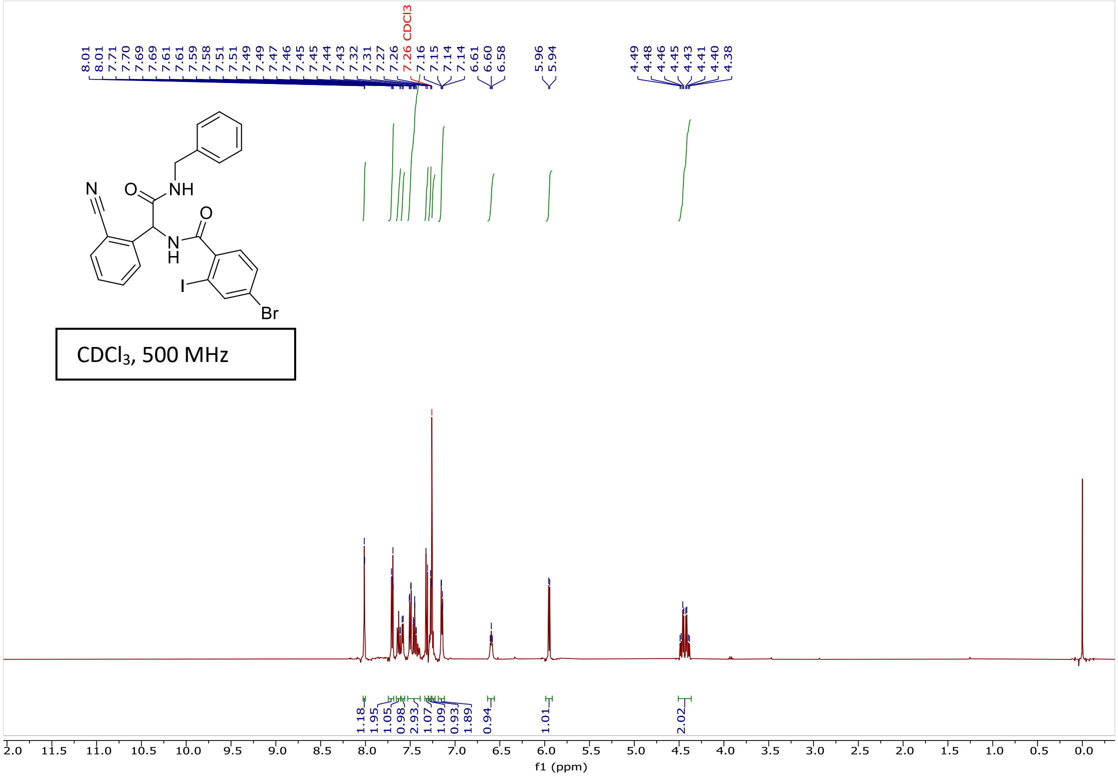

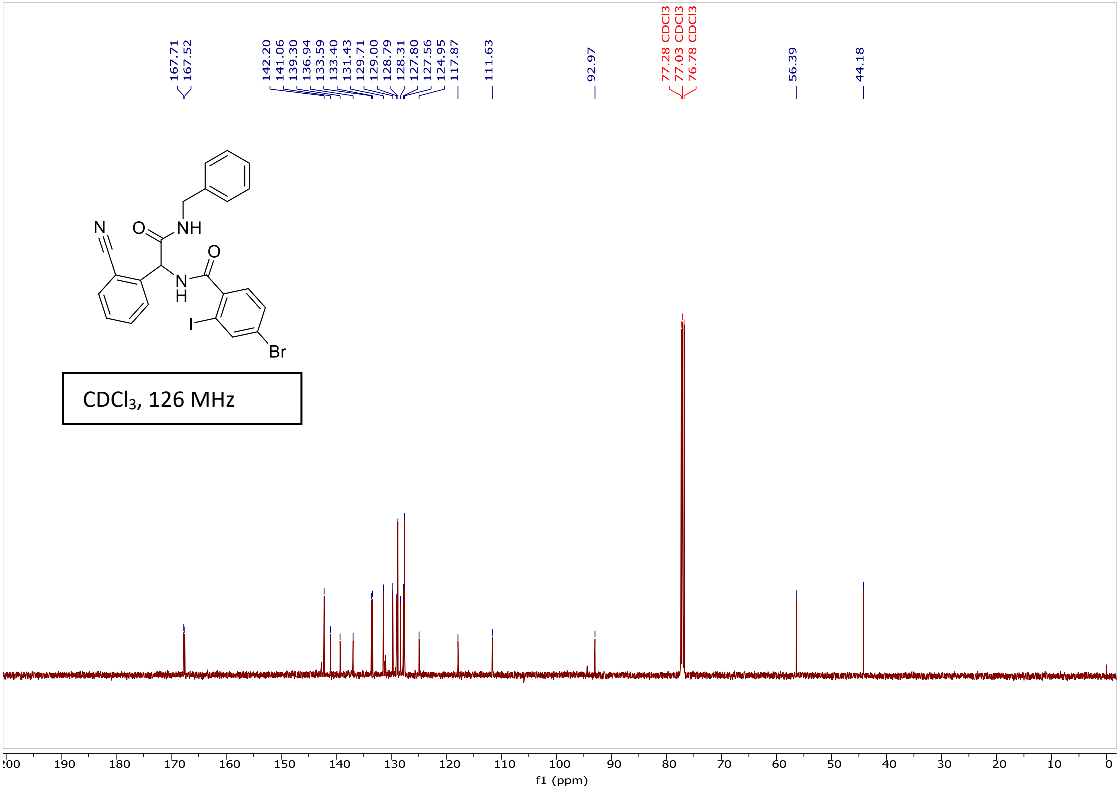

N-(2-(benzylamino)-1-(2-cyanophenyl)-2-oxoethyl)-2-bromo-5-methylbenzamide (**28a**)

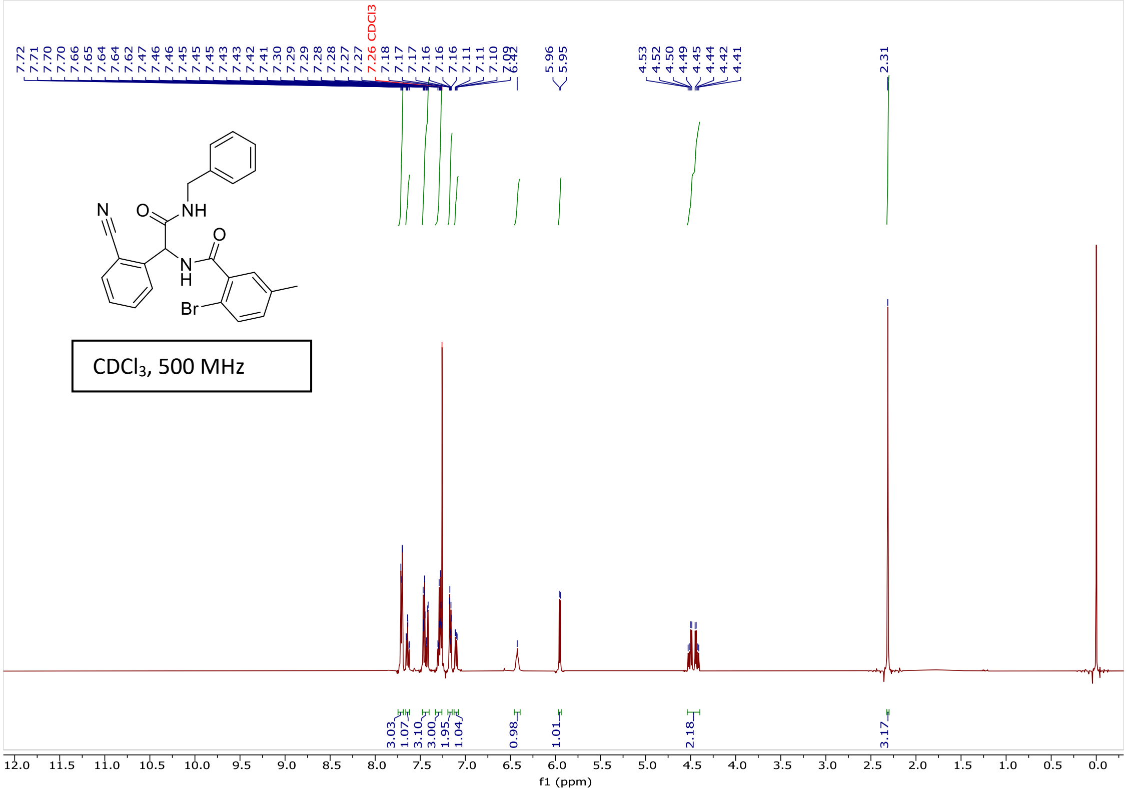

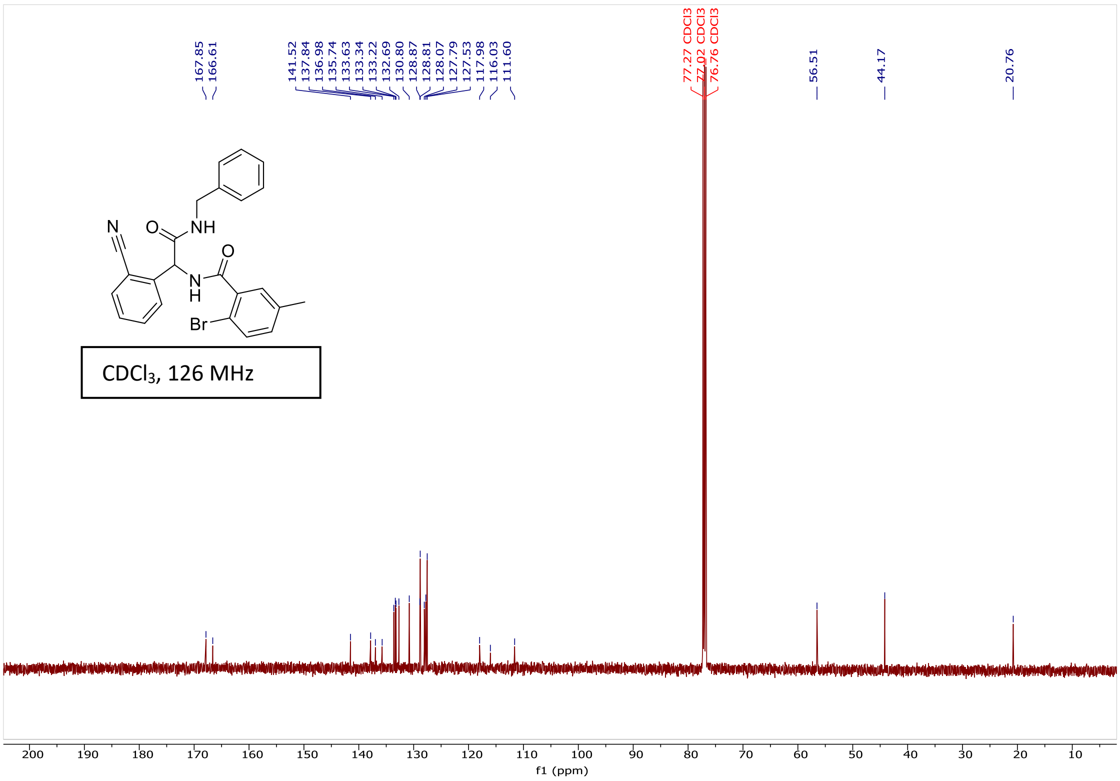

N-(2-(benzylamino)-1-(2-cyanophenyl)-2-oxoethyl)-4-bromonicotinamide (**29a**)

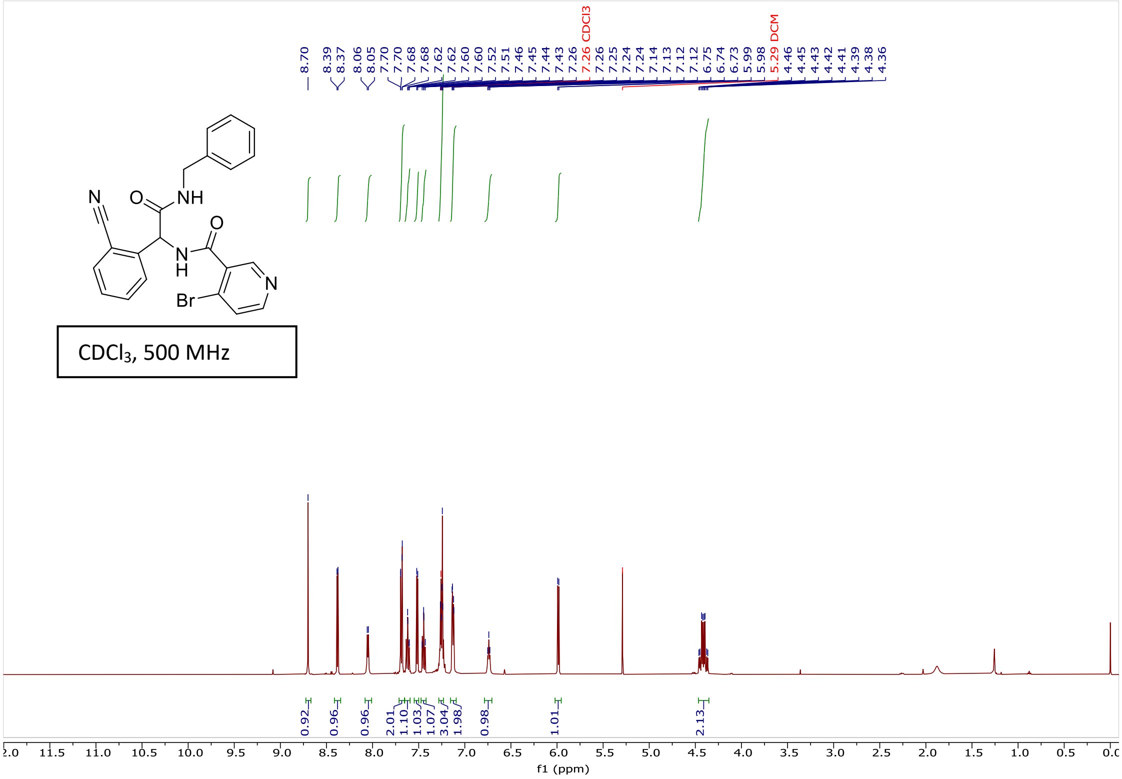

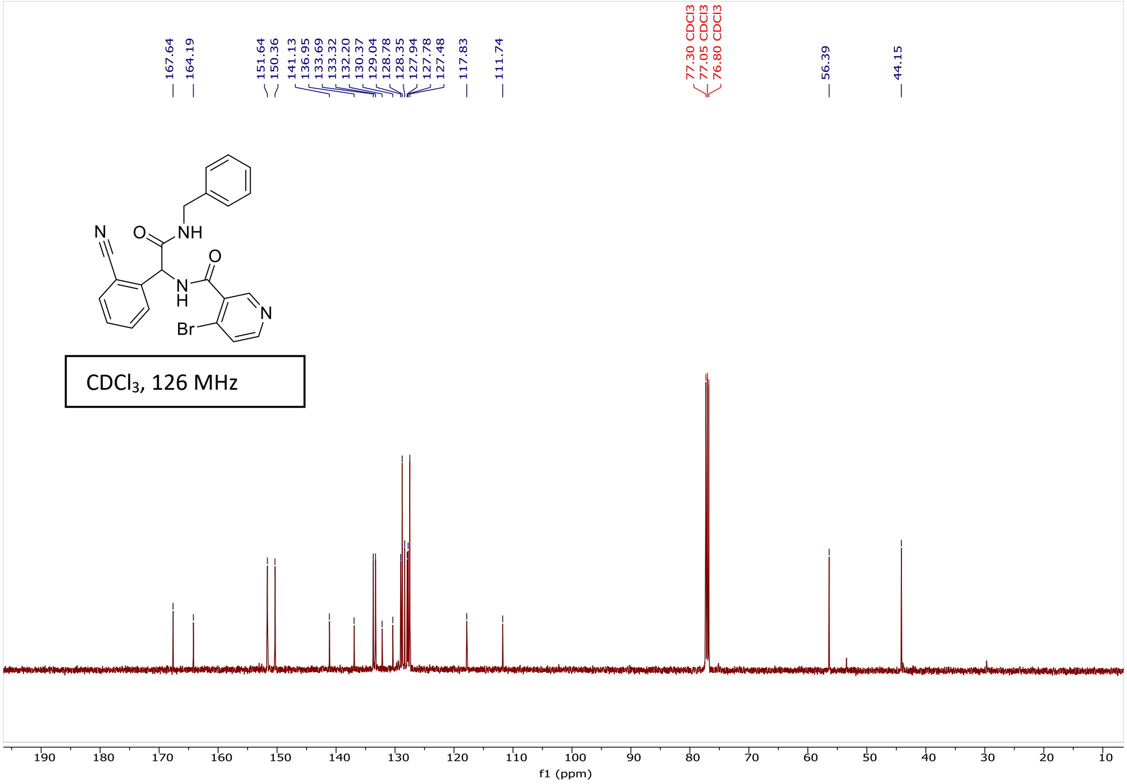

N-(2-(benzylamino)-1-(2-cyanophenyl)-2-oxoethyl)-1-bromo-2-naphthamide (**30a**)

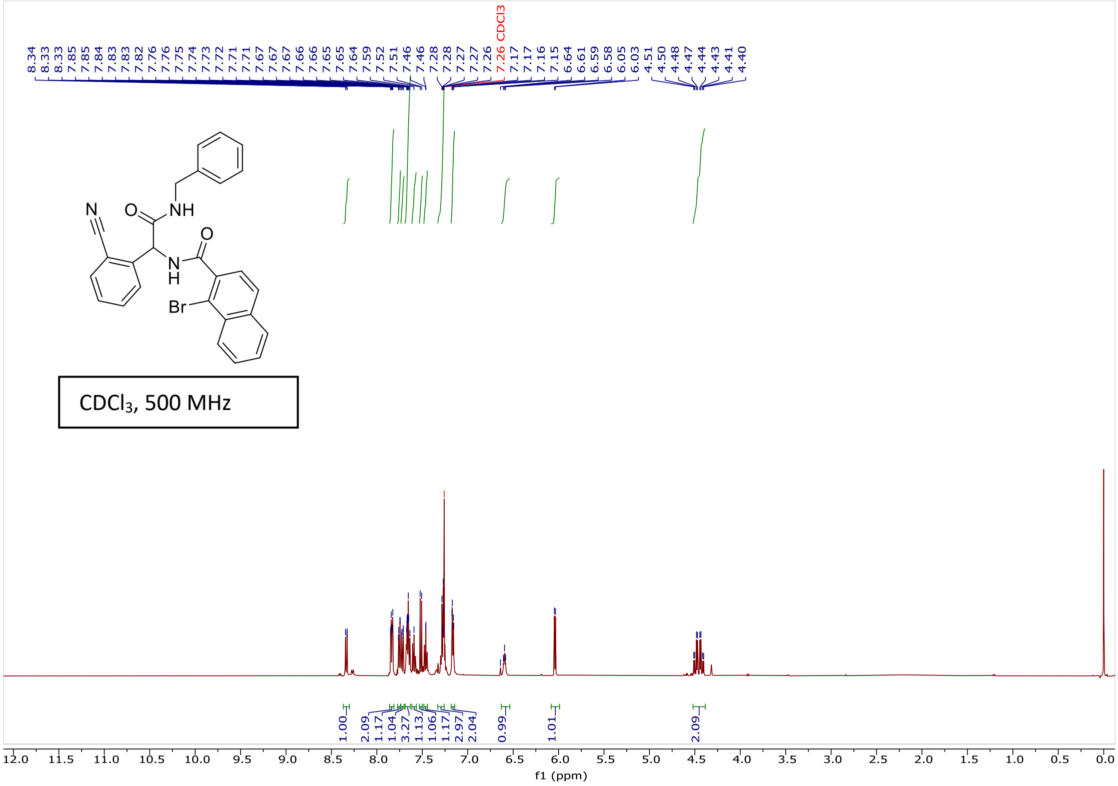

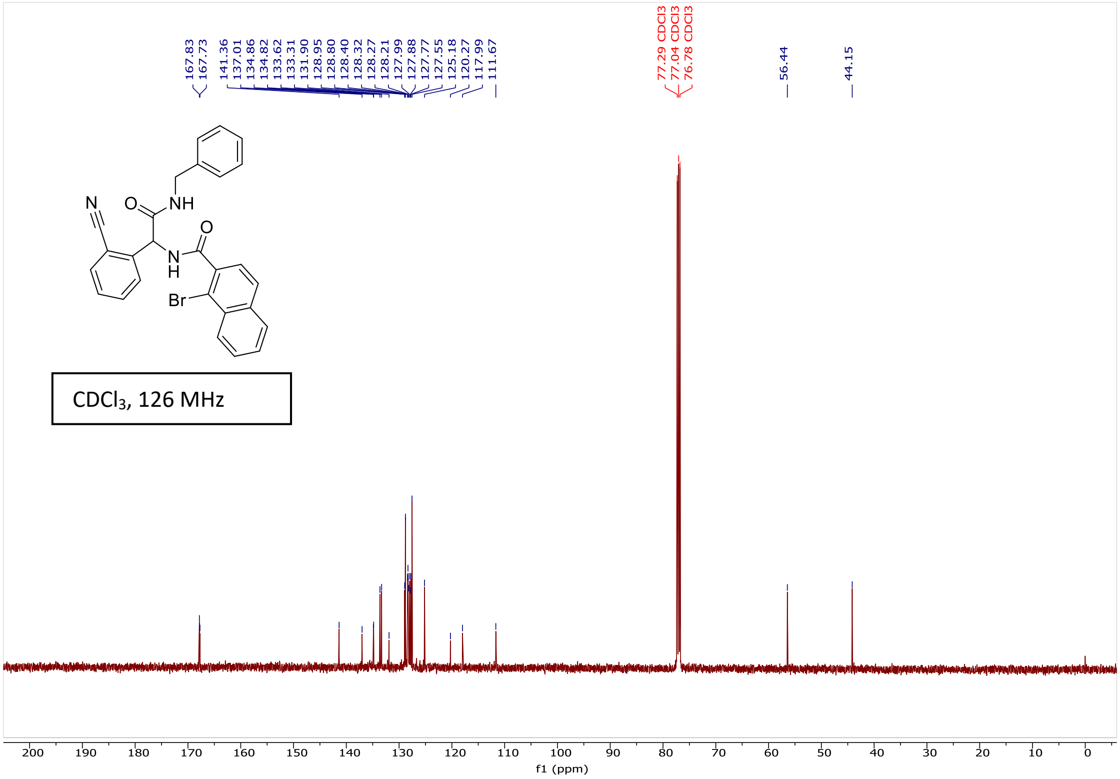

N-(2-(benzylamino)-1-(2-cyanophenyl)-2-oxoethyl)-3-bromoisonicotinamide (**31a**)

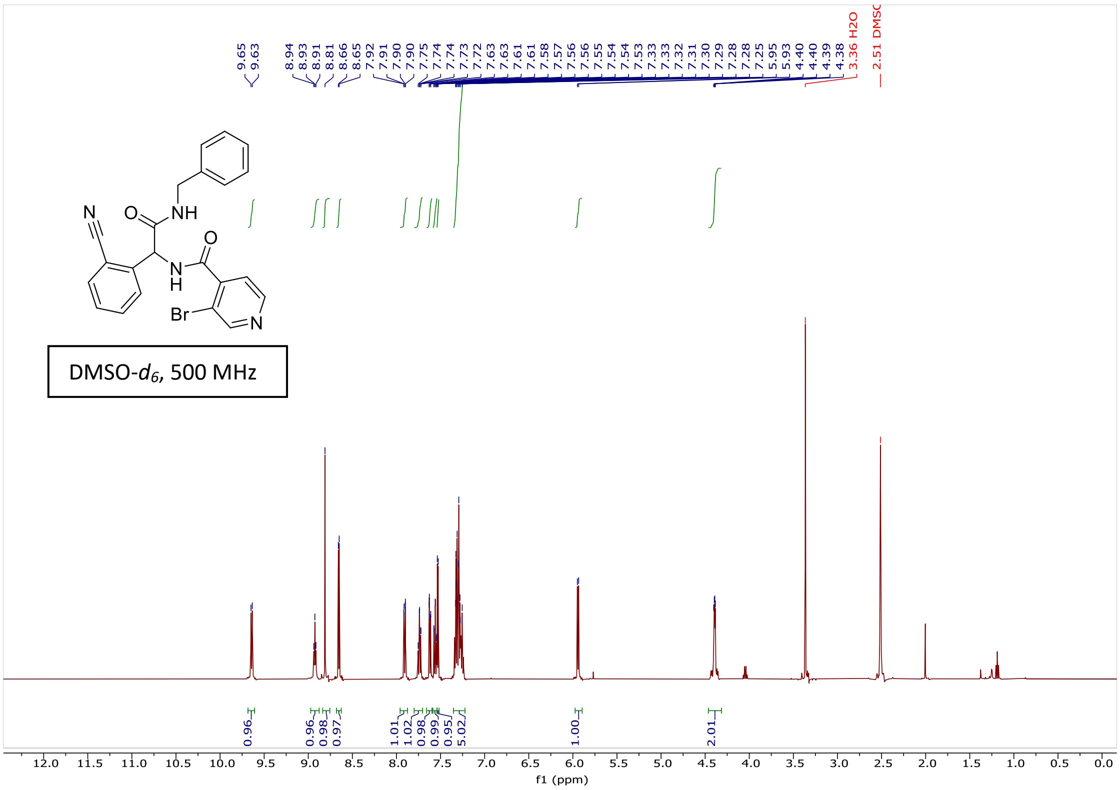

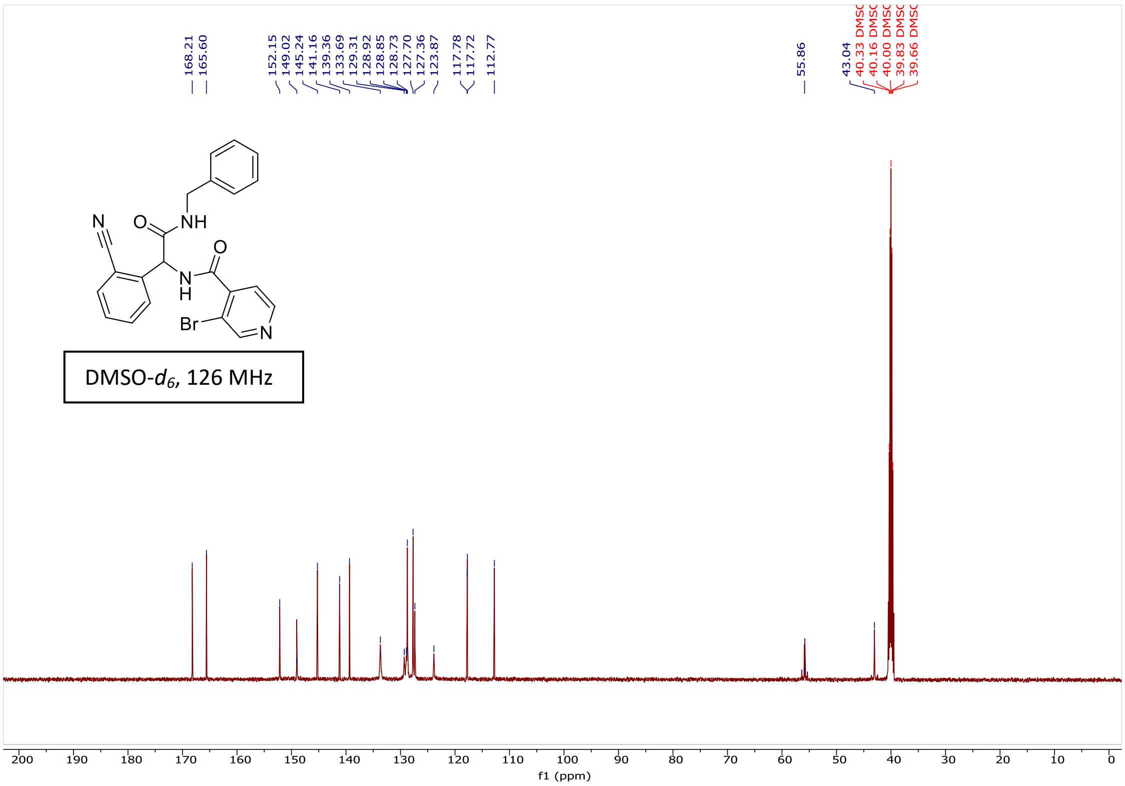

N-(2-(benzylamino)-1-(2-cyano-4-methoxyphenyl)-2-oxoethyl)-2-bromobenzamide (**32a**)

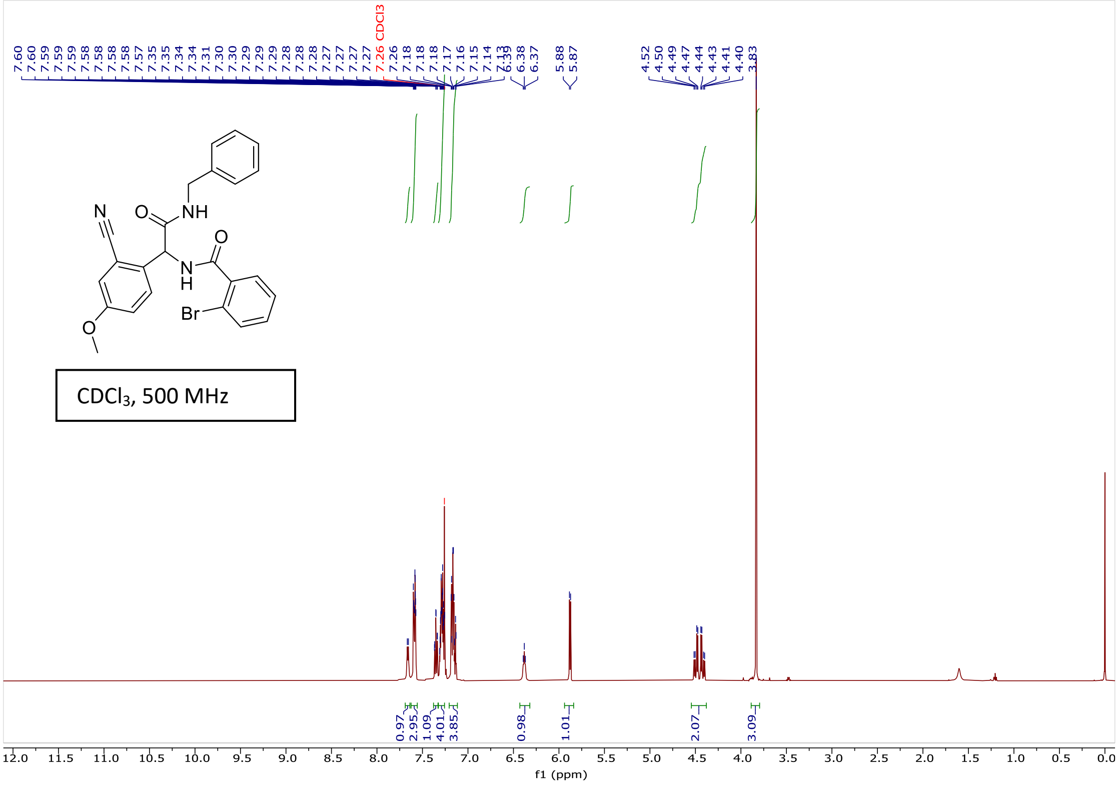

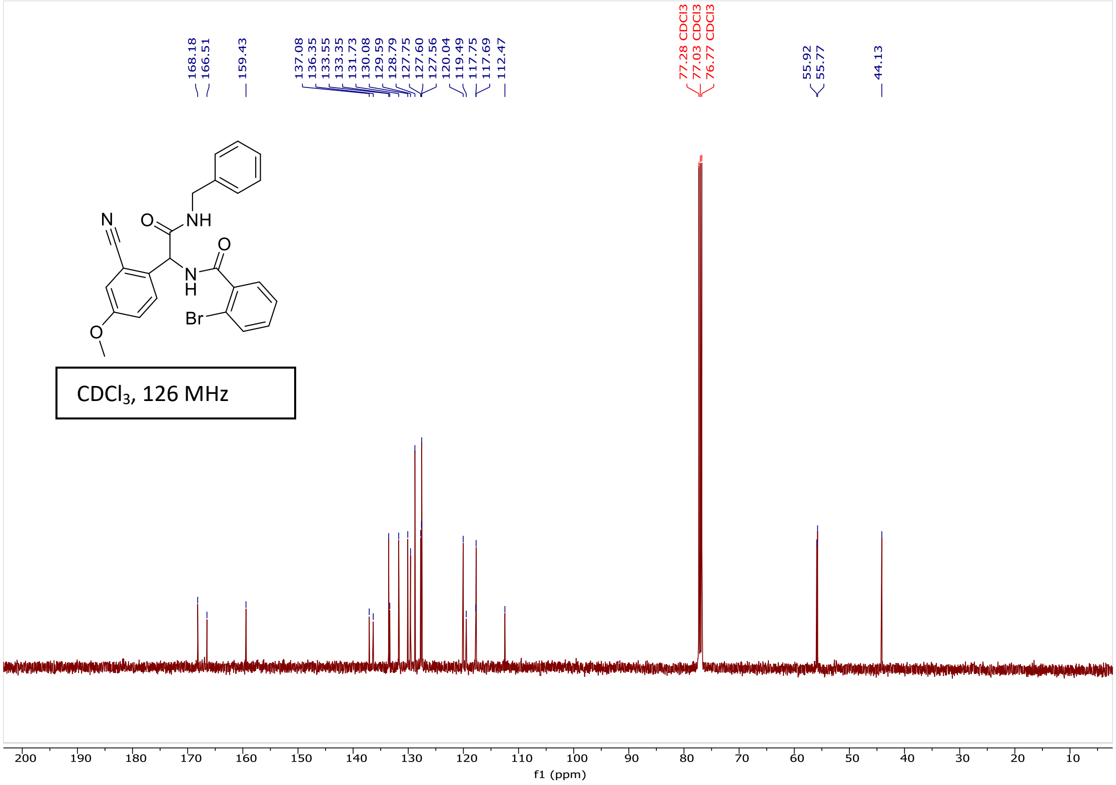

N-(2-(benzylamino)-1-(2-cyano-4-fluorophenyl)-2-oxoethyl)-2-bromobenzamide (**33a**)

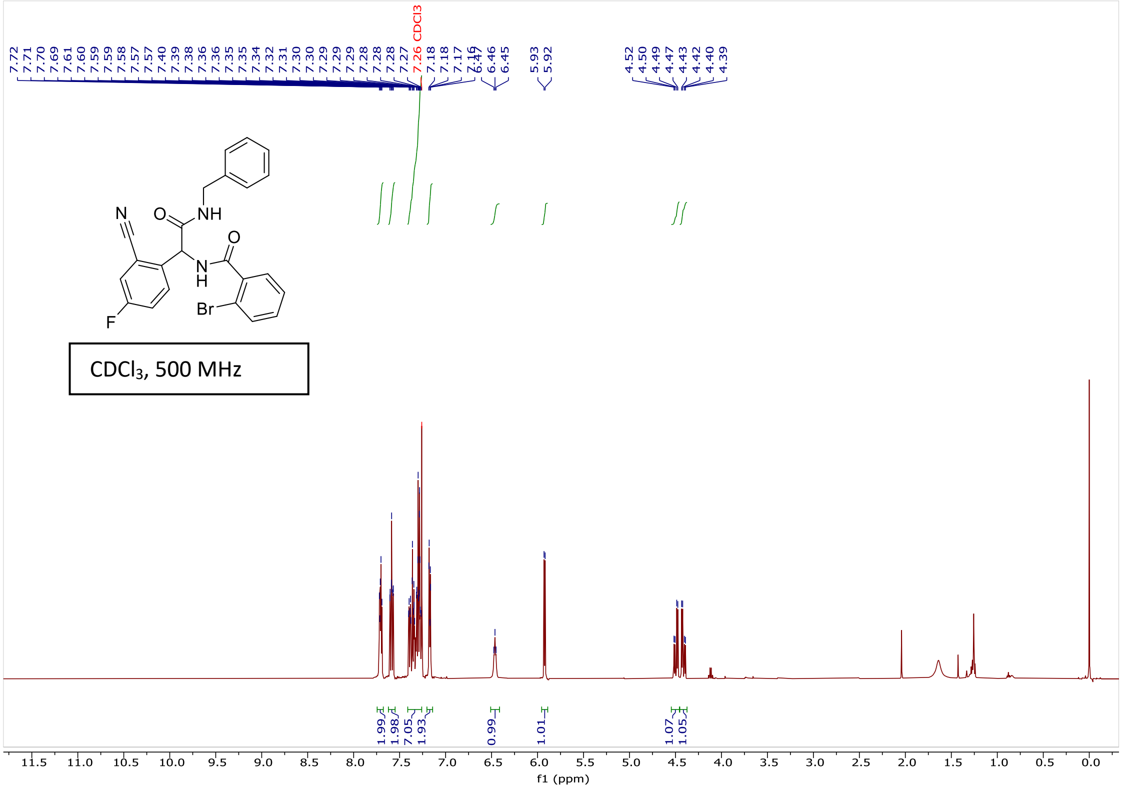

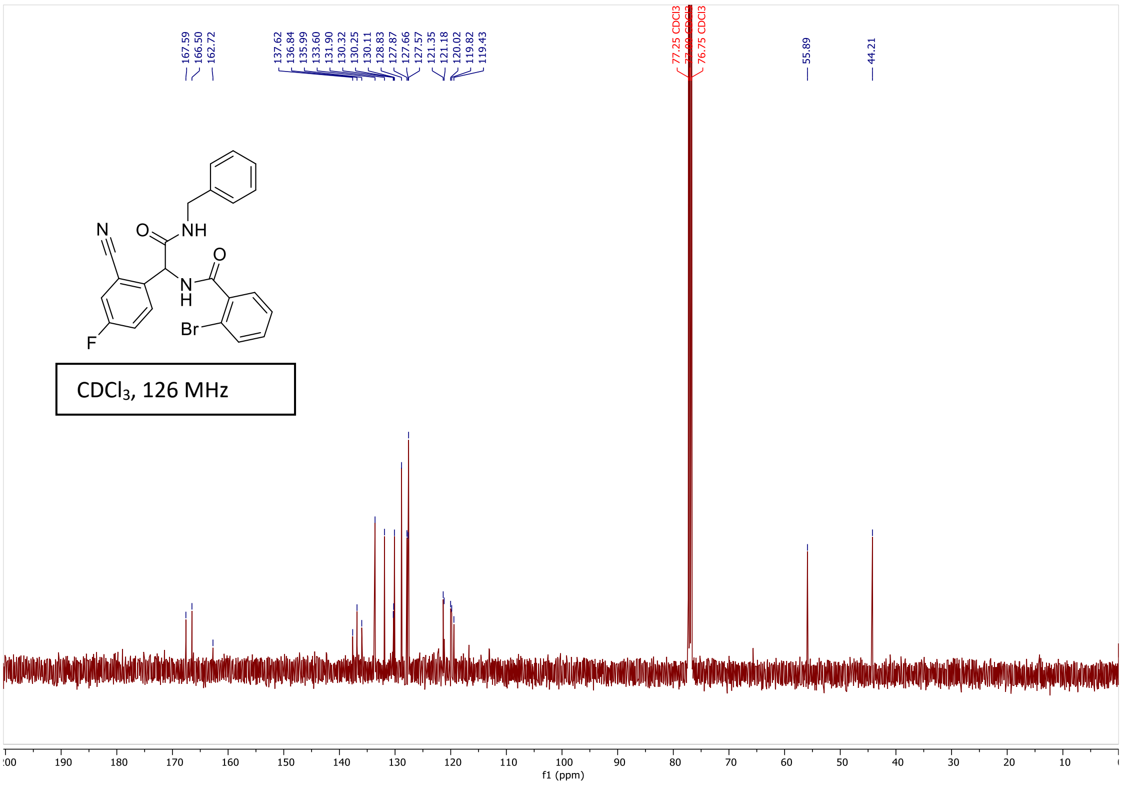

2-bromo-N-(2-((2-chloro-6-fluoro-3-methylbenzyl)amino)-1-(2-cyanophenyl)-2-oxoethyl)benzamide (**34a**)

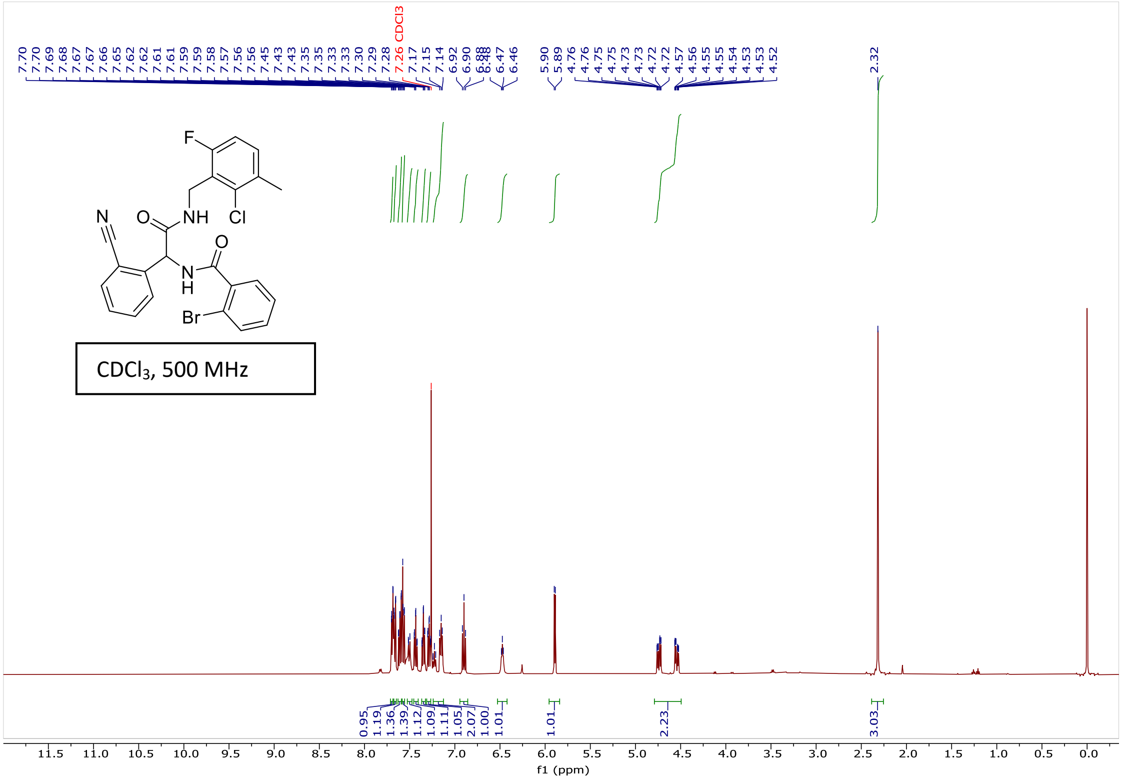

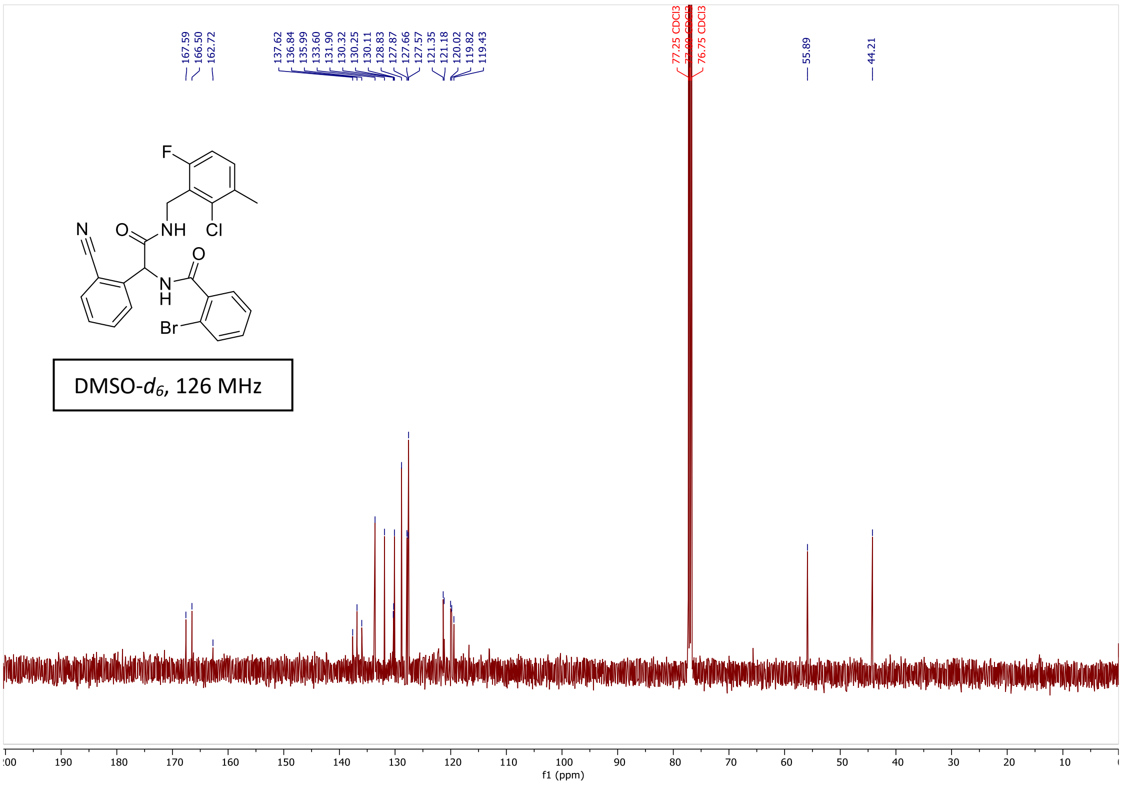

N-(2-(benzylamino)-1-(2-cyanoquinolin-3-yl)-2-oxoethyl)-2-bromobenzamide (**35a**)

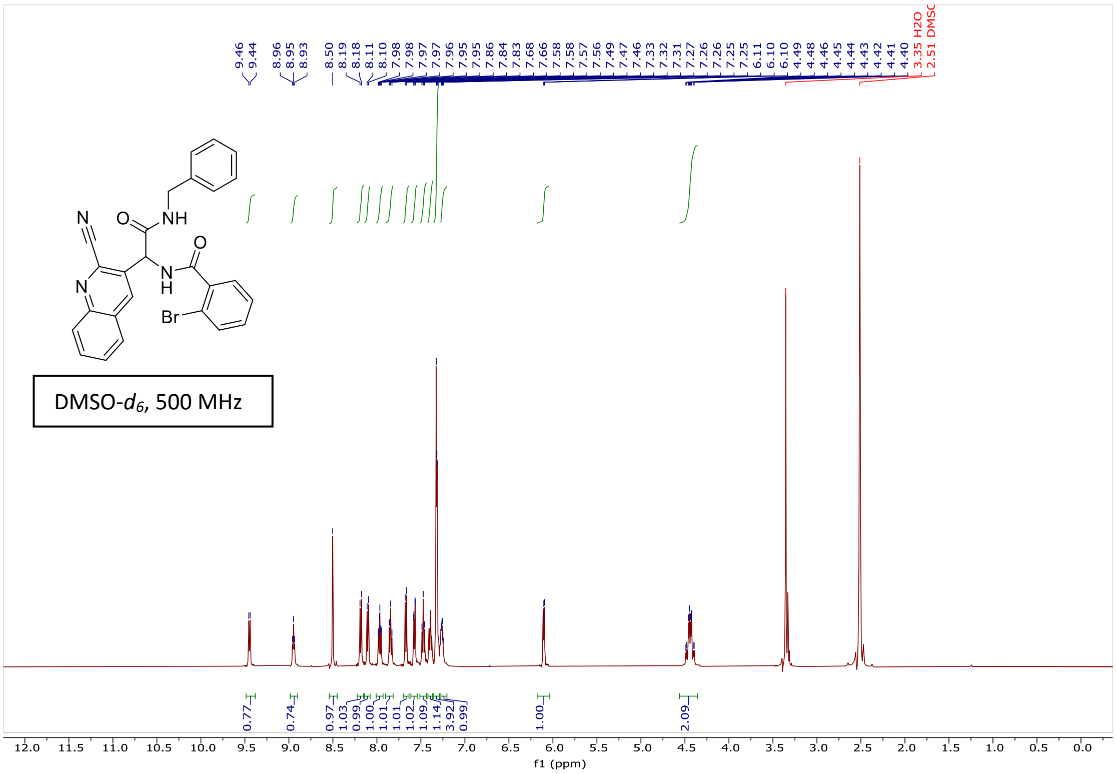

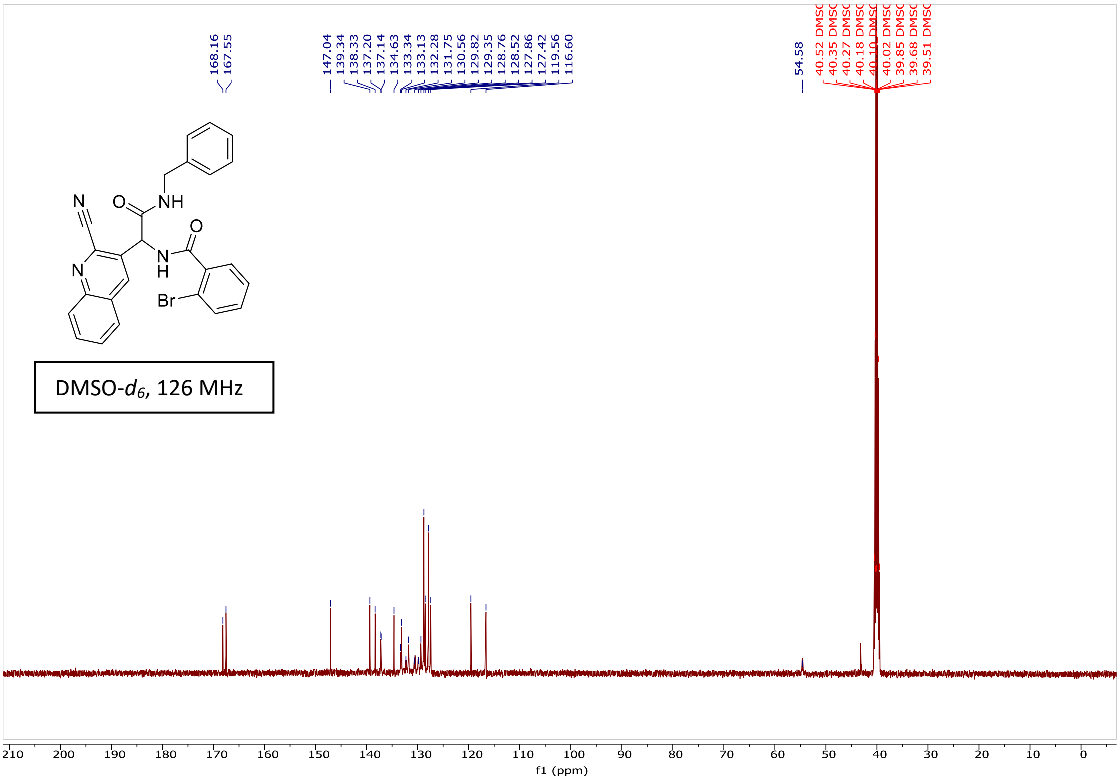

N-cyano-N-(2-(cyclohexylamino)-1-(2-iodophenyl)-2-oxoethyl)benzamide (**39a**)

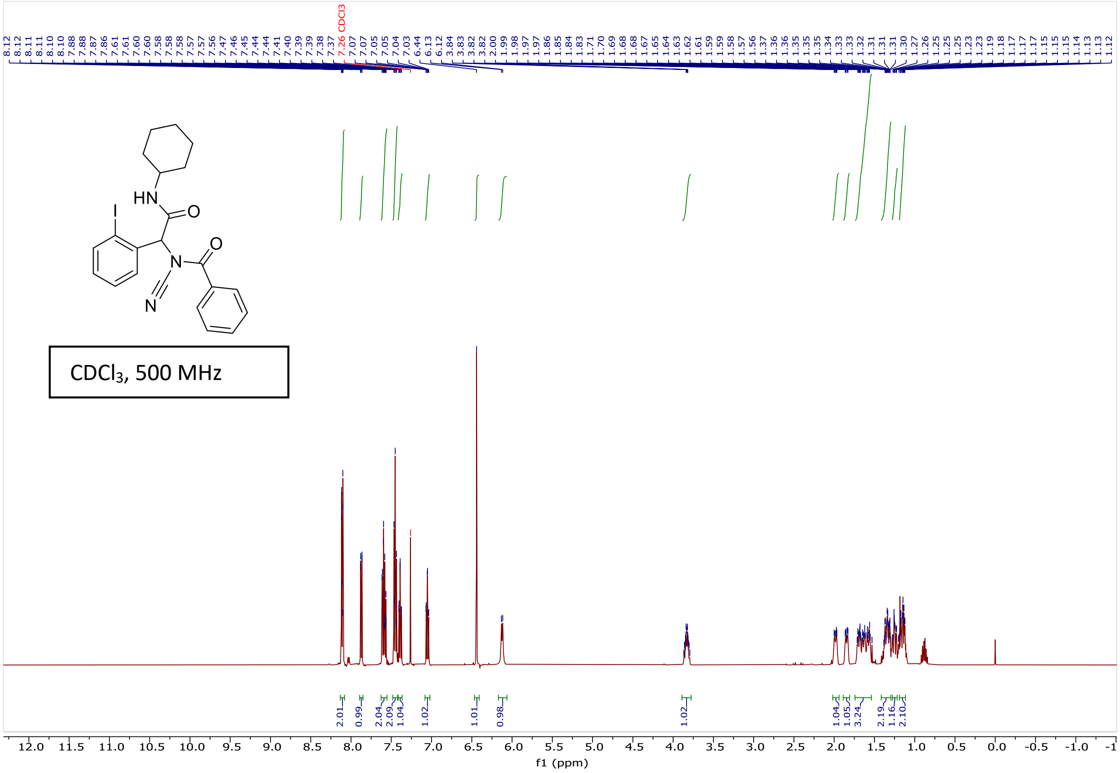

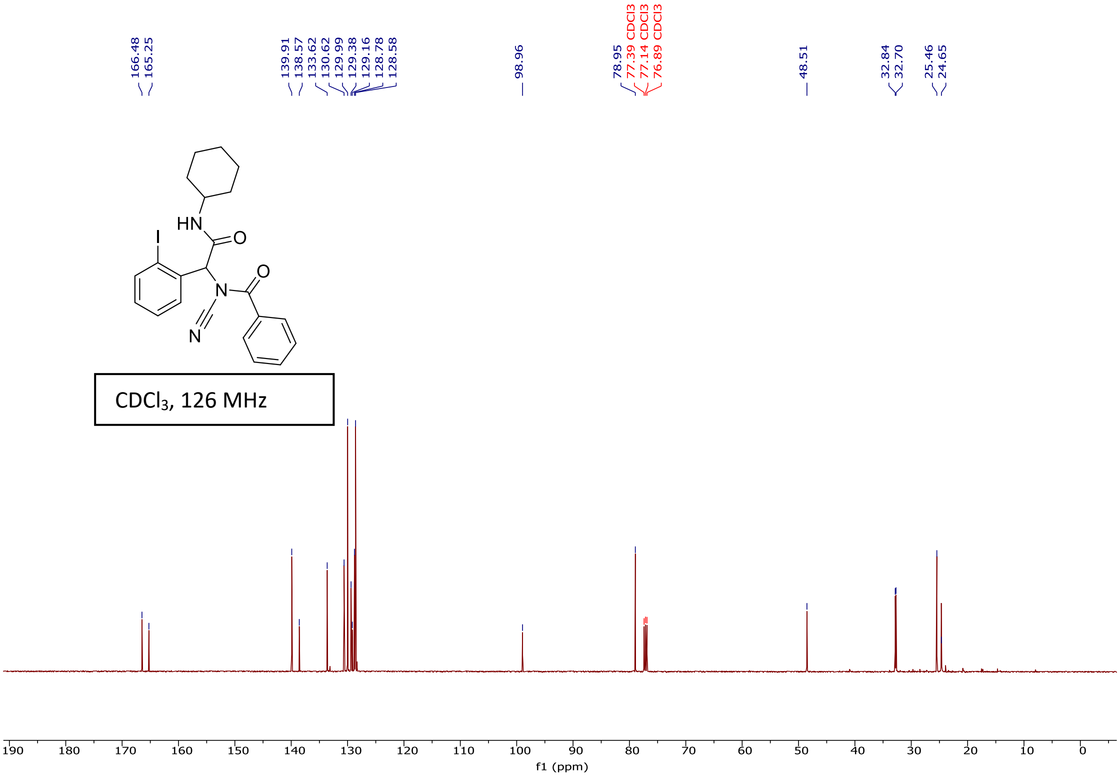

N-(2-(benzylamino)-1-(2-iodophenyl)-2-oxoethyl)-N-cyanobenzamide (**40a**)

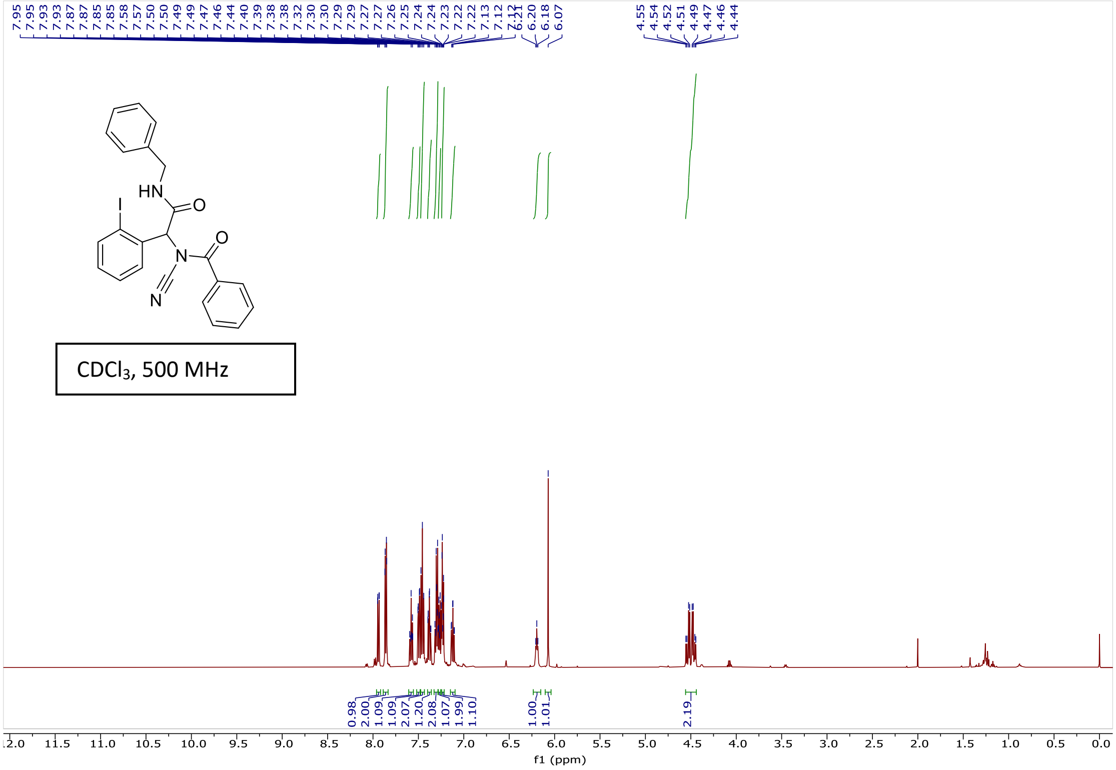

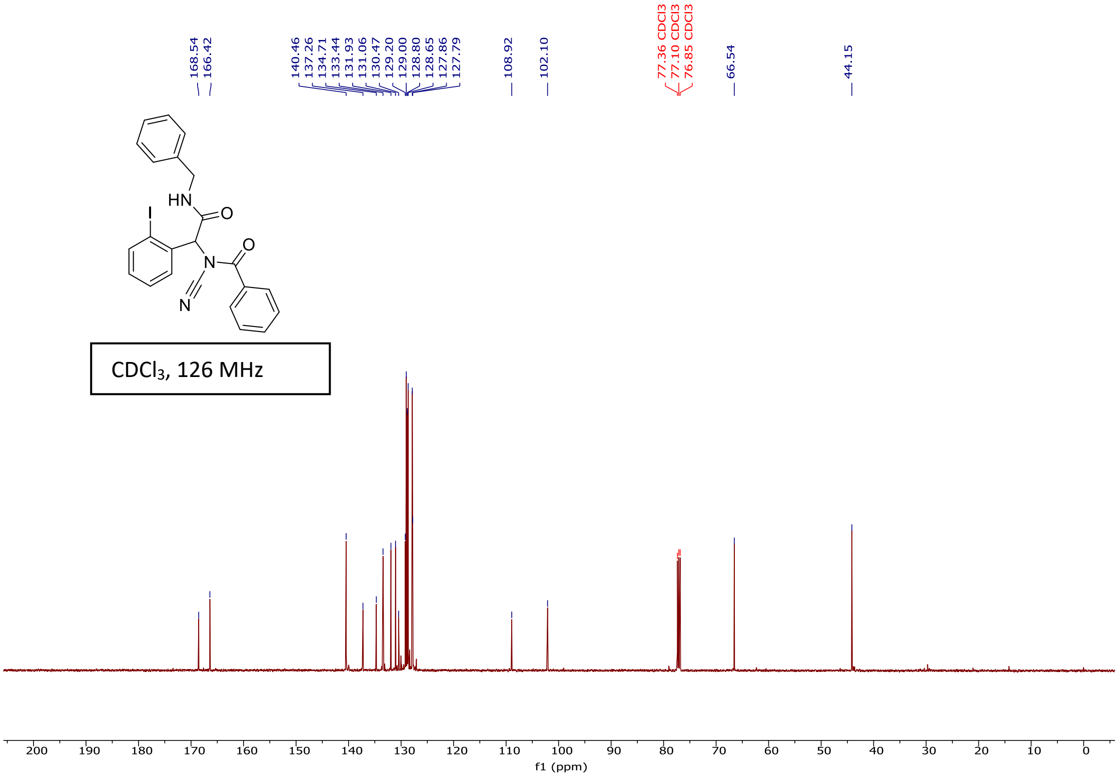

N-cyano-N-(1-(2-iodophenyl)-2-oxo-2-(((tetrahydrofuran-2-yl)methyl)amino)ethyl) benzamide (**41a**)

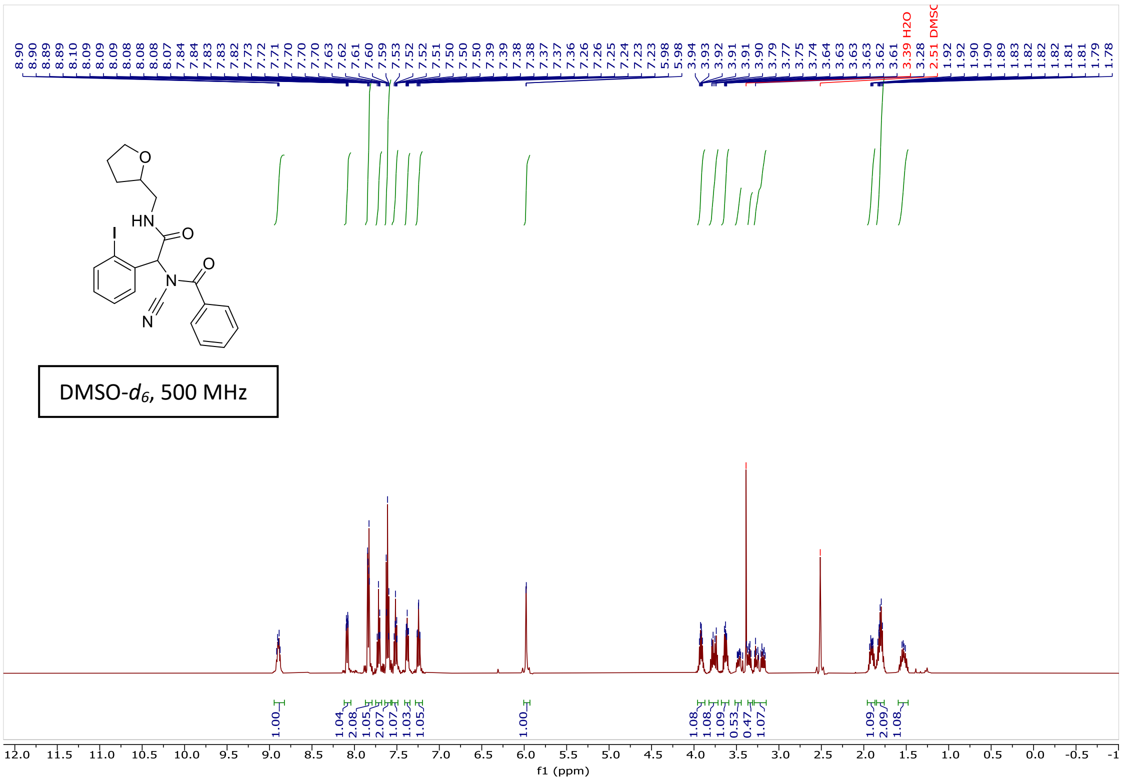

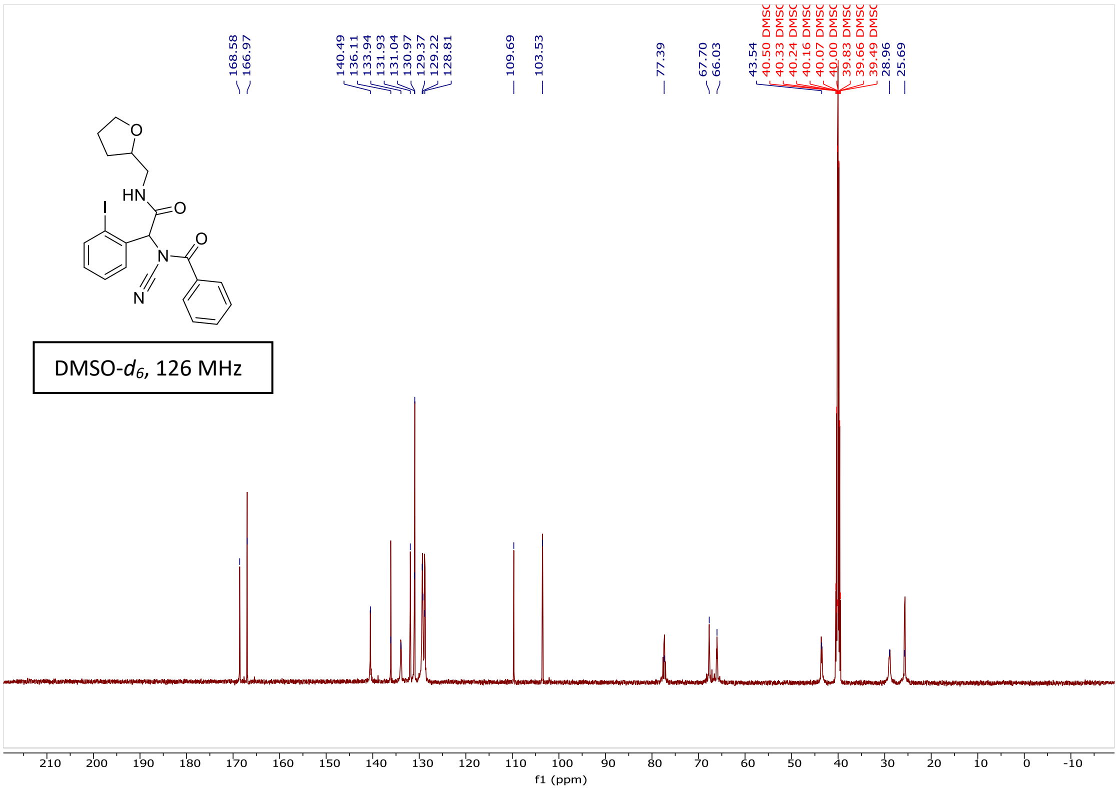

^{1}H NMR & ^{13}C{^{1}H} NMR chromatograms of cyclization products

N-cyclohexyl-10-oxo-10,12-dihydroisoindolo[1,2-b]quinazoline-12-carboxamide (**10b**)

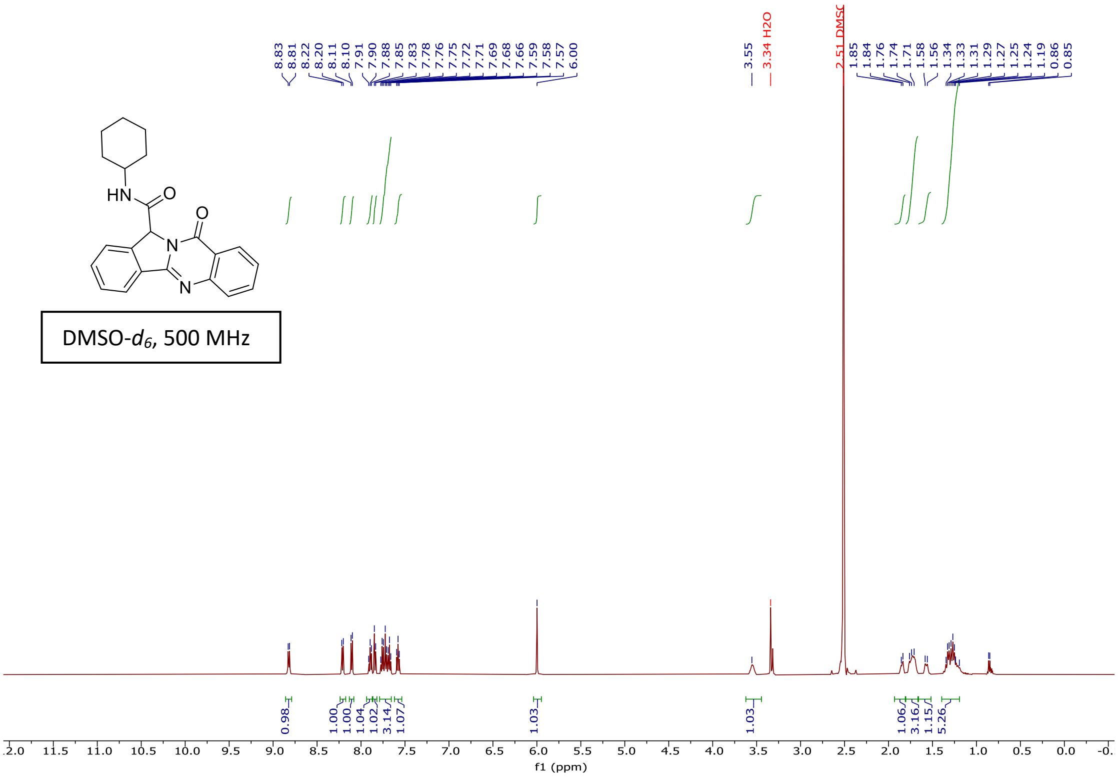

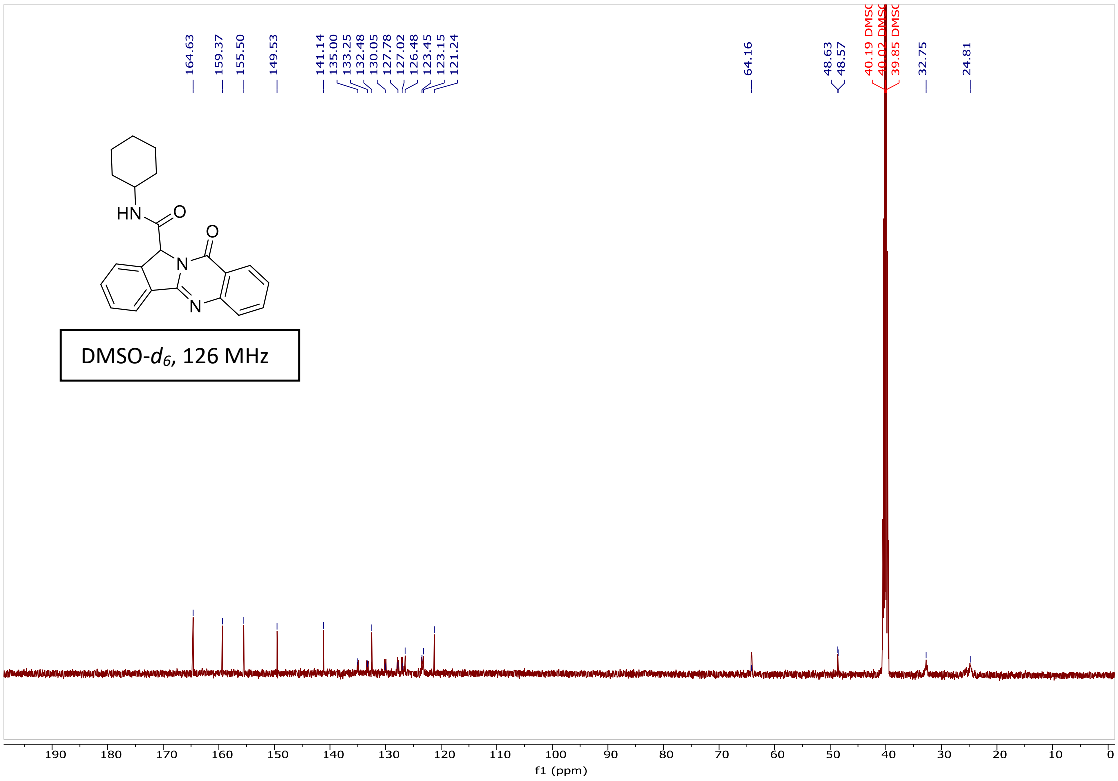

N-benzyl-10-oxo-10,12-dihydroisoindolo[1,2-b]quinazoline-12-carboxamide (**11b**)

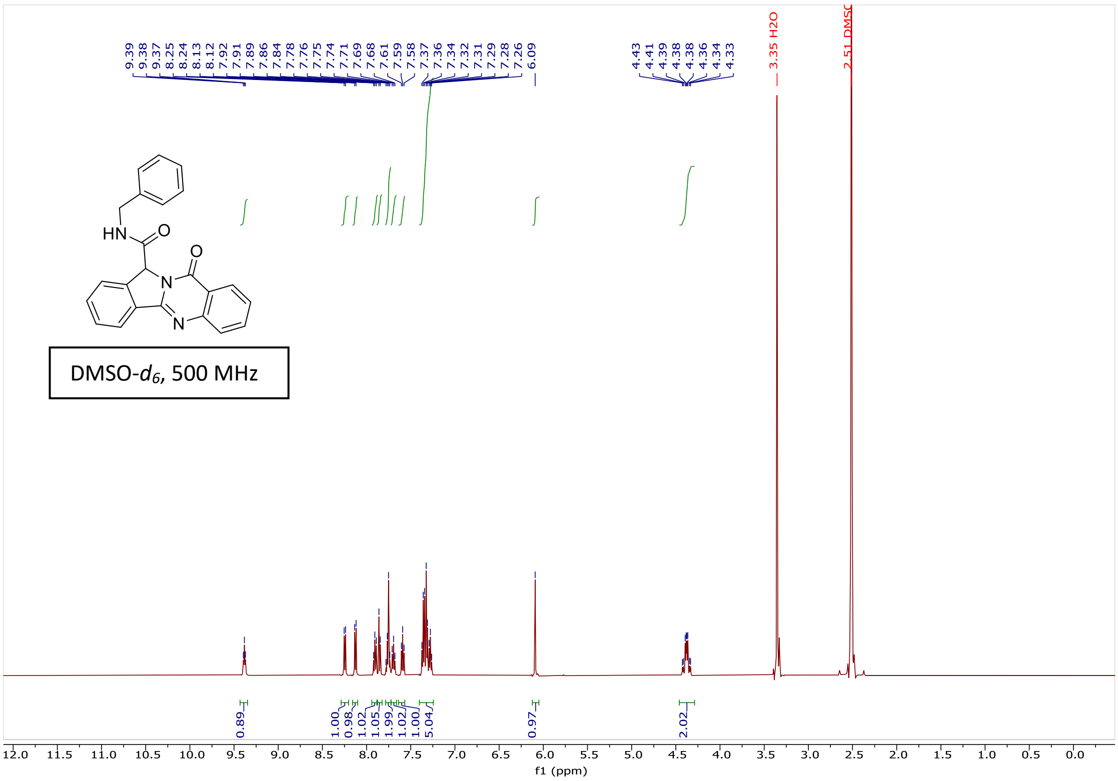

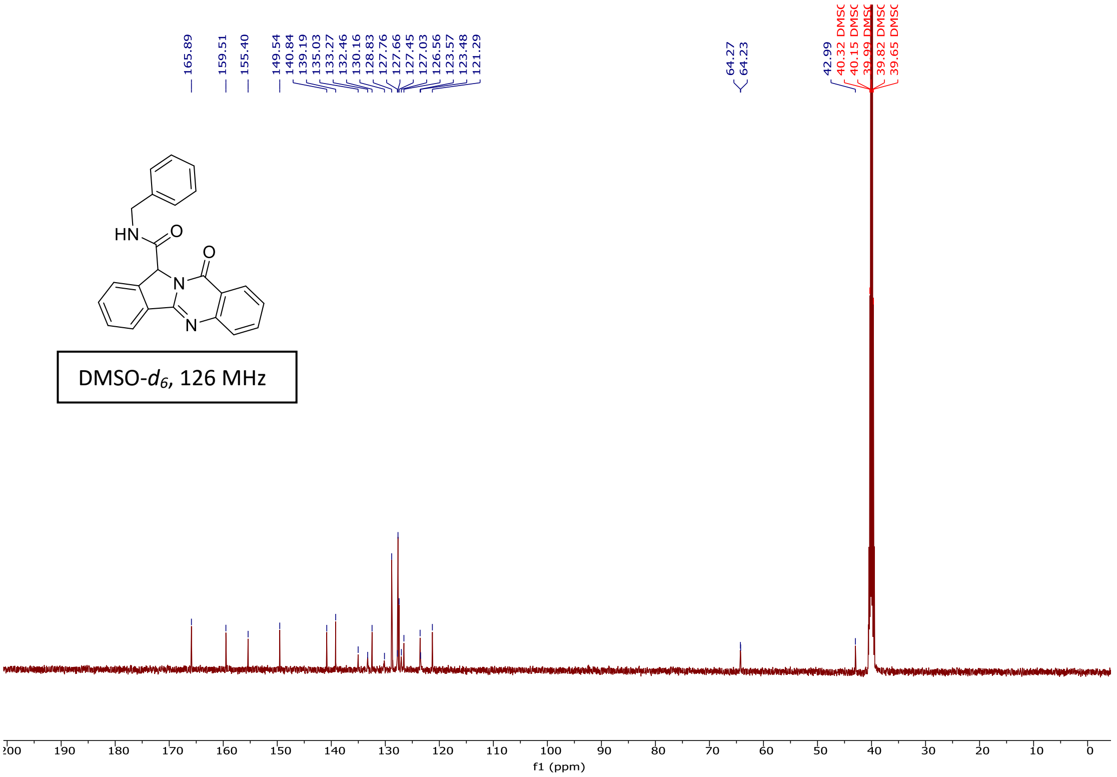

10-oxo-N-phenethyl-10,12-dihydroisoindolo[1,2-b]quinazoline-12-carboxamide (**12b**)

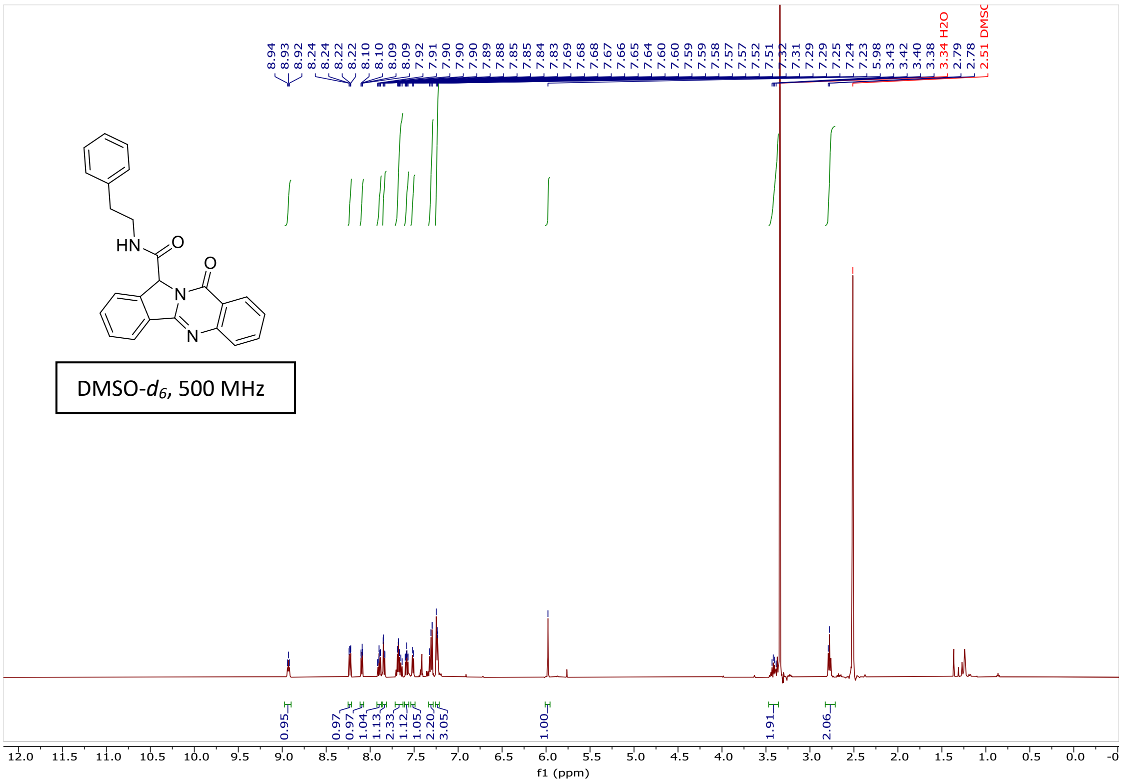

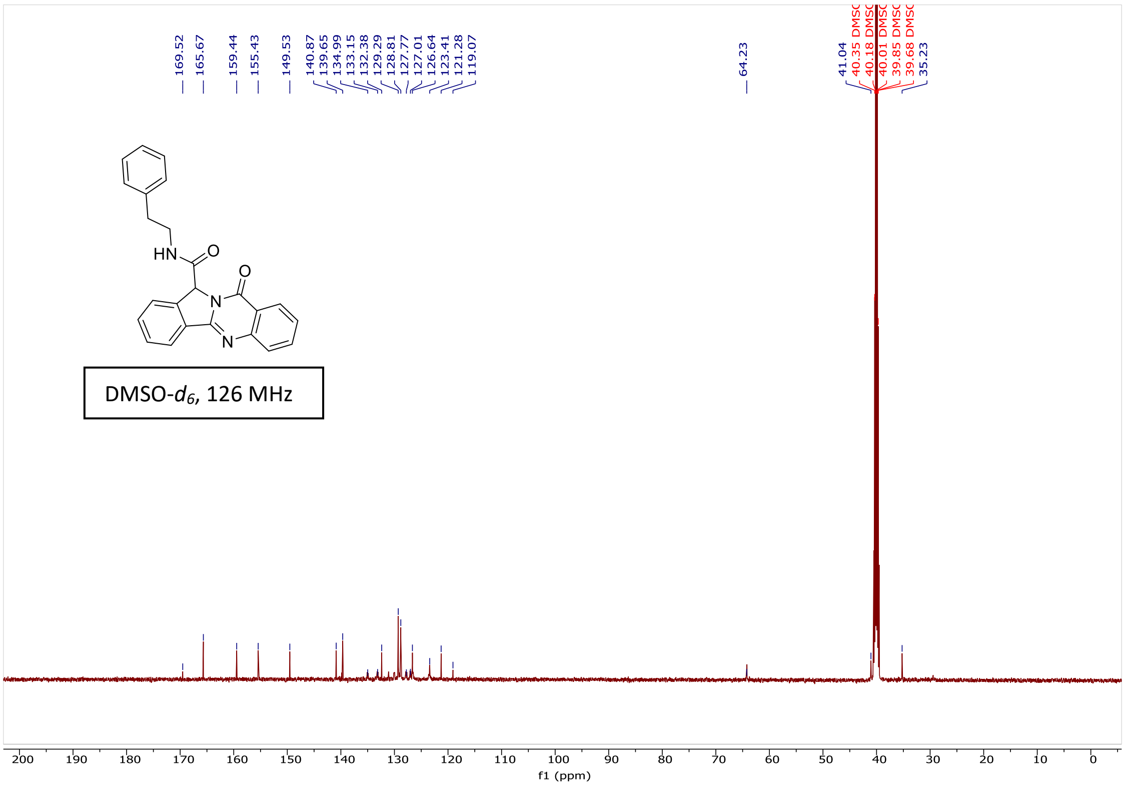

N-(4-chlorophenyl)-10-oxo-10,12-dihydroisoindolo[1,2-b]quinazoline-12-carboxamide (**13b**)

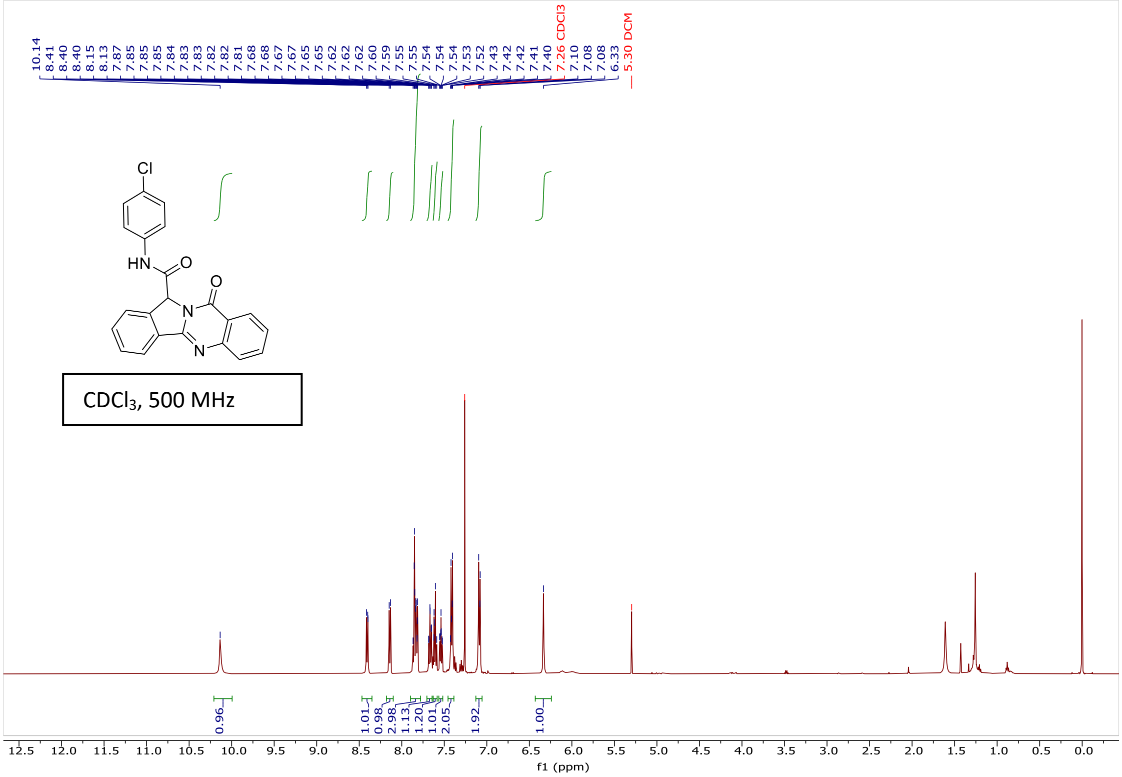

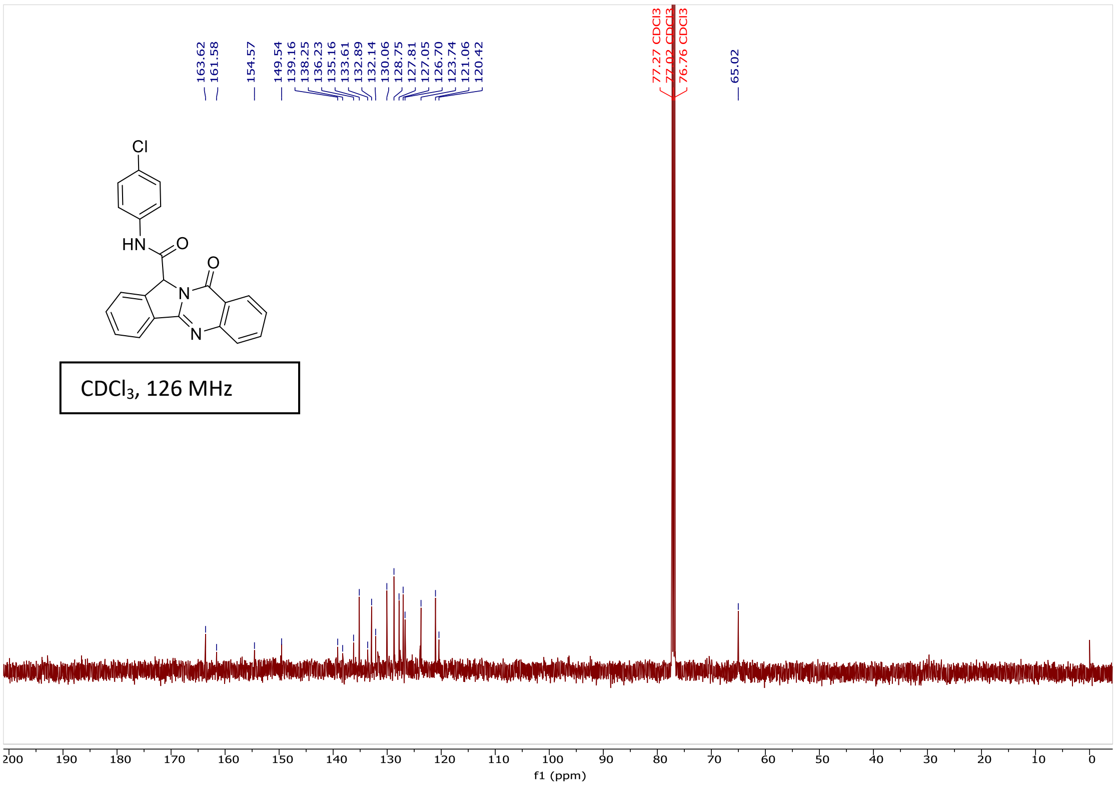

N-((3-methylthiophen-2-yl)methyl)-10-oxo-10,12-dihydroisoindolo[1,2-b] quinazoline-12-carboxamide (**14b**)

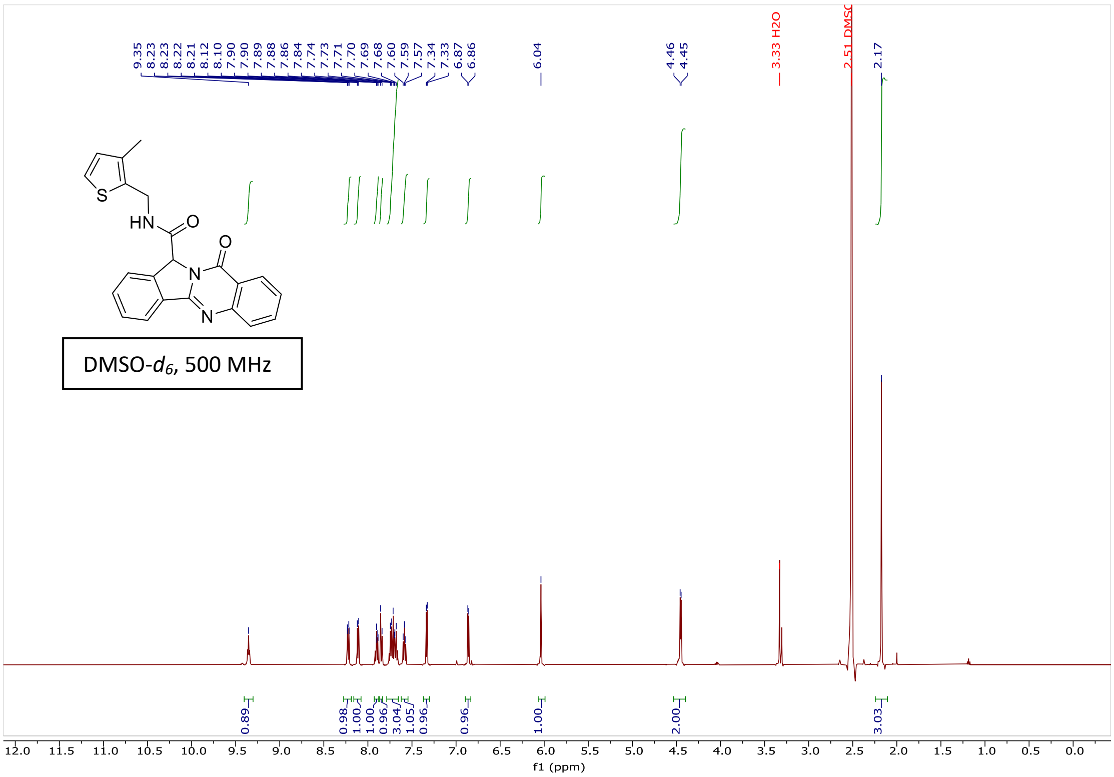

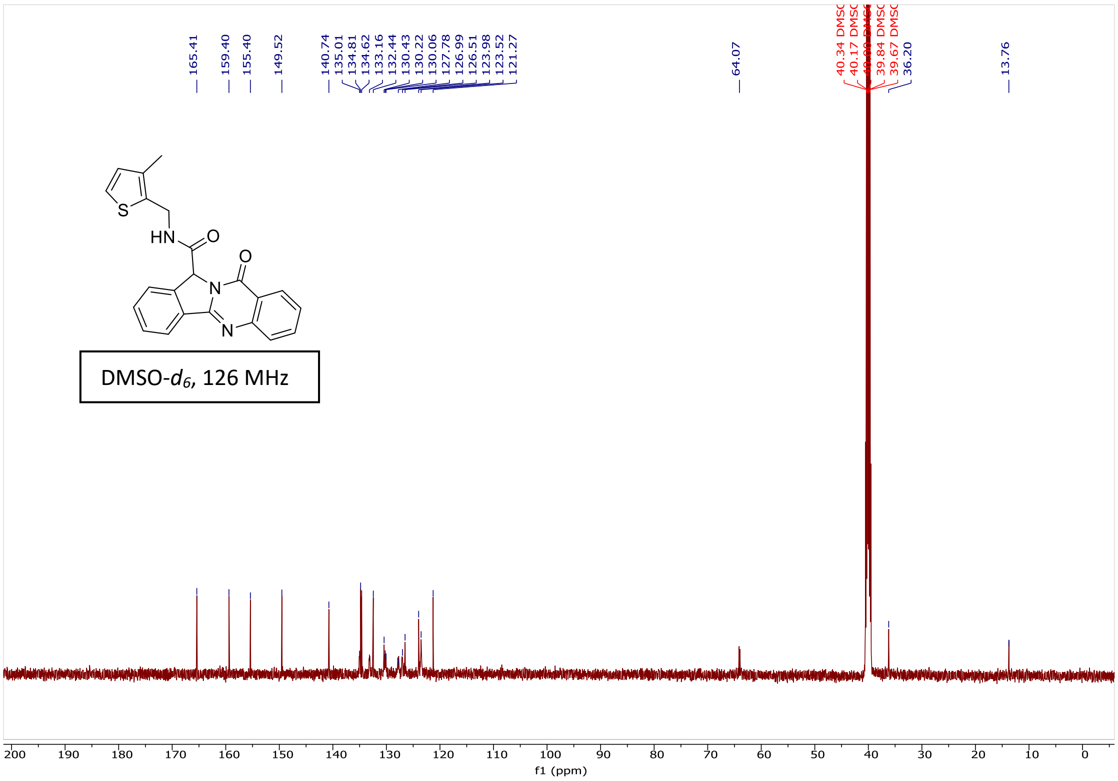

N-(2-methoxybenzyl)-10-oxo-10,12-dihydroisoindolo[1,2-b]quinazoline-12-carboxamide (**15b**)

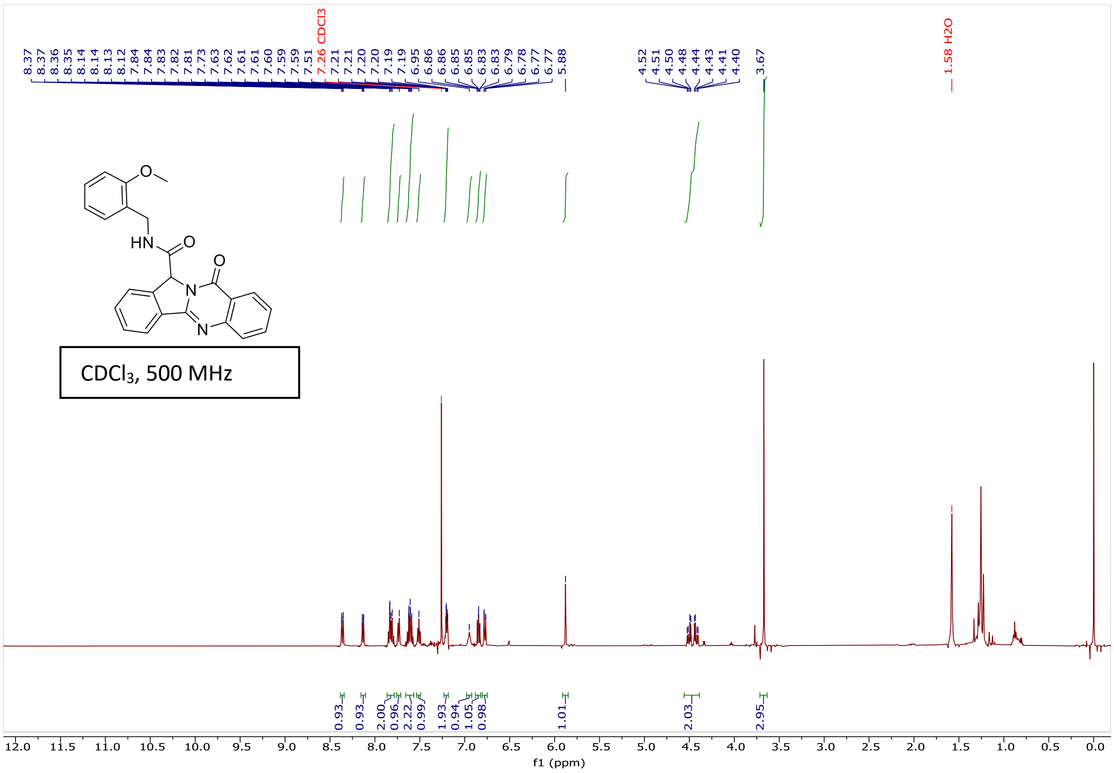

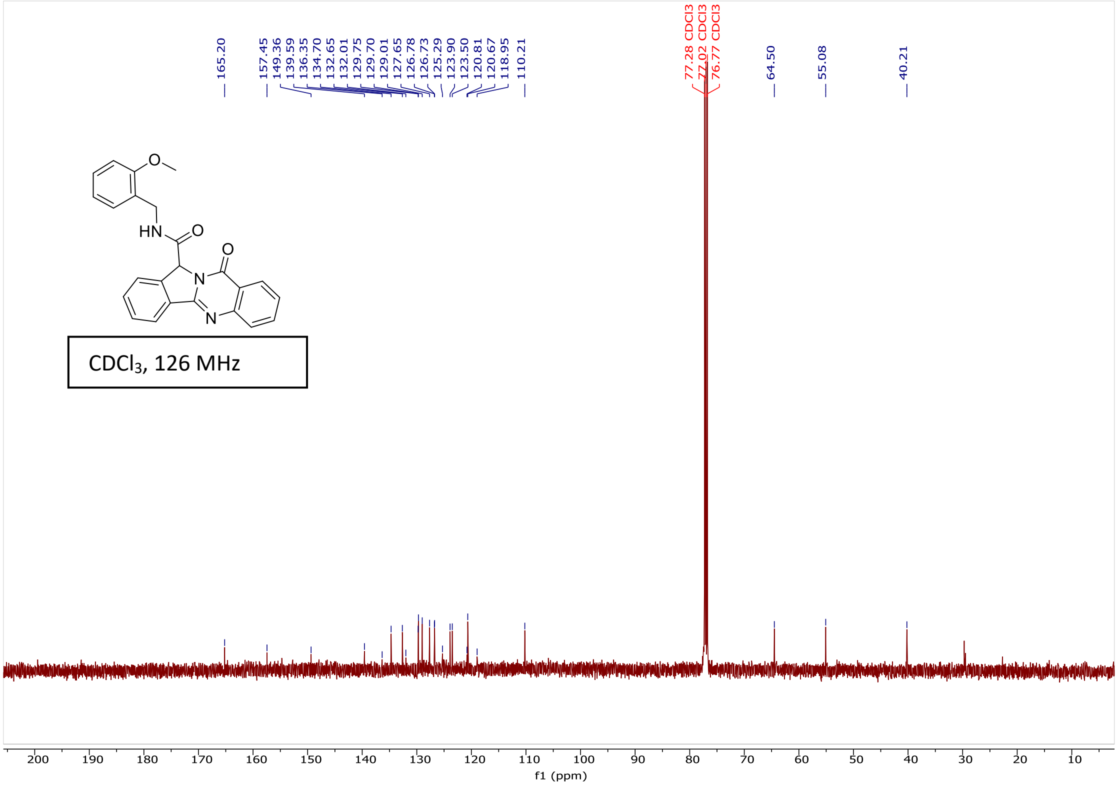

10-oxo-N-(pyridin-3-ylmethyl)-10,12-dihydroisoindolo[1,2-b]quinazoline-12-carboxamide (**16b**)

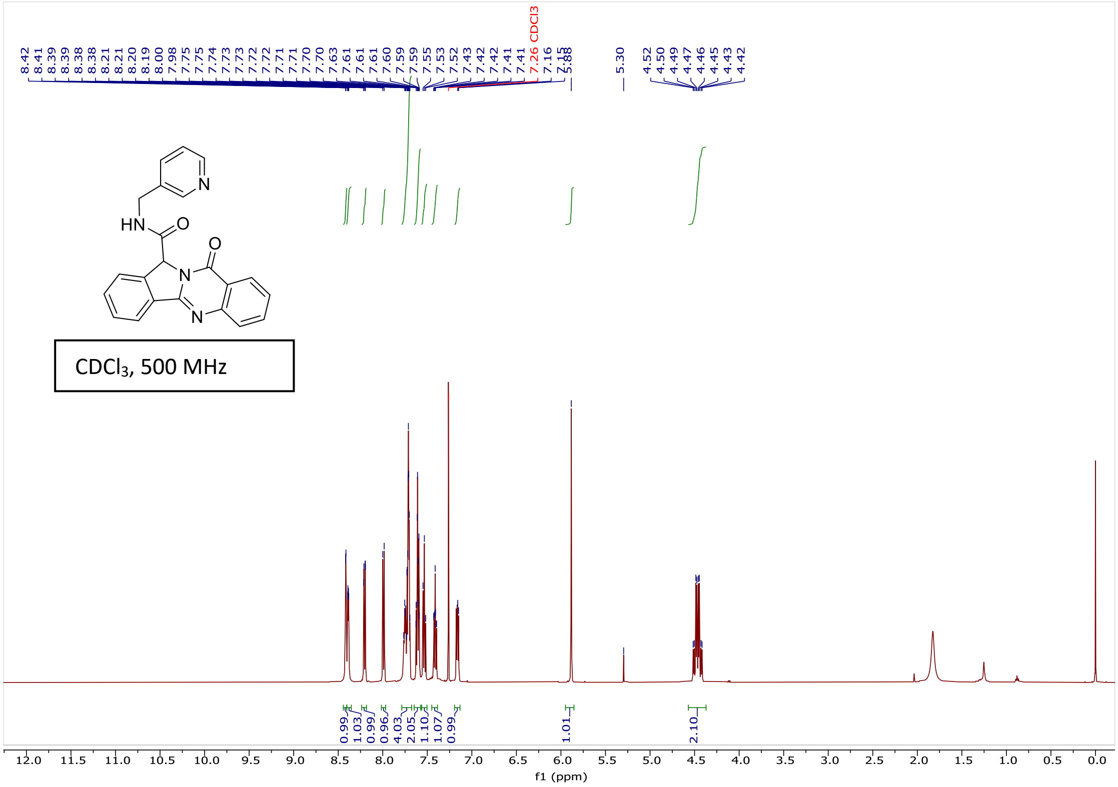

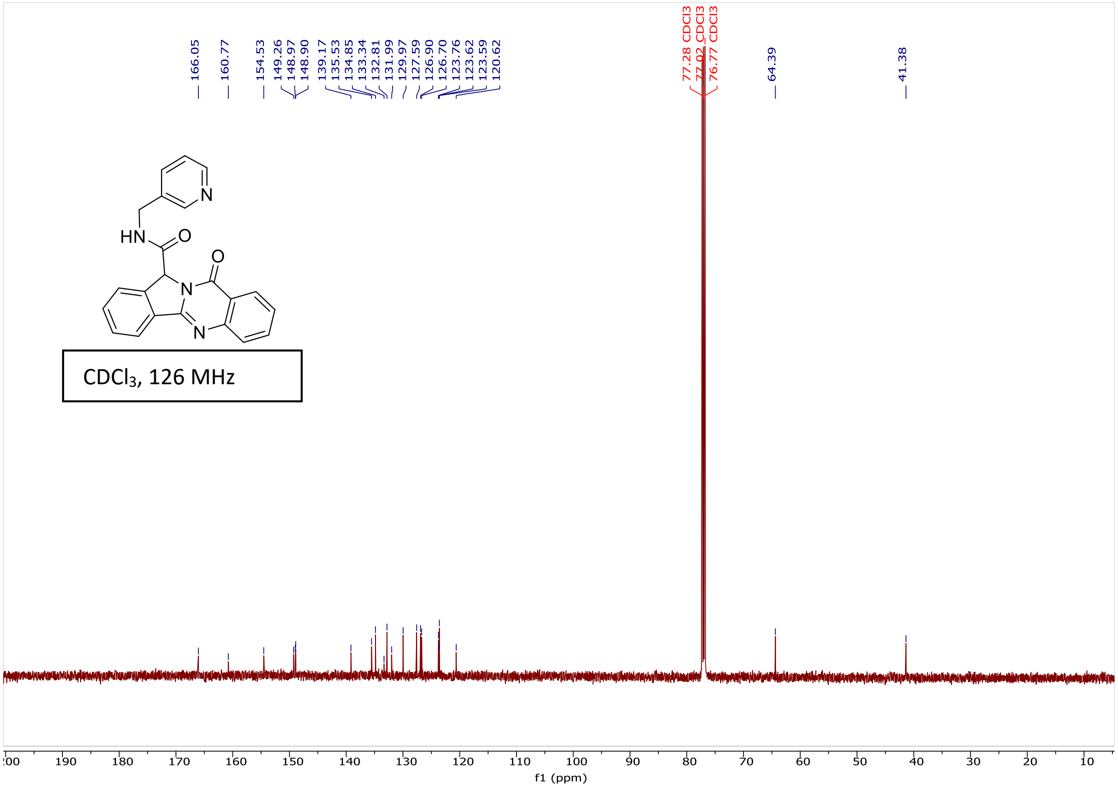

N-((3S,5S)-adamantan-1-yl)-10-oxo-10,12-dihydroisoindolo[1,2-b]quinazoline-12-carboxamide (**17b**)

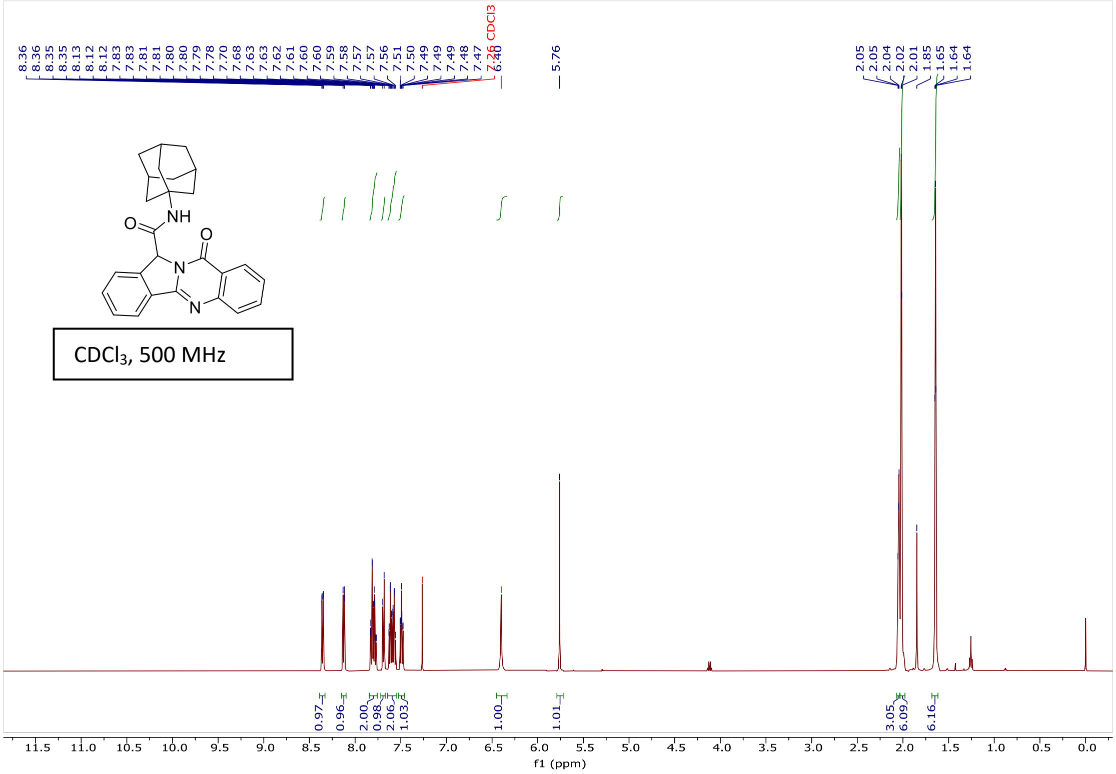

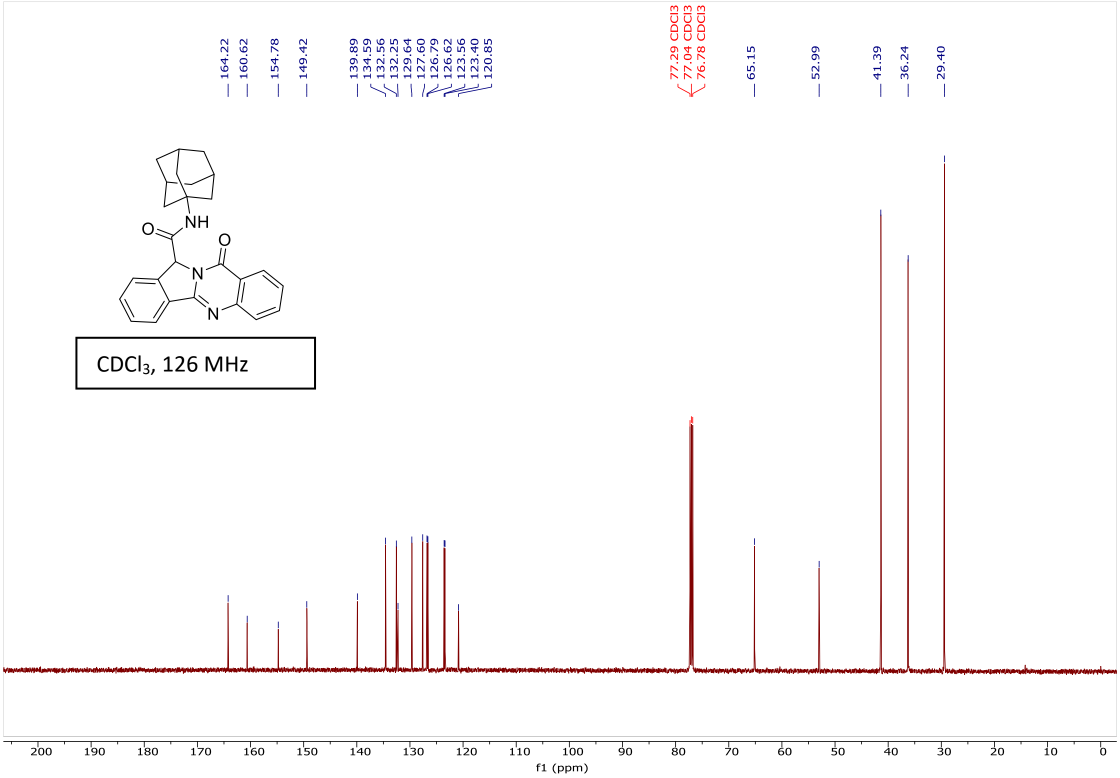

10-oxo-N-((tetrahydrofuran-2-yl)methyl)-10,12-dihydroisoindolo[1,2-b]quinazoline-12-carboxamide (**18b**)

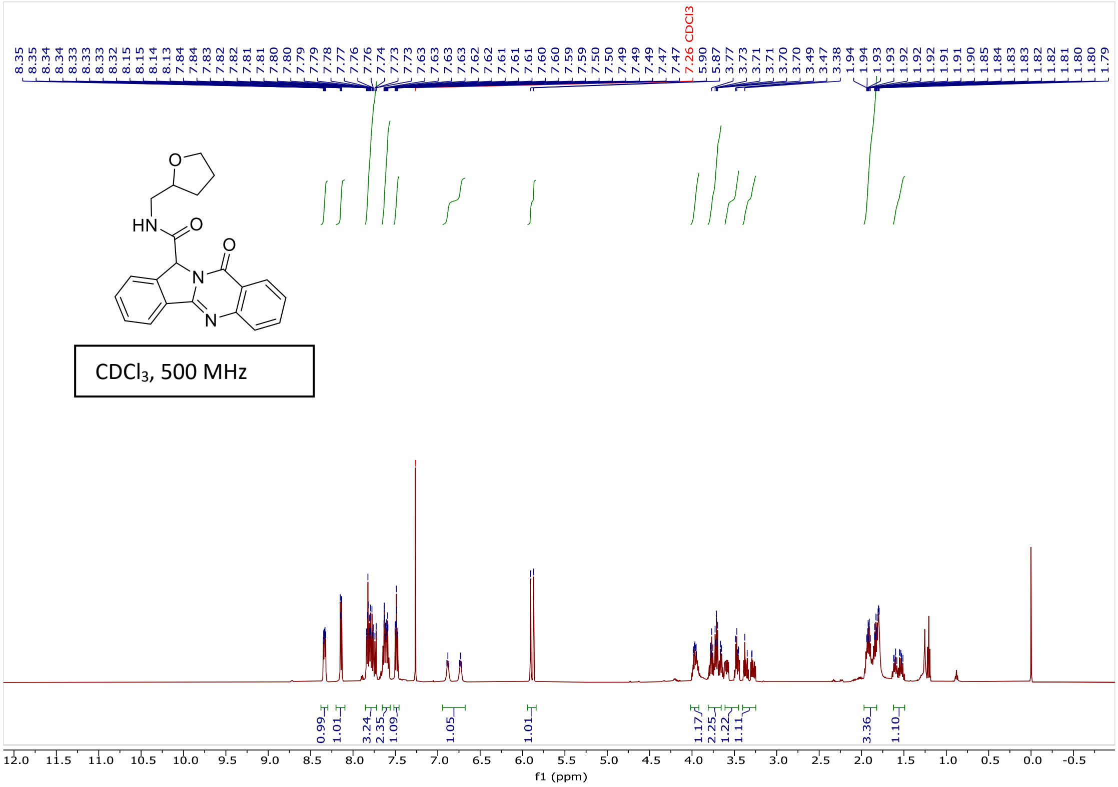

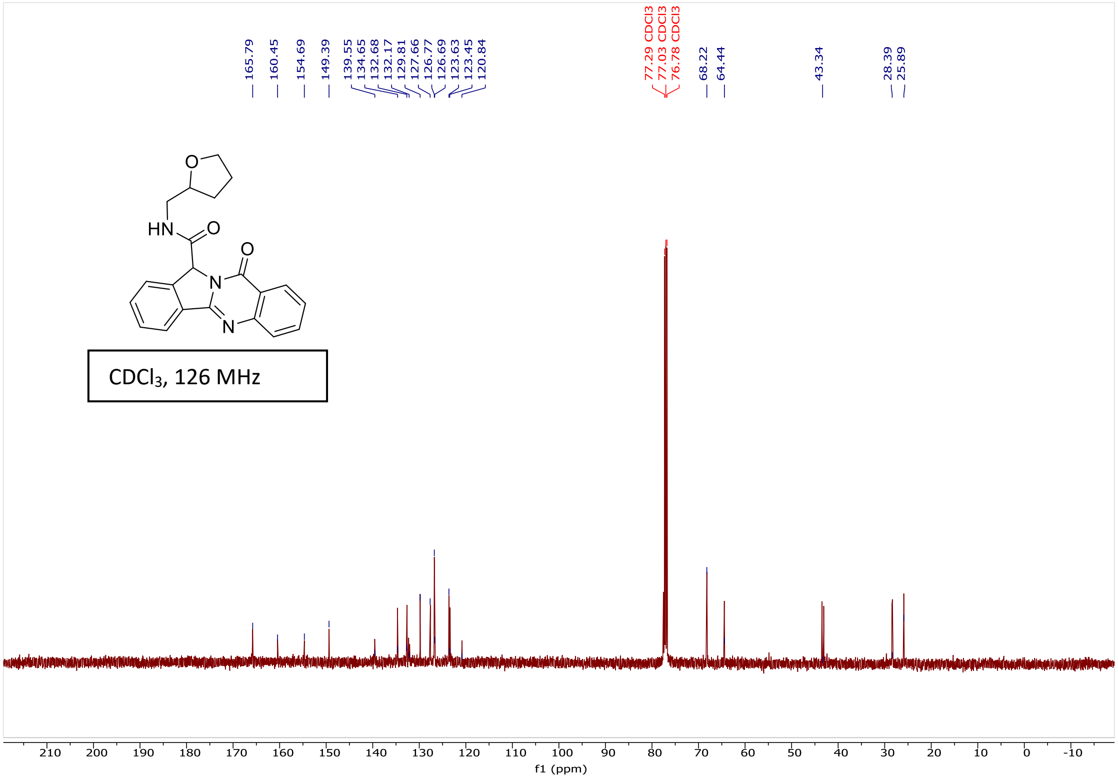

10-oxo-N-(2,4,4-trimethylpentan-2-yl)-10,12-dihydroisoindolo[1,2-b]quinazoline-12-carboxamide (**19b**)

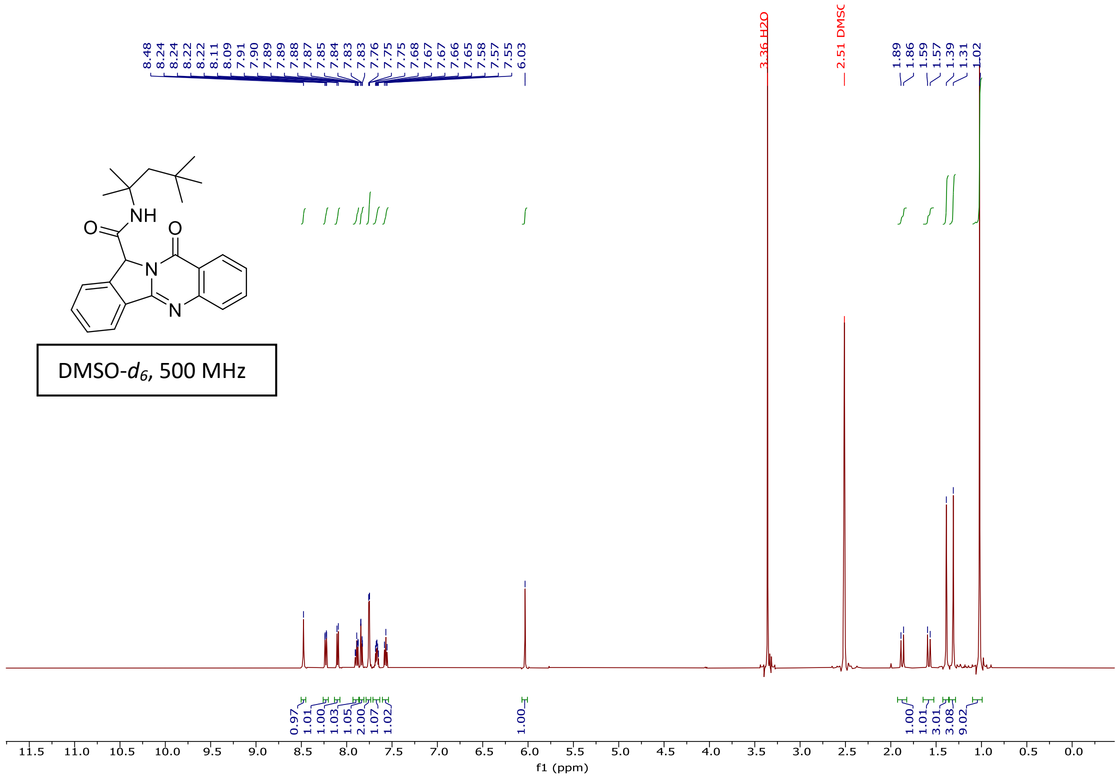

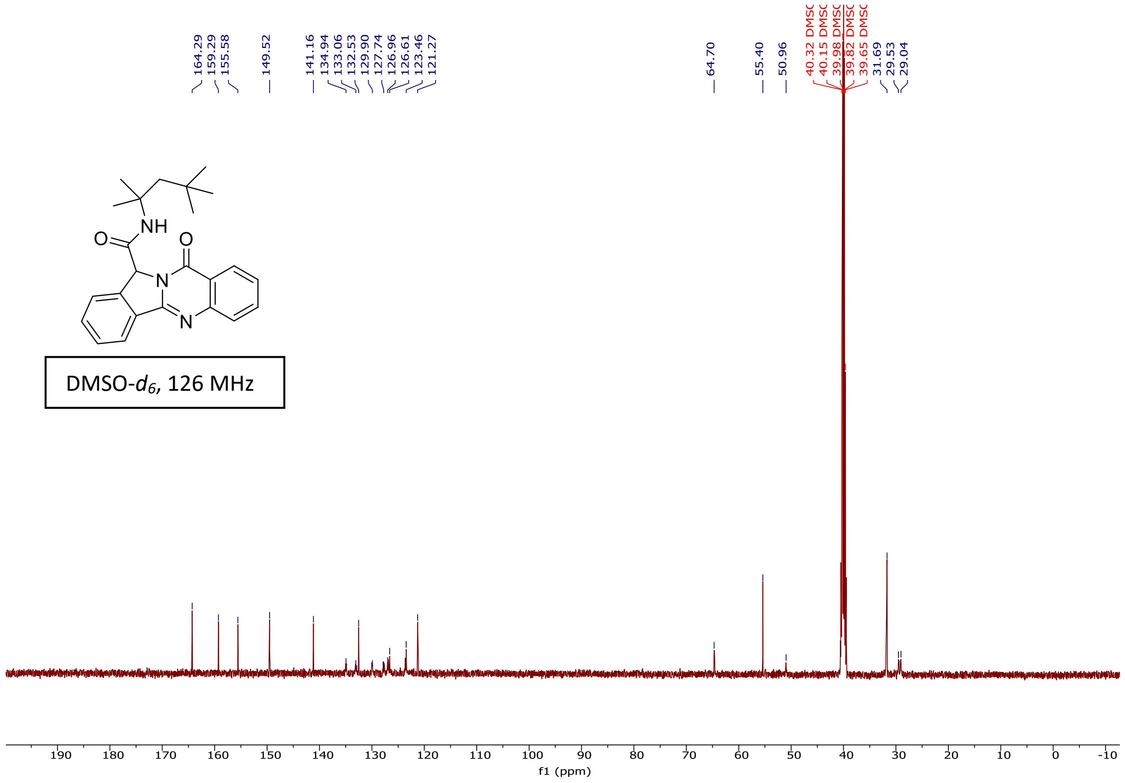

methyl (10-oxo-10,12-dihydroisoindolo[1,2-b]quinazoline-12-carbonyl)glycinate (**20b**)

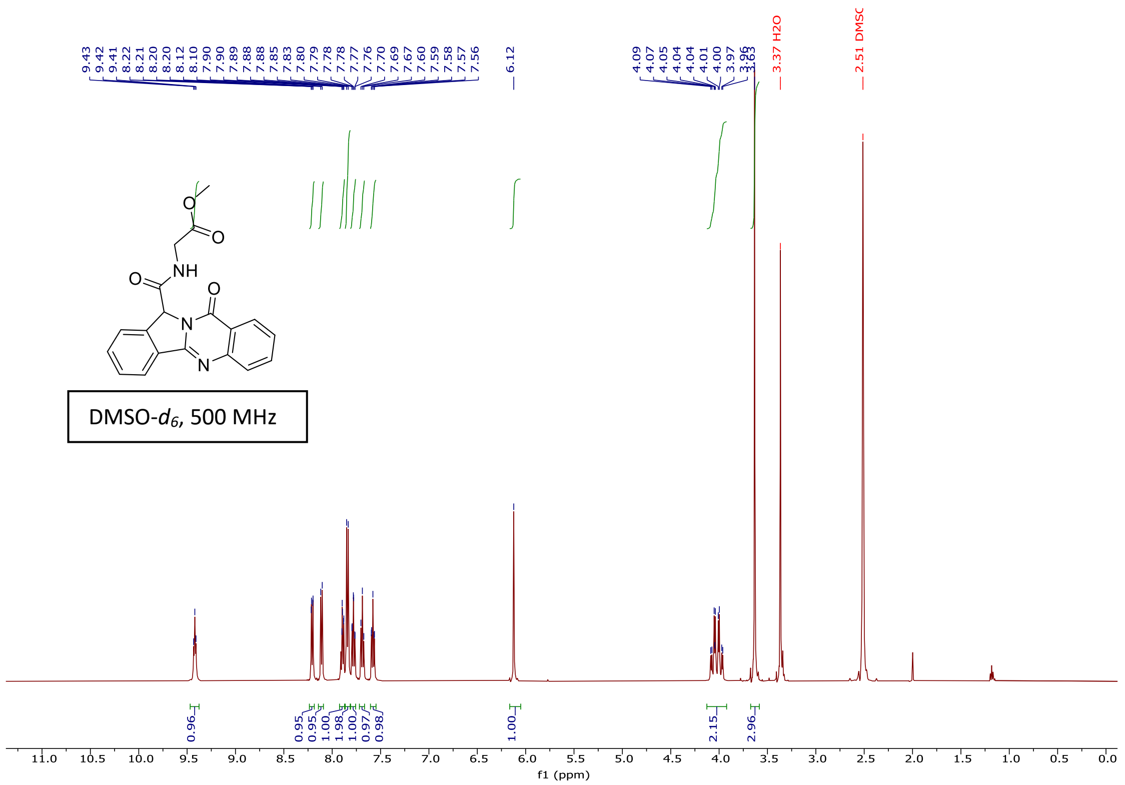

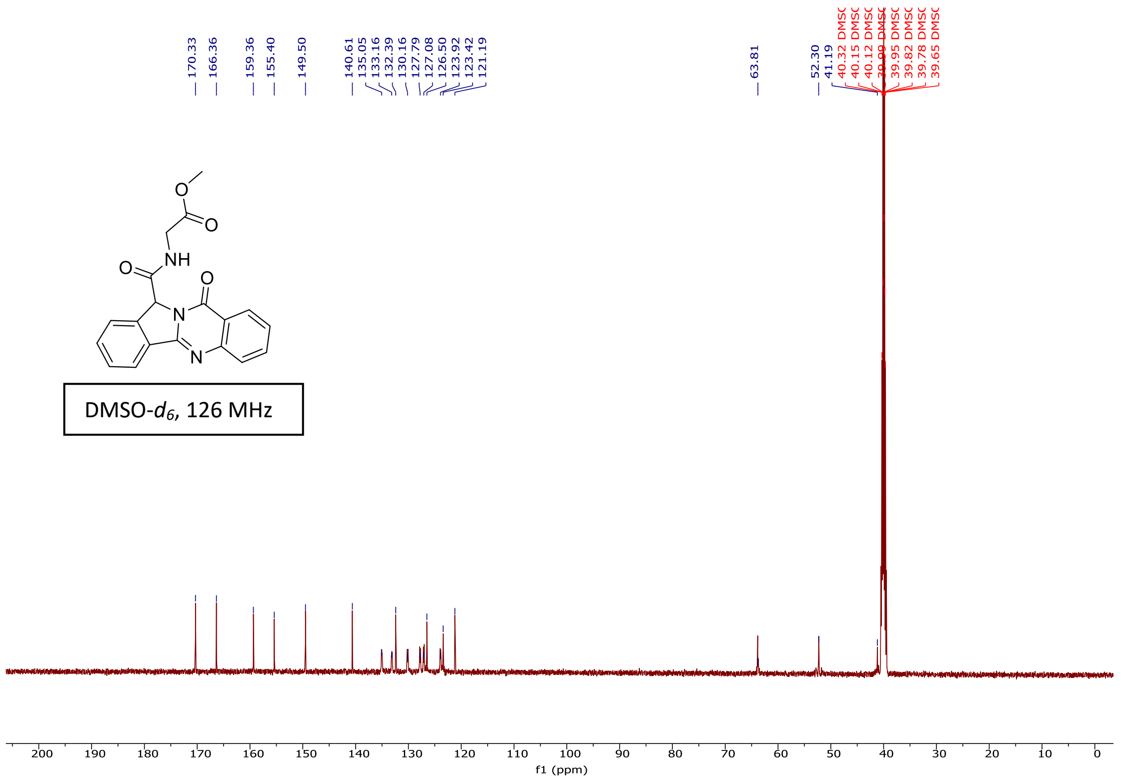

tert-butyl (2-(10-oxo-10,12-dihydroisoindolo[1,2-b]quinazoline-12-carboxamido) ethyl)-carbamate (**21b**)

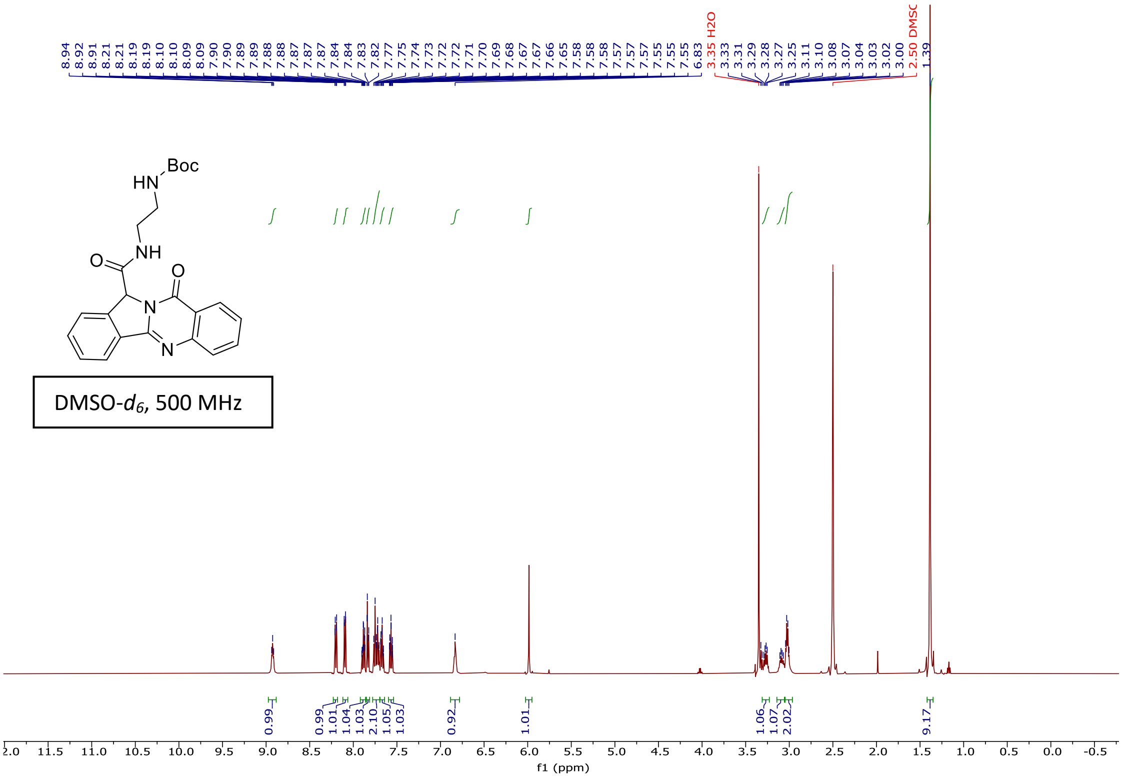

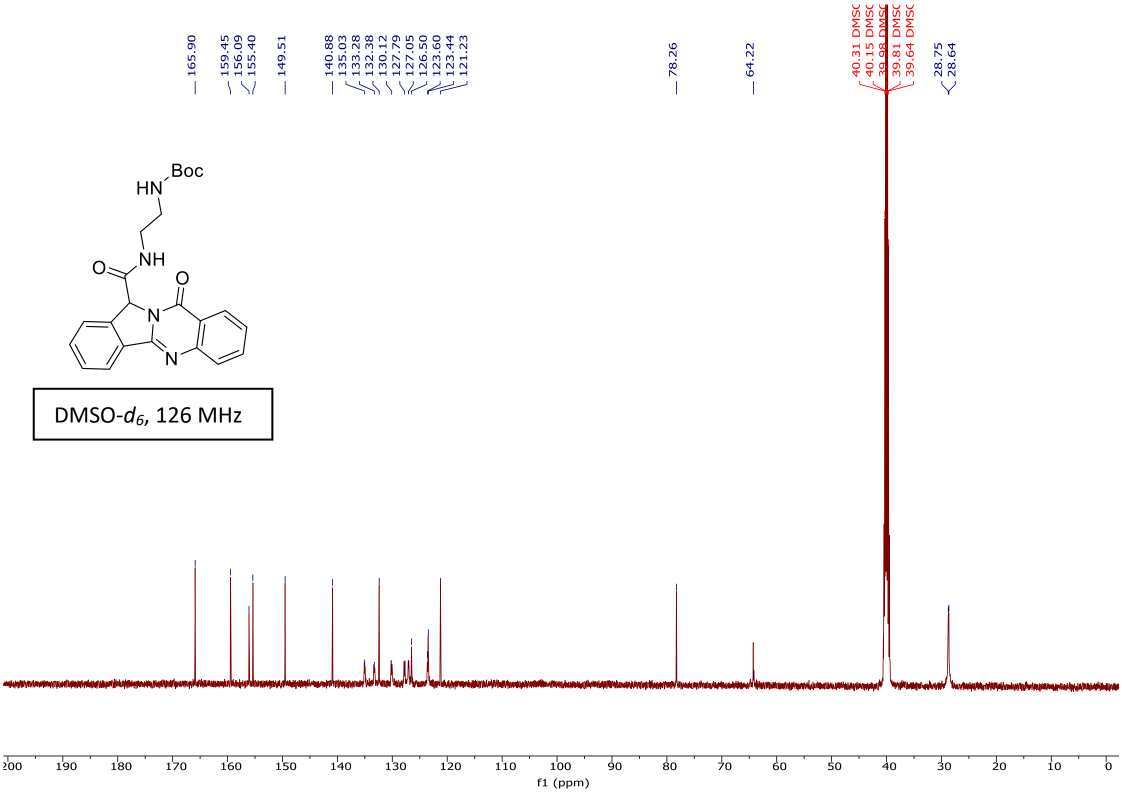

N-(3-cyanobenzyl)-10-oxo-10,12-dihydroisoindolo[1,2-b]quinazoline-12-carboxamide (**22b**)

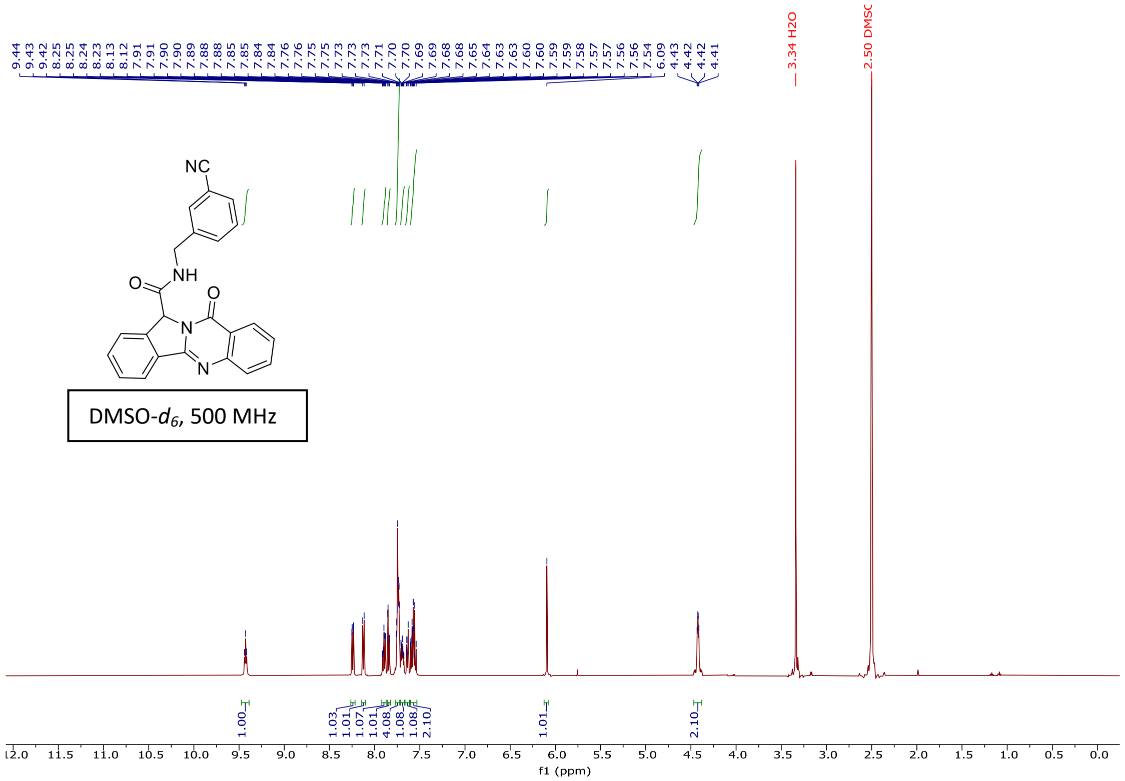

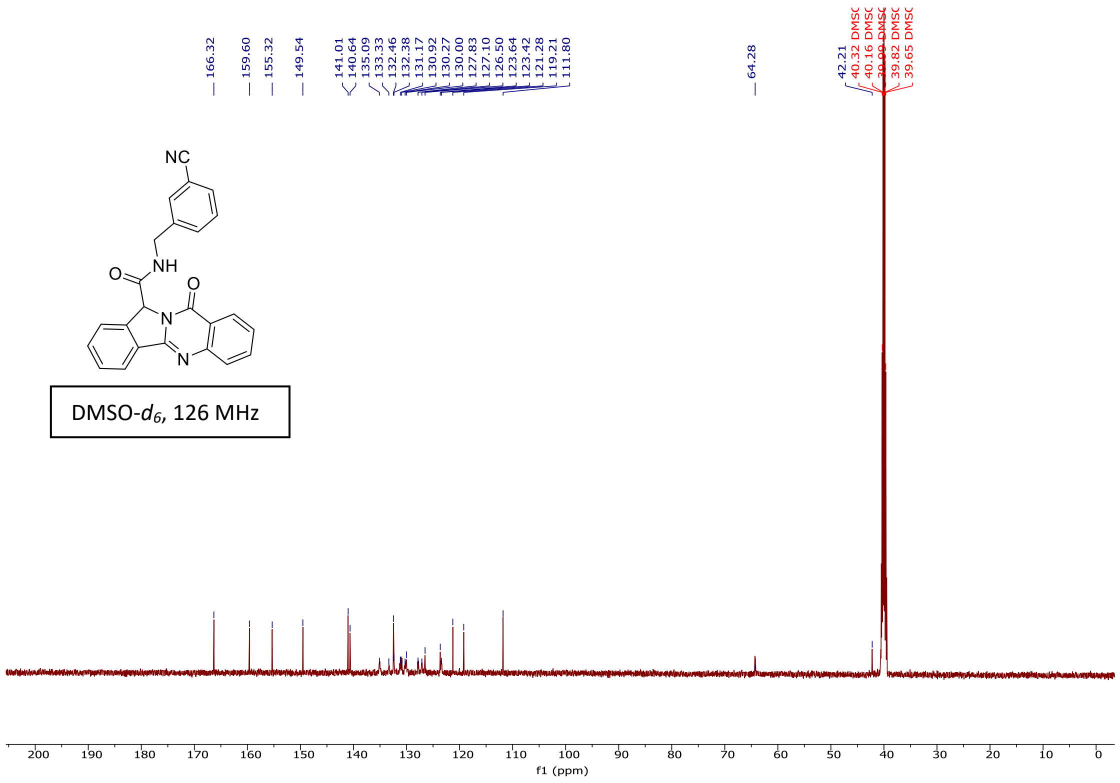

N-(2-chloro-3,6-difluorobenzyl)-10-oxo-10,12-dihydroisoindolo[1,2-b]quinazoline-12-carboxamide (**23b**)

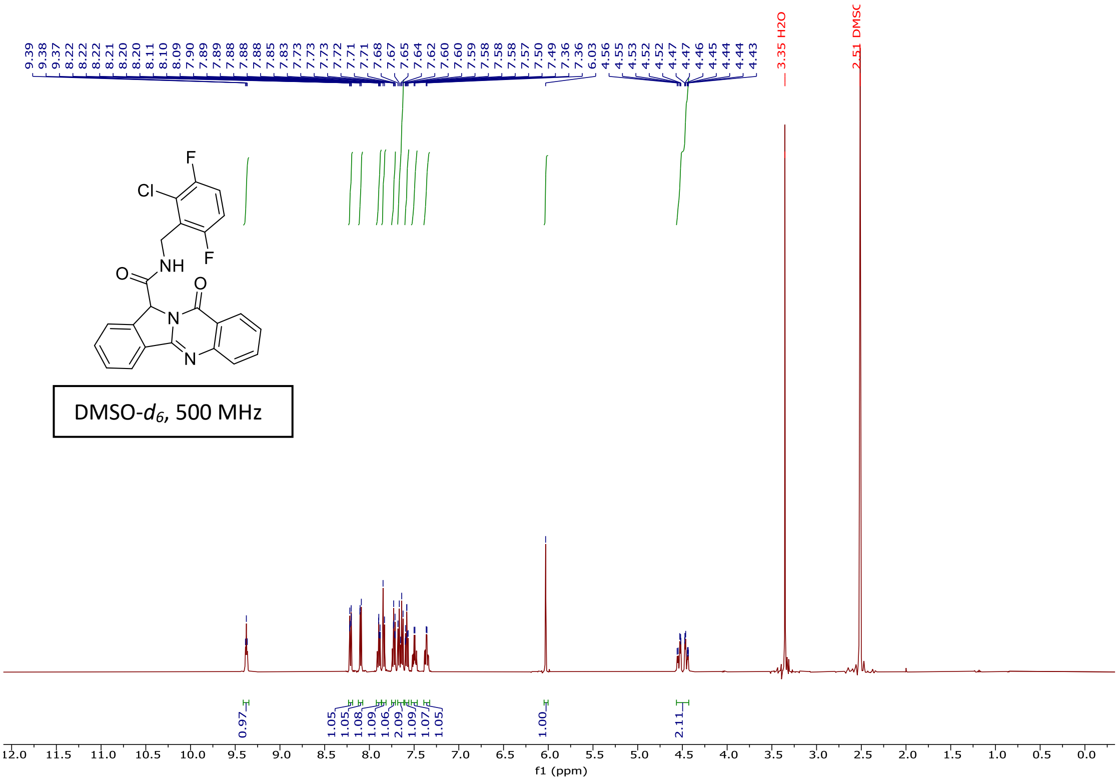

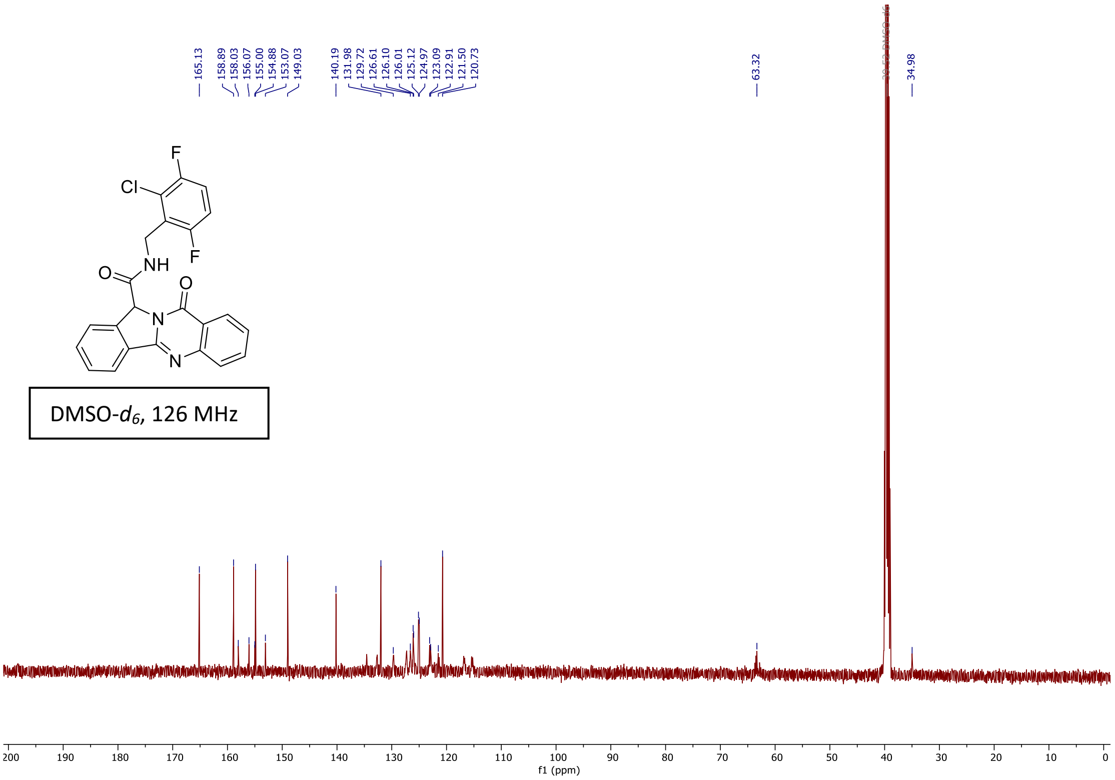

10-oxo-N-(2-(trifluoromethoxy)benzyl)-10,12-dihydroisoindolo[1,2-b]quinazoline-12-carboxamide (**24b**)

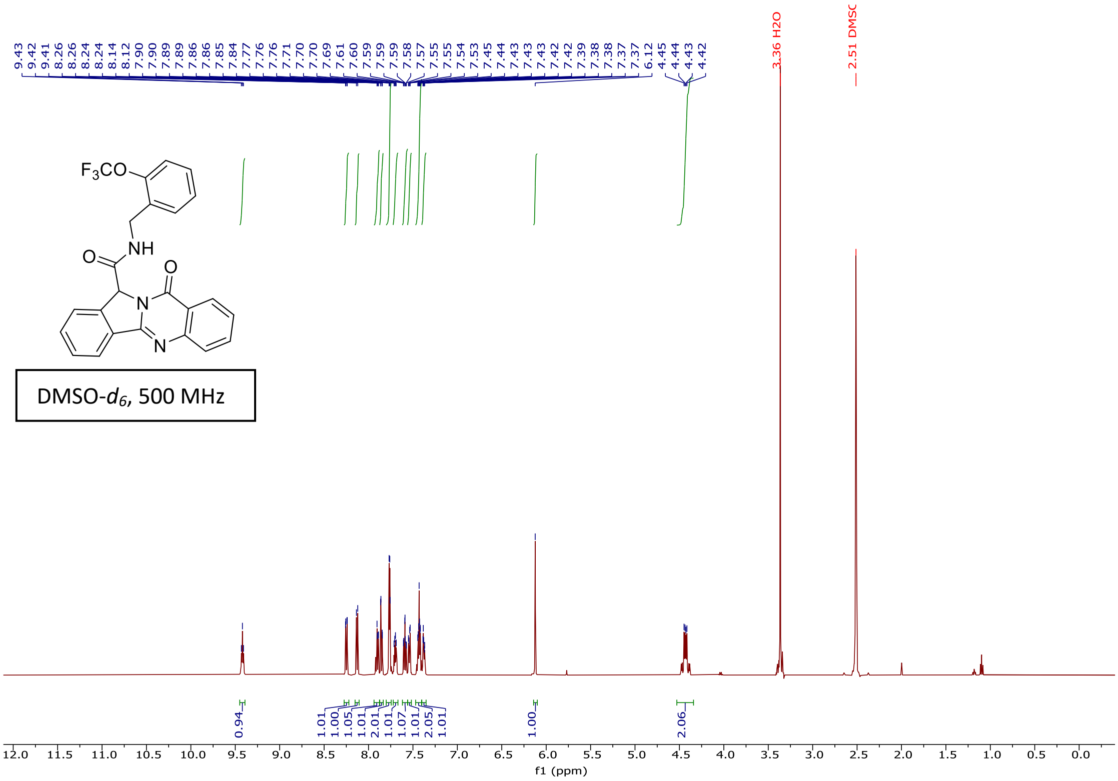

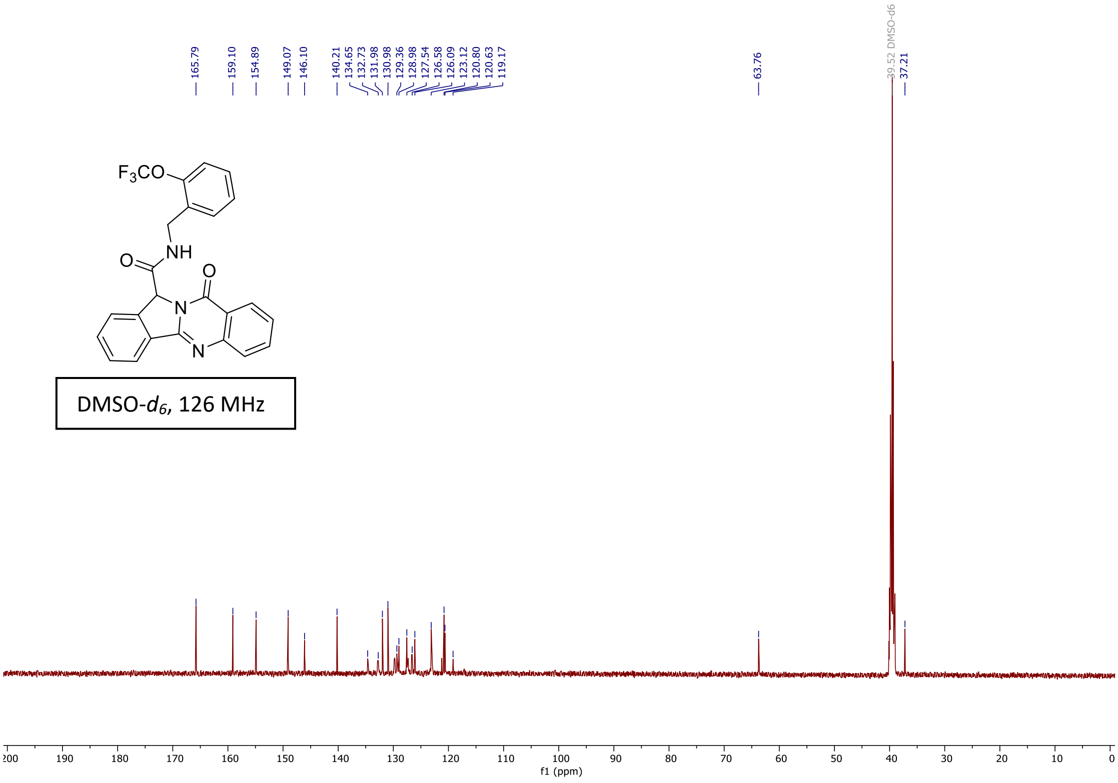

N-benzyl-7-methyl-10-oxo-10,12-dihydroisoindolo[1,2-b]quinazoline-12-carboxamide (**25b**)

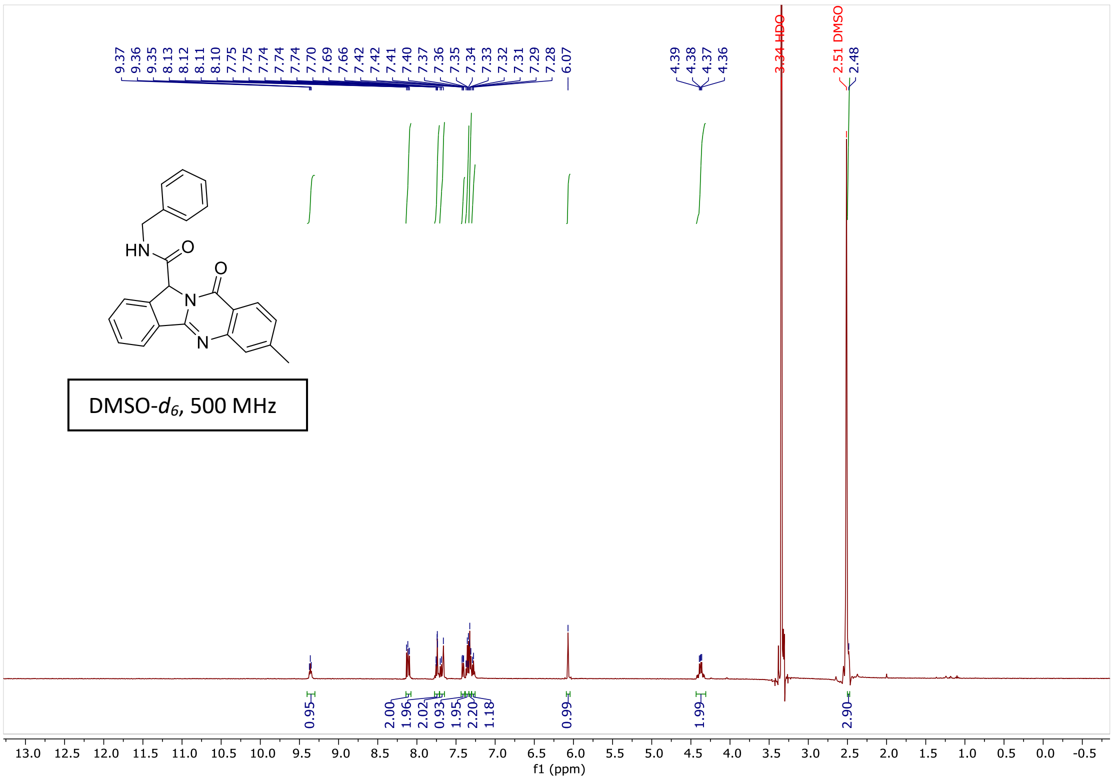

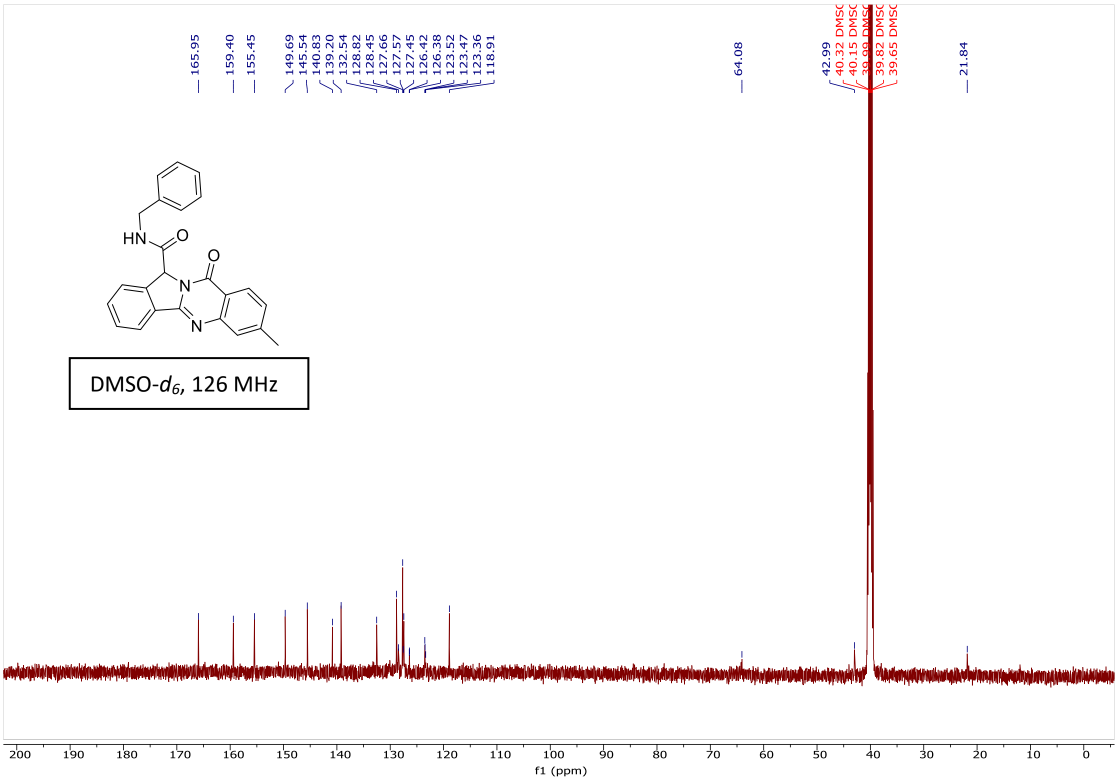

N-benzyl-4-oxo-4,6-dihydrothieno[2',3':4,5]pyrimido[2,1-a]isoindole-6-carboxamide (**26b**)

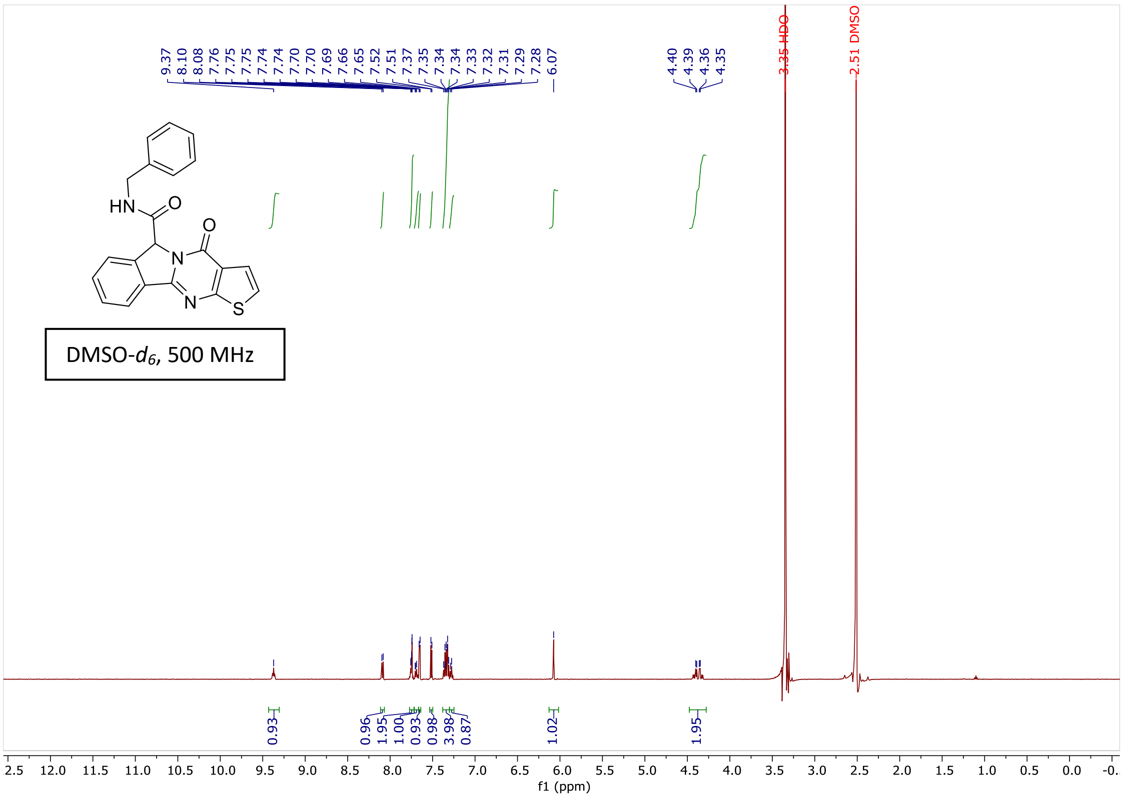

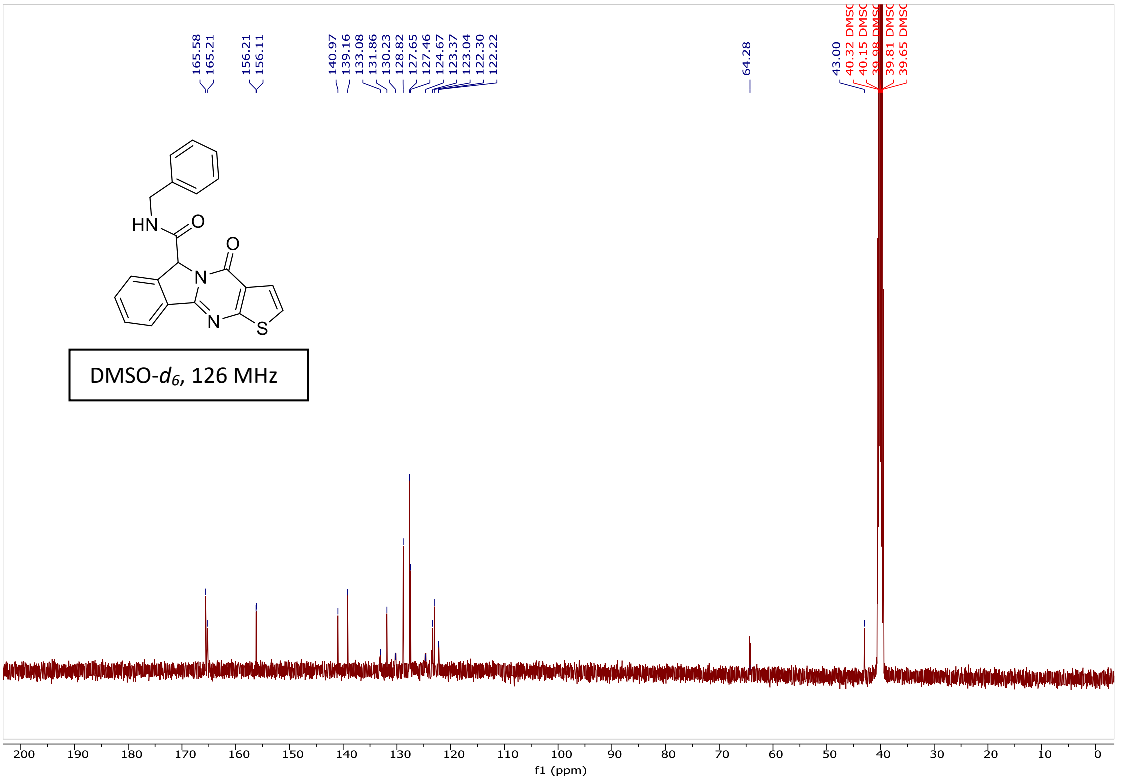

N-benzyl-7-bromo-10-oxo-10,12-dihydroisoindolo[1,2-b]quinazoline-12-carboxamide (**27b**)

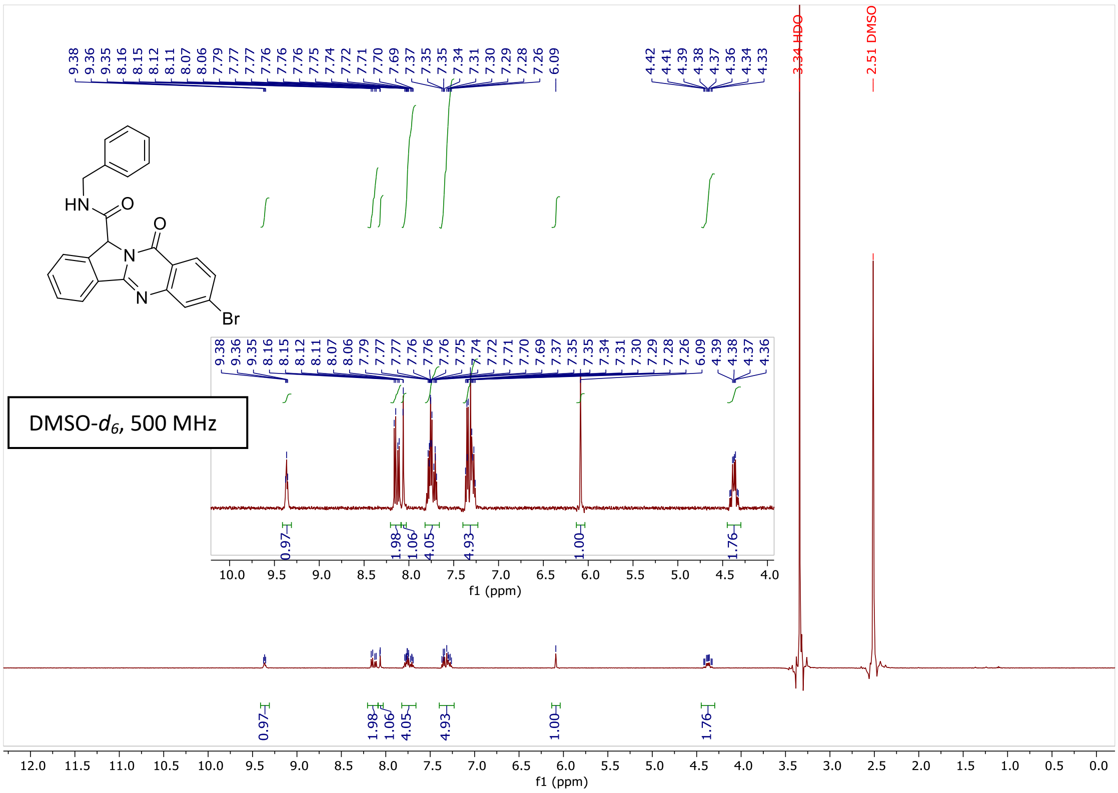

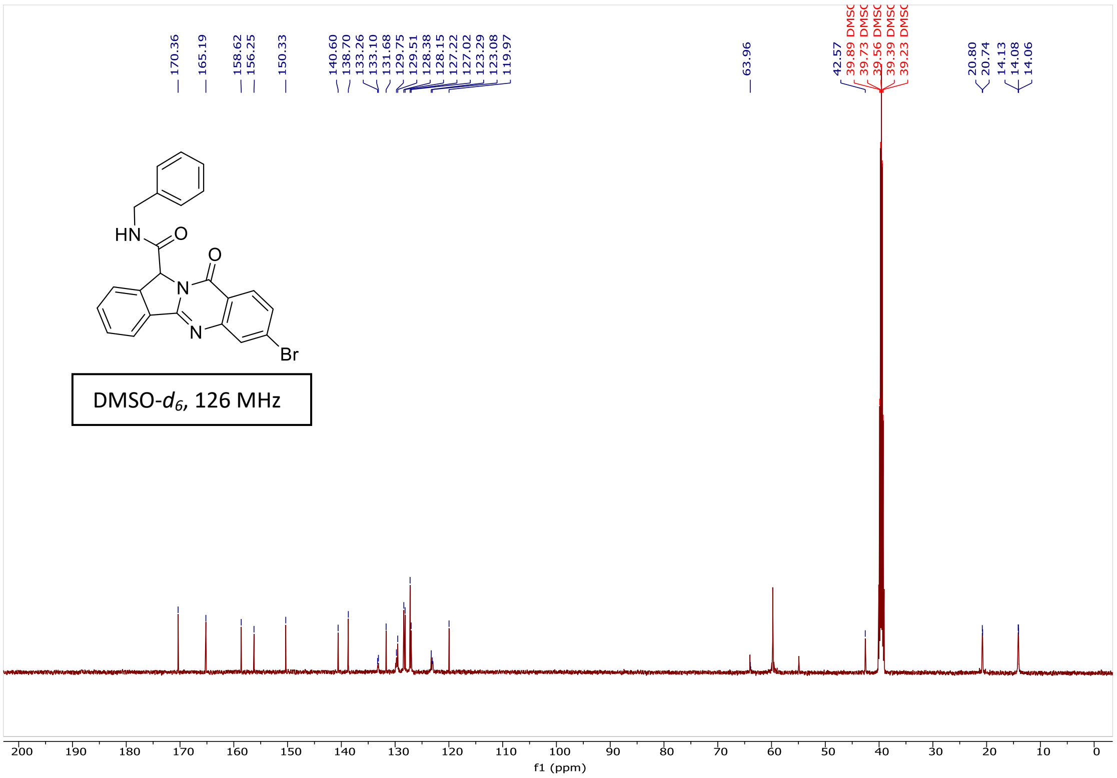

N-benzyl-8-methyl-10-oxo-10,12-dihydroisoindolo[1,2-b]quinazoline-12-carboxamide (**28b**)

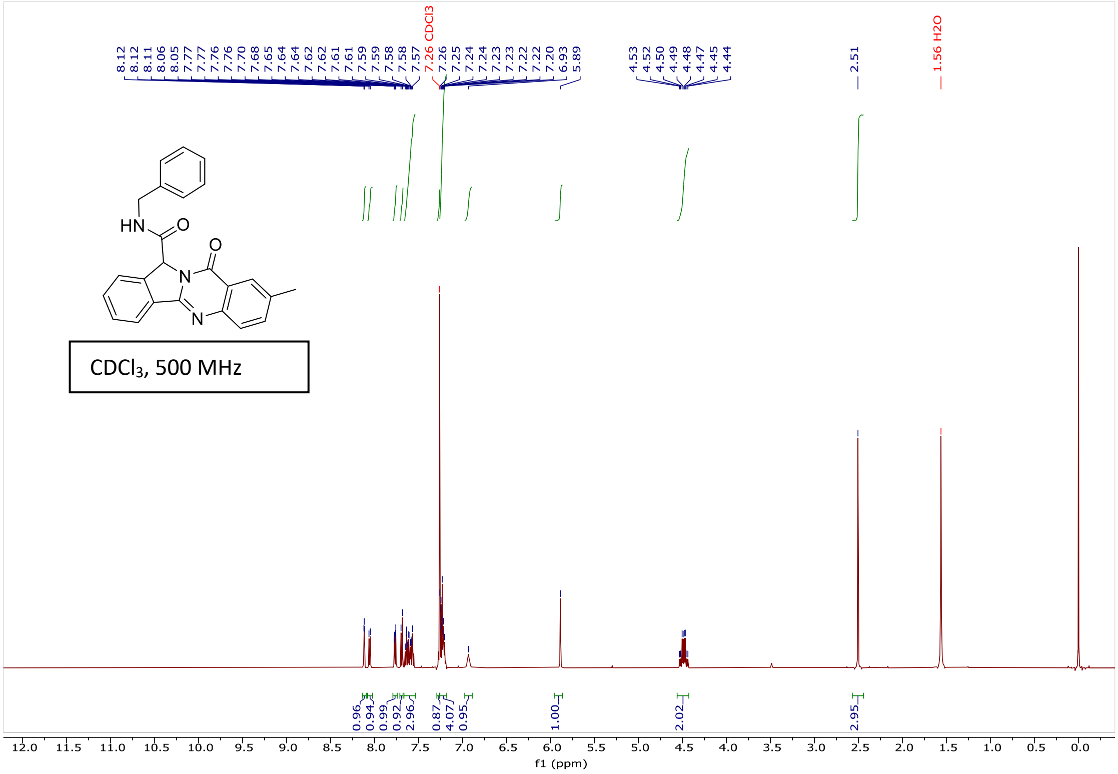

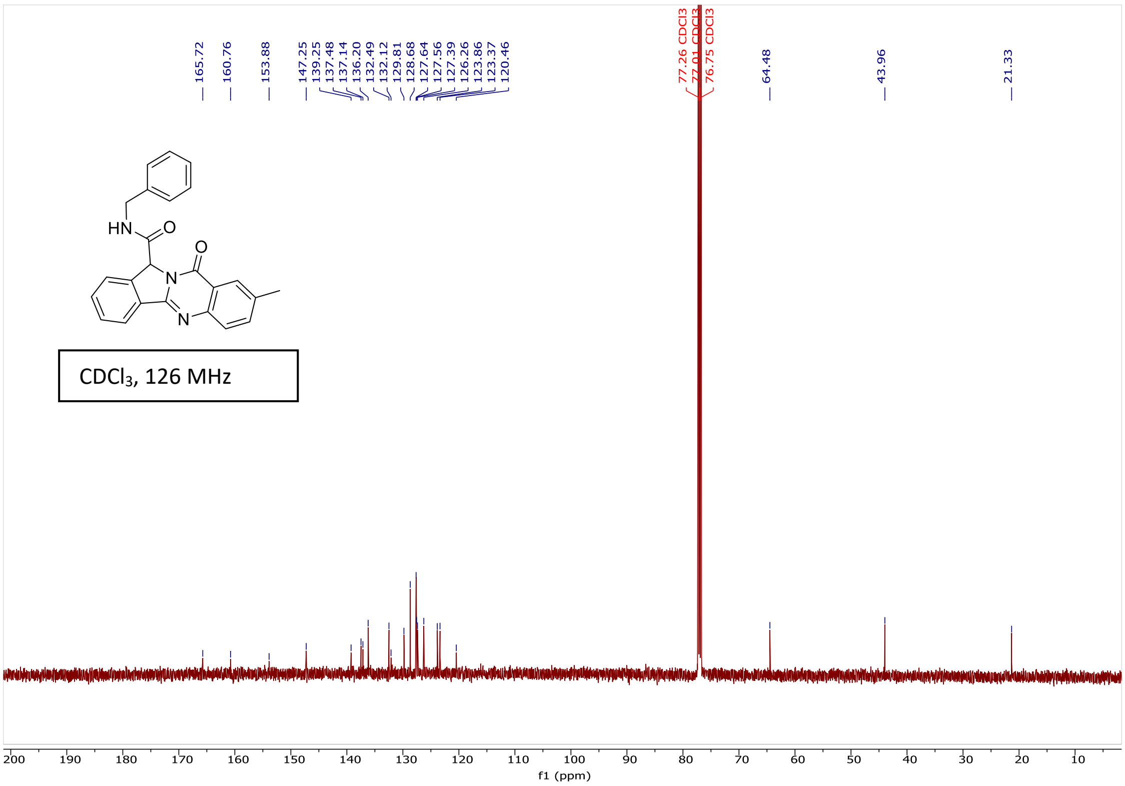

N-benzyl-12-oxo-10,12-dihydropyrido[4',3':4,5]pyrimido[2,1-a]isoindole-10-carboxamide (**29b**)

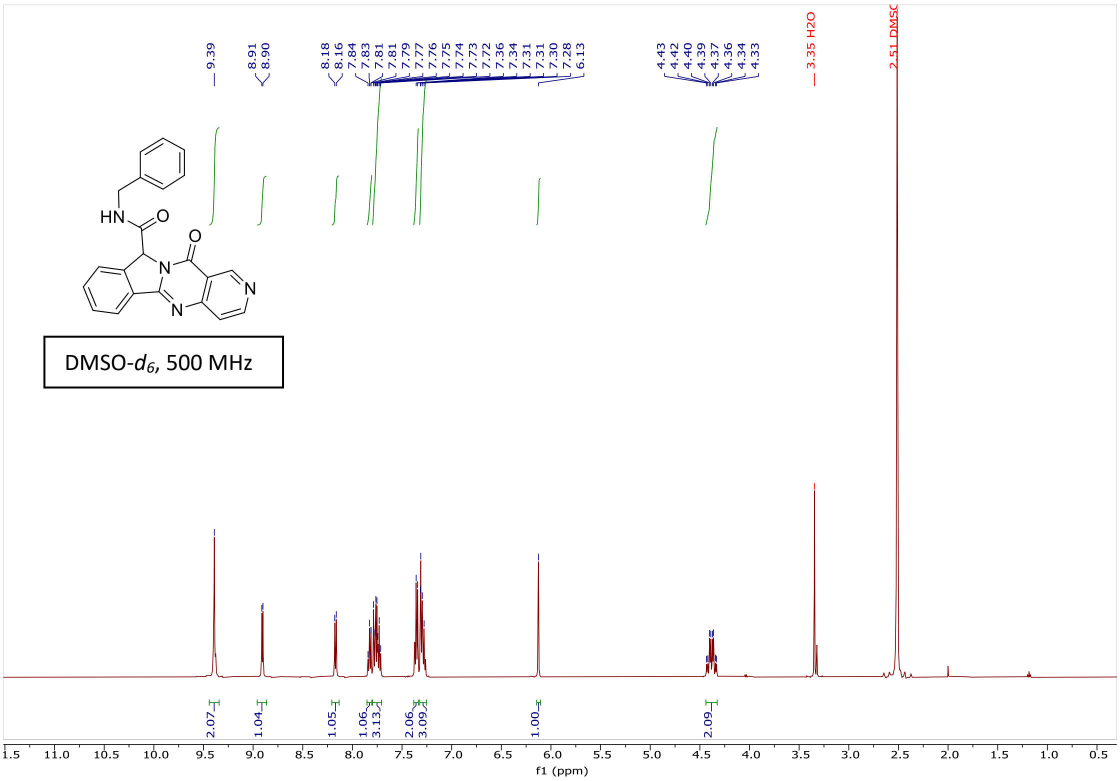

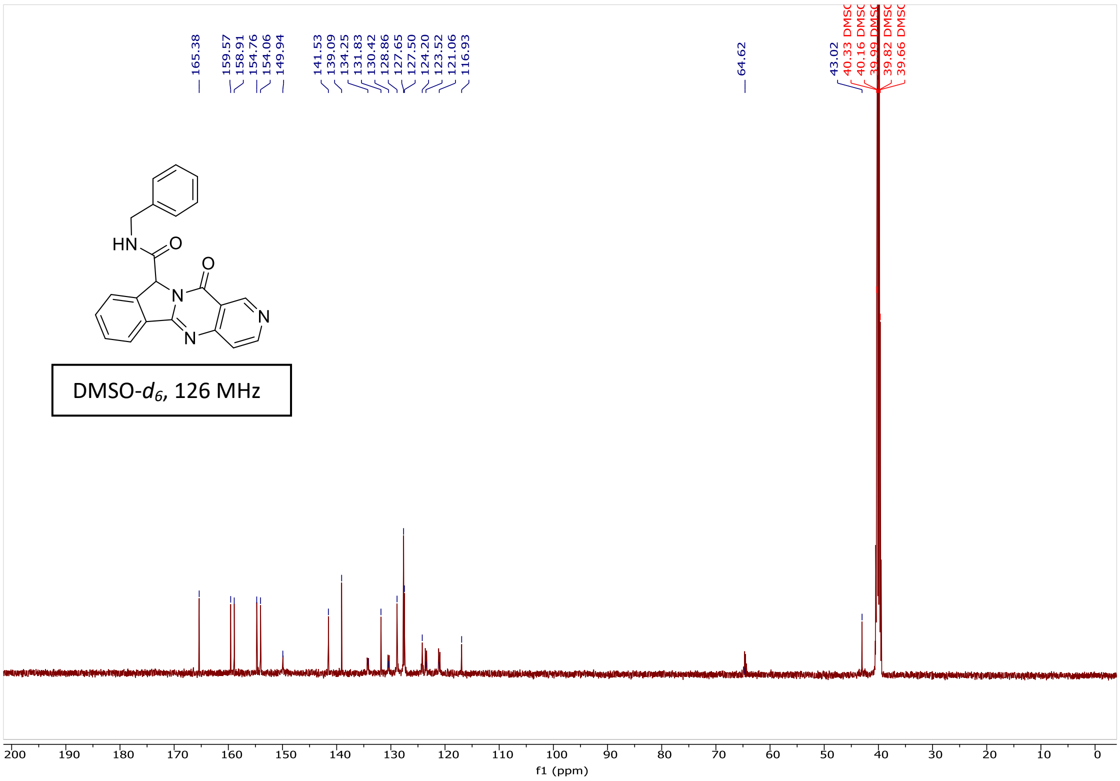

N-benzyl-7-oxo-7,9-dihydrobenzo[h]isoindolo[1,2-b]quinazoline-9-carboxamide (**30b**)

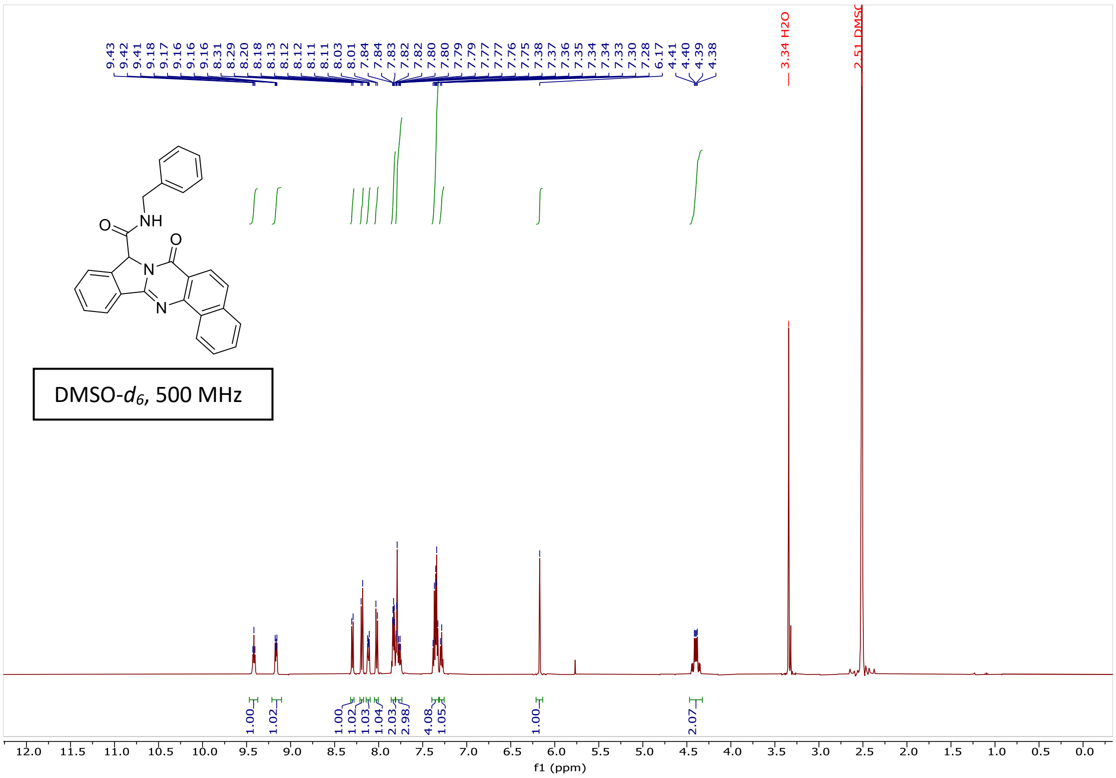

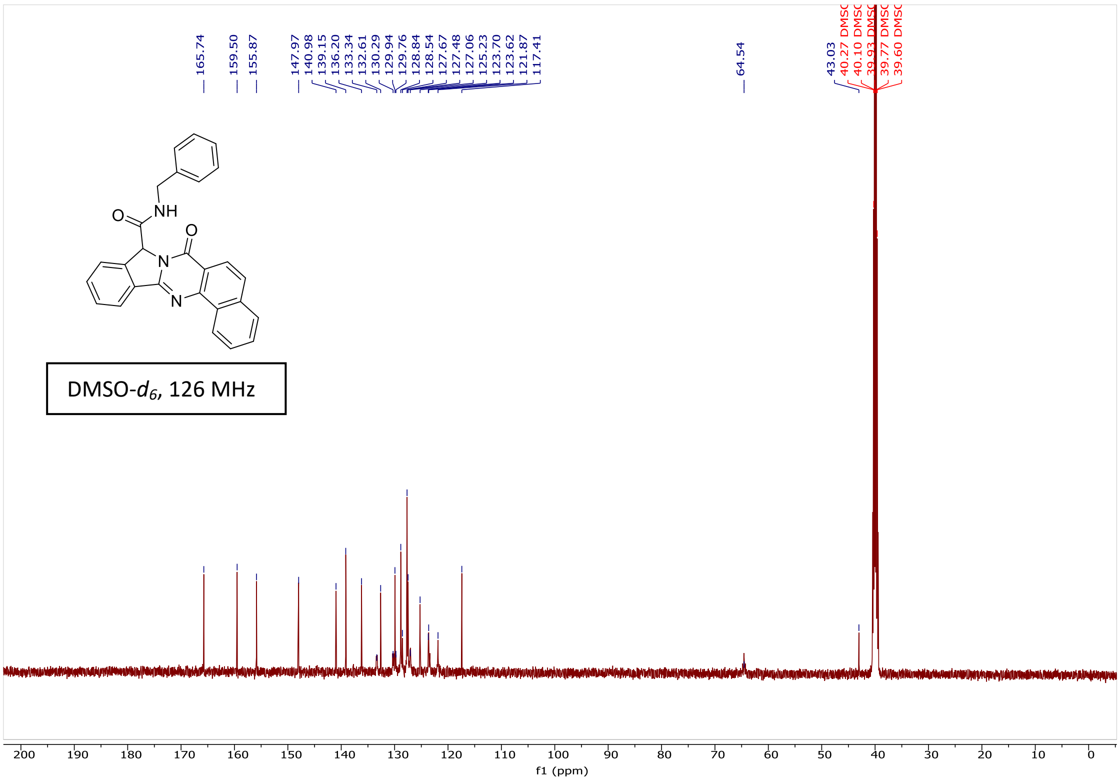

N-benzyl-5-oxo-5,7-dihydropyrido[3',4':4,5]pyrimido[2,1-a]isoindole-7-carboxamide (**31b**)

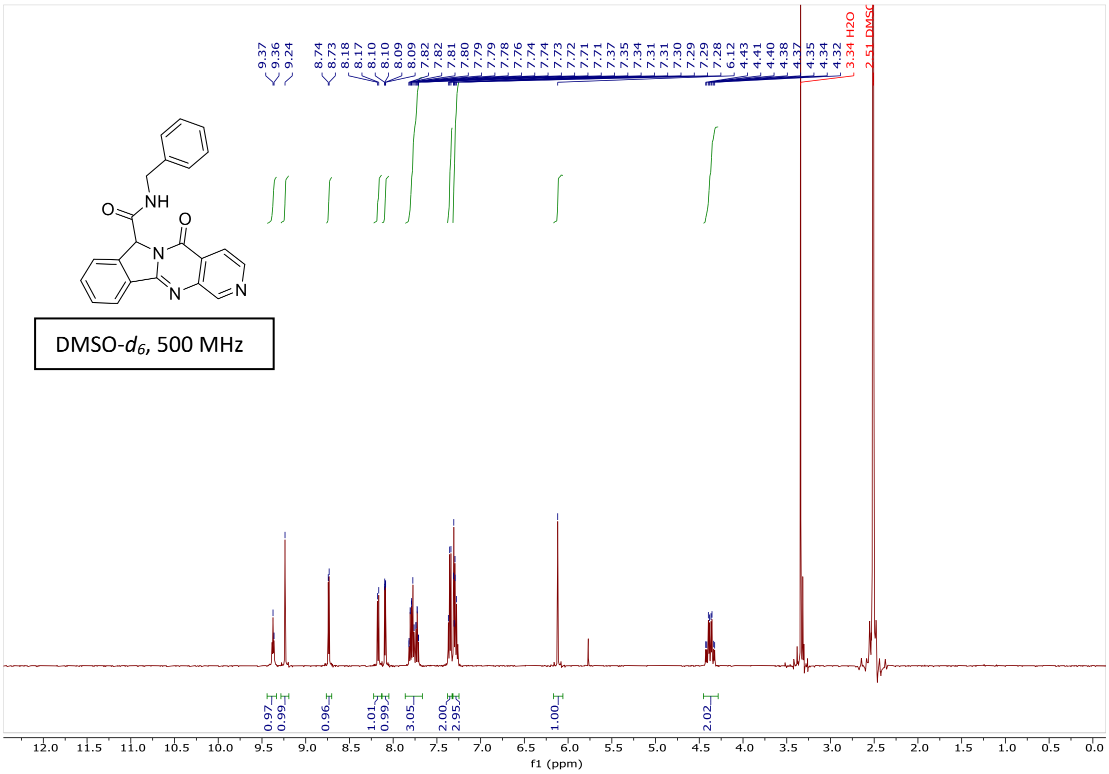

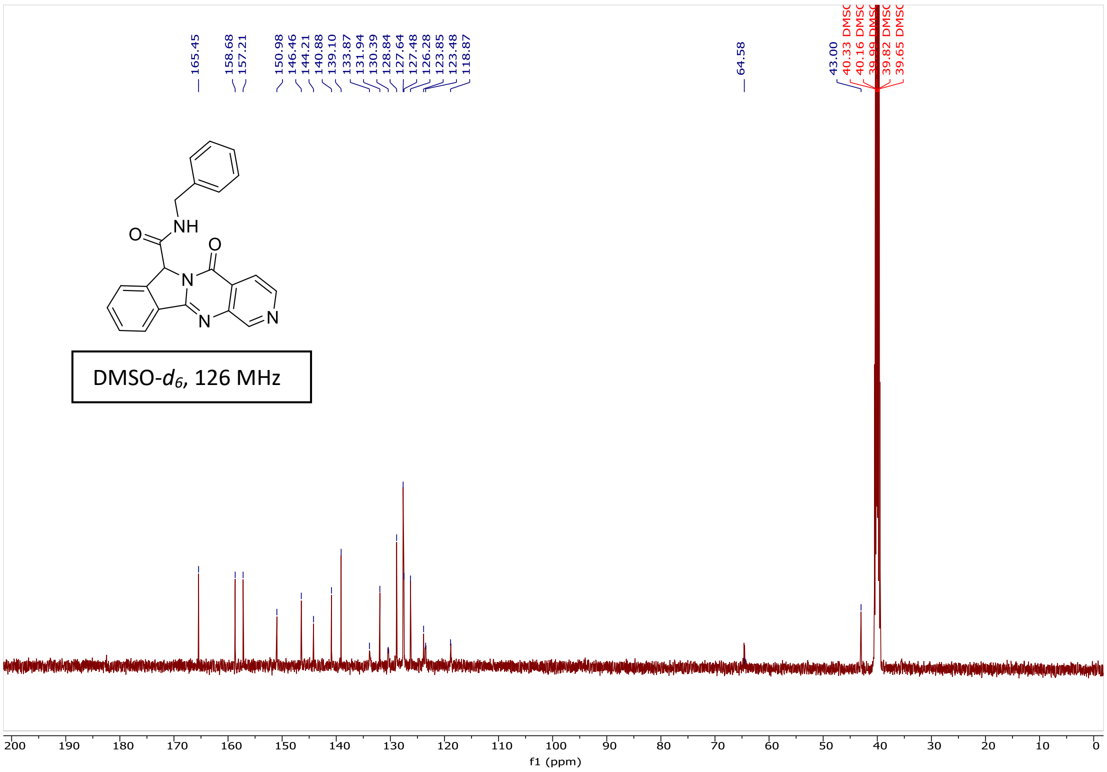

N-benzyl-3-methoxy-10-oxo-10,12-dihydroisoindolo[1,2-b]quinazoline-12-carboxamide (**32b**)

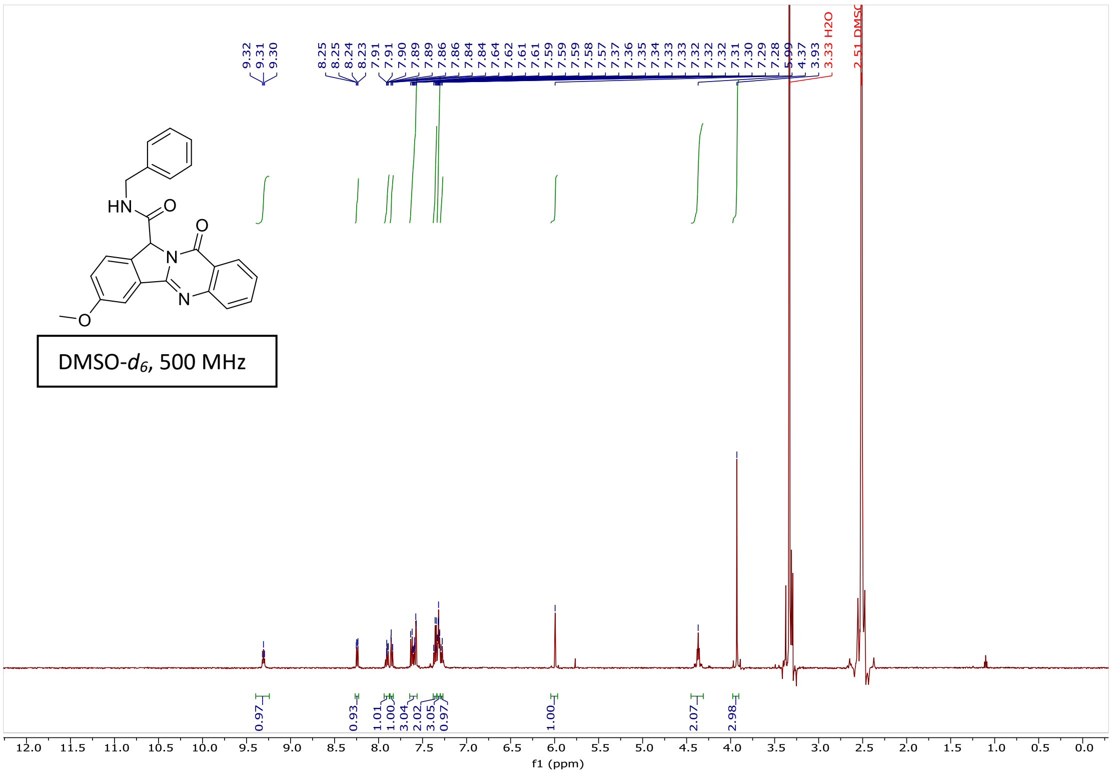

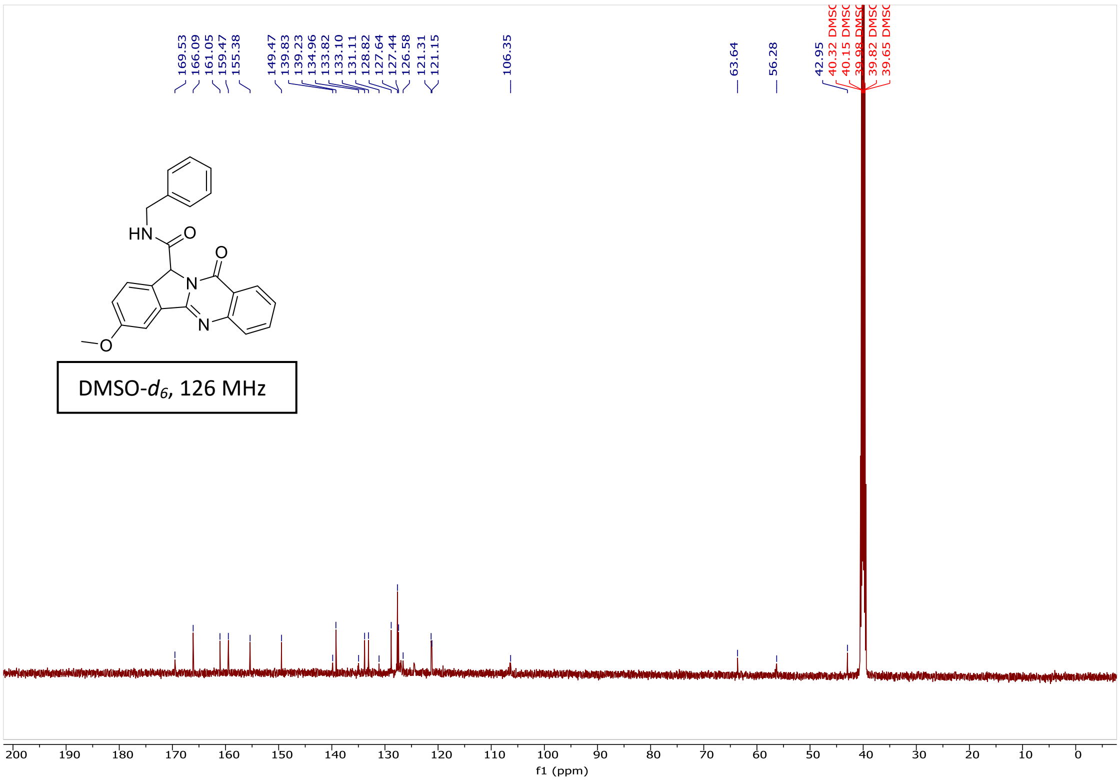

N-benzyl-3-fluoro-10-oxo-10,12-dihydroisoindolo[1,2-b]quinazoline-12-carboxamide (**33b**)

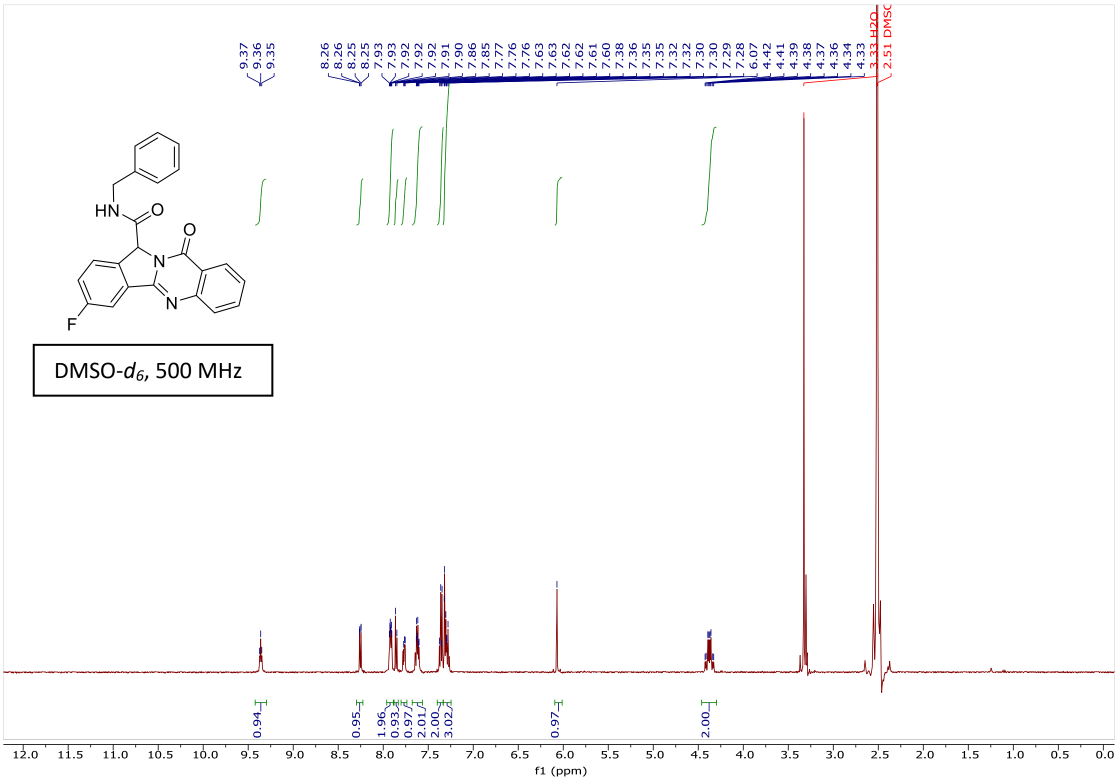

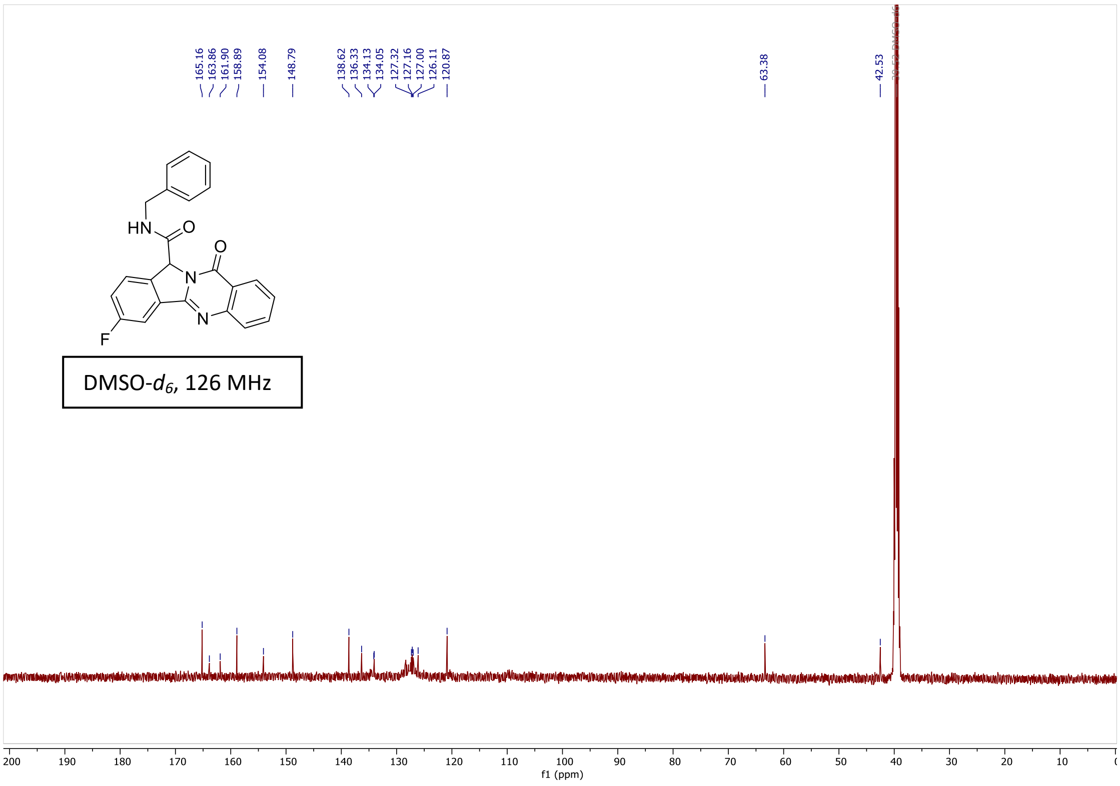

N-(2-chloro-6-fluoro-3-methylbenzyl)-10-oxo-10,12-dihydroisoindolo[1,2-b]
quinazoline-12-carboxamide (**34b**)

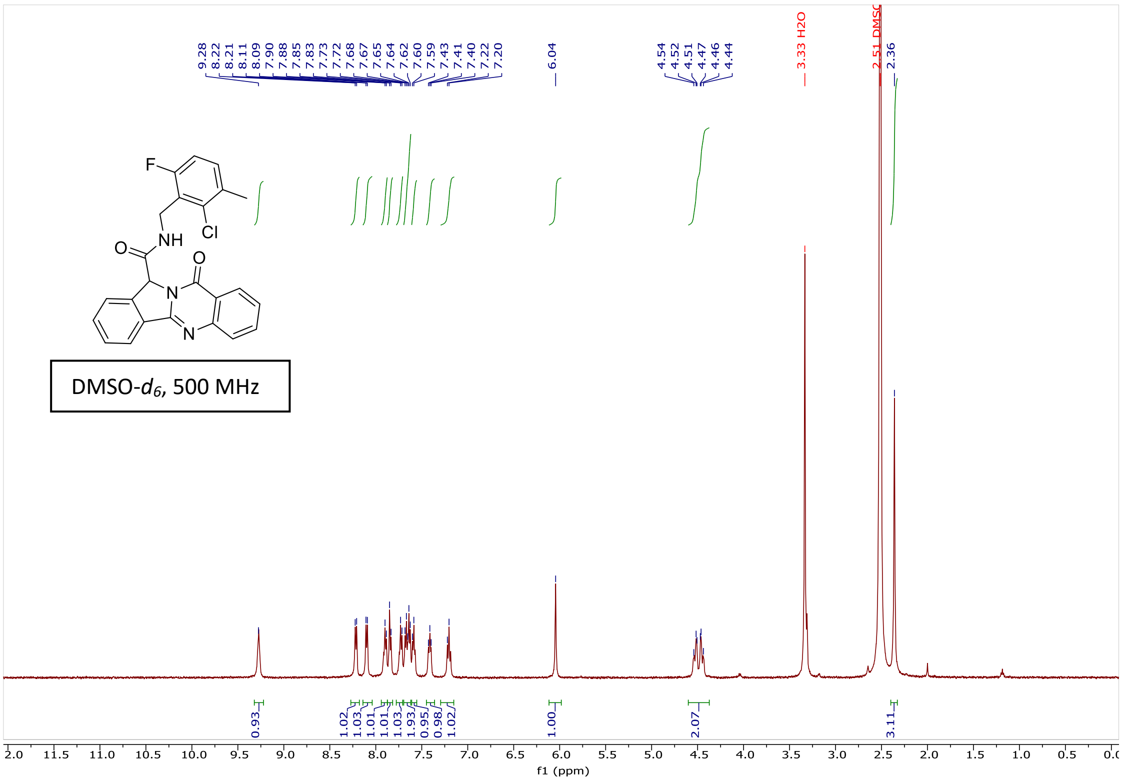

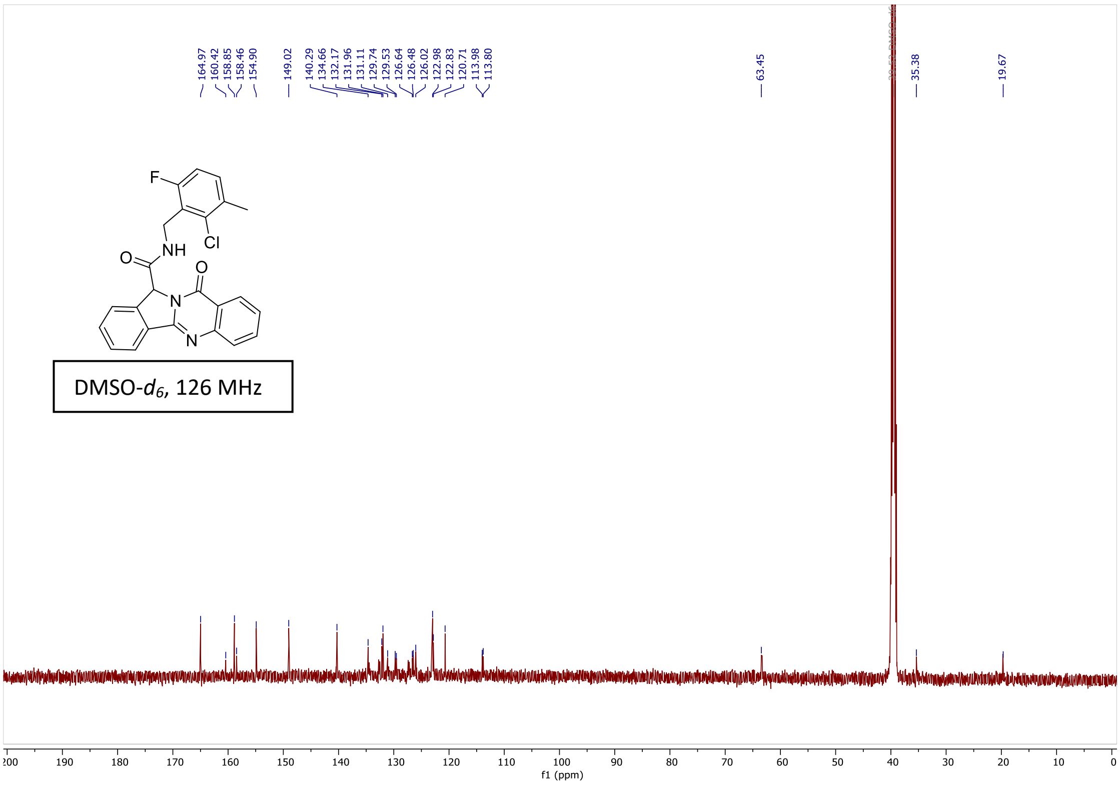

N-benzyl-11-oxo-11,13-dihydroquinolino[2',3':3,4]pyrrolo[2,1-b]quinazoline-13-carboxamide (**35b**)

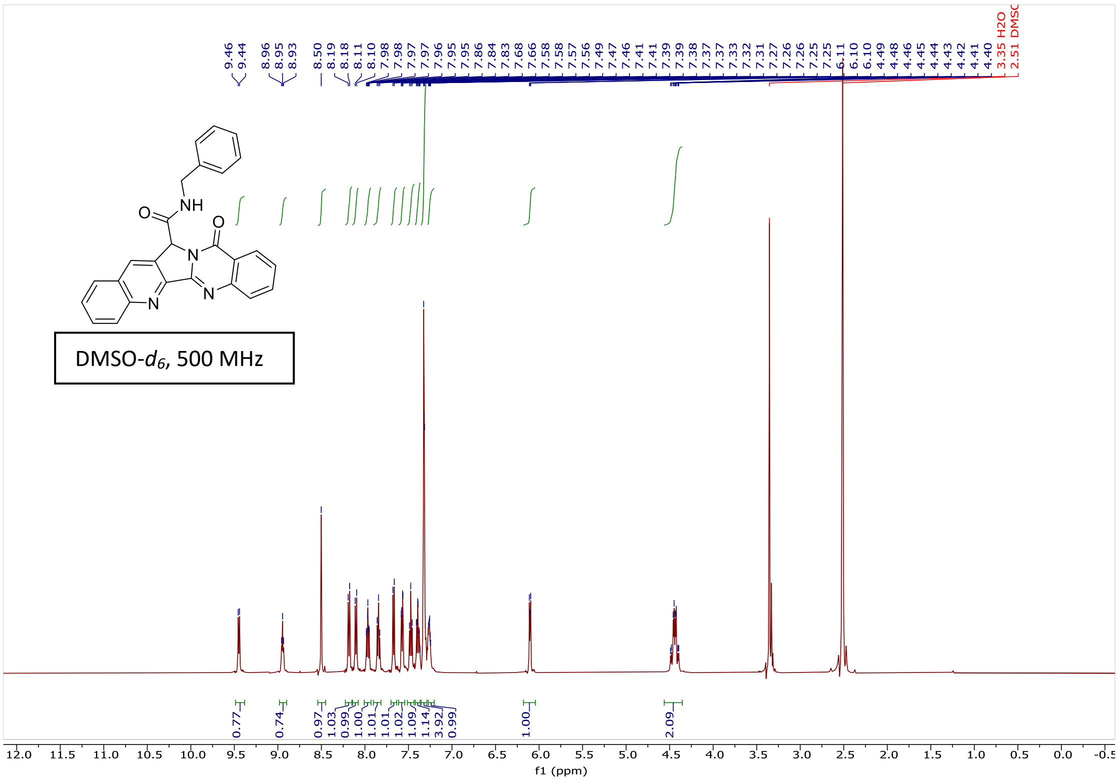

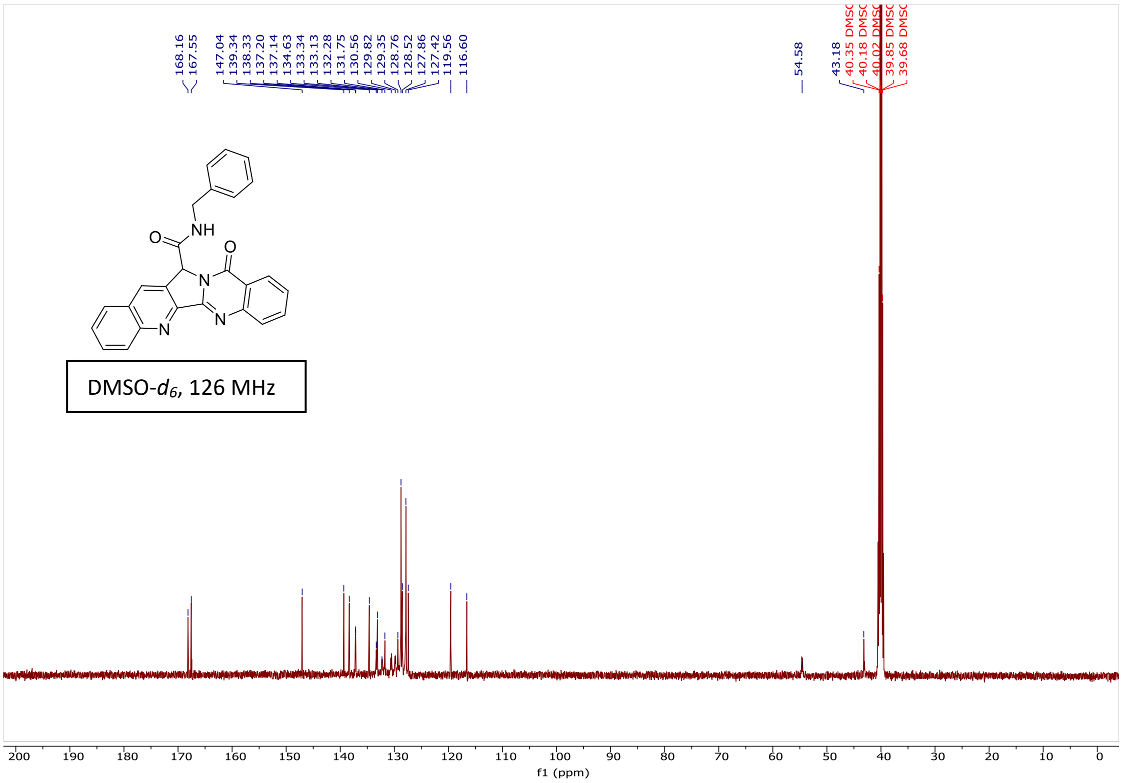

N-cyclohexyl-10-oxo-10,12-dihydroisoindolo[1,2-b]quinazoline-12-carboxamide (**39b**)

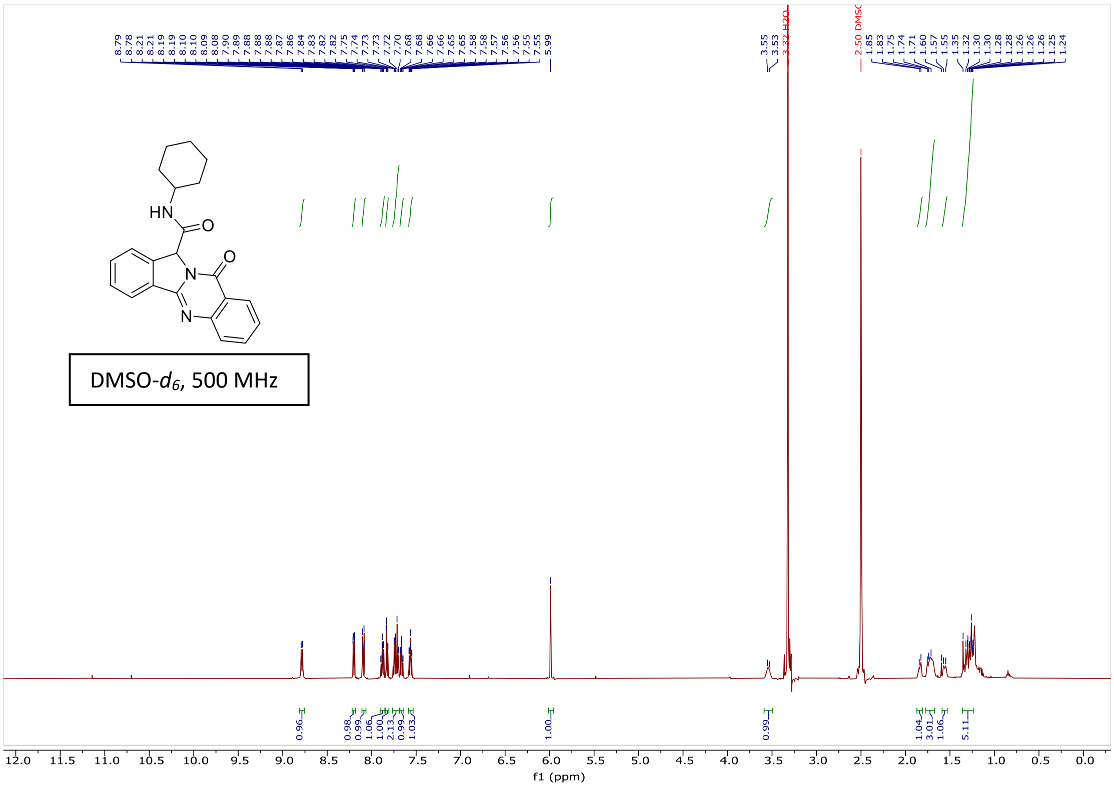

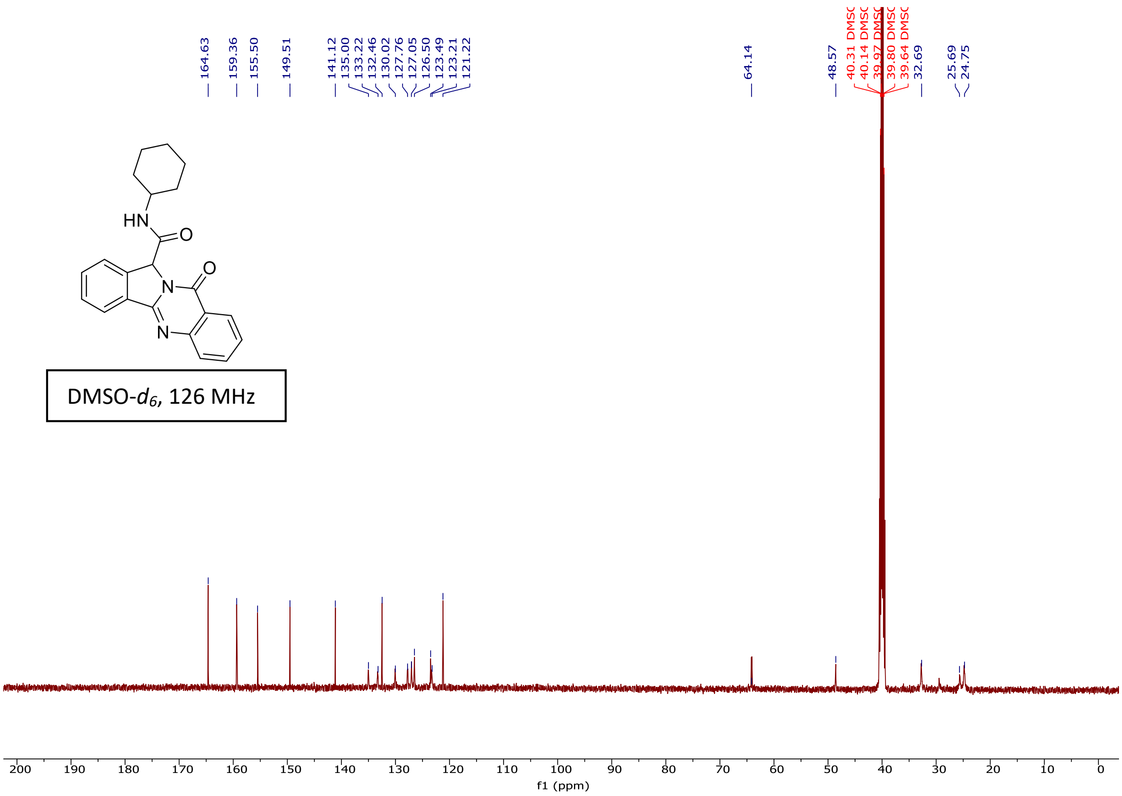

N-benzyl-10-oxo-10,12-dihydroisoindolo[1,2-b]quinazoline-12-carboxamide (**40b**)

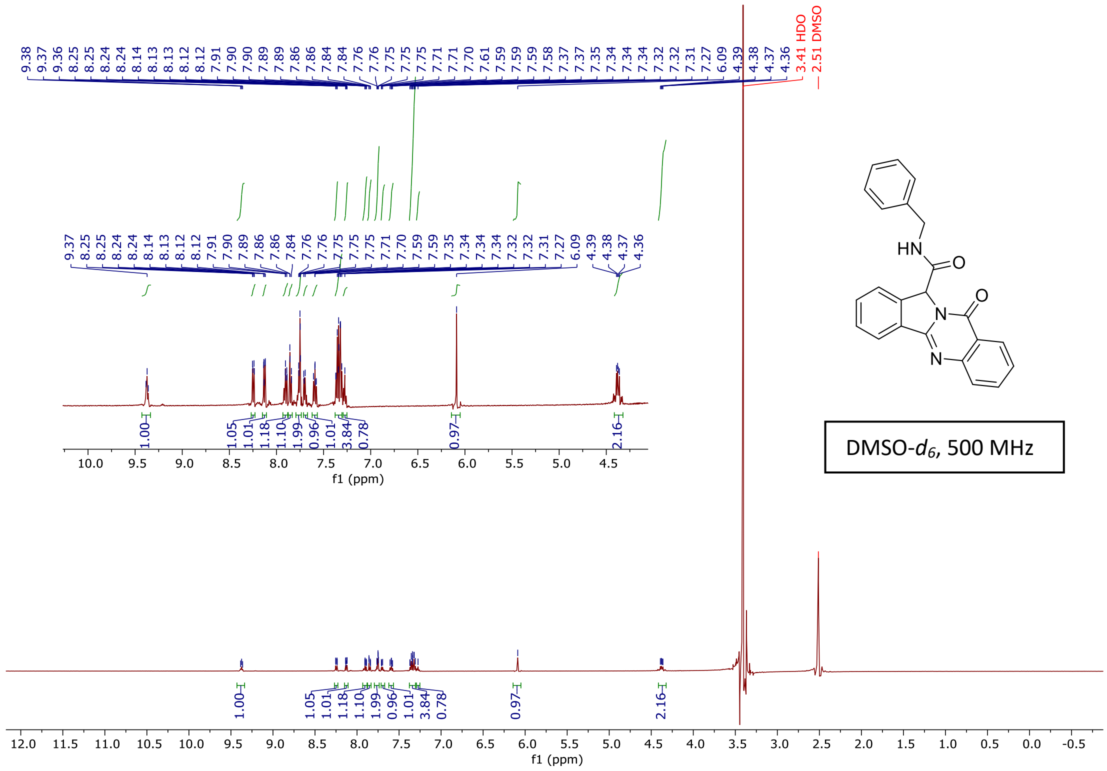

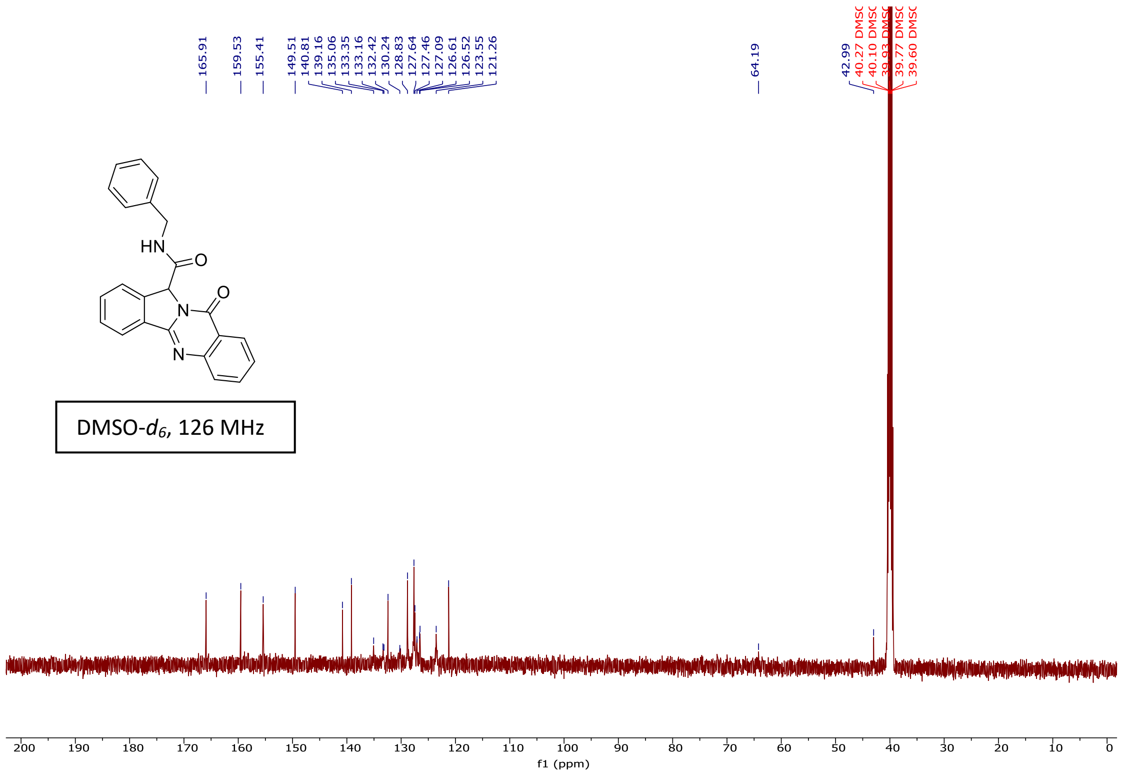

10-oxo-N-((tetrahydrofuran-2-yl)methyl)-10,12-dihydroisoindolo[1,2-b]quinazoline-12-carboxamide (**41b**)

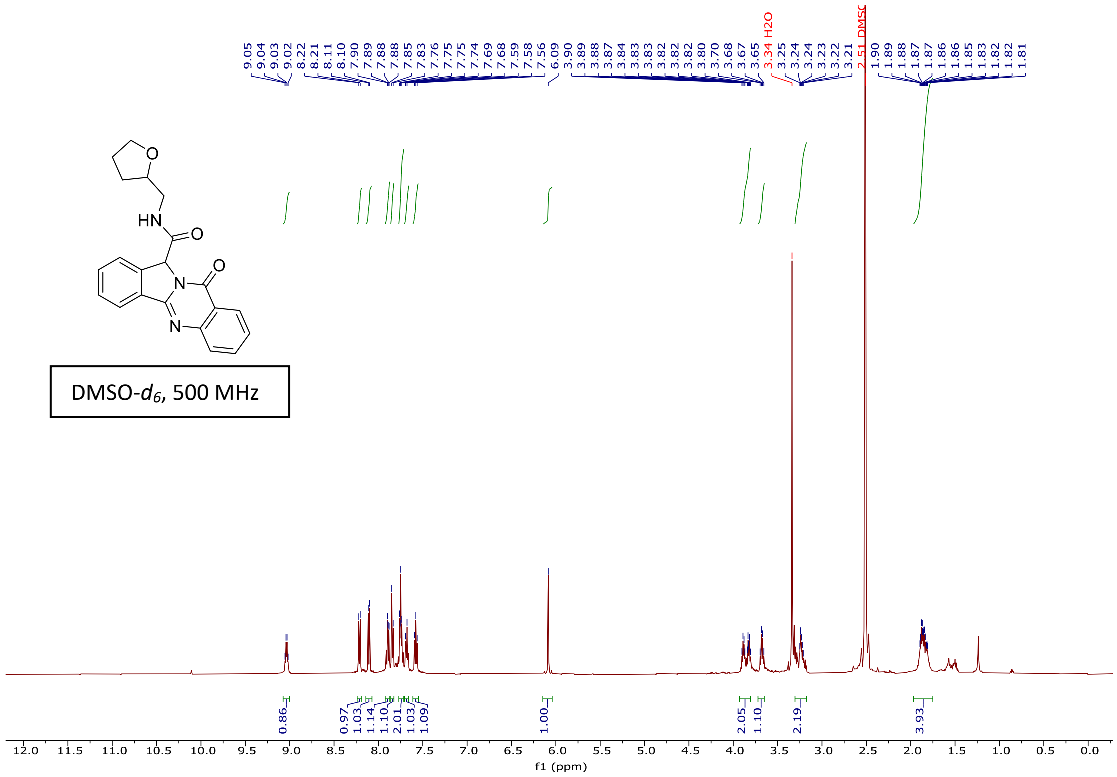
DMSO-d6, 500 MHz
f1 (ppm)

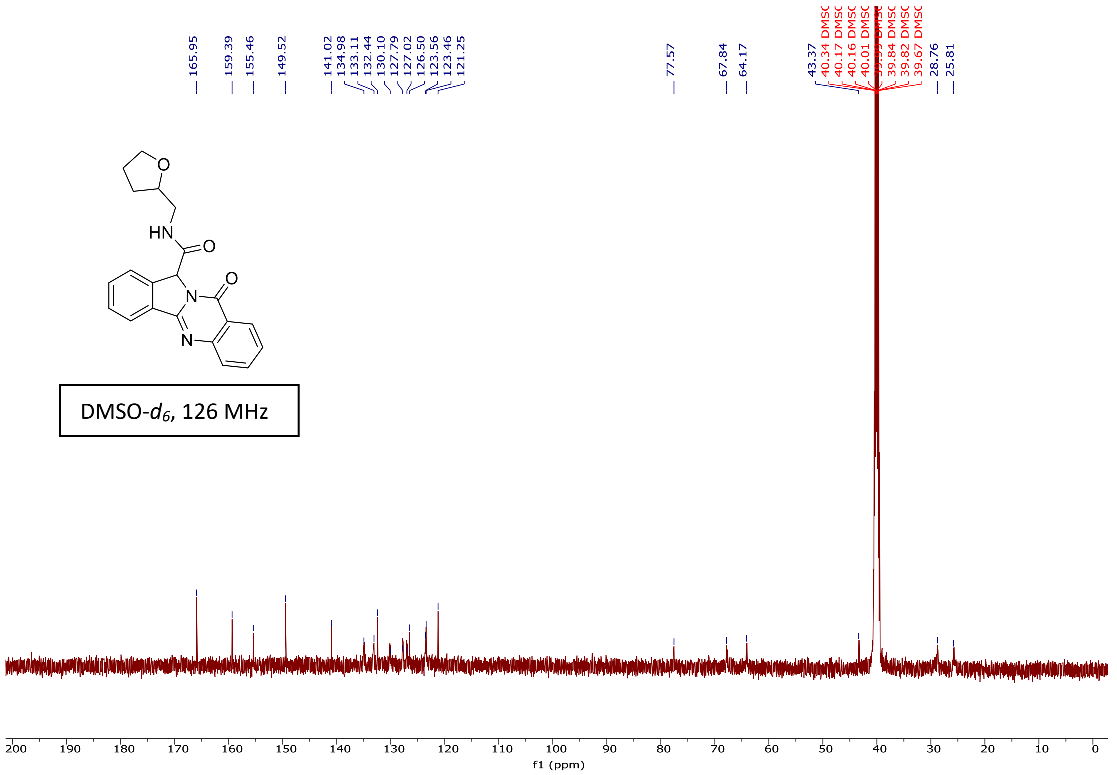
DMSO-d6, 126 MHz
f1 (ppm)
